# Supplementary material for: Navigating duplication in pharmacovigilance databases: a scoping review
Source: BMJ Open. 2024 Apr 29;14(4):e081990. doi: 10.1136/bmjopen-2023-081990 (PMC11086478; doi:10.1136/bmjopen-2023-081990)
Supplement: Supplementary data [file bmjopen-2023-081990supp002.pdf]

Appendices

Appendix I: Search strategy in PubMed

((((prevalence[tw] OR incidence[tw] OR extent[tw] OR epidemiology[tw] OR frequency[tw]) AND (duplication[tw] OR "Data Duplication"[tw] OR "Duplicate Report*"[tw] OR "Redundant Data"[tw] OR "duplicate record*"[tw] OR replication[tw])) AND ("pharmacovigilance database*"[tw] OR database*[tw]))

OR

(((prevention[tw] OR "prevention strateg*"[tw] OR cause*[tw] OR detection[tw] OR management[tw] OR "management strateg*"[tw] OR deduplication[tw]) AND (duplication[tw] OR "Data Duplication"[tw] OR "Duplicate Report*"[tw] OR "Redundant Data"[tw] OR "duplicate record*"[tw] OR replication[tw])) AND ("pharmacovigilance database*"[tw] OR database*[tw])))

OR

(((impact* OR consequence* OR effect*) AND (duplication[tw] OR "Data Duplication"[tw] OR "Duplicate Report*"[tw] OR "Redundant Data"[tw] OR "duplicate record*"[tw] OR replication[tw])) AND ("pharmacovigilance database*"[tw] OR database*[tw]))

Using the Polyglot Search Translator, we adapted the PubMed search strategy across the other peer-reviewed research databases, namely, Web of Science, Wiley Online Library and EBSCOhost.

Appendix II: Sources of grey literature search

Search terms use: Duplicate management in pharmacovigilance databases.

Source	Organizations	Website Links
London	European Medicines Agency	https://www.ema.europa.eu/en/documents/regulatory-procedural-guideline/guideline-good-pharmacovigilance-practices-gvp-module-vi-addendum-i-duplicate-management-suspected_en.pdf
India	https://allaboutpharmacovigilance.org/	https://allaboutpharmacovigilance.org/15-duplicate-search-in-pharmacovigilance/
Sweden	Uppsala	https://www.uppsalareports.org/articles/text-is-key-for-eliminating-duplicate-reports/
London	Medical literature monitoring	https://www.ema.europa.eu/en/human-regulatory/post-authorisation/pharmacovigilance/medical-literature-monitoring
London	Clarivate PLC	https://go.dialog.com/MLM_Duplication_Whitepaper/

Kiguba R, *et al. BMJ Open* 2024; 14:e081990. doi: 10.1136/bmjopen-2023-081990

London	PubHive	https://pubhive.com/pulse-literature-1/f/pubhive-automates-duplicate-detection-for-ema%E2%80%99s-mlm-service
Sweden	Uppsala	https://who-umc.org/media/3236/reporting-fact-sheet.pdf
Maryland USA	FDA	https://www.accessdata.fda.gov/scripts/cderworld/index.cfm?action=drugsafety:main&unit=1&lesson=1&topic=17
New Jersey USA	flexdatabases	https://flexdatabases.com/6-faq-pv/
New York	citeline	https://pink.citeline.com/-/media/supporting-documents/pink-sheet/2020/02/p0220ema_9.pdf
London	GOV.UK	https://assets.publishing.service.gov.uk/government/uploads/system/uploads/attachment_data/file/949102/Exceptions_and_modifications_to_the_EU_guidance_on_good_pharmacovigilance_practices_that_apply_to_UK_MAHs_v2.pdf
San Francisco	WordPress.com	https://auditandcompliance.wordpress.com/2018/12/23/basic-steps-in-pharmacovigilance-case-processing/
Pakistan	Drug Regulatory Authority of Pakistan	https://www.dra.gov.pk/wp-content/uploads/2022/04/Good-Pharmacovigilance-Guidelines-for-Registration-Holders.pdf
Sweden	Uppsala	https://www.diva-portal.org/smash/get/diva2:1670567/FULLTEXT01.pdf
Lithuania	Biomapas	https://www.biomapas.com/selecting-a-pharmacovigilance-drug-safety-database/
Erupe	ENCePP	https://www.encepp.eu/standards_and_guidances/methodologicalGuide8_4.shtml

Brussels	AFMPS	https://www.afmps.be/sites/default/files/content/faqs_ev_changes_22-11-2017_afmps-fagg.pdf
Amsterdam	European medicines agency	https://www.adrreports.eu/en/data_quality.html#:~:text=a%20report%20is%20submitted%20by,to%20the%20report%20being%20erroneous.
Amsterdam	European medicines agency	https://www.ema.europa.eu/en/documents/other/scope-training-duplicate-detection-best-practice-guide_en.pdf
Maryland USA	FDA	https://www.fda.gov/drugs/surveillance/questions-and-answers-fdas-adverse-event-reporting-system-faers
New York	IEEE	https://ieeexplore.ieee.org/abstract/document/6732591/similar#similar

Appendix III: Data extraction instrument

Auth or(s)	Jou rnal	Ti tle	Speci fic Objec tives	Cou ntry	Stu dy des ign	Year of public ation	Ext ent	Preve ntion	Ca use s	Dete ction	Manag ement	Implic ations	Key find ings

Appendix IV: Regions and countries

Region	Number of Publications	Countries
Asia Pacific	6 publications	China<br>India<br>Malaysia<br>South Korea
Europe	27 publications	Sweden<br>Georgia<br>Switzerland<br>Netherlands<br>England<br>Italy<br>Hungary<br>Switzerland<br>Croatia<br>Germany<br>Poland<br>Portugal
Australia	3 publications	Australia
North America	22 publications	USA

Kiguba R, *et al. BMJ Open* 2024; 14:e081990. doi: 10.1136/bmjopen-2023-081990

Appendix V: List of 98 articles excluded on full-text review

No.	PMID	Title	Citation	First Author	Journal/Book	Publication Year
1	22360774	Pharmacovigilance	Br J Clin Pharmacol. 2012 Jun;73(6):979-82. doi: 10.1111/j.1365-2125.2012.04249.x.	Edwards IR	Br J Clin Pharmacol	2012
2	25123728	"Big data" and the electronic health record	Yearb Med Inform. 2014 Aug 15;9(1):97-104. doi: 10.15265/IY-2014-0003.	Ross MK	Yearb Med Inform	2014
3	33634545	Suitability of databases in the Asia-Pacific for collaborative monitoring of vaccine safety	Pharmacoepidemiol Drug Saf. 2021 Jul;30(7):843-857. doi: 10.1002/pds.5214. Epub 2021 Mar 23.	Duszynski KM	Pharmacoepidemiol Drug Saf	2021
4	35179704	High-Dimensional Propensity Score-Adjusted Case-Crossover for Discovering Adverse Drug Reactions from Computerized Administrative Healthcare Databases	Drug Saf. 2022 Mar;45(3):275-285. doi: 10.1007/s40264-022-01148-5. Epub 2022 Feb 18.	Volatier E	Drug Saf	2022
5	29474173	Possibility of Database Research as a Means of Pharmacovigilance in Japan Based on a Comparison with Sertraline Postmarketing Surveillance	Value Health Reg Issues. 2018 May;15:1-5. doi: 10.1016/j.vhri.2017.05.002. Epub 2017 Jun 20.	Hirano Y	Value Health Reg Issues	2018
6	32243569	Different Strategies to Execute Multi-Database Studies for Medicines Surveillance in Real-World Setting: A Reflection on the European Model	Clin Pharmacol Ther. 2020 Aug;108(2):228-235. doi: 10.1002/cpt.1833. Epub 2020 May 5.	Gini R	Clin Pharmacol Ther	2020
7	31464008	Establishment of the MID-NET(®) medical information database network as a reliable and valuable database for drug safety assessments in Japan	Pharmacoepidemiol Drug Saf. 2019 Oct;28(10):1395-1404. doi: 10.1002/pds.4879. Epub 2019 Aug 29.	Yamaguchi M	Pharmacoepidemiol Drug Saf	2019
8	31933254	Artificial Intelligence Within Pharmacovigilance: A Means to Identify Cognitive Services and the Framework for Their Validation	Pharmaceut Med. 2019 Apr;33(2):109-120. doi: 10.1007/s40290-019-00269-0.	Mockute R	Pharmaceut Med	2019
9	35239736	Evaluation of pharmacovigilance systems for reporting medication errors in Africa and the role of patients using a mixed-methods approach	PLoS One. 2022 Mar 3;17(3):e0264699. doi: 10.1371/journal.pone.0264699. eCollection 2022.	Sabblah GT	PLoS One	2022
10	30445979	Organizational capacities of national pharmacovigilance centres in Africa: assessment of resource elements associated with successful and unsuccessful	Global Health. 2018 Nov 16;14(1):109. doi: 10.1186/s12992-018-0431-0.	Ampadu HH	Global Health	2018
11	36097239	Artificial Intelligent Context-Aware Machine-Learning Tool to Detect Adverse Drug Events from Social Media Platforms	J Med Toxicol. 2022 Oct;18(4):311-320. doi: 10.1007/s13181-022-00906-2. Epub 2022 Sep 12.	Roosan D	J Med Toxicol	2022
12	29714535	An Alternative to Disproportionality: A Frequency-Based Method for Pharmacovigilance Data Mining	Ther Innov Regul Sci. 2018 May;52(3):294-299. doi: 10.1177/2168479017728986. Epub 2017 Sep 8.	Jokinen JD	Ther Innov Regul Sci	2018
13	30185579	Electronic healthcare databases in Europe: descriptive analysis of characteristics and potential for use in medicines regulation	BMJ Open. 2018 Sep 5;8(9):e023090. doi: 10.1136/bmjopen-2018-023090.	Pacurariu A	BMJ Open	2018
14	32026758	Combining big data search analytics and the FDA Adverse Event Reporting System database to detect a potential safety signal of mirtazapine abuse	Health Informatics J. 2020 Sep;26(3):2265-2279. doi: 10.1177/1460458219901232. Epub 2020 Feb 6.	Spachos D	Health Informatics J	2020
15	23512870	Drug safety data mining with a tree-based scan statistic	Pharmacoepidemiol Drug Saf. 2013 May;22(5):517-23. doi: 10.1002/pds.3423. Epub 2013 Mar 20.	Kulldorff M	Pharmacoepidemiol Drug Saf	2013
16	25151493	Text mining for adverse drug events: the promise, challenges, and state of the art	Drug Saf. 2014 Oct;37(10):777-90. doi: 10.1007/s40264-014-0218-z.	Harpaz R	Drug Saf	2014
17	26606038	Predictive modeling of structured electronic health records for adverse drug event detection	BMC Med Inform Decis Mak. 2015;15 Suppl 4(Suppl 4):S1. doi: 10.1186/1472-6947-15-S4-S1. Epub 2015 Nov 25.	Zhao J	BMC Med Inform Decis Mak	2015
18	24599513	Reducing the noise in signal detection of adverse drug reactions by standardizing the background: a pilot study on analyses of proportional reporting ratios-by-therapeutic area	Eur J Clin Pharmacol. 2014 May;70(5):627-35. doi: 10.1007/s00228-014-1658-1. Epub 2014 Mar 7.	Grundmark B	Eur J Clin Pharmacol	2014
19	36380085	A real-world pharmacovigilance study of FDA Adverse Event Reporting System (FAERS) events for osimertinib	Sci Rep. 2022 Nov 15;12(1):19555. doi: 10.1038/s41598-022-23834-1.	Yin Y	Sci Rep	2022
20	31677951	ADVANCE: Towards near real-time monitoring of vaccination coverage, benefits and risks using European electronic health record databases	Vaccine. 2020 Dec 22;38 Suppl 2:B76-B83. doi: 10.1016/j.vaccine.2019.08.012. Epub 2019 Oct 31.	Bollaerts K	Vaccine	2020
21	21219405	Using primary care prescribing databases for pharmacovigilance	Br J Clin Pharmacol. 2011 Feb;71(2):244-9. doi: 10.1111/j.1365-2125.2010.03816.x.	Mohamed IN	Br J Clin Pharmacol	2011
22	35691583	Development and external validation of a 1- and 5-year fracture prediction tool based on electronic medical records data: The EPIC risk algorithm	Bone. 2022 Sep;162:116469. doi: 10.1016/j.bone.2022.116469. Epub 2022 Jun 9.	Tebé C	Bone	2022
23	31198563	Pharmacovigilance systems in resource-limited settings: an evaluative case study of Sierra Leone	J Pharm Policy Pract. 2019 Jun 11;12:13. doi: 10.1186/s40545-019-0173-2. eCollection 2019.	Abiri OT	J Pharm Policy Pract	2019
24	25123721	Big data - smart health strategies. Findings from the yearbook 2014 special theme	Yearb Med Inform. 2014 Aug 15;9(1):48-51. doi: 10.15265/IY-2014-0031.	Koutkias V	Yearb Med Inform	2014

No.	PMID	Title	Citation	First Author	Journal/Book	Publication Year
25	36147337	Analysis of pharmacovigilance databases for spontaneous reports of adverse drug reactions related to substandard and falsified medical products: A descriptive study	Front Pharmacol. 2022 Sep 6;13:964399. doi: 10.3389/fphar.2022.964399. eCollection 2022.	Pozsgai K	Front Pharmacol	2022
26	23331229	Automated method for detecting increases in frequency of spontaneous adverse event reports over time	J Biopharm Stat. 2013;23(1):161-77. doi: 10.1080/10543406.2013.736809.	DuMouchel W	J Biopharm Stat	2013
27	26819727	Pharmacoepidemiology in Japan: medical databases and research achievements	J Pharm Health Care Sci. 2015 May 1;1:16. doi: 10.1186/s40780-015-0016-5. eCollection 2015.	Tanaka S	J Pharm Health Care Sci	2015
28	34694230	Adverse Drug Event Prediction Using Noisy Literature-Derived Knowledge Graphs: Algorithm Development and Validation	JMIR Med Inform. 2021 Oct 25;9(10):e32730. doi: 10.2196/32730.	Dasgupta S	JMIR Med Inform	2021
29	36438789	Data mining and safety analysis of BTK inhibitors: A pharmacovigilance investigation based on the FAERS database	Front Pharmacol. 2022 Nov 11;13:995522. doi: 10.3389/fphar.2022.995522. eCollection 2022.	Wan Q	Front Pharmacol	2022
30	28595649	Data-driven prediction of adverse drug reactions induced by drug-drug interactions	BMC Pharmacol Toxicol. 2017 Jun 8;18(1):44. doi: 10.1186/s40360 017-0153-6.	Liu R	BMC Pharmacol Toxicol	2017
31	31004158	Prevalence and incidence of narcolepsy in a US health care claims database, 2008-2010	Sleep. 2019 Jul 8;42(7):zsz091. doi: 10.1093/sleep/zsz091.	Scheer D	Sleep	2019
32	31507029	Data variability across Canadian administrative health databases: Differences in content, coding, and completeness	Pharmacoepidemiol Drug Saf. 2020 Jan;29 Suppl 1:68-77. doi: 10.1002/pds.4889. Epub 2019 Sep 10.	Doyle CM	Pharmacoepidemiol Drug Saf	2020
33	32564242	Risk Factor Considerations in Statistical Signal Detection: Using Subgroup Disproportionality to Uncover Risk Groups for Adverse Drug Reactions in VigiBase	Drug Saf. 2020 Oct;43(10):999-1009. doi: 10.1007/s40264-020-00957-w.	Sandberg L	Drug Saf	2020
34	23070598	Comparison of two drug safety signals in a pharmacovigilance data mining framework	Stat Methods Med Res. 2016 Apr;25(2):615-29. doi: 10.1177/0962280212462295. Epub 2012 Oct 14.	Tubert-Bitter P	Stat Methods Med Res	2016
35	29446035	Sorting Through the Safety Data Haystack: Using Machine Learning to Identify Individual Case Safety Reports in Social-Digital Media	Drug Saf. 2018 Jun;41(6):579-590. doi: 10.1007/s40264-018-0641-7.	Comfort S	Drug Saf	2018
36	24787710	Paediatric pharmacovigilance: use of pharmacovigilance data mining algorithms for signal detection in a safety dataset of a paediatric clinical study conducted in seven African countries	PLoS One. 2014 May 1;9(5):e96388. doi: 10.1371/journal.pone.0096388. eCollection 2014.	Kajungu DK	PLoS One	2014
37	28646789	Data-mining for detecting signals of adverse drug reactions of fluoxetine using the Korea Adverse Event Reporting System (KAERS) database	Psychiatry Res. 2017 Oct;256:237-242. doi: 10.1016/j.psychres.2017.06.038. Epub 2017 Jun 13.	Kim S	Psychiatry Res	2017
38	21701609	Information technology in pharmacovigilance: Benefits, challenges, and future directions from industry perspectives	Drug Healthc Patient Saf. 2009;1:35-45. doi: 10.2147/dhps.s7180. Epub 2009 Oct 15.	Lu Z	Drug Healthc Patient Saf	2009
39	27868278	Differences in VigiBase® reporting of aminoglycoside and capreomycin-suspected ototoxicity during tuberculosis treatment	Pharmacoepidemiol Drug Saf. 2017 Jan;26(1):1-8. doi: 10.1002/pds.4125. Epub 2016 Nov 20.	Sagwa EL	Pharmacoepidemiol Drug Saf	2017
40	23921799	Triptans and serious adverse vascular events: data mining of the FDA Adverse Event Reporting System database	Cephalalgia. 2014 Jan;34(1):5-13. doi: 10.1177/0333102413499649. Epub 2013 Aug 6.	Roberto G	Cephalalgia	2014
41	31673326	Evaluation of quantitative signal detection in EudraVigilance for orphan drugs: possible risk of false negatives	Ther Adv Drug Saf. 2019 Oct 21;10:2042098619882819. doi: 10.1177/2042098619882819. eCollection 2019.	Sardella M	Ther Adv Drug Saf	2019
42	36050484	Detecting early safety signals of infliximab using machine learning algorithms in the Korea adverse event reporting system	Sci Rep. 2022 Sep 1;12(1):14869. doi: 10.1038/s41598-022-18522-z.	Lee JE	Sci Rep	2022
43	30324671	Significance of data mining in routine signal detection: Analysis based on the safety signals identified by the FDA	Pharmacoepidemiol Drug Saf. 2018 Dec;27(12):1402-1408. doi: 10.1002/pds.4672. Epub 2018 Oct 15.	Fukazawa C	Pharmacoepidemiol Drug Saf	2018
44	31156424	Computational Advances in Drug Safety: Systematic and Mapping Review of Knowledge Engineering Based Approaches	Front Pharmacol. 2019 May 17;10:415. doi: 10.3389/fphar.2019.00415. eCollection 2019.	Natsiavas P	Front Pharmacol	2019
45	28335691	Cases of drug-induced Torsade de Pointes: a review of Belgian cases in the EudraVigilance database	Acta Clin Belg. 2017 Dec;72(6):385-390. doi: 10.1080/17843286.2017.1300217. Epub 2017 Mar 24.	Vandael E	Acta Clin Belg	2017
46	16134080	Signal detection in pharmacovigilance: empirical evaluation of data mining tools	Pharmacoepidemiol Drug Saf. 2005 Sep;14(9):597-9. doi: 10.1002/pds.1128.	Chan KA	Pharmacoepidemiol Drug Saf	2005
47	35745593	Pharmacovigilance Signals of the Opioid Epidemic over 10 Years: Data Mining Methods in the Analysis of Pharmacovigilance Datasets Collecting Adverse Drug Reactions (ADRs) Reported to EudraVigilance (EV) and the FDA Adverse Event Reporting System (FAERS)	Pharmaceuticals (Basel). 2022 May 27;15(6):675. doi: 10.3390/ph15060675.	Chiappini S	Pharmaceuticals (Basel)	2022

No.	PMID	Title	Citation	First Author	Journal/Book	Publication Year
48	36050787	Challenges and opportunities for mining adverse drug reactions: perspectives from pharma, regulatory agencies, healthcare providers and consumers	Database (Oxford). 2022 Sep 2;2022:baac071. doi: 10.1093/database/baac071.	Gonzalez-Hernandez G	Database (Oxford)	2022
49	33518634	[Role and Applicability of Spontaneous Reporting Databases in Medical Big Data]	Yakugaku Zasshi. 2021;141(2):165-168. doi: 10.1248/yakushi.20-00196-1.	Sakai T	Yakugaku Zasshi	2021
50	32169289	Uses of pharmacovigilance databases: An overview	Therapie. 2020 Nov-Dec;75(6):591-598. doi: 10.1016/j.therap.2020.02.022. Epub 2020 Feb 26.	Bihan K	Therapie	2020
51	35904529	The Use of Artificial Intelligence in Pharmacovigilance: A Systematic Review of the Literature	Pharmaceut Med. 2022 Oct;36(5):295-306. doi: 10.1007/s40290-022-00441-z. Epub 2022 Jul 29.	Salas M	Pharmaceut Med	2022
52	31623850	Pharmacovigilance - The next chapter	Therapie. 2019 Dec;74(6):557-567. doi: 10.1016/j.therap.2019.09.004. Epub 2019 Oct 1.	Moore N	Therapie	2019
53	27224991	[Pharmacovigilance in Germany : It is about time]	Internist (Berl). 2016 Jun;57(6):616-23. doi: 10.1007/s00108-016-0068-0.	Douros A	Internist (Berl)	2016
54	33598764	Construction and analysis of a database for medication errors in a pharmacovigilance centre-the Moroccan experience	Eur J Clin Pharmacol. 2021 Aug;77(8):1235-1246. doi: 10.1007/s00228-021-03109-w. Epub 2021 Feb 17.	Alj L	Eur J Clin Pharmacol	2021
55	35788724	Evolution of adverse drug reactions reporting systems: paper based to software based	Eur J Clin Pharmacol. 2022 Sep;78(9):1385-1390. doi: 10.1007/s00228-022-03358-3. Epub 2022 Jul 5.	Madhushika MT	Eur J Clin Pharmacol	2022
56	23640186	The past, present and perhaps future of pharmacovigilance: homage to Folke Sjoqvist	Eur J Clin Pharmacol. 2013 May;69 Suppl 1:33-41. doi: 10.1007/s00228-013-1486-8. Epub 2013 May 3.	Moore N	Eur J Clin Pharmacol	2013
57	35579806	Artificial Intelligence in Pharmacovigilance: An Introduction to Terms, Concepts, Applications, and Limitations	Drug Saf. 2022 May;45(5):407-418. doi: 10.1007/s40264-022-01156-5. Epub 2022 May 17.	Aronson JK	Drug Saf	2022
58	36251280	Development of a multivariate prediction model to identify individual case safety reports which require clinical review	Pharmacoepidemiol Drug Saf. 2022 Dec;31(12):1300-1307. doi: 10.1002/pds.5553. Epub 2022 Oct 21.	Gosselt HR	Pharmacoepidemiol Drug Saf	2022
59	26575203	Big Data and Adverse Drug Reaction Detection	Clin Pharmacol Ther. 2016 Mar;99(3):268-70. doi: 10.1002/cpt.302. Epub 2015 Dec 23.	Harpaz R	Clin Pharmacol Ther	2016
60	32176553	The potential role of big data in the detection of adverse drug reactions	Expert Rev Clin Pharmacol. 2020 Mar;13(3):201-204. doi: 10.1080/17512433.2020.1740086. Epub 2020 Mar 16.	Sultana J	Expert Rev Clin Pharmacol	2020
61	24698186	[Terappel: description of a computerised database and examples of studies]	Therapie. 2014 Jan-Feb;69(1):31-8. doi: 10.2515/therapie/2014013. Epub 2014 Apr 4.	Vial T	Therapie	2014
62	28840504	From Big Data to Smart Data for Pharmacovigilance: The Role of Healthcare Databases and Other Emerging Sources	Drug Saf. 2018 Feb;41(2):143-149. doi: 10.1007/s40264-017-0592-4.	Trifirò G	Drug Saf	2018
63	22315152	Electronic healthcare databases for active drug safety surveillance: is there enough leverage?	Pharmacoepidemiol Drug Saf. 2012 Jun;21(6):611-21. doi: 10.1002/pds.3197. Epub 2012 Feb 8.	Coloma PM	Pharmacoepidemiol Drug Saf	2012
64	19745234	The EU-ADR project: preliminary results and perspective	Stud Health Technol Inform. 2009;148:43-9.	Trifiro G	Stud Health Technol Inform	2009
65	26092163	[Benefits of large healthcare databases for drug risk research]	Bundesgesundheitsblatt Gesundheitsforschung Gesundheitsschutz. 2015 Aug;58(8):829-837. doi: 10.1007/s00103-015-2185-7.	Garbe E	Bundesgesundheitsblatt Gesund	2015
66	23670805	Pilot evaluation of an automated method to decrease false-positive signals induced by co-prescriptions in spontaneous reporting databases	Pharmacoepidemiol Drug Saf. 2014 Feb;23(2):186-94. doi: 10.1002/pds.3454. Epub 2013 May 14.	Avillach P	Pharmacoepidemiol Drug Saf	2014
67	23208789	The EU-ADR Web Platform: delivering advanced pharmacovigilance tools	Pharmacoepidemiol Drug Saf. 2013 May;22(5):459-67. doi: 10.1002/pds.3375. Epub 2012 Dec 4.	Oliveira JL	Pharmacoepidemiol Drug Saf	2013
68	31912439	Towards Automating Adverse Event Review: A Prediction Model for Case Report Utility	Drug Saf. 2020 Apr;43(4):329-338. doi: 10.1007/s40264-019-00897-0.	Muñoz MA	Drug Saf	2020
69	22873501	Implemented data mining and signal management systems on spontaneous reporting systems' databases and their availability to the scientific community - a systematic review	Curr Drug Saf. 2012 Apr;7(2):170-5. doi: 10.2174/157488612802715645.	de Almeida Vieira Lima LM	Curr Drug Saf	2012
70	22549283	Novel data-mining methodologies for adverse drug event discovery and analysis	Clin Pharmacol Ther. 2012 Jun;91(6):1010-21. doi: 10.1038/clpt.2012.50.	Harpaz R	Clin Pharmacol Ther	2012
71	18523760	Pharmacovigilance: methods, recent developments and future perspectives	Eur J Clin Pharmacol. 2008 Aug;64(8):743-52. doi: 10.1007/s00228-008-0475-9. Epub 2008 Jun 4.	Härmark L	Eur J Clin Pharmacol	2008
72	23072620	Global patterns of adverse drug reactions over a decade: analyses of spontaneous reports to VigiBase™	Drug Saf. 2012 Dec 1;35(12):1171-82. doi: 10.1007/BF03262002.	Aagaard L	Drug Saf	2012

No.	PMID	Title	Citation	First Author	Journal/Book	Publication Year
73	15366975	The general practice research database: role in pharmacovigilance	Drug Saf. 2004;27(12):871-81. doi: 10.2165/00002018-200427120-00004.	Wood L	Drug Saf	2004
74	23761351	Detection of adverse drug reactions using hospital databases-a nationwide study in Portugal	Pharmacoepidemiol Drug Saf. 2013 Aug;22(8):907-13. doi: 10.1002/pds.3468. Epub 2013 Jun 13.	Miguel A	Pharmacoepidemiol Drug Saf	2013
75	15366974	Data quality management in pharmacovigilance	Drug Saf. 2004;27(12):857-70. doi: 10.2165/00002018-200427120-00003.	Lindquist M	Drug Saf	2004
76	15460169	Pharmacovigilance in the 21st century: new systematic tools for an old problem	Pharmacotherapy. 2004 Sep;24(9):1099-104. doi: 10.1592/phco.24.13.1099.38090.	Szarfman A	Pharmacotherapy	2004
77	16111454	The role of data mining in pharmacovigilance	Expert Opin Drug Saf. 2005 Sep;4(5):929-48. doi: 10.1517/14740338.4.5.929.	Hauben M	Expert Opin Drug Saf	2005
78	15221098	[Databases as a source for monitoring systems of drug safety]	Bundesgesundheitsblatt Gesundheitsforschung Gesundheitsschutz. 2004 Jun;47(6):513-7. doi: 10.1007/s00103-004-0834-3.	Pigeot I	Bundesgesundheitsblatt Gesund	2004
79	20721787	Mining pharmacovigilance data using Bayesian logistic regression with James-Stein type shrinkage estimation	J Biopharm Stat. 2010 Sep;20(5):998-1012. doi: 10.1080/10543401003619056.	An L	J Biopharm Stat	2010
80	16180934	Data mining in pharmacovigilance: the need for a balanced perspective	Drug Saf. 2005;28(10):835-42. doi: 10.2165/00002018-200528100-00001.	Hauben M	Drug Saf	2005
81	17604418	Signal detection in the pharmaceutical industry: integrating clinical and computational approaches	Drug Saf. 2007;30(7):627-30. doi: 10.2165/00002018-200730070-00012.	Hauben M	Drug Saf	2007
82	15360888	A knowledge based approach for automated signal generation in pharmacovigilance	Stud Health Technol Inform. 2004;107(Pt 1):626-30.	Henegar C	Stud Health Technol Inform	2004
83	12650632	Application of quantitative signal detection in the Dutch spontaneous reporting system for adverse drug reactions	Drug Saf. 2003;26(5):293-301. doi: 10.2165/00002018-200326050-00001.	van Puijenbroek E	Drug Saf	2003
84	Yuan Luo	Natural Language Processing for EHR-Based Pharmacovigilance: A Structured Review	Evanston USA	Yuan Luo	This article is a comprehensive	2017
85	ECRI INST	Wrong-Record, Wrong-Data Errors with Health IT Systems.	Pennsylvania	ECRI INSTITUTE PSO	We define data integrity failure	2015
86	European M	Guideline on good pharmacovigilance practices (GVP)<br>Module IX Addendum I – Methodological aspects of signal detection from spontaneous reports of suspected adverse reactions	Netherlands	European Medicines Agency	The decision based on the asses	2015
87	FDA	Questions and Answers on FDA's Adverse Event Reporting System (FAERS)	USA	FDA	What is FAERS? The FDA Ad	2018
88	Liu M et al	Comparative analysis of pharmacovigilance methods in the detection of adverse drug reactions using electronic medical records	New Jersey	Liu M et al	Medication safety requires that	2013
89	Norén GN	A hit-miss model for duplicate detection in the WHO drug safety database	Sweden.	Norén GN et al	We present two new generalisa	2005
90	Niklas Nor	A hit-miss model for duplicate detection in the WHO drug safety database		Niklas Norén et al	We demonstrate the effectivene	2005
91	eudrac	https://www.eudrac.com/news/good-pharmacovigilance-practice-guideline-updates	Germany			
92	PV Drug S	https://www.linkedin.com/posts/pv-drug-safety-academy-841528223_pharmacovigilance-activity-7071440228577660928-KRp9/	England			
93	Drug Regu	https://www.dra.gov.pk/wp-content/uploads/2022/04/Good-Pharmacovigilance-Guidelines-for-Registration-Holders.pdf	Pakistan			
94	cod-researc	https://cod-research.com/ease-icsr-handling-through-early-duplicate-identification-and-its-management/	India			
95	ECA Found	https://www.gmp-compliance.org/guidelines/gmp-guideline/eudralex-volume-9-gvp-module-vi-addendum-i-duplicate-management-of-suspected-adverse-reaction-reports	Germany			
96	Applied Cl	https://www.appliedclinicaltrialsonline.com/view/pharmacovigilance-literature-monitoring-best-practices	Fort Washington USA			
97	ClinosolInd	https://www.slideshare.net/ClinosolIndia/duplicate-case	India			
98	clarivate	https://clarivate.com/blog/seeing-double-eliminating-duplicate-references-in-drug-safety-literature-screening/	Jersey			
