# Supplementary material for: Navigating duplication in pharmacovigilance databases: a scoping review
Source: BMJ Open. 2024 Apr 29;14(4):e081990. doi: 10.1136/bmjopen-2023-081990 (PMC11086478; doi:10.1136/bmjopen-2023-081990)
Supplement: Supplementary data [file bmjopen-2023-081990supp001.pdf]

Supplementary Table 1: List of 58 papers included in the scoping review.

No.	Author(s)	Year of publication	Journal	Country	Title	Specific Objectives	Study design	Categories						Key findings
								Extent	Prevention	Causes	Detection	Management	Implications	
1	Norén GN	2017	Drug Safety	Sweden	The Power of the Case Narrative - Can it be Brought to Bear on Duplicate Detection?	To discuss the power of the case narrative in duplicate detection.	Commentary				✓			Kreimeyer et al. primarily focus on detecting duplicates in a specific drug or vaccine at a time, with examples in their study typically involving around 1000 reports each. Their research demonstrates the potential value of automated duplicate detection in this context. Manual identification becomes challenging, even with moderately sized case series, with over 400 possible pairs among 30 reports and 5000 pairs among 100 reports. Additionally, not all duplicates are easily identifiable by human assessors, as evidenced by instances of false positives where record pairs initially assumed to be algorithmic errors were later found to be genuine duplicates that had been overlooked.
2	Ramya	2019		Georgia	Duplicate search in pharmacovigilance	To define duplicates, identify their causes, address their handling, confirm their existence, and manage their occurrence.	Web page			✓		✓	✓	The detection and handling of duplicates by National Competent Authorities, Marketing Authorization Holders, and sponsors of clinical trials is an important element of good case management.
3	Likeng Liang *et al* .	2022	Drug Safety	China	Artificial Intelligence-Based Pharmacovigilance in the Setting of Limited Resources.	To review the possible solutions and future perspectives on AI-based pharmacovigilance in resource-limited settings.	Review article					✓		Artificial intelligence (AI) algorithms can process and analyse data relevant to pharmacovigilance. However, the key challenge lies in the initial training of AI algorithms with substantial amounts of high-quality data. Resource-limited settings face notable technical obstacles for AI-driven pharmacovigilance, including the scarcity of robust databases, inadequate human resources, underdeveloped AI technology, and insufficient governmental support. Enhancing the detection capabilities of AI-based pharmacovigilance through improved training and education, along with advocating for the advantages of AI-based approaches to governments, offers a pathway to addressing these challenges in settings constrained by resources. Future advancements in AI-based pharmacovigilance within resource-limited contexts are anticipated to be facilitated by collaborative research networks, advancements in pharmacogenomic research and practices, and the integration of sophisticated machine-learning algorithms. It is imperative to tailor these efforts to the specific contexts of such settings.
4	Manfred Hauben *et al* .	2023	Clinical Therapeutics.	USA	Artificial Intelligence and Data Mining for the Pharmacovigilance of Drug-Drug Interactions.	Despite increasing mechanistic understanding, undetected and underrecognized drug–drug interactions (DDIs) persist. The present article is intended to be part didactic primer and part narrative review of real-world and experimental deployment of AI and data mining activities for pharmacovigilance of DDIs.	Review article					✓		Artificial Intelligence and Data Mining for the Pharmacovigilance of Drug-Drug Interactions. The wide array of intricate and elegant methods has expanded the pharmacovigilance tool kit.

No.	Author(s)	Year of publication	Journal	Country	Title	Specific Objectives	Study design	Categories						Key findings
								Extent	Prevention	Causes	Detection	Management	Implications	
5	Wadhwa *et al*.	2021	Oxford Academic	India	Automation in signal management in pharmacovigilance—an insight.	To review the recent developments of automated systems associated with the detection and prioritization of potential signals with the help of effective healthcare databases to enhance public health safety.	Review article				✓			Automated signal detection is highly based upon the data mining of the spontaneous reporting system such as reports from healthcare professionals, observational studies, medical literature or from social media. The main components of automated signal detection are data extraction, data acquisition, data selection, and data analysis and data evaluation. This system must be developed in the correct format and context, which eventually emphasizes the quality of data collected and leads to optimal decision-making based on scientific evaluation.
6	Adam Lavertu *et al.*	2021	Clinical Pharmacology & Therapeutics	USA	A New Era in Pharmacovigilance: Toward Real-World Data and Digital Monitoring	Several different approaches to modern pharmacovigilance, including spontaneous reporting databases, electronic health record monitoring and research frameworks, social media surveillance, and the use of digital devices. Some of these platforms are well-established while others are still emerging or experimental. We highlight both the potential opportunity, as well as the existing challenges within these pharmacovigilance systems that have already begun to impact the drug development process, as well as the landscape of postmarket drug safety monitoring.	Mini review				✓			The development of massive data sources for future pharmacovigilance efforts creates an opportunity for advances in deep learning and anomaly detection. A continuously learning artificial intelligence system could not only learn to integrate these heterogeneous data sources for real-time case detection but could also help identify potential cases and interface with members of the pharmacotherapy community to gather more information when needed.
7	Rodrigo Postigo *et al*.	2018	Drug Safety	UK	EudraVigilance Medicines Safety Database: Publicly Accessible Data for Research and Public Health Protection	The main objective of this article is to describe the new access to the database together with the opportunities that this new access can bring for research. It is intended to promote an appropriate use of the data to support the safe and effective use of medicines.	Special article		✓					The European Union legislation gives the EudraVigilance (EV) database the core role of managing reports of suspected adverse reactions and determine changes in the safety profile of medicinal products used in medical practice. The EV has demonstrated its value especially in the area of signal detection, providing a tool to analyse the safety data submitted.
8	Jenna Wong *et al*	2022	Drug Safety	Switzerland	Applying Machine Learning in Distributed Data Networks for Pharmacoepidemiologic and Pharmacovigilance Studies: Opportunities, Challenges, and Considerations.	In this paper, we discuss opportunities, challenges, and considerations for applying Machine Learning (ML) in Distributed Data Networks (DDNs) for pharmacoepidemiologic and pharmacovigilance studies. First, we discuss the major types of activities performed by DDNs and how ML may be used. Next, we discuss practical data-related factors infuencing how DDNs work in practice. We then combine these discussions and jointly consider how opportunities for ML are afected by practical data-related factors for DDNs, leading to several challenges. We present diferent approaches for addressing these challenges and highlight eforts that real-world DDNs have taken or are currently taking to help mitigate them. Despite these challenges, the time is ripe for the emerging interest to use ML in DDNs, and the utility of these data-adaptive modeling techniques in pharmacoepidemiologic and pharmacovigilance studies will likely continue to increase in the coming years.	Review article		✓		✓			Using ML in DDNs for pharmacoepidemiology and pharmacovigilance holds great promise. This is an emerging area of interest for DDNs because of the recent methodologic advancements in the feld, and the valuable groundwork that many DDNs have already laid through investments in harmonizing datasets, developing standardised processes, tools, and analytics, and building collaborative relationships between data partners and with various stakeholders. The future holds much promise for the use of ML in DDNs, and we expect that the utility of these data-adaptive methods for enhancing pharmacoepidemiologic and pharmacovigilance studies will likely continue to increase in the years to come.
9	Philip Michael Tregunno *et al*	2014	Drug Safety	UK	Performance of Probabilistic Method to Detect Duplicate Individual Case Safety Reports	The aim of this study was to evaluate probabilistic record matching for duplicate detection, and to characterise the main sources of duplicate reports within each data set.	Original Research Article	✓	✓	✓	✓	✓	✓	Probabilistic record matching, as implemented in vigiMatch, achieved good predictive value for confirmed or likely duplicates in each data source. Most of the false positives corresponded to otherwise related reports; less than 10 % were altogether unrelated. A substantial proportion of the correctly identified duplicates had not previously been detected by national centre activity. On one hand, vigiMatch highlighted duplicates that had been missed by rule-based methods, and on the other hand its lower total number of suspected duplicates to review improved the accuracy of manual review.

No.	Author(s)	Year of publication	Journal	Country	Title	Specific Objectives	Study design	Categories						Key findings
								Extent	Prevention	Causes	Detection	Management	Implications	
10	Benjamin Kompa *et al*	2022	Drug Safety	USA	Artifcial Intelligence Based on Machine Learning in Pharmacovigilance: A Scoping Review	The present study conducted a scoping review of the use of artificial intelligence based on machine learning to understand how it is used for pharmacovigilance tasks, characterize differences with other fields, and identify opportunities to improve pharmacovigilance through the use of machine learning.	Scoping Review				✔	✔		While certain modern practices have begun to appear, many of the primary reasons for the recent success of artificial intelligence (AI) have yet to be translated into pharmacovigilance. Without certain structural changes, PV is unlikely to experience similar kinds of advancements from current approaches to AI.
11	Marin Banovac *et al*	2017	Drug Safety	UK	Patient Reporting in the EU: Analysis of EudraVigilance Data	This descriptive study aims to provide insight into patient reporting for the totality of the EU by querying the EudraVigilance (EV) database for the period of 3 years before the new pharmacovigilance legislation became operational and the 3 years after as well as comparing patient reports with those from healthcare professionals (HCPs) where feasible.	Descriptive study			✔				Patient reporting complemented reporting by HCPs. Reports were submitted for which the patient and HCP were both listed as primary sources. Patients were motivated to report adverse reactions, especially those that affected their quality of life. Sharing these results with National Competent Authorities and patient associations can inform training and awareness on patient reporting.
12	Gianmario Candore *et al*	2022	Drug Safety	Netherlands	The Impact of Mandatory Reporting of Non-Serious Safety Reports to EudraVigilance on the Detection of Adverse Reactions	This paper reports on a series of analyses performed to assess the number and nature of non-serious reports and their effect on signal detection in EudraVigilance.	Descriptive study			✔				The new requirement to report non-serious suspect reactions increased report submissions, improving sensitivity without affecting statistical efficiency. As non-serious reports in EudraVigilance rise, ongoing monitoring is crucial for signal detection impact. Further study is needed to assess the qualitative impact of non-serious reports on signal nature and evaluation.
13	John van Stekelenborg *et al*	2022	Drug Safety	USA	Individual Case Safety Report Replication: An Analysis of Case Reporting Transmission Networks	The present study aimed to investigate the magnitude of replication, the variability among recipients, and the subsequent divergence across recipients of ICSRs.	Original Research Articl						✔	Replication of ICSRs and the variation of available safety information in recipient databases were quantified and shown to be substantial. Multiple processors and medical reviewers will likely handle the same original ICSR as a result of replication. Aside from the obvious duplicate work, this phenomenon could conceivably lead to differing clinical assessments and decisions. If replication could be reduced or even eliminated, this would enable more focus on activities with a benefit for patient safety.
14	Rachel L Richesson *et al*	2008	Drug Safety	USA	An Automated Standardized System for Managing Adverse Events in Clinical Research Networks	We describe an Adverse Event Data Management System (AEDAMS) that is used across multiple study designs in the various clinical research networks and multi-site studies for which we provide data and technological support. Investigators enter AE data using a standardized and structured web-based data collection form	Author manuscript		✔			✔		The automated AEDAMS promptly reports adverse events (AEs) to various parties, enhancing participant safety oversight, especially in multi-site protocols. Standardized AE reporting in clinical research networks enables centralized monitoring, potentially improving agent safety comprehension across studies. Automated systems offer speed, efficiency, and scalability, impacting patient safety and streamlining research processes. Future studies should rigorously evaluate AE management systems, measuring efficiency, reliability, accuracy, and potential reductions in patient morbidity and mortality resulting from improved communication and decision-making. Such evaluations would facilitate faster application of investigational treatments and enhance overall research quality and safety.

No.	Author(s)	Year of publication	Journal	Country	Title	Specific Objectives	Study design	Categories						Key findings
								**Extent**	**Prevention**	**Causes**	**Detection**	**Management**	**Implications**	
15	Michele Fusaroli *et al*	2021	Frontiers in Pharmacology	Italy	Development of a Network-Based Signal Detection Tool: The COVID-19 Adversome in the FDA Adverse Event Reporting System	To propose a network-based approach on co-reported events to help assess disproportionalities and to effectively identify, in a timely manner, the disease-, comorbidity- and drug-related syndromes, especially in a rapidly changing low-resources environment such as that of COVID-19.	Brief research report article.				✓			The Adversome detects plausible new signals and iatrogenic syndromes. The network approach complements traditional pharmacovigilance analyses and may represent a more effective signal detection technique to guide clinical recommendations by regulators and specific follow-up confirmatory studies.
16	Rave Harpaz *et al*	2010	BMC Bioinformatics.	USA	Mining multi-item drug adverse effect associations in spontaneous reporting systems.	This paper examines the application of a well-established data mining method known as association rule mining and demonstrates its value.	Research article.		✓		✓	✓	✓	Our findings demonstrate that multi-item ADEs are present and can be extracted from the FDA's adverse effect reporting system using our methodology, suggesting that our method is a valid approach for the initial identification of multi-item ADEs. The study also revealed several limitations and challenges that can be attributed to both the method and quality of data.
17	F Martin-Sanchez *et al*	2014		Australia	Big data in medicine is driving big changes.	To summarise current research that takes advantage of "Big Data" in health and biomedical informatics applications.	Review article		✓	✓	✓	✓	✓	The survey highlights ongoing development of powerful new methods for turning large-scale, and often complex, data into information that provides new insights into human health, in a range of different areas. Consideration of this body of work identifies several important paradigm shifts that are facilitated by big data resources and methods in clinical and translational research, from hypothesis-driven research to data-driven research, and in medicine, from evidence-based practice to practice-based evidence. The increasing scale and availability of large quantities of health data require strategies for data management, data linkage, and data integration beyond the limits of many existing information systems. As our ability to make sense of that data improves, the value of the data will continue to increase. All areas of biomedicine stand to benefit from Big Data and the associated technologies.
18	Marie Lindquist	2004	Drug Safety	Sweden	Data Quality Management in Pharmacovigilance.	To describe the first three steps of the data processing cycle (collection, entry, storage) and the different quality dimensions associated with these steps, together with examples relevant to pharmacovigilance data	Leading article.				✓			Ensuring data quality throughout processing is crucial in pharmacovigilance. The implementation of robust data quality management practices is essential to address issues perpetuated through the processing cycle. While regulatory requirements dictate reporting standards, database systems largely manage data quality, often without comprehensive appraisal. Inter-system data exchange poses additional quality risks. However, initiatives like 'good pharmacovigilance practice' and emerging conferences indicate progress toward enhancing data quality across the pharmaceutical landscape. Transparent, well-documented quality systems are imperative. Despite acknowledgment of shortcomings in current systems, ongoing efforts aim to reduce errors and misinterpretations, facilitating safer pharmacovigilance processes globally.

No.	Author(s)	Year of publication	Journal	Country	Title	Specific Objectives	Study design	Categories						Key findings
								Extent	Prevention	Causes	Detection	Management	Implications	
19	Monica A. Muñoz *et al*	2020	Drug Safety	USA	Towards Automating Adverse Event Review: A Prediction Model for Case Report Utility.	This study aimed to develop and validate a model predictive of an individual case safety report (ICSR) pharmacovigilance utility (PVU). Techniques to systematically identify and distinguish higher utility ICSRs from lower utility ones will improve timeliness in managing safety signals.	Research article.				✓			The model demonstrated the feasibility of developing a tool predictive of ICSR utility. The model's modest discriminative ability highlighted opportunities for further enhancement and suggested algorithms tailored to safety issues may be benefcial.
20	José Luis Oliveira *et al*	2013	Pharmacoepidemiology & Drug Safety	Portugal	The EU-ADR Web Platform: delivering advanced pharmacovigilance tools.	To describe the EU-ADR Web Platform built to facilitate accessing, monitoring and exploring pharmacovigilance data, enabling an in-depth analysis of adverse drug reactions risks.	Website		✓		✓	✓		The EU-ADR European project embraces innovative pharmacovigilance research methodologies through the creation of a web platform providing advanced drug data exploration and assessment features. Whereas, in the past, post-marketing drug assessment required intense validation tasks, the in silico pharmacology community is now endowed with the tools required to quickly analyse specific ADRs, further improving drug safety monitoring. The EU-ADR Web Platform enables streamlined access to drug dataset analysis features, including the evaluation of results from EU-ADR workflows and the sharing of data amongst research partners, all within a highly responsive and unique web-based workspace.
21	Helen R. Gosselt *et al*	2022	Pharmacoepidemiology & Drug Safety	Netherlands	Development of a multivariate prediction model to identify individual case safety reports which require clinical review.	Objectives: To develop a prediction model to identify individual case safety reports that require clinical review, including potential signal triggering reports. Secondly, to identify the most important features of these reports.	Original Research Article.						✓	The number of individual case safety reports in pharmacovigilance databases are increasing.<br>New approaches are required to prioritise individual case reports based on their potential signal triggering value since case-by-case review is very time consuming.<br>The authors developed a prediction model using structured data of individual case safety reports to identify potential signal triggering reports in the Netherlands.<br>The authors identified nine most important predictors that were used to identify potential signal triggering reports.<br>The prediction model could be further optimised using additional predictors such as text fields or other unstructured data from case reports.
22	Tomas Lasys *et al*	2023	BJCP	Netherland	Unintended impact of pharmacovigilance regulatory interventions: A systematic review.	This systematic literature review investigated how often the unintended impact of regulatory interventions was considered in publications of studies investigating pharmacovigilance regulatory interventions in Europe.	Review article.			✓				The unintended impact of pharmacovigilance regulatory interventions was reported in only a quarter of identified publications. There was no apparent increase in attention to unintended impact assessments after the update of the Good Pharmacovigilance Practice guidelines.
23	Ruta Mockute *et al*	2017	Drug Safety	USA	Artificial Intelligence Within Pharmacovigilance: A Means to Identify Cognitive Services and the Framework for their Validation.	The aim of this study was to identify areas across the PV value chain that can be augmented by cognitive service solutions using the methodologies of contextual analysis and cognitive load theory.	Review article.		✓					As individual case safety report volumes increase, artificial intelligence can be a means to help mitigate complex decision making for pharmacovigilance professionals. At various decision points in the PV process, cognitive services were identified and developed to assist pharmacovigilance users. These services were validated using a framework leveraging the Acceptance Quality Limit method, to ensure appropriate performance and quality control.

No.	Author(s)	Year of publication	Journal	Country	Title	Specific Objectives	Study design	Categories						Key findings
								Extent	Prevention	Causes	Detection	Management	Implications	
24	Jianxing He *et a*	2019	Nat Med.	China	The practical implementation of artificial intelligence technologies in medicine.	The development of artificial intelligence (AI)-based technologies in medicine is advancing rapidly, but real-world clinical implementation has not yet become a reality. Here we review some of the key practical issues surrounding the implementation of AI into existing clinical workflows, including data sharing and privacy, transparency of algorithms, data standardization, and interoperability across multiple platforms, and concern for patient safety. The study summarizes the current regulatory environment in the United States and highlights comparisons with other regions in the world, notably Europe and China.	Review article.					✓		AI has recently experienced an era of explosive growth across many industries, and the healthcare industry is no exception. Studies across multiple medical specialties have employed AI to mimic the diagnostic abilities of physicians. The hope is that AI may augment the ability of humans to provide healthcare. However, although these technologies are rapidly advancing, their implementation into patient-care settings has not yet become widespread.
25	Juergen Schmide	2018	Clinical Pharmacology & Therapeutics.	USA	Innovation in Pharmacovigilance: Use of Artificial Intelligence in Adverse Event Case Processing.	The current pilot was undertaken to prove the viability of commercially offered machine-learning solutions in the application for case processing. The pilot paradigm was used to simultaneously test the proposed solutions of three commercial vendors for the ability to extract case critical information from source documents to identify valid AE cases after training the machine-learning algorithms with source documents and database content rather than annotated source documents.	Research article.				✓			The pilot was successful in confirming the feasibility of using AI-based tools to support PV operations and in demonstrating the viability of an efficient training method that does not require time-consuming and costly annotations. Finally, the evaluation and scoring method used in the pilot was able to differentiate vendor capabilities and identify vendor 1 as the best candidate to move into the discovery phase.
26	David John Le wis *et al*	2019	PMC	Germany	Utilizing Advanced Technologies to Augment Pharmacovigilance Systems: Challenges and Opportunities.	This paper provides a series of considerations for the applicability of new IT, with explanations of the technology and its applicability within specific domains of the PV system. The overall goal of automation is to provide high-quality safety data in the correct format, in context, more quickly, and with less manual effort.	Rreview article		✓		✓	✓		The rapid advancement of technologies often outpaces regulations governing pharmacovigilance (PV) systems. Yet, intelligent automation offers avenues for system improvement. Owners must ensure the suitability, design, and reliability of advanced technologies, ensuring PV systems remain effective. Trust in intelligent automation is paramount, aiming to deliver safety data accurately and informatively, enhancing evidence quality for optimal scientific evaluation. Alignment with perspectives in biomedical sciences underscores the need for PV system owners to adapt progressively to artificial intelligence, starting with process automation. Algorithmic processing has demonstrated superior performance in binary, repetitive tasks, advocating for its integration into PV systems.
27	European Medicines Agency	2017	Not applicable	Europe	Guideline on good pharmacovigilance practices (GVP): Module VI – Collection, management and submission of reports of suspected adverse reactions to medicinal products (Rev 2)	Section VI.B. of this Module highlights the general principles, based on the pharmacovigilance guidelines E2A, E2B and E2D of the International Council for Harmonisation of Technical Requirements for Pharmaceuticals for Human Use (ICH) (see GVP Annex IV), in relation to the collection, recording and submission of individual reports of suspected adverse reactions associated with medicinal products for human use. The definitions and guidance provided in Section VI.A. and the EU specific requirements presented in Section VI.C. should be followed. All applicable legal requirements are referenced in the way explained in the GVP Introductory Cover Note and are usually identifiable by the modal verb “shall”. Guidance for the implementation of legal requirements is provided using the modal verb “should”.	Guidelines		✓		✓	✓		Duplicate detection in EudraVigilance – Collaboration between the Agency, competent authorities in Member States and marketing authorisation holders - Duplicate ICSRs submitted to EudraVigilance by the same sender and identified by the Agency.
28	ArisGlobal	2019	Not applicable	USA	ArisGlobal Announces LifeSphere® MultiVigilance 10, Industry's First End-to-End Safety System With Production-Ready Automation.	LifeSphere® MultiVigilance 10 features all new architecture and design, incorporating the latest in cognitive automation technology to deliver groundbreaking efficiency gains to PV teams	Website					✓		Under tightening budgets and evolving global regulations, safety case volumes continue to grow by nearly 15% each year, creating challenges for today's drug safety teams. To address these pressures, the software leverages cognitive computing technologies such as natural language processing and machine learning, providing a single, global database that supports end-to-end automation of all adverse event processing.

No.	Author(s)	Year of publication	Journal	Country	Title	Specific Objectives	Study design	Categories						Key findings
								Extent	Prevention	Causes	Detection	Management	Implications	
29	Rajesh Ghosh *et al*	2020	Pharmaceutical	USA	Automation Opportunities in Pharmacovigilance: An Industry Survey.	This paper aims to address the need for a systematic review framework for automation of the ICSR process from the value, impact, perceived risk, and opportunity point of view.	Original reserch article					✓		In total, 15 member companies of TransCelerate participated in the survey, identifying ICSR process steps with high effort, potential for significant automation benefits, manageable automation risks, and low current automation levels as prime candidates for future automation. These steps include language translations, case verification, quality control, prioritization, data entry, alerting, workflow management, and monitoring. Conversely, certain steps like submission, already automated for some time, show lower potential for further automation. Despite successful adoption of intelligent automation in other domains, its integration into pharmacovigilance, particularly the ICSR process, remains limited, partly due to perceived risks to patient safety, which are expected to decrease with further successful applications.
30	Michael G Kahn *et al*	2016	The Journal for Electronic Health Data and Methods	USA	A Harmonized Data Quality Assessment Terminology and Framework for the Secondary Use of Electronic Health Record Data.	To describe harmonized data quality (DQ) assessment terms, methods, and reporting practices and establish a common understanding of the strengths and limitations of electronic health record (EHR) data for operational analytics, quality improvement, and research.	Review article		✓	✓				A consistent, common DQ terminology, organized into a logical framework, is an initial step in enabling data owners and users, patients, and policymakers to evaluate and communicate data quality findings in a well-defined manner with a shared vocabulary. Future work will leverage the framework and terminology to develop reusable data quality assessment and reporting methods.
31	Tomas Bergvall *et al*	2013	Drug Safety	Sweden	vigiGrade: A Tool to Identify Well-Documented Individual Case Reports and Highlight Systematic Data Quality Issues.	The objective of this study was to propose a measure of completeness and identify predictors of well-documented reports, globally.	Research article.			✓				vigiGrade distinguishes different aspects of quality according to the outline in Lindquist. An important advantage compared with the earlier implementation of documentation grading in VigiBase is that the vigiGrade completeness score considers each dimension in parallel, instead of in sequence: even when information on time-to-onset is lacking. The other dimensions are evaluated and accounted for in the total completeness score. vigiGrade considers many of the same fields as the structured assessment proposed by Agbabiaka et al. but is less comprehensive. Specifically, it does not evaluate dimensions for which absence of information cannot be distinguished from information on absence in VigiBase. On the other hand, it is a scalable solution that allows automated database-wide analyses. By design, it allows for significant penalties of a variety of missing dimensions.

No.	Author(s)	Year of publication	Journal	Country	Title	Specific Objectives	Study design	Categories						Key findings
								Extent	Prevention	Causes	Detection	Management	Implications	
32	M. Hauben *et al*	2007	Drug Safety	USA	Extreme Duplication' in the US FDA Adverse Events Reporting System Database.	The paper describes duplication as one of the numerous well-recognised forms of data corruption and distortion in spontaneous reporting systems, with duplicate detection algorithms and procedures being included in the commercial data-mining software the authors used.	Research article.				✓	✓	✓	Extreme duplication was discovered within the US FDA Adverse Events Reporting System (AERS) Database. This duplication problem in regulatory and company databases can arise from various sources and a failure to connect related cases. Vendors do make efforts to eliminate duplicate cases before providing data to customers, but sometimes the criteria used may not be sufficient for accurate identification and removal of duplicates. The impact of extreme duplication in AERS, compared to the World Health Organization (WHO) database, is evident, as it affected data-mining results in AERS but not in WHO. Duplicate reports in spontaneous reporting systems may carry unique information value and deserve further investigation. Acknowledging the limitations and challenges of spontaneous reporting system (SRS) data, including inconsistencies, errors, duplications, and incomplete data in critical fields. It's crucial to approach any findings based on SRS data with an appropriate level of caution, taking into account these limitations and the potential impact of duplication on data analysis.
33	CIOMS	2010	Not applicable	Switzerland	Practical Aspects of Signal Detection in Pharmacovigilance.	The objective of the CIOMS VIII report is to provide useful points for consideration to manufacturers, regulatory authorities, international monitoring centers, and others wishing to establish or understand the output of a systematic and holistic strategy to better manage the entire "lifecycle" of a drug safety signal. This lifecycle includes signal detection, signal prioritization, and signal evaluation.	Report			✓	✓		✓	Many databases of large spontaneous reporting systems contain duplicate reports. The identification and elimination of duplicates from analyses, therefore, is advantageous for signal evaluation. However, current duplicate detection procedures, some of which are applied prospectively (i.e. prior to data mining) and others retrospectively (i.e. after data mining), have limitations and enhanced methods of duplicate detection are being developed.
34	Kevin Pozsgai *et al*	2022	Front. Pharmacol.	Hungary	Analysis of pharmacovigilance databases for spontaneous reports of adverse drug reactions related to substandard and falsified medical products: A descriptive study.	To identify and describe the characteristics of cases that are related to adverse drug reactions potentially originating from counterfeit medication using publicly available pharmacovigilance data.	Descriptive case series			✓				The identification and elimination of duplicates between pharmacovigilance databases is almost impossible. Multiple ADRs may be presented for one individual, resulting in report duplications or triplications and making it almost impossible to eliminate this bias with manual analysis.
35	Hung E *et al*	2023	Drug Safety	USA	More extreme duplication in FDA Adverse Event Reporting System detected by literature reference normalization and fuzzy string matching.	The objective of this analysis is to determine if variations of the same literature references observed in FAERS can be resolved with text normalization and fuzzy string matching.	Research article.	✓						Normalized references can be merged via fuzzy string matching to improve enumeration of all the individual case safety reports that refer to the same article. Inclusion of the PubMed ID and adherence to the Vancouver convention could facilitate identification of duplicates in the FAERS dataset. Awareness of this phenomenon may improve disproportionality analysis, especially in areas such as addictovigilance.
36	Uppsala Monitoring Centre	2017	Uppsala Reports	Sweden	Text is key for eliminating duplicate reports.	To describe the role of text in eliminating duplicate reports.	Leading article.			✓				Duplicate Cases – Two or more records describing the same occurrence of events – same adverse effect(s) experienced with the same medical product at the same time – for the same patient. Due to reporting inaccuracies, two records can be considered duplicates even if they do not contain an identical list of events.

No.	Author(s)	Year of publication	Journal	Country	Title	Specific Objectives	Study design	Categories						Key findings
								Extent	Prevention	Causes	Detection	Management	Implications	
37	Uppsala Monitoring Centre		Reporting Fact Sheet	Sweden	Sharing pharmacovigilance data in the WHO Programme for International Drug Monitoring.	To describe the sharing of pharmacovigilance data in the WHO Programme for International Drug Monitoring.	Leading article.			✓	✓	✓		The minimum information required for an ICSR to be valid is a case identifier, reporter and patient information, and information on suspect medicine and reaction/event. All national case reports fulfilling the minimum requirements should be shared with the WHO Programme. However, quality of data and completeness of case reports are always important and reports should include as much information as possible to facilitate assessment. Free text/additional information can be provided in the original language. All information available on the original case report should be included when sending an ICSR to VigiBase, with the exception of confidential patient and reporter details that could potentially be used to identify individuals.
38	European Medicines Agency	2017		Netherlands	Guideline on good pharmacovigilance practices (GVP) Module VI Addendum I – Duplicate management of suspected adverse reaction reports.	The revision contains the following changes: - Alignment with revision 2 of GVP Module VI; - Update of electronic reporting modalities of ICSRs in the new ICH-E2B(R3) format; - Update overall with the revised pharmacovigilance legislation as regards the roles and responsibilities of the Agency, the competent authorities in Member States as well as marketing authorisation holders in relation to the operation of duplicate detection and management of reports of suspected adverse reactions; - Guidance on how to inform the Agency of suspected duplicates in EudraVigilance; - Changes for consistent presentation of GVP document.	Report		✓	✓	✓	✓	✓	Duplicate cases are generally managed through a process of merging two or more cases into one master case. This process can consist of one of the following approaches: The master case can either be based on one of the existing cases, with information from the other subordinate duplicate cases added unless the same, or more precise, information is already present in the master case (this is referred to in this document as "Allocation of a master case"), or; The master case can be created as a new case combining the information from the subordinate duplicate cases (this is referred to in this document as "Creation of a master case"). Regardless of the approach chosen, the master case should always contain all case reference numbers from all subordinate duplicate cases, such that they can be easily traced. The master case should reflect the most accurate and up-to-date information available to the organisation.
39	Agency for Medicinal Products and Medical Devices of Croatia (HALMED)	2008-2013	Not applicable	Croatia	Strengthening Collaboration for Operating Pharmacovigilance in Europe (SCOPE).	SCOPE was divided into eight separate work packages, with five work packages focusing on pharmacovigilance topics to deliver specific and measureable objectives, ranging from improvements in Adverse Drug Reaction (ADR) reporting to the assessment of quality management systems.	Report		✓	✓	✓	✓	✓	The first step in duplicate management is to identify possible ADR report duplicates. Screening for duplicates is usually carried out when a new report arrives in the database, i.e. during data entry or during the process of loading ICSRs that have been received electronically. Duplicates can be detected even before entering the case into the database – that is usually the case during manual data entry of cases received by paper, fax or phone. Duplicates can also be detected during periodic data review and the signal management process when detailed analysis of cases is performed.

No.	Author(s)	Year of publication	Journal	Country	Title	Specific Objectives	Study design	Categories						Key findings
								Extent	Prevention	Causes	Detection	Management	Implications	
40	Ritesh Bhangale *et al*	2017	Perspect Clin Res	India	A day in the life of a pharmacovigilance case processor.	The paper describes the routine experiences of a PV case processor.	Leading article.		✔					For the case processor, every case is different, even within the same study. In fatal cases, the case processor will have to check many things such as the cause of death and autopsy details, whereas in other scenarios, the processor will have to check adverse events of special interest and important medical events and report the same to stakeholders. The case processor should ensure that there are no grammatical/spelling errors in the case, while routing it to the next workflow, and shares the best practices, which helps other associates to do their job efficiently.
41	Priyanka Paygude *et al* .	2013	International Journal of Modern engineering Research	India	Automated Data Validation Testing Tool for Data Migration Quality Assurance.	The paper proposed automation of the data migration validation testing process for quality assurance and risk control across industries.	Research article.		✔					Data migration projects pose significant challenges and risks, e.g., time overruns and budget constraints. The proposed system ensures data quality through standardized testing methods across the enterprise. This solution could help organizations save time, reduce costs, minimize manual efforts, and guarantee data quality assurance throughout the migration process.
42	Saqib Hakak *et al*	2018	Plos One	Malaysia	A new split based searching for exact pattern matching for natural texts.	This work proposes a novel idea to achieve both time efficiency and memory consumption by splitting query string for searching in Corpus.	Research article.				✔			This paper proposed a novel idea for exact string matching to achieve both time and space efficiency regardless of query pattern length, dataset size and scripts.
43	Christen P	2012	SpringerLink.	UK	Data matching: concepts and techniques for record linkage, entity resolution, and duplicate detection. Springer: Data-centric systems and applications.	First book on a topic of growing importance for applications. Brings together research from various areas like databases, statistics, information retrieval, data mining, and machine learning. Details the data matching process step by step. Includes an overview of freely available data matching systems and a detailed discussion of practical aspects and limitations.	Book				✔			By providing the reader with a broad range of data matching concepts and techniques and touching on all aspects of the data matching process, this book helps researchers as well as students specializing in data quality or data matching aspects to familiarize themselves with recent research advances and to identify open research challenges in the area of data matching. To this end, each chapter of the book includes a final section that provides pointers to further background and research material.
44	Liu H *et al* .	2014	EANN	UK	Categorization and Construction of Rule Based Systems.	This paper aims to introduce the theory of rule-based systems especially on categorization and construction of such systems from a conceptual point of view. This also details rule-based systems for classification tasks.	Conference paper				✔			This introduces novel insights for both researchers and practitioners, presenting a new paradigm for rule-based systems in various applications. It advocates for a data-driven approach within machine learning for constructing complex systems, contrasting with traditional knowledge-based methods. Emphasizing the significance of the data-driven approach, it delves into a specialized classification rule-based system, detailing its construction framework and crucial considerations in method selection for rule generation, simplification, and representation operations.
45	Wong CK *et al*	2015	Drug Safety	Australia	Standardisation of the FAERS database: a systematic approach to manually recoding drug name variants	The aim of this study was to develop and implement a data cleaning protocol to identify and resolve drug nomenclature issues. The key 'data treatment' plan involved standardising drug names held in the FAERS database.	Research article.				✔	✔		The millions of reports enclosed in the FAERS contain valuable information that is of interest to pharmacovigilance, toxicology and post-marketing surveillance researchers. With the standardisation of the drug nomenclature, the database can be better utilised by research groups around the world.

No.	Author(s)	Year of publication	Journal	Country	Title	Specific Objectives	Study design	Categories						Key findings
								Extent	Prevention	Causes	Detection	Management	Implications	
46	Jae Dong Yang.	1997	Data & Knowledge Engineering	South Korea	A fuzzy match framework for rule-based programming.	To propose a framework to support a semantic based inexact match with Fuzzy Match Predicate (F_MP).	Research article.				✓			The paper demonstrates that: (1) F_MP is a uniform framework to provide the rule-based languages with fuzzy match facilities semantically enhanced, and that (2) its semantics conform well to those of the relational one.
47	Kreimeyer K *et al*	2022	Drug Safety	USA	Increased Confidence in Deduplication of Drug Safety Reports with Natural Language Processing of Narratives at the US Food and Drug Administration.	To improve and optimize an existing deduplication algorithm that used both structured and free-text data; develop a web-based application to support data processing; and conduct a 6-month dedicated evaluation to assess the potential operationalization of the deduplication process in the FDA.	Research article.				✓	✓	✓	Comparing algorithm predictions with reviewer determinations of duplicates for twenty-seven files for case series reviews (with a median size of 281 reports), the average pairwise recall and precision were 0.71 (SD ± 0.32) and 0.67 (SD ± 0.34). Overall, reviewers felt confident about the algorithm and expressed their interest in using it. These findings support the operationalization of the deduplication process for case series review as a supplement to human review.
48	Kreimeyer K *et al*	2017	Drug Safety	USA	Using Probabilistic Record Linkage of Structured and Unstructured Data to Identify Duplicate Cases in Spontaneous Adverse Event Reporting Systems.	To develop a probabilistic record linkage algorithm for identifying duplicate cases in the US Vaccine Adverse Event Reporting System (VAERS) and the US Food and Drug Administration Adverse Event Reporting System (FAERS).	Research article.				✓		✓	The algorithm was shown to be effective at identifying pre-linked duplicate VAERS reports. The narrative text was not shown to be a key component in the automated detection evaluation; however, it is essential for supporting the semi-automated approach that is likely to be deployed at the Food and Drug Administration, where medical reviewers perform some manual review of the most highly ranked reports identified by the algorithm.
49	Harpaz R *et al*	2017	Journal of Bioinformatics.	USA	Toward multimodal signal detection of adverse drug reactions.	Improving mechanisms to detect adverse drug reactions (ADRs) is key to strengthening post-marketing drug safety surveillance. Signal detection is presently unimodal, relying on a single information source. Multimodal signal detection is based on jointly analyzing multiple information sources. Building on, and expanding the work done in prior studies, the aim of the article is to further research on multimodal signal detection, explore its potential benefits, and propose methods for its construction and evaluation.	Research article.				✓			The results support the notion that utilizing and jointly analyzing multiple data sources may lead to improved signal detection. Given certain data and benchmark limitations, the early stage of development, and the complexity of ADRs, it is currently not possible to make definitive statements about the ultimate utility of the concept. Continued development of multimodal signal detection requires a deeper understanding of the data sources used, additional benchmarks, and further research on methods to generate and synthesize signals.
50	Kreimeyer K *et al*	2021	Computers in Biology and Medicine	USA	Feature engineering and machine learning for causality assessment in pharmacovigilance: Lessons learned from application to the FDA Adverse Event Reporting System.	To support the automated classification of Food and Drug Administration (FDA) Adverse Event Reporting System (FAERS) reports for their usefulness in assessing the possibility of a causal relationship between a drug product and an adverse event.	Research article.				✓			Causal inference from FAERS reports depends on many components with complex logical relationships that are yet to be made fully computable. Efforts focused on readily addressable tasks, such as quickly eliminating unassessable reports, fit naturally in SE's thought processes to provide real enhancements for FDA workflows.
51	Kim HR *et al*	2022	Medicine	South Korea	Analyzing adverse drug reaction using statistical and machine learning methods: A systematic review.	This systematic review aimed to examine the analytical tools by considering original articles that utilized statistical and machine learning methods for detecting ADRs.	Systematic review				✓			This review provides guidelines on which databases are frequently utilized and which analysis methods can be connected. For statistical analysis, >90% of the cases were analyzed by disproportionate or regression analysis of each spontaneous reporting system or electronic medical record database; for machine learning research, however, there was a strong tendency to analyze various data combinations. Only half of the DrugBank database was occupied, and the k-nearest neighbor method accounted for the greatest proportion.

No.	Author(s)	Year of publication	Journal	Country	Title	Specific Objectives	Study design	Categories						Key findings
								Extent	Prevention	Causes	Detection	Management	Implications	
52	CIOMS	2021	Website	Sweden	Making medicines safer for patients everywhere.	This is the fourth of a series of special newsletters that describe the context of CIOMS activities and the work of its member organizations and partners. It describes what the Uppsala Monitoring Centre (UMC) is doing to support and promote patient safety through effective global pharmacovigilance practice.	Not applicable				✓	✓		UMC promotes collaboration between all stakeholders involved in drug risk management and supports harmonization, good pharmacovigilance practice and the dissemination of new guidelines. CIOMS is an important partner in this regard. New working groups on pharmacovigilance topics are needed, for example to explore the link between pharmacovigilance practices and public health, or the role of pharmacoepidemiology as a tool to assess the impact of emerging adverse effects.
53	MedDRA		Website	Not applicable	MeDRA History	In the 1990s, the International Conference on Harmonisation (ICH) recognized the necessity for standardized medical terminology. At that time, available terminologies like COSTART, WHO-ART, J-ART, H-ARTS, ICD-9, and ICD-9CM lacked the required scope and granularity for regulatory authorities and industry needs. Due to infrequent updates, users often created customized versions, leading to a loss of standardization.	Not applicable					✓		MedDRA was based on terminology belonging to the Medicines and Healthcare products Regulatory Agency (MHRA) of UK (previously named Medicines Control Agency) and was developed using the ICH process by the ICH partners, including WHO.
54	Oracle.	2021	Website	USA	Argus Trusted safety case management.	To describe how to promote efficient case management while reducing the manual effort of a safety and pharmacovigilance team.	Not applicable					✓		Oracle Argus enabled greater and faster insight into product safety, improved productivity, and streamlined and automated safety reporting.
55	Ablebits.	2023	Website	Poland	Ablebits. Ablebits Ultimate Suite.	To describe the tools that can automate most daily tasks in Excel and increase productivity by at least 50%	Not applicable					✓		Finding and removing duplicates in Excel may be a real headache, especially when worksheets contain thousands of rows. Duplicate Remover for Excel does the job quickly and perfectly – it can find and delete duplicates based on one or several key columns, identify unique values, highlight, copy or move the found entries.
56	Brinker AD *et al*	2002	Am J Hematol.	USA	Spontaneous reports of thrombocytopenia in association with quinine: clinical attributes and timing related to regulatory action.	This report includes clinical attributes from the largest case series to date of apparently isolated thrombocytopenia in association with quinine and trends in the receipt of spontaneous adverse event reports to FDA's Center for Drug Evaluation and Research (CDER) for this drug-event combination in relation to regulatory action. In this study, we reviewed reports of spontaneous adverse drug events received by CDER.	Descriptive study						✓	This case series confirms previous smaller series that suggest quinine-associated thrombocytopenia may present rapidly with symptoms of profound thrombocytopenia. Clinicians evaluating patients with new-onset and apparently idiopathic thrombocytopenia should maintain clinical vigilance for ingestion of quinine and elicit a detailed food/dietary supplement history from the patient.
57	Azeroual O *et al* .	2022	Multimodal Technol. Interact.	Germany	A Record Linkage-Based Data Deduplication Framework with DataCleaner Extension.	To make the proposed framework usable we integrated it into a tool that is already used in practice, by developing a prototype of an extension for the well-known DataCleaner. The framework detects and visualises duplicates thereby identifying and providing the user with identified redundancies in a user-friendly manner allowing their further elimination. By removing the redundancies, the quality of the data is improved therefore improving analyses and decision-making. This study makes a call for other researchers to take a step towards the "golden record" that can be achieved when all data quality issues are recognised and resolved, thus moving towards absolute data quality.	Research Article.				✓		✓	This paper, presents a Record Linkage framework consisting of six interconnected steps, i.e., (1) data preparation, (2) search space definition, (3) attribute value comparison, (4) a decision model, (5) clustering of the duplicates and (6) verification. It then transforms into an extension for the DataCleaner tool that follows validation through its application to the real-world RIS. This allows for easy and automatic identification of duplicates stored in the system, with their subsequent elimination. This enriches the original records and thereby contributes to their completeness, thus preparing the data for further processing.

No.	Author(s)	Year of publication	Journal	Country	Title	Specific Objectives	Study design	Categories						Key findings
								Extent	Prevention	Causes	Detection	Management	Implications	
58	Norén GN *et al* .	2007	Data Mining and Knowledge Discovery.	Sweden	Duplicate detection in adverse drug reaction surveillance.	We propose a duplicate detection method based on the hit-miss model for statistical record linkage described by Copas and Hilton, which handles the limited amount of training data well and is well suited for the available data (categorical and numerical rather than free text). We propose two extensions of the standard hit-miss model: a hit-miss mixture model for errors in numerical record fields and a new method to handle correlated record fields.	Research Article.				✓			This paper introduced two generalisations of the standard hit-miss model and demonstrated the usefulness of the extended hit-miss model for automated duplicate detection in WHO drug safety data. The results indicate that the hit-miss model can detect a significant proportion of the duplicates without generating many false leads. Its strong theoretical basis together with the excellent results, should make it a strong candidate for other duplicate detection and record linkage applications.
