# Supplementary material for: Navigating duplication in pharmacovigilance databases: a scoping review
Source: BMJ Open. 2024 Apr 29;14(4):e081990. doi: 10.1136/bmjopen-2023-081990 (PMC11086478; doi:10.1136/bmjopen-2023-081990)
Supplement: Author's manuscript [file bmjopen-2023-081990.draft_revisions.pdf]

BMJ Open

BMJ Open is committed to open peer review. As part of this commitment we make the peer review history of every article we publish publicly available.

When an article is published we post the peer reviewers' comments and the authors' responses online. We also post the versions of the paper that were used during peer review. These are the versions that the peer review comments apply to.

The versions of the paper that follow are the versions that were submitted during the peer review process. They are not the versions of record or the final published versions. They should not be cited or distributed as the published version of this manuscript.

BMJ Open is an open access journal and the full, final, typeset and author-corrected version of record of the manuscript is available on our site with no access controls, subscription charges or pay-per-view fees (http://bmjopen.bmj.com).

If you have any questions on BMJ Open's open peer review process please email info.bmjopen@bmj.com

BMJ Open

Navigating Duplication in Pharmacovigilance Databases: A Scoping Review

Journal:	*BMJ Open*
Manuscript ID	bmjopen-2023-081990
Article Type:	Original research
Date Submitted by the Author:	11-Nov-2023
Complete List of Authors:	Kiguba, Ronald; Makerere University, Department of Pharmacology and Therapeutics<br>Isabirye, Gerald; National Drug Authority<br>Mayengo, Julius; National Drug Authority<br>Owiny, Jonathan; National Drug Authority<br>Tregunno, Phil; Medicines and Healthcare Products Regulatory Agency<br>Harrison, Kendal; Medicines and Healthcare Products Regulatory Agency<br>Pirmohamed, Munir; The University of Liverpool, Department of Pharmacology<br>Ndagije, Helen ; National Drug Authority, Kampala
Keywords:	Adverse events < THERAPEUTICS, Pharmacology < TROPICAL MEDICINE, CLINICAL PHARMACOLOGY

SCHOLARONE™
Manuscripts

BMJ

I, the Submitting Author has the right to grant and does grant on behalf of all authors of the Work (as defined in the below author licence), an exclusive licence and/or a non-exclusive licence for contributions from authors who are: i) UK Crown employees; ii) where BMJ has agreed a CC-BY licence shall apply, and/or iii) in accordance with the terms applicable for US Federal Government officers or employees acting as part of their official duties; on a worldwide, perpetual, irrevocable, royalty-free basis to BMJ Publishing Group Ltd ("BMJ") its licensees and where the relevant Journal is co-owned by BMJ to the co-owners of the Journal, to publish the Work in this journal and any other BMJ products and to exploit all rights, as set out in our licence.

The Submitting Author accepts and understands that any supply made under these terms is made by BMJ to the Submitting Author unless you are acting as an employee on behalf of your employer or a postgraduate student of an affiliated institution which is paying any applicable article publishing charge ("APC") for Open Access articles. Where the Submitting Author wishes to make the Work available on an Open Access basis (and intends to pay the relevant APC), the terms of reuse of such Open Access shall be governed by a Creative Commons licence – details of these licences and which Creative Commons licence will apply to this Work are set out in our licence referred to above.

Other than as permitted in any relevant BMJ Author's Self Archiving Policies, I confirm this Work has not been accepted for publication elsewhere, is not being considered for publication elsewhere and does not duplicate material already published. I confirm all authors consent to publication of this Work and authorise the granting of this licence.

For peer review only

Navigating Duplication in Pharmacovigilance Databases: A Scoping Review

Authors:

Ronald Kiguba[1], Gerald Isabirye[2], Julius Mayengo[2], Jonathan Owiny[2], Phil Tregunno[3], Kendal Harrison[3], Munir Pirmohamed[4], Helen B. Ndagije[2]*

*Correspondence to: Ronald Kiguba (Tel: +256 701 840683; Email: kiguba@gmail.com)

Affiliations:

[1]Department of Pharmacology and Therapeutics, College of Health Sciences, Makerere University, Kampala, Uganda

[2]National Pharmacovigilance Centre, National Drug Authority, Kampala, Uganda

[3]Safety and Surveillance Group, Medicines and Healthcare products Regulatory Agency, London, United Kingdom

[4]Centre for Drug Safety Science and Wolfson Centre for Personalised Medicine, Institute of Systems, Molecular and Integrative Biology, University of Liverpool, Liverpool, United Kingdom

Running Head:

Duplication in Pharmacovigilance Databases

For peer review only

Abstract

Objectives:

Pharmacovigilance databases are crucial for drug safety monitoring. The duplication of reports in these databases, however, undermines data integrity and affects the reliability of pharmacovigilance activities. This scoping review sought to provide a comprehensive understanding of duplication in pharmacovigilance databases worldwide.

Inclusion criteria:

This scoping review included peer-reviewed publications and grey literature describing duplication and/or methods relevant to duplication in pharmacovigilance databases.

Methods:

We used the Joanna Briggs Institute guidelines for scoping reviews and conformed with the Preferred Reporting Items for Systematic Reviews and Meta-Analyses Extension for Scoping Reviews. Reviewers comprehensively searched the literature in PubMed, Embase, Web of Science, Google Scholar, and relevant websites from inception to 1 September 2023. Two reviewers independently screened titles, abstracts, and full-texts. One reviewer extracted the data and performed descriptive analysis, which the second reviewer assessed. Disagreements were resolved by discussion and consensus or in consultation with a third reviewer.

Results:

We screened 2776 unique titles and 156 were eligible for full-text review. Of the 156 titles, 58 (47 peer-reviewed; 11 grey literature) fulfilled the inclusion criteria for the scoping review. The included titles addressed the extent of duplication (5 papers), prevention strategies (15 papers), causes (32 papers), detection methods (24 papers), management strategies (24 papers), and implications (14 papers) of duplicate reports in pharmacovigilance databases. The papers overlapped, discussing more than one field. Advances in artificial intelligence, particularly natural language processing, hold promise in enhancing the efficiency and precision of deduplication of large and complex pharmacovigilance databases.

Conclusion:

This review suggests that duplication in pharmacovigilance databases compromises risk assessment and decision-making, potentially threatening patient safety. Therefore, efficient duplicate prevention, detection, and management is essential for accurate and reliable pharmacovigilance data. Recent advances in artificial intelligence will significantly improve efficiency, accuracy, and consistency in the detection and management of duplicate reports.

Keywords: Adverse event reporting, Duplication, Duplicate management, Pharmacovigilance, Pharmacovigilance database.

For peer review only - http://bmjopen.bmj.com/site/about/guidelines.xhtml

Article summary

Strengths and limitations of this study:

- We conducted a comprehensive literature search of peer-reviewed publications across several electronic databases, combining the results with evidence found in the grey literature.
- This review adhered to the rigorous methodology published by the Joanna Briggs Institute and the Preferred Reporting Items for Systematic Reviews and Meta-Analyses Extension for Scoping Reviews.

For peer review only

For peer review only - http://bmjopen.bmj.com/site/about/guidelines.xhtml

INTRODUCTION:

Pharmacovigilance is crucial for drug safety as it promotes the prevention, detection and evaluation of suspected adverse reactions to minimize their impact on patient health[1,2]. One of the challenges of pharmacovigilance is the duplication of cases in pharmacovigilance databases, which hinders accurate risk assessment and decision-making[3-5]. From 2000 to 2010, about 2.5% of reports in the World Health Organization (WHO) global pharmacovigilance database were duplicates; the percentage was higher for reports from the literature (11%) and those with fatal outcomes (5%)[4]. Duplication is defined as multiple, unconnected records that refer to the same potential adverse event[3,4,6]. Duplicate reporting leads to inaccurate drug safety and efficacy assessments, which could have significant implications for patient safety. To promote the accuracy of pharmacovigilance databases, it is important to routinely screen and remove duplicate cases[4]. Global, regional, and country-level guidelines have been implemented to address duplication in pharmacovigilance databases.

Global pharmacovigilance initiatives, such as the International Council for Harmonization (ICH) guidelines and the Uppsala Monitoring Centre, which coordinates the WHO programme for international drug monitoring, promote standardization and harmonization of pharmacovigilance activities, which are essential for accurate and efficient data collection, analysis, and reporting[2,7-10]. In the US, the Food and Drug Administration (FDA) uses the Adverse Event Reporting System (FAERS) to manage adverse event reports, including duplicates[11,12]. In Europe, the European Medicines Agency (EMA) oversees the safety of medicines via the EudraVigilance system which uses unique identification numbers for each report and has a built-in deduplication mechanism to identify and eliminate duplicate reports[13,14]. In Asia, a deduplication technique was adopted by the Pharmacovigilance Program of India, together with the assignment of unique identification numbers and data standardization. Similar to this, in China, the National Medical Products Administration has put in place a system called the Adverse Drug Reaction Monitoring and Re-evaluation System that incorporates a deduplication mechanism for reporting and monitoring suspected adverse reactions[15]. In Africa, WHO built a regional pharmacovigilance centre in Ghana to support the region's pharmacovigilance systems through training, including duplicate management[16]. Pharmaceutical companies are also responsible for managing duplicate reports. Comprehensive and integrated pharmacovigilance systems are required to effectively manage data from multiple sources, e.g. electronic health records, spontaneous reporting databases, social media, and digital devices, among others; and to eliminate duplicate reports[17,18]. These systems should ensure compliance with the global pharmacovigilance regulations and standards.

Duplicates arise from various causes including data entry errors[19,20], multiple reporters and reporting channels[21,22], pressure from mandatory reporting requirements for healthcare professionals and pharmaceutical companies[4,23], challenges in data integration and harmonization in pharmacovigilance databases[18], multiple representation of the same data due to the merging of pharmacovigilance databases[6], lack of standardized data management practices[6], and incomplete case information of key data elements[11,24].

The implications of duplication are far-reaching, hence the need for the routine screening and elimination of duplicates from pharmacovigilance databases[6]. Software programs significantly improve efficiency, accuracy, and consistency in the detection and management of duplicates. The Uppsala Monitoring Centre developed the software VigiMatch to detect potential duplicates in VigiBase, the global pharmacovigilance database[25]. VigiBase can merge duplicate reports identified by VigiMatch[4,26]. Automated standalone software programs have been developed to handle large volumes of data and can be customized to meet specific regulatory requirements[27-29].

For peer review only - http://bmjopen.bmj.com/site/about/guidelines.xhtml

Advances in technology, e.g. machine learning and natural language processing hold promise in augmenting the efficiency and precision of pharmacovigilance efforts, particularly in the deduplication of large and complex pharmacovigilance databases[5,30,31]. Due to the likely occurrence of false signals, however, over-reliance on automated standalone software programs should be discouraged in favour of holistic methods which include manual review[32,33]. The objective of this scoping review is to provide a comprehensive overview of duplication in pharmacovigilance databases on a global scale. This review addresses the extent, causes, prevention strategies, detection methods, management and impact of duplication in pharmacovigilance databases.

METHODS

Research Question:

1. What is the extent of duplication in pharmacovigilance databases worldwide?
2. What are the causes of, and prevention strategies, detection methods and management strategies for duplicate reports in pharmacovigilance databases worldwide?
3. What is the impact of duplicate reports in pharmacovigilance databases worldwide?

Stud Design:

This scoping review was conducted in accordance with the Joanna Briggs Institute (JBI) guidelines for scoping reviews[34] and conformed with the Preferred Reporting Items for Systematic Reviews and Meta-Analyses Extension for Scoping Reviews (PRISMA-ScR)[35]. The protocol was developed according to the JBI scoping review template.

Eligibility Criteria:

The inclusion criteria for the selection of articles, reports, and guidelines were as follows:

1. Articles, reports, and guidelines related to duplication and/ or methods relevant to duplication in pharmacovigilance databases
2. Articles published in peer-reviewed journals and grey literature from official websites of regulatory authorities and pharmacovigilance organizations.

Articles, reports, and guidelines which, on full-text review, did not align with the precise objectives of the scoping review and were therefore considered out of scope were excluded. This rigorous screening procedure was used to ensure that only relevant and excellent papers were eventually included in the analysis.

Search strategy:

The search strategy was designed to identify all relevant studies related to the research question(s). We therefore searched multiple databases, including PubMed, Embase, Google Scholar, and official websites of regulatory authorities and pharmacovigilance organizations, from inception to 1 September 2023. The reference lists of relevant studies were also screened. Reviewers searched the literature comprehensively in PubMed, Embase, Web of Science, Google Scholar, and relevant websites. The PubMed search terms for peer-reviewed publications are detailed in **Appendix I**. We adapted the search strategy for each peer-reviewed research database using the Polyglot Search Translator[36]. The grey literature search was based on the Canadian Agency for Drugs and Technologies in Health Guide[37] (see **Appendix II)**.

For peer review only

For peer review only - http://bmjopen.bmj.com/site/about/guidelines.xhtml

Study selection:

Two reviewers independently screened the titles and abstracts of all the identified articles for eligibility. Full-text articles were retrieved for all potentially eligible studies and independently assessed for inclusion by two reviewers. Disagreements were resolved by discussion and consensus or in consultation with a third reviewer.

Data extraction:

Data extraction was done by Gerald Isabirye (GI) using a tool created by the reviewers, as detailed in **Appendix III**. This tool was designed to capture various parameters, including the Author, Year of publication, Journal, Country, Title, Specific Objectives, Study design, Categories (extent, prevention, causes, detection, management and impact of duplication in pharmacovigilance databases), and key findings. The extracted data were reviewed by Ronald Kiguba (RK) and a third reviewer, Helen B. Ndagije (HBN), and revised accordingly.

Data analysis and presentation:

The extracted data are provided in a descriptive summary according to the review's objectives. The risk of bias or quality assessment was not done across the included studies. This decision aligns with the guidelines outlined by the JBI because a scoping review is not intended to appraise the risk of bias of a cumulative body of evidence[35]. Instead, it focuses on providing a comprehensive overview of the existing literature.

Patient and public involvement:

Patients and the public did not participate in the design, conduct, reporting and drafting of the dissemination plans of this research.

Regions and countries:

The regions and countries of papers included in this review are presented in **Appendix IV**. The papers were from the Asia Pacific region, Europe, Australia and North Africa.

For peer review only - http://bmjopen.bmj.com/site/about/guidelines.xhtml

RESULTS

We screened 2776 unique titles and 156 were eligible for full-text review. Of the 156 titles, 58 (47 peer-reviewed; 11 grey literature) fulfilled the inclusion criteria for the scoping review (see **Tables 1 & 2** and **Figure 1**). The included titles addressed the extent of duplication (5 papers), prevention strategies (15 papers), causes (32 papers), detection methods (24 papers), management (24 papers) and implications (14 papers) of duplicate reports. The papers overlapped, discussing more than one field. The list of excluded titles is provided in **Appendix V**.

Extent of duplication: The prevalence of duplication in the WHO global pharmacovigilance database, VigiBase, was 2.5% for reports submitted from 2000 to 2010, being highest for reports from published literature (11%) and fatal outcomes (5%)[4]. In Europe, the prevalence of duplication in VigiBase for three countries was 1.4% in the United Kingdom, 1% in Denmark and 0.7% in Spain[4]. In the US, duplicates represented 5-20% of reports in the FAERS up to the year 2000[12]. A very high level of duplication, from 66% to 87%, was observed in the FAERS database for reports from the literature during the first quarter of 2021[38]. In specific drug-event pair analysis, duplicates accounted for 8% (287/3572) of clozapine-myocarditis cases in VigiBase from inception to January 2021 and for 20% (28/141) of quinine-induced thrombocytopenia cases in the FAERS from 1974 to 2000[39,40]. In Africa and Asia, information was scarce on the extent of duplication in pharmacovigilance databases.

Causes of duplication: Duplicates in pharmacovigilance databases arise from various causes including: 1) data entry errors, e.g. i) submission of follow-up reports of the same case with new identification numbers[4], ii) multiple entries of the same case by different data entrants[20,41], and iii) misspelling the name of a drug or patient[20]; 2) multiple reporters e.g. patients, healthcare professionals or pharmaceutical companies[9,21,41,42]; 3) pressure from mandatory reporting requirements for healthcare professionals and pharmaceutical companies[4,23,43,44]; 4) challenges in data integration and harmonization in database software, e.g. technical glitches that could occur during i) data synchronization, ii) data import/export, or iii) the integration of different databases[25,45]; 5) multiple representation of the same data due to the merging of data from different databases such as regulatory authorities, pharmaceutical companies and clinical trials[6]; 6) lack of standardized data management practices characterized by inconsistencies in data entry standards and coding systems[6], and 7) incomplete or missing case information of key data elements, e.g. patient identifiers[24,46,46].

Strategies to minimize duplication: Several papers described best practices for minimizing duplication. These strategies include standardized data entry to enable consistent recording of adverse event data, making it easier to identify duplicates[45,47,48]; regular data cleaning and deduplication[47]; assigning unique identifiers to patients and adverse events[3,6]; automated and semi-automated search criteria based on similarities in patient demographics (age, gender), unique identifiers, adverse reactions, and suspected medicines[4,6,47,49]; data validation and quality assurance processes[50,51]; collaboration between health facilities, pharmaceutical companies, and patients[52,53]; and, data sharing between regulatory authorities and international databases[52,53].

Detection of duplicates: Twenty-four papers discussed the detection of duplicates. Pharmacovigilance databases should be reviewed routinely for duplicates and screening for duplicates should be a priority when new reports are submitted[6,54]. Automated or semi-automated detection systems are recommended to identify duplicates during both manual and electronic data capture[6,47]. To detect duplicates in small databases, reports may be sorted into simple tables by case ID numbers, patient characteristics (age, sex), adverse reaction(s) or suspected medicine(s). For large databases, however, the flagging of potential duplicates is largely automated, and is frequently followed up with manual review particularly to confirm inconclusive duplicates detected by the automated methods[28]. Duplicate detection algorithms should, however, be validated and fine-tuned

For peer review only - http://bmjopen.bmj.com/site/about/guidelines.xhtml

periodically because the level of detail in submitted reports could vary with time, e.g. if data fields become mandatory, these variables ought to be included in the algorithms[6,25,55].

Approaches to duplicate detection:

Exact matching: This is a straightforward method which compares two records to identify precise matches (exact duplicates) based on key data fields such as the report ID, patient demographics, name of adverse event, onset date of the adverse event, and name of suspected drug, among others[56,57].

Rule-based matching: This method defines specific conditions to identify and eliminate potential duplicates based on predefined criteria informed by domain knowledge, historical data and observed patterns in the data. It allows for more targeted identification of duplicates based on known patterns, e.g., the same case with the same adverse event reported several times in a short period[58]. These rules are typically based on particular fields in the data, e.g. patient's name, date of birth, or drug name, and are intended to match reports that have similar data[59,60]. Simple duplicates can often be found using rule-based systems, but more complex duplicates may be missed because they require more complicated algorithms[58].

Fuzzy matching: This technique identifies records that are similar though not necessarily identical. Unlike exact matching, which requires an exact match between data fields, fuzzy matching allows for variations, misspellings, and small differences in the data. This is particularly useful where the data may be inconsistently entered or contain errors, frequently in real-world databases[61]. Fuzzy matching algorithms assess the degree of resemblance between two records using a variety of similarity metrics, including Levenshtein distance and Jaro-Winkler distance. Fuzzy matching algorithms are frequently used in pharmacovigilance to identify duplicates that contain distinct patient names, medical terms, or drug names spelled differently or abbreviated differently[61].

Machine learning and natural language processing: This approach employs supervised machine learning models to train algorithms using labelled data that differentiate duplicate and non-duplicate pairs. These models then use the knowledge gained from labelled data to predict whether new record pairs are duplicates or not based on learned patterns[5,62,63,64]. Natural language processing techniques have been developed to augment computerised duplicate detection using the narrative in adverse event reports[64]. These models can be more precise than rule-based systems, particularly when working with large and complex datasets. The performance of machine learning models can be improved over time as they gain knowledge from fresh instances and adapt to changing data[17,53]. Machine learning models are trained on extensive datasets of adverse event reports to identify patterns and connections between various factors, including drug names, patient demographics, and symptoms[65]. Once trained, these models can be used to automatically identify and categorize duplicates based on a variety of factors, including the similarity of medication names, the occurrence of specific medical conditions, and the timing and severity of symptoms[66,67]. Machine learning models can be very accurate and efficient in finding complex duplicates; however, their creation and maintenance demands a significant quantity of high-quality training data and specialized knowledge[68,69].

Probabilistic record linkage: This method identifies and remove duplicate reports from large and complex datasets. An example is the hit-miss model which uses computational methods to analyze extensive healthcare databases. It distinguishes "hits" (adverse events) from "misses" (non-events) by identifying patterns and signals[3,47]. The method relies on statistical techniques to estimate the probability that two reports refer to the same event or individual, considering various data fields, such as patient name, age, and drug name. Probabilistic record linkage is particularly useful when dealing with datasets that are incomplete, inconsistent, or have missing data fields because they can still produce reliable results. However, this method can be computationally intensive and requires substantial computational resources, making it less practical for smaller datasets or less well-resourced pharmacovigilance systems[4].

For peer review only - http://bmjopen.bmj.com/site/about/guidelines.xhtml

Manual review: This method is used to confirm reports that have been flagged as potential duplicates. Manual review is used in instances where automated methods are inconclusive[6,47].

Confirmation of duplicates: Manual confirmation is always necessary following the detection of potential duplicates. Well documented reports, with elaborate case narratives, make the confirmation process easier[5,62,64]. However, reports with limited information require that reporters are contacted for additional information, which necessitates the timely review of submitted reports[6]. When duplicates are detected, root cause analysis should be done and corrective action taken. Lastly, confirmed duplicates should be managed appropriately[6].

Management of duplicates: This is the process of merging two or more reports to form one master report. Two approaches could be used: i) allocating an existing report as the master report, with the information from other reports being added unless the same or more precise information is already present in the master report, and ii) creating a master report by combining the information from several duplicate reports[6,20]. The master report should always include the case reference numbers of all subordinate duplicate reports for easy traceability. Subordinate duplicates remain in the database for the purpose of audit trail but are not used for any other pharmacovigilance purpose. One challenge with duplicate management is when conflicting information is provided by different reporters. Clarification of conflicting information should be obtained wherever possible[6].

Software programs: These are valuable tools for deduplication in pharmacovigilance databases. VigiMatch is a software program developed by the Uppsala Monitoring Centre to detect potential duplicates in the global database of adverse event reports, VigiBase[25,70]. VigiMatch compares various data elements of adverse event reports and assigns a matching score to each pair of reports[4]. Although VigiMatch and VigiBase are separate programs, they are linked in the sense that VigiMatch is often used as a tool to identify and consolidate potential duplicate reports within VigiBase. VigiBase includes data cleaning and deduplication tools that use various criteria to identify potential duplicates, including patient information, drug information, and adverse event descriptions. VigiBase can merge duplicate reports identified by VigiMatch, ensuring that the database remains accurate and efficient[47]. VigiMatch is available for use by national pharmacovigilance centers and regulatory authorities worldwide. However, access to the software depends on various factors, such as the availability of funding, infrastructure, and technical expertise to effectively implement the software[70].

Other software packages have been developed. The Medical Dictionary for Regulatory Activities (MedDRA) coding system is a standardized software for regulatory reporting that can identify duplicates and minimize data entry errors[71]. ArisGlobal's LifeSphere is a cloud-based pharmacovigilance software with a deduplication module to identify and consolidate duplicate reports[72]. Oracle's Argus Safety is a comprehensive safety management system with a deduplication feature to eliminate duplicate reports and improve data accuracy[73]. Ablebits Ultimate Suite of tools is software with a variety of *add-ins* and *plugins* for Microsoft Office Excel and Google Sheets. The software has a range of tools, including the "Remove Duplicates" feature that allows users to find and eliminate duplicate entries in a dataset. The primary purpose of Ablebits is to enhance the functionality of spreadsheet programs and make it easier for users to perform various tasks. Ablebits offers a range of tools that can automate repetitive tasks, format data, merge cells, and compare data, etc. The software can also merge duplicate reports to create a single case record, thereby reducing manual review and data entry. The goal of Ablebits is to help users save time and improve their productivity while working with Excel or Google Sheets[74].

Advantages and limitations of software programs: The **advantages** of using software programs for deduplication include improved efficiency, accuracy, and consistency in identifying and managing duplicates. These programs can also handle large volumes of data and can be customized to meet specific regulatory

For peer review only - http://bmjopen.bmj.com/site/about/guidelines.xhtml

requirements[48]. Using Excel with the Ablebits Ultimate Suite of tools could be more cost-effective for small datasets – the software is accessible and easy to use particularly if the user is familiar with Excel. However, Ablebits may not be as efficient or accurate as the dedicated software programs, particularly for large and complex pharmacovigilance databases.

However, there are also **limitations** to using software programs for deduplication. These programs may require extensive training and expertise to operate effectively, and might not detect all potential duplicates owing to variations in data entry or differences in data elements used for comparison. Additionally, the cost of implementing and maintaining these software programs may be a barrier for some regulatory authorities, especially in low- and middle-income countries. Artificial intelligence algorithms need to be trained with large quantities of high-quality data. The technical challenges of artificial intelligence-based pharmacovigilance, particularly in low- and middle-income countries, are the lack of high-quality databases, insufficient human resources, weak artificial intelligence technology, data sharing and privacy challenges, transparency of algorithms, interoperability across multiple platforms and insufficient support from governments[27,32,69,75-77].

Impact of duplication:

Inaccurate risk assessment: Duplicates in pharmacovigilance databases can lead to overestimation or underestimation of the frequency and severity of adverse events, introduce inconsistencies in data analysis and reporting, distort the understanding of the safety profile of medicines, and hinder signal detection and risk assessment. This compromises the integrity and reliability of databases as sources of accurate information, and potentially impacts important decisions related to drug labelling, risk management, and patient safety[3,4,6,12,20,47,62,78]. As an example, Brinker et al.[40] observed an exaggerated safety signal of quinine-induced thrombocytopenia as a result of duplicate reports.

Resource wastage: Duplicates consume valuable database storage space, computing resources, and human effort. Maintaining and managing duplicate records uses up resources, which could be better utilized for other critical tasks[4,78-80]. Having duplicate entries necessitates additional manual effort to identify and resolve them. Pharmacovigilance staff need to spend more time on data cleaning and deduplication processes, which diverts their attention from other essential activities[3].

Delays in data analysis: Sorting through duplicate entries to identify and remove them can cause delays in data analysis and signal detection. Timely identification and response to safety concerns may be hindered due to the time-consuming nature of duplicate management[3].

Regulatory compliance challenges: Duplicates can complicate regulatory reporting and compliance obligations. Thus, submitting accurate and non-duplicated data to regulatory authorities, may become more difficult and time-consuming, particularly for pharmaceutical companies[6].

For peer review only - http://bmjopen.bmj.com/site/about/guidelines.xhtml

DISCUSSION

This scoping review sought to evaluate the extent, causes, preventive strategies, detection methods, management, and impact of duplication in pharmacovigilance databases worldwide. The extent of duplication in pharmacovigilance databases varied across different databases. The WHO global database, VigiBase, exhibited a 2.5% prevalence of duplication for reports submitted from 2000 to 2010[4]. This prevalence rate, though relatively low, represents a large number of duplicates (~750,000 duplicates of the 30+ million reports in VigiBase in 2023) in a global context[81]. Duplication rates were highest for reports from published literature (11%) and fatal outcomes (5%), and were even more extreme (66% to 87%) from published literature in the United States' FAERS database. These high duplication rates could be due to the heightened awareness and documentation of certain types of adverse events, particularly serious outcomes and those of special interest[4,38]. In Europe, however, the observed prevalence of duplication was lower, ranging from 0.7% in Spain to 1.4% in the United Kingdom[4]. Thus, special attention to data quality is required when integrating pharmacovigilance datasets with varying risks of duplication. Also, regional variations call for standardized deduplication strategies to ensure consistency and data reliability across different settings.

Duplication in pharmacovigilance databases can arise from data entry errors, multiple reporters, mandatory reporting requirements, technical software glitches, and incomplete case information. This is compounded by the growing volume of pharmacovigilance reports worldwide, particularly due to the large number of adverse event reports following mass immunization with COVID-19 vaccines[82]. Thus, the possibility of duplicates cannot be ruled out, and it is crucial to develop robust and efficient systems to minimize them[5]. Understanding the causes of duplication is important to identify and institute effective measures to prevent them. Such measures could reduce the occurrence of duplication, promote coordinated approaches to deduplication, and enhance data quality and consistency. Ultimately, these strategies contribute to more reliable pharmacovigilance databases.

New adverse event reports should be screened at the time of submission to promote the early detection of duplicates in pharmacovigilance databases[6]. Approaches to duplicate detection range from manual review to automated methods such as exact matching, rule-based matching, fuzzy matching, probabilistic record linkage and artificial intelligence. Probabilistic record linkage is effective for dealing with incomplete or inconsistent data but demands computational resources[4]. Artificial intelligence models based on machine learning provide more precise and efficient means of identifying duplicates, especially in large and complex datasets[17,53]. Natural language processing techniques which use the narrative in adverse event reports have also been developed to augment duplicate detection using machine learning. These advances in natural language processing hold promise in enhancing the efficiency and precision of the deduplication of large and complex pharmacovigilance databases[64]. Automated detection systems are the recommended methods for large and complex databases[5,30,31]. However, manual review is necessary for inconclusive potential duplicates from automated systems[6,28,47]. The duplicate detection algorithms of automated systems require routine fine-tuning to adapt them to changing data requirements, ensuring database accuracy[17,53].

Software programs for the deduplication of pharmacovigilance databases significantly enhance the quality of pharmacovigilance data and ultimately improve patient safety. Dedicated software programs for duplicate management are suitable for very large databases and might not be cost-effective for small databases, particularly in LMIC[25,71-73]. Additional challenges for implementing dedicated deduplication software in LMIC include poor data quality, limited resources and infrastructure, inadequate training and capacity building, a lack of harmonization and standardization across regulatory systems, and limited access to data and information. These gaps can hinder the ability of regulatory authorities in LMIC to effectively monitor and assess drug safety and efficacy, and the impact of duplication on pharmacovigilance databases[76]. Alternative deduplication

For peer review only - http://bmjopen.bmj.com/site/about/guidelines.xhtml

software for small datasets in LMIC include the Ablebits Ultimate Suite of tools with *add-ins* for Microsoft Office Excel. Using Ablebits in addition to manual review for small datasets is cost-effective and improves the efficiency and accuracy of deduplication[74].

Duplicates have consequences for pharmacovigilance databases such as the inaccurate risk assessment which could lead to overestimation or underestimation of adverse event frequencies. This could, in turn, distort the understanding of a drug's safety profile and hinder signal detection[3-5]. Resource wastage, increased workload for pharmacovigilance staff, delays in data analysis, and regulatory compliance challenges are additional consequences of duplication[3,4,6,78-80]. These consequences underscore the importance of addressing duplication to promote the integrity and effectiveness of pharmacovigilance systems.

CONCLUSION

This scoping review provides a comprehensive overview of duplication in pharmacovigilance databases worldwide. It highlights the need for standardized practices, advanced detection methods, and collaborative efforts to minimize duplication and enhance the accuracy and reliability of drug safety data. Addressing duplication is crucial to supporting evidence-based decision-making in healthcare and drug regulation, ensuring patient safety. Software programs are commonly used for deduplication in pharmacovigilance databases and they significantly enhance the quality of pharmacovigilance data and ultimately improve patient safety. Overall, software programs offer advantages in terms of efficiency and accuracy, although there are limitations. For smaller datasets, Microsoft Office Excel with Ablebits Ultimate Suite may be cost-effective, and ought to be piloted, whereas larger and more complex datasets require dedicated software programs.

RECOMMENDATIONS

Recommendations include increased investment in pharmacovigilance infrastructure and capacity building, improved collaboration and data sharing among regulatory authorities and industry partners, and greater emphasis on the harmonization and standardization of regulatory systems worldwide. Additionally, the development and implementation of innovative technologies and approaches such as machine learning and natural language processing could help improve the efficiency and accuracy of pharmacovigilance and drug safety monitoring, particularly in LMIC. Low- and middle-income countries with small datasets could conduct pilot studies to demonstrate the feasibility of using the Ablebits Suite of tools in Microsoft Office Excel for data deduplication in their pharmacovigilance databases.

STATEMENTS AND DECLARATIONS

Funding

This study was funded by the Medical Research Council (MR/V03510X/1).

Conflicts of interest statement

Ronald Kiguba and Munir Pirmohamed obtained funding from the Medical Research Council; however, the funder had no role in drafting this manuscript. Ronald Kiguba, Gerald Isabirye, Julius Mayengo, Jonathan Owiny, Phil Tregunno, Kendal Harrison and Helen B. Ndagije declare that they have no conflicts of interest. Munir Pirmohamed has received partnership funding for the following: Medical Research Council Clinical Pharmacology Training Scheme (co-funded by Medical Research Council and Roche, UCB, Eli Lilly and Novartis). He also has unrestricted educational grant support for the UK Pharmacogenetics and Stratified Medicine Network from Bristol-Myers Squibb. He has developed an HLA genotyping panel with MC

For peer review only - http://bmjopen.bmj.com/site/about/guidelines.xhtml

Diagnostics, but does not benefit financially from this. He is part of the IMI Consortium ARDAT (www.ardat.org). None of this funding MP received is related to the current paper.

Ethical approval

This review did not involve humans as study participants therefore ethical approval was not required.

Author's contributions

RK and GI conceptualized the study, collected the data, performed data curation, conducted formal analysis and data interpretation, and developed the first draft of the manuscript. JM, JO, PT, KH, MP and HBN contributed to the study design, analysis and interpretation of the data. All authors read and approved the final draft of the manuscript.

Acknowledgements

We are grateful to the National Drug Authority - Uganda, particularly the National Pharmacovigilance Centre, which supported the situation analysis of duplication in pharmacovigilance databases worldwide.

Data Sharing Statement

All data are included in the manuscript or uploaded as supplementary material.

For peer review only

References

1. World Health Organization. The importance of pharmacovigilance. 2002. https://apps.who.int/iris/bitstream/handle/10665/42493/a75646.pdf (accessed 28 August 2023).
2. World Health Organization. Pharmacovigilance. https://www.who.int/teams/regulation-prequalification/regulation-and-safety/pharmacovigilance (accessed 13 September 2023).
3. Norén GN, Orre R, Bate A, Edwards IR. Duplicate detection in adverse drug reaction surveillance. *Data Mining and Knowledge Discovery* 2007; **14**(3): 305-28.
4. Tregunno PM, Fink DB, Fernandez-Fernandez C, Lázaro-Bengoa E, Norén GN. Performance of probabilistic method to detect duplicate individual case safety reports. *Drug safety* 2014; **37**(4): 249-58.
5. Norén GN. The Power of the Case Narrative - Can it be Brought to Bear on Duplicate Detection? *Drug safety* 2017; **40**(7): 543-6.
6. European Medicines Agency and Heads of Medicines Agencies. Guideline on good pharmacovigilance practices (GVP) Module VI Addendum I – Duplicate management of suspected adverse reaction reports. 2017. https://www.ema.europa.eu/en/documents/regulatory-procedural-guideline/guideline-good-pharmacovigilance-practices-gvp-module-vi-addendum-i-duplicate-management-suspected_en.pdf (accessed 07 August 2023).
7. European Medicines Agency. Mapping of pharmacovigilance initiatives. https://www.ema.europa.eu/en/documents/other/icmra-mapping-pharmacovigilance-initiatives_en.pdf (accessed 13 September 2023).
8. Shankar PR. Website review: The pharmacovigilance toolkit. *Journal of pharmacology & pharmacotherapeutics* 2013; **4**(4): 307-8.
9. Council for International Organizations of Medical Sciences (CIOMS). Practical Aspects of Signal Detection in Pharmacovigilance. 2010. https://cioms.ch/wp-content/uploads/2018/03/WG8-Signal-Detection.pdf (accessed 21 September 2023).
10. Council for International Organizations of Medical Sciences (CIOMS). Making medicines safer for patients everywhere. 2021. https://cioms.ch/wp-content/uploads/2021/11/CIOMS_SpecialNewsletter_UMC-9Nov2021.pdf (accessed 21 September 2023).
11. United States (US) Food and Drug Administration (FDA) Adverse Event Reporting System (FAERS). Questions and Answers on FDA's Adverse Event Reporting System (FAERS). 2018. https://www.fda.gov/drugs/surveillance/questions-and-answers-fdas-adverse-event-reporting-system-faers (accessed 09 September 2023).
12. Hauben M, Reich L, DeMicco J, Kim K. 'Extreme duplication' in the US FDA Adverse Events Reporting System database. *Drug safety* 2007; **30**(6): 551-4.
13. European Medicines Agency. EudraVigilance. 2011. https://www.ema.europa.eu/en/human-regulatory/research-development/pharmacovigilance/eudravigilance (accessed 29 August 2023).
14. Oliveira JL, Lopes P, Nunes T, et al. The EU-ADR Web Platform: delivering advanced pharmacovigilance tools. *Pharmacoepidemiol Drug Saf* 2013; **22**(5): 459-67.
15. Nwokike J, Eghan K. Pharmacovigilance in Ghana: A Systems Analysis. 2010. https://pdf.usaid.gov/pdf_docs/pnadu524.pdf (accessed 29 August 2023).
16. Asiamah M, Akuffo KO, Nortey P, Donkor N, Danso-Appiah A. Spontaneous reporting of adverse drug reaction among health professionals in Ghana. *Archives of public health = Archives belges de sante publique* 2022; **80**(1): 33.
17. Lavertu A, Vora B, Giacomini KM, Altman R, Rensi S. A New Era in Pharmacovigilance: Toward Real-World Data and Digital Monitoring. *Clinical pharmacology and therapeutics* 2021; **109**(5): 1197-202.
18. Martin-Sanchez F, Verspoor K. Big data in medicine is driving big changes. *Yearbook of medical informatics* 2014; **9**(1): 14-20.
19. ECRI Institute. Wrong-Record, Wrong-Data Errors with Health IT Systems. 2015. https://www.ecri.org/Resources/In_the_News/PSONavigator_Data_Errors_in_Health_IT_Systems.pdf (accessed 29 August 2023).
20. Ramya. Duplicate search in pharmacovigilance. 2019. https://allaboutpharmacovigilance.org/15-duplicate-search-in-pharmacovigilance/ (accessed 24 August 2023).

For peer review only - http://bmjopen.bmj.com/site/about/guidelines.xhtml

21. Pozsgai K, Szűcs G, Kőnig-Péter A, et al. Analysis of pharmacovigilance databases for spontaneous reports of adverse drug reactions related to substandard and falsified medical products: A descriptive study. 2022; **13**.
22. Harpaz R, Chase HS, Friedman C. Mining multi-item drug adverse effect associations in spontaneous reporting systems. *BMC Bioinformatics* 2010; **11**(9): S7.
23. Uppsala Monitoring Centre. Text is key for eliminating duplicate reports. 2017. https://www.uppsalareports.org/articles/text-is-key-for-eliminating-duplicate-reports/ (accessed 29 August 2023).
24. Just BH, Marc D, Munns M, Sandefer R. Why Patient Matching Is a Challenge: Research on Master Patient Index (MPI) Data Discrepancies in Key Identifying Fields. *Perspectives in health information management* 2016; **13**(Spring): 1e.
25. Uppsala Monitoring Centre (UMC). Sharing pharmacovigilance data in the WHO Programme for International Drug Monitoring https://who-umc.org/media/3236/reporting-fact-sheet.pdf (accessed 13 September 2023).
26. Amedome SN, Dadson BA. Pharmacovigilance Practices: Knowledge and Attitudes among the Healthcare Professionals at the Volta Regional Hospital of Ghana. *Journal of Pharmacovigilance* 2017; **5**(3).
27. Ghosh R, Kempf D, Pufko A, Barrios Martinez LF, Davis CM, Sethi S. Automation Opportunities in Pharmacovigilance: An Industry Survey. *Pharmaceutical medicine* 2020; **34**(1): 7-18.
28. Muñoz MA, Dal Pan GJ, Wei YJ, et al. Towards Automating Adverse Event Review: A Prediction Model for Case Report Utility. *Drug safety* 2020; **43**(4): 329-38.
29. Gandomi A, Haider M. Beyond the hype: Big data concepts, methods, and analytics. *International Journal of Information Management* 2015; **35**(2): 137-44.
30. Lewis DJ, McCallum JF. Utilizing Advanced Technologies to Augment Pharmacovigilance Systems: Challenges and Opportunities. *Therapeutic innovation & regulatory science* 2020; **54**(4): 888-99.
31. Luo Y, Thompson WK, Herr TM, et al. Natural Language Processing for EHR-Based Pharmacovigilance: A Structured Review. *Drug safety* 2017; **40**(11): 1075-89.
32. Hauben M. Artificial Intelligence and Data Mining for the Pharmacovigilance of Drug-Drug Interactions. *Clinical Therapeutics* 2023; **45**(2): 117-33.
33. Gosselt HR, Bazelmans EA, Lieber T, van Hunsel F, Härmark L. Development of a multivariate prediction model to identify individual case safety reports which require clinical review. *Pharmacoepidemiol Drug Saf* 2022; **31**(12): 1300-7.
34. Peters MD, Godfrey CM, Khalil H, McInerney P, Parker D, Soares CB. Guidance for conducting systematic scoping reviews. *International journal of evidence-based healthcare* 2015; **13**(3): 141-6.
35. Tricco AC, Lillie E, Zarin W, et al. PRISMA Extension for Scoping Reviews (PRISMA-ScR): Checklist and Explanation. *Annals of internal medicine* 2018; **169**(7): 467-73.
36. Clark JM, Sanders S, Carter M, et al. Improving the translation of search strategies using the Polyglot Search Translator: a randomized controlled trial. *Journal of the Medical Library Association : JMLA* 2020; **108**(2): 195-207.
37. Canadian Agency for Drugs and Technologies in Health. Grey matters: a practical tool for searching health-related grey literature. 2023. https://greymatters.cadth.ca/ (accessed 22 September 2023).
38. Hung E, Hauben M, Essex H, Zou C, Bright S. More extreme duplication in FDA Adverse Event Reporting System detected by literature reference normalization and fuzzy string matching. *Pharmacoepidemiol Drug Saf* 2023; **32**(3): 387-91.
39. De Las Cuevas C, Sanz EJ, Ruan CJ, de Leon J. Clozapine-associated myocarditis in the World Health Organization's pharmacovigilance database: Focus on reports from various countries. *Rev Psiquiatr Salud Ment (Engl Ed)* 2022; **15**(4): 238-50.
40. Brinker AD, Beitz J. Spontaneous reports of thrombocytopenia in association with quinine: clinical attributes and timing related to regulatory action. *Am J Hematol* 2002; **70**(4): 313-7.
41. European Medicines Agency. Guideline on good pharmacovigilance practices (GVP) Module IX Addendum I – Methodological aspects of signal detection from spontaneous reports of suspected adverse reactions. 2017. https://www.ema.europa.eu/en/documents/scientific-guideline/guideline-good-pharmacovigilance-practices-gvp-module-ix-addendum-i-methodological-aspects-signal_en.pdf (accessed 07 August 2023).
42. Banovac M, Candore G, Slattery J, et al. Patient Reporting in the EU: Analysis of EudraVigilance Data. *Drug safety* 2017; **40**(7): 629-45.

For peer review only - http://bmjopen.bmj.com/site/about/guidelines.xhtml

43. Candore G, Monzon S, Slattery J, et al. The Impact of Mandatory Reporting of Non-Serious Safety Reports to EudraVigilance on the Detection of Adverse Reactions. *Drug safety* 2022; **45**(1): 83-95.
44. Lasys T, Santa-Ana-Tellez Y, Siiskonen SJ, Groenwold RHH, Gardarsdottir H. Unintended impact of pharmacovigilance regulatory interventions: A systematic review. *Br J Clin Pharmacol* 2023.
45. Kahn MG, Callahan TJ, Barnard J, et al. A Harmonized Data Quality Assessment Terminology and Framework for the Secondary Use of Electronic Health Record Data. *EGEMS (Washington, DC)* 2016; **4**(1): 1244.
46. US Food & Drug Administration. Questions and Answers on FDA's Adverse Event Reporting System (FAERS). 2018. https://www.fda.gov/drugs/surveillance/questions-and-answers-fdas-adverse-event-reporting-system-faers (accessed 29 Augsut 2023).
47. Agency for Medicinal Products and Medical Devices of Croatia (HALMED). Strengthening Collaboration for Operating Pharmacovigilance in Europe (SCOPE). https://www.ema.europa.eu/en/documents/other/scope-training-duplicate-detection-best-practice-guide_en.pdf

(accessed 24 August 2023).
48. Richesson RL, Malloy JF, Paulus K, Cuthbertson D, Krischer JP. An automated standardized system for managing adverse events in clinical research networks. *Drug safety* 2008; **31**(10): 807-22.
49. Mockute R, Desai S, Perera S, et al. Artificial Intelligence Within Pharmacovigilance: A Means to Identify Cognitive Services and the Framework for Their Validation. *Pharmaceutical medicine* 2019; **33**(2): 109-20.
50. Bhangale R, Vaity S, Kulkarni N. A day in the life of a pharmacovigilance case processor. *Perspectives in clinical research* 2017; **8**(4): 192-5.
51. Paygude P, Devale PJIJMER. Automated data validation testing tool for data migration quality assurance. 2013; **3**(1): 599-603.
52. Postigo R, Brosch S, Slattery J, et al. EudraVigilance Medicines Safety Database: Publicly Accessible Data for Research and Public Health Protection. *Drug safety* 2018; **41**(7): 665-75.
53. Wong J, Prieto-Alhambra D, Rijnbeek PR, Desai RJ, Reps JM, Toh S. Applying Machine Learning in Distributed Data Networks for Pharmacoepidemiologic and Pharmacovigilance Studies: Opportunities, Challenges, and Considerations. *Drug safety* 2022; **45**(5): 493-510.
54. Lindquist M. Data quality management in pharmacovigilance. *Drug safety* 2004; **27**(12): 857-70.
55. Wadhwa D, Kumar K, Batra S, Sharma S. Automation in signal management in pharmacovigilance-an insight. *Briefings in bioinformatics* 2021; **22**(4).
56. Hakak S, Kamsin A, Shivakumara P, Idna Idris MY, Gilkar GA. A new split based searching for exact pattern matching for natural texts. *PLoS One* 2018; **13**(7): e0200912.
57. Christen P. Data matching: concepts and techniques for record linkage, entity resolution, and duplicate detection. Springer: Data-centric systems and applications; 2012.
58. Liu H, Gegov A, Stahl F. Categorization and Construction of Rule Based Systems 2014. https://researchportal.port.ac.uk/files/1938654/EANN2014_038.pdf (accessed 29 August 2023).
59. Wong CK, Ho SS, Saini B, Hibbs DE, Fois RA. Standardisation of the FAERS database: a systematic approach to manually recoding drug name variants. *Pharmacoepidemiol Drug Saf* 2015; **24**(7): 731-7.
60. Grigoriev I, zu Castell W, Tsvetkov P, Antonov AV. AERS spider: an online interactive tool to mine statistical associations in Adverse Event Reporting System. *Pharmacoepidemiol Drug Saf* 2014; **23**(8): 795-801.
61. Yang JD. F_MP: A fuzzy match framework for rule-based programming. *Data & Knowledge Engineering* 1997; **24**(2): 183-203.
62. Kreimeyer K, Dang O, Spiker J, et al. Increased Confidence in Deduplication of Drug Safety Reports with Natural Language Processing of Narratives at the US Food and Drug Administration. 2022; **2**.
63. Schmider J, Kumar K, LaForest C, Swankoski B, Naim K, Caubel PM. Innovation in Pharmacovigilance: Use of Artificial Intelligence in Adverse Event Case Processing. *Clinical pharmacology and therapeutics* 2019; **105**(4): 954-61.
64. Kreimeyer K, Menschik D, Winiecki S, et al. Using Probabilistic Record Linkage of Structured and Unstructured Data to Identify Duplicate Cases in Spontaneous Adverse Event Reporting Systems. *Drug safety* 2017; **40**(7): 571-82.
65. Harpaz R, DuMouchel W, Schuemie M, et al. Toward multimodal signal detection of adverse drug reactions. *Journal of biomedical informatics* 2017; **76**: 41-9.

For peer review only - http://bmjopen.bmj.com/site/about/guidelines.xhtml

66. Kreimeyer K, Dang O, Spiker J, et al. Feature engineering and machine learning for causality assessment in pharmacovigilance: Lessons learned from application to the FDA Adverse Event Reporting System. *Computers in Biology and Medicine* 2021; **135**: 104517.
67. Fusaroli M, Raschi E, Gatti M, De Ponti F, Poluzzi E. Development of a Network-Based Signal Detection Tool: The COVID-19 Adversome in the FDA Adverse Event Reporting System. *Frontiers in pharmacology* 2021; **12**: 740707.
68. Kim HR, Sung M, Park JA, et al. Analyzing adverse drug reaction using statistical and machine learning methods: A systematic review. *Medicine* 2022; **101**(25): e29387.
69. Kompa B, Hakim JB, Palepu A, et al. Artificial Intelligence Based on Machine Learning in Pharmacovigilance: A Scoping Review. *Drug safety* 2022; **45**(5): 477-91.
70. Council for International Organizations of Medical Sciences (CIOMS). Making medicines safer for patients everywhere. 2021. https://cioms.ch/wp-content/uploads/2021/11/CIOMS_SpecialNewsletter_UMC-9Nov2021.pdf (accessed 29 August 2023).
71. MedDRA. History. https://www.meddra.org/about-meddra/history (accessed 03 October 2023).
72. ArisGlobal. ArisGlobal Announces LifeSphere® MultiVigilance 10, Industry's First End-to-End Safety System With Production-Ready Automation. 2019. https://www.arisglobal.com/press-release/lifesphere-multivigilance-10-first-safety-system-production-ready-automation/ (accessed 09 September 2023).
73. Oracle. Trusted safety case management. 2021. https://www.oracle.com/a/ocom/docs/argus-trusted-safety-case-management.pdf (accessed 03 October 2023).
74. Ablebits. Ablebits Ultimate Suite. 2023. https://www.ablebits.com/ (accessed 03 October 2023).
75. Liang L, Hu J, Sun G, et al. Artificial Intelligence-Based Pharmacovigilance in the Setting of Limited Resources. *Drug safety* 2022; **45**(5): 511-9.
76. Kiguba R, Olsson S, Waitt C. Pharmacovigilance in low- and middle-income countries: A review with particular focus on Africa. *Br J Clin Pharmacol* 2023; **89**(2): 491-509.
77. He J, Baxter SL, Xu J, Xu J, Zhou X, Zhang K. The practical implementation of artificial intelligence technologies in medicine. *Nature Medicine* 2019; **25**(1): 30-6.
78. van Stekelenborg J, Kara V, Haack R, et al. Individual Case Safety Report Replication: An Analysis of Case Reporting Transmission Networks. *Drug safety* 2023; **46**(1): 39-52.
79. Rathbone J, Carter M, Hoffmann T, Glasziou P. Better duplicate detection for systematic reviewers: evaluation of Systematic Review Assistant-Deduplication Module. *Systematic reviews* 2015; **4**(1): 6.
80. Azeroual O, Jha M, Nikiforova A, Sha K, Alsmirat M, Jha S. A Record Linkage-Based Data Deduplication Framework with DataCleaner Extension. 2022; **6**(4): 27.
81. Uppsala Monitoring Centre. What is VigiBase? 2023. https://who-umc.org/vigibase/ (accessed 16 October 2023).
82. van der Boor SC, Schmitz-de Vries ETJ, Smits D, Scholl JHG, Rolfes L, van Hunsel F. Spontaneously reported adverse events following COVID-19 basic and booster immunizations in the Netherlands. *Vaccine* 2023; **41**(29): 4319-26.

For peer review only - http://bmjopen.bmj.com/site/about/guidelines.xhtml

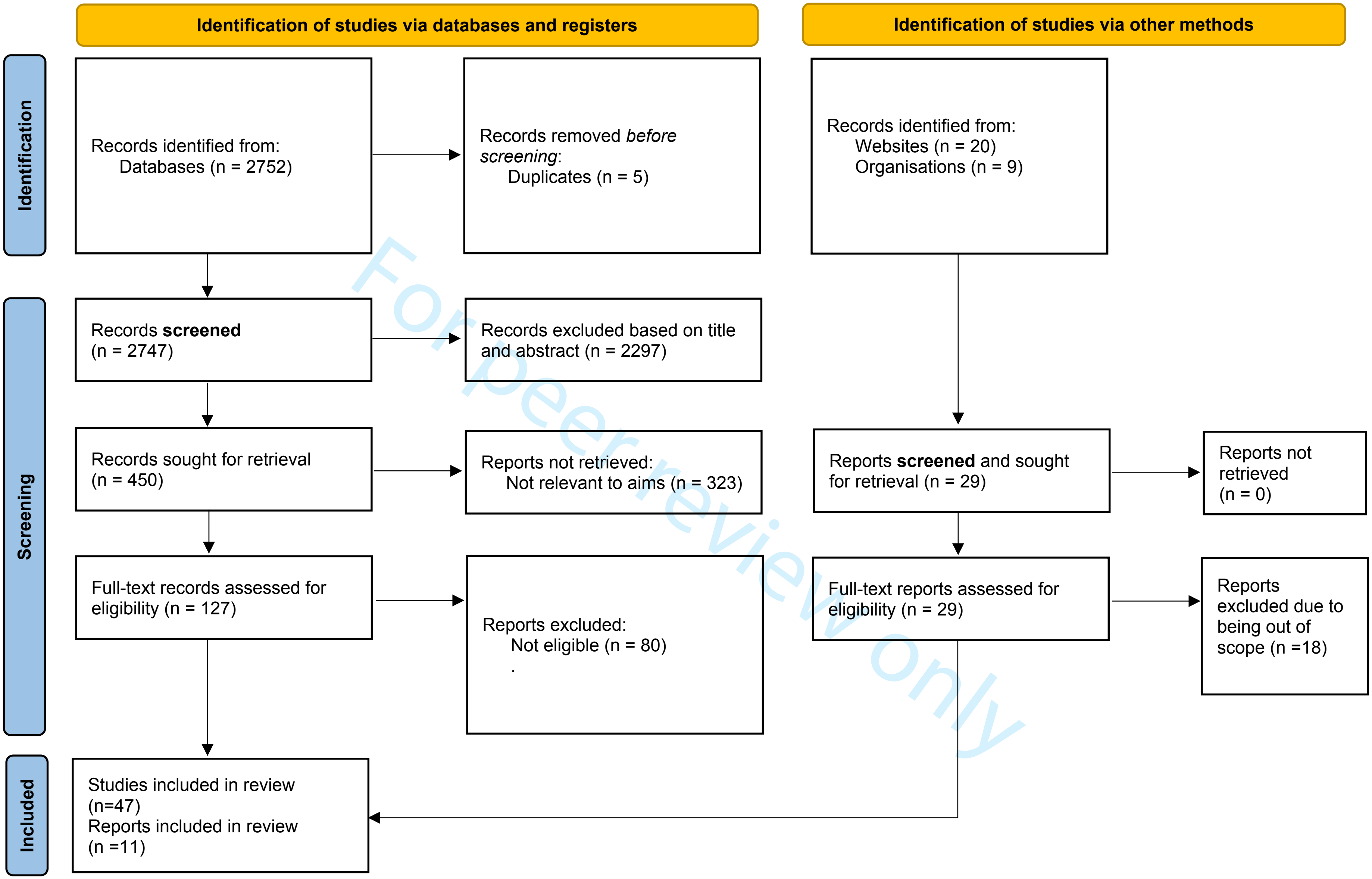

Figure 1: *PRISMA flow diagram used to select papers for the scoping review*

For peer review only - http://bmjopen.bmj.com/site/about/guidelines.xhtml

Table 1: Summary of screening process

Screening Process	Number of Papers
Initial Screening	2776
Full Text Review	156
Eligibility Criteria	
- Met Criteria?	
- Yes	58
- No	98
Specific Categories*	
- Extent of Duplicate	5
- Prevention Strategies	15
- Causes of Duplicates	13
- Methods of Duplicate Detection	32
- Management Strategies	24
- Implications of Duplicates	14

* The papers overlapped, discussing more than one field

For peer review only - http://bmjopen.bmj.com/site/about/guidelines.xhtml

Appendices

Appendix I: Search strategy in PubMed

((((((((((cause*) OR (impact*)) OR (consequence*)) OR (detection)) OR (detection tool*)) OR (prevalence)) OR (incidence)) OR (management)) OR (deduplication)) OR (prevention)) AND ((pharmacovigilance) AND (database))

Appendix II: Sources of grey literature search

Search terms use: Duplicate management in pharmacovigilance databases.

Source	Organizations	Website Links
London	European Medicines Agency	https://www.ema.europa.eu/en/documents/regulatory-procedural-guideline/guideline-good-pharmacovigilance-practices-gvp-module-vi-addendum-i-duplicate-management-suspected_en.pdf
India	https://allaboutpharmacovigilance.org/	https://allaboutpharmacovigilance.org/15-duplicate-search-in-pharmacovigilance/
Sweden	Uppsala	https://www.uppsalareports.org/articles/text-is-key-for-eliminating-duplicate-reports/
London	Medical literature monitoring	https://www.ema.europa.eu/en/human-regulatory/post-authorisation/pharmacovigilance/medical-literature-monitoring
London	Clarivate PLC	https://go.dialog.com/MLM_Duplication_Whitepaper/
London	PubHive	https://pubhive.com/pulse-literature-1/f/pubhive-automates-duplicate-detection-for-ema%E2%80%99s-mlm-service
Sweden	uppsala	https://who-umc.org/media/3236/reporting-fact-sheet.pdf
Maryland USA	FDA	https://www.accessdata.fda.gov/scripts/cderworld/index.cfm?action=drugsafety:main&unit=1&lesson=1&topic=17
New Jersey USA	flexdatabases	https://flexdatabases.com/6-faq-pv/

For peer review only - http://bmjopen.bmj.com/site/about/guidelines.xhtml

New York	citeline	https://pink.citeline.com/-/media/supporting-documents/pink-sheet/2020/02/p0220ema_9.pdf
London	GOV.UK	https://assets.publishing.service.gov.uk/government/uploads/system/uploads/attachment_data/file/949102/Exceptions_and_modifications_to_the_EU_guidance_on_good_pharmacovigilance_practices_that_apply_to_UK_MAHs_v2.pdf
San Francisco	WordPress.com	https://auditandcompliance.wordpress.com/2018/12/23/basic-steps-in-pharmacovigilance-case-processing/
Pakistan	Drug Regulatory Authority of Pakistan	https://www.dra.gov.pk/wp-content/uploads/2022/04/Good-Pharmacovigilance-Guidelines-for-Registration-Holders.pdf
Sweden	Uppsala	https://www.diva-portal.org/smash/get/diva2:1670567/FULLTEXT01.pdf
Lithuania	Biomapas	https://www.biomapas.com/selecting-a-pharmacovigilance-drug-safety-database/
Erupe	ENCePP	https://www.encepp.eu/standards_and_guidances/methodologicalGuide8_4.shtml
Brussels	AFMPS	https://www.afmps.be/sites/default/files/content/faqs_ev_changes_22-11-2017_afmps-fagg.pdf
Amsterdam	European medicines agency	https://www.adrreports.eu/en/data_quality.html#:~:text=a%20report%20is%20submitted%20by,to%20the%20report%20being%20erroneous.
Amsterdam	European medicines agency	https://www.ema.europa.eu/en/documents/other/scope-training-duplicate-detection-best-practice-guide_en.pdf
Maryland USA	FDA	https://www.fda.gov/drugs/surveillance/questions-and-answers-fdas-

For peer review only - http://bmjopen.bmj.com/site/about/guidelines.xhtml

		adverse-event-reporting-system-faers
New York	IEEE	https://ieeexplore.ieee.org/abstract/document/6732591/similar#similar

For peer review only

For peer review only - http://bmjopen.bmj.com/site/about/guidelines.xhtml

Appendix III: Data extraction instrument

Auth or(s)	Jou rnal	Ti tle	Speci fic Objec tives	Cou ntry	Stu dy des ign	Year of public ation	Ext ent	Preve ntion	Ca use s	Dete ction	Manag ement	Implic ations	Key find ings

Appendix IV: Regions and countries

Region	Number of Publications	Countries
Asia Pacific	6 publications	China<br>India<br>Malaysia<br>South Korea
Europe	28 publications	Sweden<br>Georgia<br>Switzerland<br>Netherlands<br>England<br>Italy<br>Hungary<br>Switzerland<br>Croatia<br>Germany<br>Poland
Australia	3 publications	Australia
North America	22 publications	USA

Appendix V: Articles excluded on full text review

See the attached Excel Sheet.

For peer review only - http://bmjopen.bmj.com/site/about/guidelines.xhtml

Table 2: List of 58 papers included in the scoping review

No.	Author(s)	Year of publication	Journal	Country	Title	Specific Objectives	Study design	Categories						Key findings
								Extent	Preventio	Causes	Detection	Managemen	Implication	
1	Norén GN	2017	Drug Safety	Sweden	The Power of the Case Narrative - Can it be Brought to Bear on Duplicate Detection?	In this issue of Drug Safety, Kreimeyer et al. point to the opportunity that the power of case narratives might be brought to bear also on the challenge of duplicate detection.					✓			The focus of Kreimeyer et al. is duplicate detection of more limited scope, restricted to one single drug or vaccine at a time. The examples in their paper have only in the order of 1000 reports each. As they show, automated duplicate detection may bring value in this context too. For one thing, manual identification becomes challenging even for case series of moderate size: there are more than 400 possible pairs among 30 reports, and for 100 reports, there are 5000. Also, not all duplicates are easy for human assessors to detect, as illustrated by the examples of false false positives where record pairs first assumed to have been erroneously flagged by the algorithms, on closer inspection have turned out to be overlooked true duplicates
2	Ramya.	2019		Georgia	Duplicate search in pharmacovigilance	To definne duplicates, causes of duplicates, handling of duplicates, and confirmation of duplicates and managemnt of diuplicates				✓		✓	✓	Detection and handling of duplicates by National competent Authoritties (NCAs) , Marketing Authorisation Holders MAHs and sponsors of clinical trials (sponsors) is an important element of good case management
3	Likeng Liang et al	2022	Drug Safety	China	Artifcial Intelligence-Based Pharmacovigilance in the Setting of Limited Resources	This study also discusses possible solutions and future perspectives on AI-based pharmacovigilance in resource-limited settings.	Review article					✓		Artificial intelligence (AI) algorithms can process and analyze pharmacovigilance-related data but need to be first trained with good quantities of quality data, which is the fundamental issue to be addressed. The technical challenges for AI-based pharmacovigilance in resource-limited settings are lack of high-quality databases, insufficient human resources, weak AI technology and insufficient support from governments. AI-based pharmacovigilance detection, improving training and education, and informing government of the benefits of AI-based pharmacovigilance help to solve these challenges in settings with limited resources. A collaborative research network, pharmacogenomic research and practices, and advanced machine-learning algorithms will improve AI-based pharmacovigilance in resource-limited settings in the future, but it is important to consider the particular contexts
4	Manfred Hauben et al	January 31, 2023	Clinical Therapeutics	New york	Artificial Intelligence and Data Mining for the Pharmacovigilance of Drug-Drug Interactions	Despite increasing mechanistic understanding, undetected and underrecognized drug–drug interactions (DDIs) persist. This elusiveness relates to an interwoven complexity of increasing polypharmacy, multiplex mechanistic pathways, and human biological individuality. This persistent elusiveness motivates development of artificial intelligence (AI)-based approaches to enhancing DDI detection and prediction capabilities. The literature is vast and roughly divided into "prediction" and "detection." The former relatively emphasizes biological and chemical knowledge bases, drug development, new drugs, and beneficial interactions, whereas the latter utilizes more traditional sources such as spontaneous reports, claims data, and electronic health records to detect novel adverse DDIs with authorized drugs. However, it is not a bright line, either nominally or in practice, and both are in scope for pharmacovigilance supporting signal detection but also signal refinement and evaluation, by providing data-based mechanistic arguments for/against DDI signals. The wide array of intricate and elegant methods has expanded the pharmacovigilance tool kit. How much they add to real prospective pharmacovigilance, reduce the public health impact of DDIs, and at what cost in terms of false alarms amplified by automation bias and its sequelae are open questions.	Review article					✓		Artificial Intelligence and Data Mining for the Pharmacovigilance of Drug-Drug Interactions

For peer review only

No.	Author(s)	Year of publication	Journal	Country	Title	Specific Objectives	Study design		Categories					Key findings
								Extent	Preventio	Causes	Detection	Managemen	Implication	
5	Wadhwa et al.	2021	Oxford Academic	India	Automation in signal management in pharmacovigilance—an insight	The process of signal management comprises of techniques to identify individual case safety reports systematically. Automated signal detection is highly based upon the data mining of the spontaneous reporting system such as reports from health care professional, observational studies, medical literature or from social media. If a signal is not managed properly, it can become an identical risk associated with the drug which can be hazardous for the patient safety and may have fatal outcomes which may impact health care system adversely. Once a signal is detected quantitatively, it can be further processed by the signal management team for the qualitative analysis and further evaluations. The main components of automated signal detection are data extraction, data acquisition, data selection, and data analysis and data evaluation. This system must be developed in the correct format and context, which eventually emphasizes the quality of data collected and leads to the optimal decision-making based upon the scientific evaluation.	Review article				✓			Automated systems consist of automated databases which store ICSRs for the electronic submission of data to the regulatory authorities, and in addition to it, various data mining algorithms can be used by different regulatory authorities in order to generate or detect signals optimistically. All the automated systems are primarily validated to avoid possible errors which can lead to inaccurate information or false signals in future. Pharmacovigilance experts can now focus on the quality triage and on prioritizing the signals instead of manually reviewing the reports and it can only be possible due to the automated systems. Recently, pharmacovigilance industry has been developed expeditedly due to the automated systems used by maximum of multinational companies such as Novartis
6	Adam Lavertu et al	2021	CLINICAL PHARMACOLOGY & THERAPEUTICS| VOLUME 109 NUMBER 5 | May 2021	California	A New Era in Pharmacovigilance: Toward Real- World Data and Digital Monitoring	Several different approaches to modern pharmacovigilance, including spontaneous reporting databases, electronic health record monitoring and research frameworks, social media surveillance, and the use of digital devices. Some of these platforms are well-established while others are still emerging or experimental. We highlight both the potential opportunity, as well as the existing challenges within these pharmacovigilance systems that have already begun to impact the drug development process, as well as the landscape of postmarket drug safety monitoring. Further research and investment into different and complementary pharmacovigilance systems is needed to ensure the continued safety of pharmacotherapy	Mini review				✓			Clearly, the development of these massive sources of data for future pharmacovigilance efforts creates an opportunity for capitalizing on recent advances in deep learning and anomaly detection. A con-tinuously learning artificial intelligence system could not only learn to integrate these heterogeneous data sources for real-time ADR detection, but could help identify potential cases and interface with members of the pharmacotherapy community to gather more information when needed. The field of pharmacovigilance is rap-idly evolving, however, the resources we have highlighted are only part of the solution; the FDA and National Institutes of Health (NIH) will need to continue their funding of research that focuses on how to effectively analyze these data streams. Ideally, funding
7	Rodrigo Postigo	2018	Drug Safety	Drug Safety	EudraVigilance Medicines Safety Database: Publicly Accessible Data for Research and Public Health Protection	The main objective of this article is to describe the new access to the database together with the opportunities that this new access can bring for research. It is intended to promote an appropriate use of the data to support the safe and effective use of medicines	SPECIAL ARTICLE		✓					Pharmacovigilance is an essential discipline for the promotion and protection of patient and public health. In the EEA, the EU regulatory network formed by the European Commission, the NCAs and the EMA has a key role together with the MAHs on the safety surveillance on medicinal products. The EU legislation places the EV database in a core role in the management of reports of suspected adverse reactions and in determining changes in the safety profile of the medicinal products once they are used in everyday medical practice. For over 16 years, EV has demonstrated its value especially in the area of signal detection, providing a tool to analyse the safety data submitted. Although EV has been fully accessible to the EMA and the NCAs in the countries that belong to the EEA, the access provided in the past to other stakeholders has been more limited. The new extended EV access provides a unique platform for all stakeholders to use the data according to their needs, interest and pharmacovigilance obligations. This enhanced access brings opportunities for a broader involvement in safety monitoring, for increased transparency, for collective collaborations, for methods improvement and for innovation in safety

For peer review only

No.	Author(s)	Year of publication	Journal	Country	Title	Specific Objectives	Study design	Categories						Key findings
								Extent	Preventio	Causes	Detection	Managemen	Implication	
8	Jenna Wong et al	2022	Drug Safety	Switzerland	Applying Machine Learning in Distributed Data Networks for Pharmacoepidemiologic and Pharmacovigilance Studies: Opportunities, Challenges, and Considerations	In this paper, we discuss opportunities, challenges, and considerations for applying ML in DDNs for pharmacoepidemiologic and pharmacovigilance studies. We frst discuss major types of activities performed by DDNs and how ML may be used. Next, we discuss practical data-related factors infuencing how DDNs work in practice. We then combine these discussions and jointly consider how opportunities for ML are afected by practical data-related factors for DDNs, leading to several challenges. We present diferent approaches for addressing these challenges and highlight eforts that real-world DDNs have taken or are currently taking to help mitigate them. Despite these challenges, the time is ripe for the emerging interest to use ML in DDNs, and the utility of these data-adaptive modeling techniques in pharmacoepidemiologic and pharmacovigilance studies will likely continue to increase in the coming years	Review article		✔		✔			In conclusion, there is great potential, and great desire, to use ML in DDNs to enhance their activities in pharmacoepidemiology and pharmacovigilance. Indeed, the time is ripe for this emerging area of interest among DDNs because of not only the recent methodologic advancements in the feld, but also the valuable groundwork that many DDNs have already laid through investments in harmonizing datasets to a CDM, developing standardized processes, tools, and analytics, and building collaborative relationships between data partners and with various stakeholders. The future holds much promise for the use of ML in DDNs, and we expect that the utility of these data-adaptive methods for enhancing pharmacoepidemiologic and pharmacovigilance studies will likely continue to increase in the years to come
9	Philip Michael Tregunno et al	2014	Drug Safety	London	Performance of Probabilistic Method to Detect Duplicate Individual Case Safety Reports	The aim of this study was to evaluate probabilistic record matching for duplicate detection, and to characterise the main sources of duplicate reports within each data set.	Original Research Articl	✔	✔	✔	✔	✔	✔	Probabilistic record matching, as implemented in vigiMatch, achieved good predictive value for confirmed or likely duplicates in each data source. Most of the false positives corresponded to otherwise related reports; less than 10 % were altogether unrelated. A substantial proportion of the correctly identified duplicates had not previously been detected by national centre activity. On one hand, vigiMatch highlighted duplicates that had been missed by rule-based methods, and on the other hand its lower total number of suspected duplicates to review improved the accuracy of manual review
10	Benjamin Kompa et al	2022	Drug Safety		Artifcial Intelligence Based on Machine Learning in Pharmacovigilance: A Scoping Review	The present study conducted a scoping review of the use of artificial intelligence based on machine learning to understand how it is used for pharmacovigilance tasks, characterize differences with other fields, and identify opportunities to improve pharmacovigilance through the use of machine learning.	Scoping Review				✔	✔		We have conducted a scoping review of the use of ML for PV applications. Our aim was to assess the extent to which PV has been or is ready to be improved by current deep learning-based AI techniques. We found that while certain modern practices have begun to appear, many of the primary reasons for the recent success of AI have yet to be translated. We conclude that without certain structural changes, PV is unlikely to experience similar kinds of advancements from current approaches to AI
11	Marin Banovac et al	2017	Drug Safety		Patient Reporting in the EU: Analysis of EudraVigilance Data	This descriptive study aims to provide insight into patient reporting for the totality of the EU by querying the EudraVigilance (EV) database for the period of 3 years before the new pharmacovigilance legislation became operational and the 3 years after as well as comparing patient reports with those from healthcare professionals (HCPs) where feasible.	Descriptive study			✔			1	Patient reporting complemented reporting by HCPs. Patients were motivated to report ADRs, especially those that affected their quality of life. Sharing these results with NCAs and patient associations can inform training and awareness on patient reporting.
12	Gianmario Candore et al	2022	Drug Safety	Netherlands	The Impact of Mandatory Reporting of Non-Serious Safety Reports to EudraVigilance on the Detection of Adverse Reactions	European Union legislation has mandated the submission of European Economic Area non-serious reports to the EudraVigilance database since November 2017. As spontaneous reports of suspected adverse reactions to medicines represent a key source of safety signals, the European Medicines Agency has undertaken this work to assess the effects of this requirement on the characteristics of the reports submitted to EudraVigilance and on the detection of adverse drug reactions through routine analyses of the database.	Original Research Articl			✔				The change in legislation has led to a small increase in sensitivity, without affecting the efficiency of the routine statistical measures used. The number of non-serious reports as a proportion of reports in EudraVigilance is likely to increase over time and further monitoring of the impact on signal detection is required. Further work is also required on the qualitative impact of non-serious reports on the nature of signals detected and on their evaluation.

For peer review only

No.	Author(s)	Year of publication	Journal	Country	Title	Specific Objectives	Study design	Extent	Preventio	Causes	Detection	Managemen	Implication	Key findings
									Categories					
13	John van Stekelenborg,	2022	Drug Safety		Individual Case Safety Report Replication: An Analysis of Case Reporting Transmission Networks	The present study aimed to investigate the magnitude of replication, the variability among recipients, and the subsequent divergence across recipients of ICSRs.	Original Research Articl						✔	Replication of ICSRs and the variation of available safety information in recipient databases were quantified and shown to be substantial. Our work shows that multiple processors and medical reviewers will likely handle the same original ICSR as a result of replication. Aside from the obvious duplicate work, this phenomenon could conceivably lead to differing clinical assessments and decisions. If replication could be reduced or even eliminated, this would enable more focus on activities with a benefit for patient safety.
14	Rachel L Richesson et al	2008	Drug Safety	Florida	An Automated Standardized System for Managing Adverse Events in Clinical Research Networks	We describe an Adverse Event Data Management System (AEDAMS) that is used across multiple study designs in the various clinical research networks and multi-site studies for which we provide data and technological support. Investigators enter AE data using a standardized and structured web-based data collection form	Author manuscript		✔			✔		The automated nature of the AEDAMS ensures that AEs are quickly reported to multiple parties responsible for participant safety, including investigators and DSMBs. In clinical research networks and multi-site protocols, a standard approach to AE reporting and management allows AEs to be monitored and examined at a network or organizational level, which might increase the understanding of the safety of agents used across protocols. An automated approach impacts the speed and efficiency of the system and is scalable. Efficient, scalable and flexible systems for AE monitoring can impact patient safety and streamline the clinical research process to enable faster application of investigational treatments to the community. Future research should include formal evaluation of this and other AE management systems, quantitatively measuring their benefits and performance in terms efficiency, reliability, accuracy and any reduction of patient morbidity and mortality resulting from enhanced communication and decision making.
15	Michele Fusaroli et al	2021	Frontiers in Pharmacology	Bologna	Development of a Network-Based Signal Detection Tool: The COVID-19 Adversome in the FDA Adverse Event Reporting System	The analysis of pharmacovigilance databases is crucial for the safety profiling of new and repurposed drugs, especially in the COVID-19 era. Traditional pharmacovigilance analyses–based on disproportionality approaches–cannot usually account for the complexity of spontaneous reports often with multiple concomitant drugs and events. We propose a network-based approach on co-reported events to help assessing disproportionalities and to effectively and timely identify disease-, comorbidity- and drug-related syndromes, especially in a rapidly changing low-resources environment such as that of COVID-19.	BRIEF RESEARCH REPORT article				✔			New drugs, for which safety was tested only as a secondary outcome in small numbers and ideal conditions-clinical trials, are introduced continuously in the real world, and require a strict monitoring by pharmacovigilance. COVID-19 pandemic, with its rapidly changing environment and limited resources, and with the repurposing of already marketed agents, has shown an even stronger need for pharmacovigilance and its signal detection.<br><br>However, current methods (e.g., single disproportionality analyses) struggle with two issues: 1) the richness and complexity of cases, often containing multiple drugs and adverse events; and 2) the presence of multiple important biases confounding the inference of associations as reaction.<br><br>In order to deal with these issues we developed the Adversome, a new approach that complements traditional signal detection. The Adversome explicitly takes into account the co-reporting of multiple suspected Adverse Drug Reactions (ADRs) and helps to include this information in the clinical assessment of statistical signals, as a

For peer review only

No.	Author(s)	Year of publication	Journal	Country	Title	Specific Objectives	Study design		Categories					Key findings
								Extent	Preventio	Causes	Detection	Managemen	Implication	
16	Rave Harpaz	2010	BMC Bioinformatics	New York	Mining multi-item drug adverse effect associations in spontaneous reporting systems	Multi-item adverse drug event (ADE) associations are associations relating multiple drugs to possibly multiple adverse events. The current standard in pharmacovigilance is bivariate association analysis, where each single drug-adverse effect combination is studied separately. The importance and difficulty in the detection of multi-item ADE associations was noted in several prominent pharmacovigilance studies. In this paper we examine the application of a well established data mining method known as association rule mining, which we tailored to the above problem, and demonstrate its value. The method was applied to the FDAs spontaneous adverse event reporting system (AERS) with minimal restrictions and expectations on its output, an experiment that has not been previously done on the scale and generality proposed in this work	Research Article		✓		✓	✓	✓	Our findings demonstrate that multi-item ADEs are present and can be extracted from the FDA's adverse effect reporting system using our methodology, suggesting that our method is a valid approach for the initial identification of multi-item ADEs. The study also revealed several limitations and challenges that can be attributed to both the method and quality of data.
17	F Martin-Sanchez et al	2014		Australia	Big data in medicine is driving big changes	To summarise current research that takes advantage of "Big Data" in health and biomedical informatics applications.	Review article		✓	✓	✓	✓	✓	The survey highlights ongoing development of powerful new methods for turning that large-scale, and often complex, data into information that provides new insights into human health, in a range of different areas. Consideration of this body of work identifies several important paradigm shifts that are facilitated by Big Data resources and methods: in clinical and translational research, from hypothesis-driven research to data-driven research, and in medicine, from evidence-based practice to practice-based evidence.The increasing scale and availability of large quantities of health data require strategies for data management, data linkage, and data integration beyond the limits of many existing information systems, and substantial effort is underway to meet those needs. As our ability to make sense of that data improves, the value of the data will continue to increase. Health systems, genetics and genomics, population and public health; all areas of biomedicine stand to benefit from Big Data and the associated technologies.
18	Marie Lindquist et al	2004	Drug Safety	Sweden	Data Quality Management in Pharmacovigilance	Pharmacovigilance relies on information gathered from the collection of indi vidual case safety reports and other pharmacoepidemiological data. Even gi ven the inherent limitations of spontaneous reports, the usefulness of this dat a source can be improved with good data quality management. Although un der-re┐porting cannot be remedied this way, the negative impact of incompl ete reports, which is another serious problem in pharmacovigilance, can be r educed. Quality management consists of quality planning, quality control, q uality assurance and quality improvements. The pharmacovigilance data pro cessing cycle starts with data collection and, in computerised systems, data e ntry; the next step is data storage and maintenance; followed by data selectio n, retrieval and manipulation. The resulting data output is analysed and asses sed. Finally, conclusions are drawn and decisions made. The increased know ledge feeds back into the data processing cycle. Focussing on the first three steps of the data processing cycle, the different quality dimensions associate d with these steps are described in this review, together with examples relev ant to pharmacovigilance data.	leading article				✓			There are a number of data quality dimensions involving data processing and its results. The maintenance of good quality must be upheld throughout the processing cycle, and in the computerized systems employed as supporting platforms. Although a truism, the statement 'no chain is stronger than its weakest link' is clearly valid. A problem at one level may not only be perpetuated but exacerbated at a later stage. To further improve the situation, the implementation of data quality management practices in pharmacovigilance needs to be put high on the agenda. The aim should be to have transparent, well-documented, and functioning quality systems in place. In this instance, it should be said that the author, regrettably, is fully aware that the system she is most familiar with does not yet live up to the standards put forward in this paper.<br><br>Although most countries have regulatory requirements in place determining who can or must report, what should be reported, and when, the quality management of the data actually received is to a large extent left to those in charge of the database systems where this information is normally stored. Even if database systems are of

For peer review only

No.	Author(s)	Year of publication	Journal	Country	Title	Specific Objectives	Study design	Categories							Key findings
								Extent	Preventio	Causes	Detection	Managemen	Implication		
19	Monica A. Muñ oz et al	2020	Drug Safety	Kentucky	Towards Automating Adverse Event Review: A Prediction Model for Case Report Utility	The rapidly expanding size of the Food and Drug Administration's (FDA) Adverse Event Reporting System database requires modernized pharmacovigilance practices. Techniques to systematically identify high utility individual case safety reports (ICSRs) will support safety signal management. Objectives The aim of this study was to develop and validate a model predictive of an ICSR's pharmacovigilance utility (PVU). Techniques to systematically identify and distinguish higher utility individual case safety reports (ICSRs) from lower utility ones will improve timeliness in managing safety signals.<br>We developed a predictive model to prioritize ICSRs for manual review that performed better than using data field completeness alone.<br>The model's modest performance demonstrated the feasibility of this approach but also highlighted opportunities for further refinement such as extracting additional predictors from unstructured data and developing algorithms for particular safety issues					✓				Our model demonstrated the feasibility of developi ng a tool predictive of ICSR utility. The model's m odest discriminative ability highlights opportunitie s for further enhancement and suggests algorithms tailored to safety issues may be benefcial Our stu dy demonstrated the feasibility of developing predi c┐tive tools to augment review of ICSRs from FA ERS. The<br>model's modest discriminative ability highlights o pportu┐nities for further enhancement and suggests algorithms tai┐lored to safety issues may be benef
20	José Luis Oliveira	2013	Drug Safety		The EU-ADR Web Platform: delivering advanced pharmacovigilance tools	Pharmacovigilance methods have advanced greatly during the last decades, making post-market drug assessment an essential drug evaluation component. These methods mainly rely on the use of spontaneous reporting systems and health information databases to collect expertise from huge amounts of real-world reports. The EU-ADR Web Platform was built to further facilitate accessing, monitoring and exploring these data, enabling an in-depth analysis of adverse drug reactions risks.			✓		✓	✓			The EU-ADR European project embraces innovative pharmacovigilance research methodologies through the creation of a web platform providing advanced drug data exploration and assessment features. Whereas, in the past, post-marketing drug assessment required intense validation tasks, the in silico pharmacology community is now endowed with the tools required to quickly analyse specific ADRs, further improving drug safety monitoring. The EU-ADR Web Platform enables streamlined access to drug dataset analysis features, including the evaluation of results from EU-ADR workflows and the sharing of data amongst research partners, all within a highly responsive and unique web-based workspace, which is available at http://bioinformatics.ua.pt/euadr
21	Helen R. Gosselt et al.	2022	wiley	Netherlan ds	Development of a multivariate prediction model to identify individual case safety reports which require clinical review	Objectives: To develop a prediction model to identify ICSRs that require cli nical review, including PSTRs.. Secondly, to identify the most important fea tures of these reports. ey Points<br>• The number of individual case safety reports in pharmacovigilance databas es are increasing.<br>• New approaches are required to prioritise individual case reports based on their potential sig┐nal triggering value since case-by-case review is very tim e consuming.<br>• We developed a prediction model using structured data of individual case s afety reports to<br>identify potential signal triggering reports in the Netherlands.<br>• We identified nine most important predictors that were used to identify pot ential signal trig┐gering reports.<br>• The prediction model could be further optimised using additional predictor s such as text<br>fields or other unstructured data from case reports.	Original Research Articl						✓		This study showed promising results, with recall a nd precision scores above 0.80,38 which gives reas on for Lareb to further investigate the application o f this technique in future studies. Furthermore, whe n assessing an individual ICSR our spontaneous re port system is coupled to a tool in which is analyze d how many reports have been reported previously on the same drug-ADR combi┐nations, accompani ed by a disproportionality score, which could also play role in determining whether a report is a PST R. Moreover, it may be interesting to do a sub-anal ysis on different drug groups since dif┐ferent featu res of the ICSR might be important for different ty pes of medicines. Lastly, it would be interesting to do an analysis of the 5% of ICSRs that were correc tly classified as not being a PSTR. If a significant proportion of those ICSR have been assessed by a sci┐entific reviewer, implementing the model can s ave time and resources.

For peer review only - http://bmjopen.bmj.com/site/about/guidelines.xhtml

No.	Author(s)	Year of publicatio n	Journal	Country	Title	Specific Objectives	Study design	Extent	Preventio	Causes	Detection	Managemen	Implication	Key findings
								Categories						
22	Tomas Lasys et al	2023	BJCP		Unintended impact of pharmacovigilance regulatory interventions: A systematic review	Studies assessing the impact of pharmacovigilance regulatory interventions often focus on the expected (or intended) outcomes, while any possible unintended impact may be overlooked. The update of the Good Pharmacovigilance Practice guideline in 2017 elaborated on impact assessment, emphasizing the need also to assess possible unintended impact. This systematic literature review investigated how often the unintended impact of regulatory interventions was considered in publications of studies investigating pharmacovigilance regulatory interventions in Europe.	Review article			✔				While regulatory interventions issued by EMA are mandatory for all Member States, most regulatory interventions are implemented by national authorities, which can lead to differences in outcomes between the Member States. Different impact assessments between countries might affect the generalizability of results which could be even more aggravated because some studies only use data from a single country or even from parts of countries. Among included publications, multinational studies examining the impact of pharmacovigilance regulatory interventions for diclofenac and hydroxyzine initiation and discontinuation reported that the possible impact of interventions differed between countries based on trend and step changes.28, 29 This could be due to different implementation strategies used by competent authorities and different levels of drug use in the preintervention periods. In addition to the possible differences between national implementation of pharmacovigilance regulatory interventions, not all countries are equally involved in conducting impact assessments. Even though the regulatory interventions are mandatory
23	Ruta Mockute et al	2017	Drug Safety	USA	Artificial Intelligence Within Pharmacovigilance: A Means to Identify Cognitive Services	The aim of this study was to identify areas across the PV value chain that can be augmented by cognitive service solutions using the methodologies of contextual analysis and cognitive load theory. It will also provide a framework of how to validate these PV cognitive services leveraging the acceptable quality limit approach.	Review		✔					As individual case safety report volumes increase, artificial intelligence can be a means to help mitigate complex decision making for pharmacovigilance professionals. At various decision points in the PV process, cognitive
24	Jianxing He et al	2019	Nat Med	china	The practical implementati	The development of artificial intelligence (AI)-based technologies in medicine is advancing rapidly, but real-world clinical implementation has not yet become a reality. Here we review some of the key practical issues surrounding the implementation of AI into existing clinical workflows, including data sharing and privacy, transparency of algorithms, data standardization, and interoperability across multiple platforms, and concern for patient safety. We summarize the current regulatory environment in the United States and highlight comparisons with other regions in the world, notably Europe and China.	Review article					✔		A I has recently experienced an era of explosive growth across many industries, and the healthcare industry is no exception. Studies across multiple medical specialties have employed AI to mimic the diagnostic abilities of physicians1–4. The hope is that AI may augment the ability of humans to provide healthcare. However, although these technologies are rapidly advancing, their implementation into patient-care settings has not yet become widespread. Here we review some key issues surrounding implementation of AI-based technologies in healthcare.
25	Juergen Schmide	2018	Clin Pharma col Ther		Innovation in Pharmacovig	Is it viable to use advanced AI tools in the application for AE case processing, specifically the extraction of case critical information from source documents to identify valid AE cases after training the machine-learning algorithms with source documents and database content rather than annotated source documents?					✔			In conclusion, the pilot was successful in confirming the feasibility of using AI-based tools to support PV operations and in demonstrating the viability of an efficient training method that does not require time-consuming and costly annotations. Finally, the evaluation and scoring method used in the pilot was able to differentiate vendor capabilities and identify vendor 1 as the best candidate to move into the discovery phase.

For peer review only

No.	Author(s)	Year of publication	Journal	Country	Title	Specific Objectives	Study design	Categories						Key findings
								Extent	Preventio	Causes	Detection	Managemen	Implication	
26	David John Lewi	2019	PMC	Germany	Utilizing Advanced Techno	The overall goal of automation is to provide high quality safety data in the correct format, in context, more quickly, and with less manual effort. This will improve the evidence available for scientific assessment and helps to inform and expedite decisions about the minimization of risks associated with medicines.	Rreview article		✔		✔	✔		We recognize that the rapid development of new technologies will invariably outpace the regulations applicable to PV systems. Nevertheless, we believe that PV systems may be improved by intelligent automation. It is incumbent on the owners of PV systems to oversee applicability, design, deployment, performance, validation, and maintenance of advanced technologies to ensure that the PV system continues to be ft for purpose. It is equally, if not more, important that intelligent automated PV systems should be trusted. Our aim is to provide the right type of safety data in the right format and context, thereby increasing the quality of the evidence available for scientifc evaluation to inform decisions optimally. Ultimately, our views concerning PV systems are similar to those expressed by other authors in the feld of biomedical sciences [53–55]. We believe that PV system owners will have to adapt to artifcial intelligence by the progressive, intelligent adoption of new technologies, commencing with process automation. It has already been established that algorithmic processing can outperform humans when performing binary, repetitive tasks [56]. T
27		2017	European	Europe	Guideline on good pharma	Section VI.B. of this Module highlights the general principles, based on the pharmacovigilance guidelines E2A, E2B and E2D of the International Council for Harmonisation of Technical Requirements for Pharmaceuticals for Human Use (ICH) (see GVP Annex IV), in relation to the collection, recording and submission of individual reports of suspected adverse reactions associated with medicinal products for human use. The definitions and guidance provided in Section VI.A. and the EU specific requirements presented in Section VI.C. should be followed. All applicable legal requirements are referenced in the way explained in the GVP Introductory Cover Note and are usually identifiable by the modal verb "shall". Guidance for the implementation of legal requirements is provided using the modal verb "should". The guidance provided in this Module does not address th	Guidelines		✔		✔	✔		Duplicate detection in EudraVigilance – Collaboration between the Agency, competent authorities in Member States and marketing authorisation holders - Duplicate ICSRs submitted to EudraVigilance by the same sender and identified by the Agency
28					ArisGlobal Announces Life	LifeSphere® MultiVigilance 10 features all new architecture and design, incorporating the latest in cognitive automation technology to deliver groundbreaking efficiency gains to PV teams	Website					✔		LifeSphere® MultiVigilance 10 (LSMV10), the industry's only end-to-end drug safety system with production-ready automation. LSMV10 has been developed and tested in partnership with leading global pharmaceutical companies and health authorities, resulting in a solution that provides immediate efficiency gains and out-of-the-box compliance with all global submission requirements. Under tightening budgets and evolving global regulations, safety case volumes continue to grow by nearly 15% each year, creating challenges for today's drug safety teams. To address these pressures, LSMV10 leverages cognitive computing technologies such as natural language processing and machine learning, providing a single, global database that supports end-to-end automation of all adverse event processing. In addition, LSMV10 is part of ArisGlobal's unified LifeSphere® Platform and is the first and only safety system to offer out-of-the-box integration with medical information and product complaint solutions, enabling the seamless exchange of safety data between these critical functions

For peer review only

No.	Author(s)	Year of publication	Journal	Country	Title	Specific Objectives	Study design	Categories						Key findings
								Extent	Preventio	Causes	Detection	Managemen	Implication	
29	Rajesh Ghosh et	2020	SpringerI	USA	Automation Opportunities	TransCelerate's IAO in Pharmacovigilance initiative initially worked to articulate an end-to-end ICSR process that would generically apply to various pharmacovigilance organizations, despite organizational variations in specific ICSR process steps. This paper aims to address the need for a systematic review framework for automation of the ICSR process from the value, impact, perceived risk, and opportunity point of view.	ORIGINAL RESEARCH ARTICLE					✓		In total, 15 TransCelerate member companies responded to the survey and indicated that ICSR process steps with current high effort, expected high automation benefit, low or manageable automation risk, and low levels of current automation present the best opportunities for future automation. Such steps include language translations, case verification, in-line quality control, prioritization/triage, data entry, alerts for cases of interest, workflow management, and monitoring. Some steps (e.g., submission) have been automated for a number of years and appear on the heat map as having low potential for further automation. The survey responses implied that, despite successful use of intelligent automation technologies in other areas, adoption within pharmacovigilance and the ICSR process in particular remains limited. The perceived high risk to patient safety is expected to decrease with additional successful applications in pharmacovigilance
30	Michael G Kahn	2016	EDM For	Colorado	A Harmonized Data Qualit	Harmonized data quality (DQ) assessment terms, methods, and reporting practices can establish a common understanding of the strengths and limitations of electronic health record (EHR) data for operational analytics, quality improvement, and research. Existing published DQ terms were harmonized to a comprehensive unified terminology with definitions and examples and organized into a conceptual framework to support a common approach to defining whether EHR data is 'fit' for specific uses.			✓	✓				A consistent, common DQ terminology, organized into a logical framework, is an initial step in enabling data owners and users, patients, and policy makers to evaluate and communicate data quality findings in a well-defined manner with a shared vocabulary. Future work will leverage the framework and terminology to develop reusable data quality assessment and reporting methods.
31	Tomas Bergvall e	2013	Drug Saf	Sweden	vigiGrade: A Tool to Ident	The objective of this study was to propose a measure of completeness and identify predictors of well-documented reports, globally.				✓				vigiGrade distinguishes different aspects of quality according to the outline in Lindquist [10]. An important advantage compared with the earlier implementation of documentation grading in VigiBase is that the vigiGrade completeness score considers each dimension in parallel, instead of in sequence: even when information on time-to-onset is lacking, the other dimensions are evaluated and accounted for in the total completeness score. vigiGrade considers many of the same fields as does the structured assessment proposed by Agbabiaka et al. [13], but is less comprehensive. Specifically, it does not evaluate dimensions for which absence of information cannot be distinguished from information on absence in VigiBase. On the other hand, it is a scalable solution that allows automated database-wide analyses. By design, it allows for significant penalties of a variety of missing dimensions

For peer review only

No.	Author(s)	Year of publication	Journal	Country	Title	Specific Objectives	Study design	Categories						Key findings
								Extent	Preventio	Causes	Detection	Managemen	Implication	
32	M. Hauben	2007	Drug Safety		Extreme Duplication' in the US FDA Adverse Events Reporting System Database	We use the term 'extreme duplication' because the majority of reports of the drug-event pair under study were found to be dupli cate reports, and these duplicates resulted in the occurrence of a large signal of disproportionate re porting (SDR)[1] discovered during a data-mining exercise. This occurred during a recent demonstra tion within our company of a commercial vendor's data-mining software containing these data. Data mining includes emerging computer-based quantita tive tools for screening spontaneous reporting sys tem (SRS) databases to assist in the identification of potential new drug safety issues. Although duplicate reporting is one of the numerous well recognised forms of data corruption and distortion in SRS databases, the finding surprised us nonetheless, giv en the duplicate detection algorithms and procedures included in the commercial data-mining software we used.					✓	✓	✓	Extreme duplication was discovered within the US FDA Adverse Events Reporting System (AERS) Database during a data analysis conducted by a commercial vendor. This duplication problem in regulatory and company databases can arise from various sources and a failure to connect related cases. Vendors do make efforts to eliminate duplicate cases before providing data to customers, but sometimes the criteria used may not be sufficient for accurate identification and removal of duplicates. The impact of extreme duplication in AERS, compared to the World Health Organization (WHO) database, is evident, as it affected data-mining results in AERS but not in WHO. The document also suggests that duplicate reports in spontaneous reporting systems may carry unique information value and deserve further investigation. Acknowledging the limitations and challenges of spontaneous reporting system (SRS) data, including inconsistencies, errors, duplications, and incomplete data in critical fields. It's crucial to approach any findings based on SRS data with an appropriate level of caution, taking into account these limitations and the potential impact of duplication on data analysis
33	CIOMS	2010		Switzerland	Practical Aspects of Signal Detection in Pharmacovigilance	The objective of the CIOMS VIII report is to provide useful points for con sideration to manufacturers, regulatory authorities, international monitoring centers and others wishing to establish or understand the output of a systematic and holistic strategy to better manage the entire "lifecycle" of a drug safety signal. This lifecycle includes signal detection, signal prioritization, and signal evaluation. If the evalua tion of a drug safety signal establishes a new adverse drug reaction, then this stage of the signal's lifecycle will lead to an update of the product's prescribing information and, possibly, other regulatory actions including further risk communications and risk minimization efforts.				✓			✓	Many large SRS databases contain duplicate reports, i.e. reports from different sources on the same adverse event in the same patient. For example, duplicate reports are present in AERS, Vigibase, and EudraVigilance. The identifi cation and elimina tion of duplicates from analyses will, therefore, be advantageous for any signal evalu ation. However, current duplicate detection procedures, some of which are applied prospectively (i.e. prior to data mining) and others retrospectively (i.e. after data min ing), have limitations (see ref 10) and enhanced methods of duplicate detection are being developed (11). Data quality and
34	Kevin Pozsgai et al	2022	National Library of Medicine	Hungary.	Analysis of pharmacovigilance databases for spontaneous reports of adverse drug reactions related to substandard and falsified medical products: A descriptive study	Our aim was to identify and describe the characteristics of cases that are related to adverse drug reactions potentially originating from counterfeit medication using publicly available pharmacovigilance data.				✓				he application of pharmacovigilance datasets to identify potential counterfeit medicine ADRs can be a valuable tool in recognition of potential risk groups of consumers and the affected active pharmaceutical ingredients and products. However, the further development and standardization of ADR reporting, pharmacovigilance database analysis, and prospective and real-time collection of potential patients with health consequences are warranted in the future.
35	Hung E et al	2023	Drug safety	New york	More extreme duplication in FDA Adverse Event Reporting System detected by literature reference normalization and fuzzy string matching	The objective of this analysis is to determine if variations of the same literature references observed in FAERS can be resolved with text normalization and fuzzy string matching.		✓						We have shown that these normalized references can be merged via fuzzy string matching to improve enumeration of all the individual case safety reports that refer to the same article. Inclusion of the PubMed ID and adherence to the Vancouver convention could facilitate identification of duplicates in the FAERS dataset. Awareness of this phenomenon may improve disproportionality analysis, especially in areas such as

For peer review only

No.	Author(s)	Year of publication	Journal	Country	Title	Specific Objectives	Study design		Categories					Key findings
								Extent	Preventio	Causes	Detection	Managemen	Implication	
36	Uppsala	2017		Sweden.	TEXT IS KEY FOR ELIMINATING DUPLICATE REPORTS	Spontaneous reporting systems are one of the best means of detecting rare adverse effects related to the use of medical products. The US Food and Drug Administration (FDA) monitors product safety issues in part by employing an automated tool to flag product-adverse effect pairs that are reported more commonly together than expected. This method may report a false association if there are duplicates of some cases in the data. Even a handful of duplicates can appear as a surge in reporting that triggers an alert and requires time and manual investigation to debunk				✓				Duplicate Cases – Two or more records describing the same occurrence of events – same adverse effect(s) experienced with the same medical product at the same time – for the same patient. Due to reporting inaccuracies, two records can be considered duplicates even if they do not contain an identical list of events.
37	Uppsala			Sweden.	Sharing pharmacovigilance data in the WHO Programme for International Drug Monitoring	The appointed National Centre (NC) for Pharmaco vigilance (PV) in each member country is responsible for sharing their Individual Case Safety Reports (ICSRs) with other members of the WHO Programme for International Drug Monitoring by sending the ICSRs to Uppsala Monitoring Centre (UMC) for inclusion in VigiBase, the WHO global ICSRs database.				✓	✓	✓		The minimum information required for an ICSR to be valid is a case identifier, reporter and patient information, and information on suspect medicine and reaction/event. All national case reports fulfilling the minimum requirements should be shared with the WHO Programme. However, quality of data and completeness of case reports are always important and reports should include as much information as possible to facilitate assessment. Free text/additional information can be provided in the original language. All information available on the original case report should be included when sending an ICSR to VigiBase, with the exception of confidential patient and reporter details that could potentially be used to identify individuals. More on the 1(2) importance of quality of ICSRs can be found on our website.
38	European Medicines Agency	2017		Netherlands	Guideline on good pharmacovigilance practices (GVP) Module VI Addendum I – Duplicate management of suspected adverse reaction reports	The revision contains the following changes: - Alignment with revision 2 of GVP Module VI; - Update of electronic reporting modalities of ICSRs in the new ICH-E2B(R3) format; - Update overall with the revised pharmacovigilance legislation as regards the roles and responsibilities of the Agency, the competent authorities in Member States as well as marketing authorisation holders in relation to the operation of duplicate detection and management of reports of suspected adverse reactions; - Guidance on how to inform the Agency of suspected duplicates in EudraVigilance; - Changes for consistent presentation of GVP docume			✓	✓	✓	✓	✓	Duplicate cases are generally managed through a process of merging two or more cases into one master case. This process can consist of one of the following approaches: 🕐 🕐 The master case can either be based on one of the existing cases, with information from the other subordinate duplicate cases added unless the same, or more precise, information is already present in the master case (this is referred to in this document as “Allocation of a master case”), or; The master case can be created as a new case combining the information from the subordinate duplicate cases (this is referred to in this document as “Creation of a master case”). Regardless of the approach chosen, the master case should always contain all case reference numbers from all subordinate duplicate cases, such that they can be easily traced. The master case should reflect the most accurate and up-to-date information available to the organisation.
39	Agency for Medicinal Products and Medical Devices of Croatia (HALMED).			Croatia	Strengthening Collaboration for Operating Pharmacovigilance in Europe (SCOPE).	SCOPE was divided into eight separate work packages, with five work packages focusing on pharmacovigilance topics to deliver specific and measureable objectives, ranging from improvements in Adverse Drug Reaction (ADR) reporting to the assessment of quality management systems.			✓	✓	✓	✓	✓	The first step in duplicate management is to identify possible ADR report duplicates. Screening for duplicates is usually carried out when a new report arrives in the database, i.e. during data entry or during the process of loading ICSRs that have been received electronically. Duplicates can be detected even before entering the case into the database – that is usually the case during manual data entry of cases received by paper, fax or phone. Duplicates can also be detected during periodic data review and the signal management process when detailed analysis of cases is performed.

For peer review only

No.	Author(s)	Year of publication	Journal	Country	Title	Specific Objectives	Study design		Categories					Key findings
								Extent	Preventio	Causes	Detection	Managemen	Implication	
40	Ritesh Bhangale	2017	National Library of Medicine	India	A day in the life of a pharmacovigilance case processor	t relies on information gathered from the collection of individual case safety reports and other pharmacoepidemiological data. The PV data processing cycle starts with data collection in computerized systems followed by complete data entry which includes adverse event coding, drug coding, causality and expectedness assessment, narrative writing, quality control, and report submissions followed by data storage and maintenance.			✓					For the case processor, every case is different, even within the same study. In fatal cases, the case processor will have to check many things such as the cause of death and autopsy details, whereas in other scenarios, the processor will have to check adverse events of special interests and important medical events and report the same to the stakeholders. The case processor should ensure that there are no grammatical/spelling errors in the case, while routing it to the next workflow. The case processor shares the best practices, which help other associates to do their job efficiently.
41	Priyanka Paygude et al	2013	Research Gate	India	Automated Data Validation Testing Tool for Data Migration Quality Assurance	The paper proposed automation of data migration validation testing process for quality assurance and risk control across industries			✓					Data migration is a tough project with high level of risks like time overruns, budget etc. Use of quality ETL tool will minimize the risk of defects in data of target database. Even though, testing of data for its validation is important and can‟t be overlooked. Testing validity of data using manual or just writing „MINUS‟ querise, are not the effective way causing risk with data quality. Here, the proposed system assure data quality using standardize way of data testing in migration projects across the enterprise, multiple platforms, and applications. Using proposed solution, one can save time, cost, and manual efforts; along with data quality assuranc
42	Saqib Hakak	2018	PLOS ONE	Malaysia.	A new split based searching for exact pattern matching for natural texts	In this work, we propose a novel idea to achieve both time efficiency and memory consumption by splitting query string for searching in Corpus.					✓			In this paper, we have proposed a novel idea for exact string matching to achieve both time and space efficiency regardless of query pattern length, dataset size and scripts. The proposed algorithm split given query pattern length into two halves and then it considers right halve for searching in a text. Once the match is found for right halve, the proposed algorithm uses left halve directly from the matched reference
43	Christen P et al	2012	SpringerLink		Data matching: concepts and techniques for record linkage, entity resolution, and duplicate detection. Springer: Data-centric systems and applications	First book on a topic of growing importance for applications Brings together research from various areas like databases, statistics, information retrieval, data mining, and machine learning Details the data matching process step by step Includes an overview of freely available data matching systems and a detailed discussion of practical aspects and limitations					✓			By providing the reader with a broad range of data matching concepts and techniques and touching on all aspects of the data matching process, this book helps researchers as well as students specializing in data quality or data matching aspects to familiarize themselves with recent research advances and to identify open research challenges in the area of data matching. To this end, each chapter of the book includes a final section that provides pointers to further background and research material.
44	Liu H et al				Categorization and Construction of Rule Based Systems	This paper also introduces rule based systems for classification tasks in detail.					✓			This brings new insights to researchers and practitioners and positions a new type of rule based systems for applications. It also motivates the use of data based approach in the context of machine learning for construction of complex systems instead of knowledge based approach. The importance of the data based approach is also highlighted. A special type of rule based system used for classification tasks is introduced in detail explaining the construction framework and reflecting some important aspects of choosing methods or techniques for rule generation, simplification and representation operations.
45	Wong CK et al	2015	Drug safety	Australia	Standardisation of the FAERS database: a systematic approach to manually recoding drug name variants	The aim of this study was to develop and implement a data cleaning protocol to identify and resolve drug nomenclature issues. The key 'data treatment' plan involved standardising drug names held in the FAERS database.					✓	✓		The millions of reports enclosed in the FAERS contain valuable information that is of interest to pharmacovigilance, toxicology and post-marketing surveillance researchers. With the standardisation of the drug nomenclature, the database can be better utilised by research groups around the world.

For peer review only

No.	Author(s)	Year of publication	Journal	Country	Title	Specific Objectives	Study design	Extent	Categories: Preventio	Causes	Detection	Managemen	Implication	Key findings
46	Jae Dong Yang	1997	Data & Knowledge Engineering	South Korea	A fuzzy match framework for rule-based programming	Proposed in this paper is a framework to support a semantic based inexact match with Fuzzy Match Predicate (F_MP). In a uniform way it allows matches including fuzzy linguistic variables as well as fuzzy numbers.					✓			In this paper, we demonstrate that: (1) F_MP is a uniform framework to provide the rule-based languages with fuzzy match facilities semantically enhanced, and that (2) its semantic conform well to that of the relational one
47	Kreimeyer k et al	2022	Drug safety	USA	Increased Confidence in Deduplication of Drug Safety Reports with Natural Language Processing of Narratives at the US Food and Drug Administration	Duplication has important negative implications for data analysis. We improved and optimized an existing deduplication algorithm that used both structured and free-text data, developed a web-based application to support data processing, and conducted a 6-month dedicated evaluation to assess the potential operationalization of the deduplication process in the FDA.					✓	✓	✓	Comparing algorithm predictions with reviewer determinations of duplicates for twenty-seven files for case series reviews (with a median size of 281 reports), the average pairwise recall and precision were equal to 0.71 (SD ± 0.32) and 0.67 (SD ± 0.34). Overall, reviewers felt confident about the algorithm and expressed their interest in using it. These findings support the operationalization of the deduplication process for case series review as a supplement to human review
48	Kreimeyer K et al	2017	Drug safety	USA	Using Probabilistic Record Linkage of Structured and Unstructured Data to Identify Duplicate Cases in Spontaneous Adverse Event Reporting Systems	We have developed a probabilistic record linkage algorithm for identifying duplicate cases in the US Vaccine Adverse Event Reporting System (VAERS) and the US Food and Drug Administration Adverse Event Reporting System (FAERS).					✓		✓	The algorithm was shown to be effective at identifying pre-linked duplicate VAERS reports. The narrative text was not shown to be a key component in the automated detection evaluation; however, it is essential for supporting the semi-automated approach that is likely to be deployed at the Food and Drug Administration, where medical reviewers will perform some manual review of the most highly ranked reports identified by the algorithm.
49	Harpaz R et al	2017	Journal of Bioinformatics	USA	Toward multimodal signal detection of adverse drug reactions	Improving mechanisms to detect adverse drug reactions (ADRs) is key to strengthening post-marketing drug safety surveillance. Signal detection is presently unimodal, relying on a single information source. Multimodal signal detection is based on jointly analyzing multiple information sources. Building on, and expanding the work done in prior studies, the aim of the article is to further research on multimodal signal detection, explore its potential benefits, and propose methods for its construction and evaluation.					✓			The results support the notion that utilizing and jointly analyzing multiple data sources may lead to improved signal detection. Given certain data and benchmark limitations, the early stage of development, and the complexity of ADRs, it is currently not possible to make definitive statements about the ultimate utility of the concept. Continued development of multimodal signal detection requires a deeper understanding the data sources used, additional benchmarks, and further research on methods to generate and synthesize signals.
50	Kreimeyer k et al	2021	Computers in Biology and Medicine	USA	Feature engineering and machine learning for causality assessment in pharmacovigilance: Lessons learned from application to the FDA Adverse Event Reporting System	Our objective was to support the automated classification of Food and Drug Administration (FDA) Adverse Event Reporting System (FAERS) reports for their usefulness in assessing the possibility of a causal relationship between a drug product and an adverse event.					✓			Causal inference from FAERS reports depends on many components with complex logical relationships that are yet to be made fully computable. Efforts focused on readily addressable tasks, such as quickly eliminating unassessable reports, fit naturally in SE's thought processes to provide real enhancements for FDA workflows.
51	Kim HR et al	2022	Medicine	South Korea	Analyzing adverse drug reaction using statistical and machine learning methods: A systematic review	This systematic review aimed to examine the analytical tools by considering original articles that utilized statistical and machine learning methods for detecting ADRs.					✓			Using the 72 main articles, this review provides guidelines on which databases are frequently utilized and which analysis methods can be connected. For statistical analysis, >90% of the cases were analyzed by disproportionate or regression analysis with each spontaneous reporting system (SRS) data or electronic medical record (EMR) data; for machine learning research, however, there was a strong tendency to analyze various data combinations. Only half of the DrugBank database was occupied, and the k-nearest neighbor method accounted for the greatest proportion.
52	CIOMS	2021		Sweden.	Making medicines safer for patients everywhere	This is the fourth of a series of special newsletters that describe the context of CIOMS activities and the work of its member organizations and partners. It describes what the Uppsala Monitoring Centre (UMC) is doing to support and promote patient safety through effective and global pharmacovigilance practice.					✓	✓		UMC will promote collaboration between all stake holders involved in drug risk management and will support harmonization, good pharmacovigilance practice and the dissemination of new guidelines. CIOMS is an important partner in this regard. New working groups on pharmacovigilance topics will be considered, for example to explore the link between pharmacovigilance practices and public health, or the role of pharmacoepidemiology as a tool to assess the impact of emerging adverse effects.

For peer review only

For peer review only - http://bmjopen.bmj.com/site/about/guidelines.xhtml

No.	Author(s)	Year of publication	Journal	Country	Title	Specific Objectives	Study design	Categories						Key findings
								Extent	Preventio	Causes	Detection	Managemen	Implication	
53			Website		Medra History	The need for a standardised medical terminology was identified by the ICH in the 1990s since there was not a standard terminology available that provided the scope and level of granularity needed by regulatory authorities and industry. In the past, the terminologies in use, such as COSTART, WHO-ART, J-ART, H-ARTS, ICD-9 and ICD-9CM were updated so infrequently that individual users created their own version and standardisation was lost						✓		MedDRA was based on a terminology belonging to the Medicines and Healthcare products Regulatory Agency (MHRA) of UK (previously named Medicines Control Agency) and was developed using the ICH process by the ICH partners, including WHO. The following significant milestones led to the initial release of MedDRA:
54	ORACLE	2021		North america	Argus Trusted safety case management	With caseloads inflating by 30–50% annually and regulations undergoing continuous change, efficient case management and maintaining compliance are important concerns. What if you could address these concerns while also reducing the manual effort of your safety and pharmacovigilance team by 50%						✓		Oracle Argus enabled greater and faster insight into product safety, improved productivity, and streamlined and automated safety reporting. Acorda is committed to patient safety, and the Oracle Argus platform helps us maintain high compliance standards."
55	Ablebits	2023			Ablebits. Ablebits Ultimate Suite	With these time-saving tools that collectively have over 300 useful features, you can automate most of your daily tasks in Excel and increase your productivity at least by 50%! It's many years of our experience with Office development embodied in the perfect code of solutions that work flawlessly on all Excel versions and all data sets.						✓		Finding and removing duplicates in Excel may be a real headache, especially when your worksheets contain thousands of rows. Duplicate Remover for Excel does the job quickly and perfectly – it can find and delete duplicates based on one or several key columns, identify unique values, highlight, copy or move the found entries
56	Brinker AD et al	2002	Am J Hematol	USA	Spontaneous reports of thrombocytopenia in association with quinine: clinical attributes and timing related to regulatory action	This report includes clinical attributes from the largest case series to date of apparently isolated thrombocytopenia in association with quinine and trends in the receipt of spontaneous adverse event reports to FDA's Center for Drug Evaluation and Research (CDER) for this drug-event combination in relation to regulatory action. In this study, we reviewed reports of spontaneous adverse drug events received by CDER.							✓	This case series confirms previous smaller series that suggest quinine-associated thrombocytopenia may present rapidly with symptoms of profound thrombocytopenia. Clinicians evaluating patients with new-onset and apparently idiopathic thrombocytopenia should maintain clinical vigilance for ingestion of quinine and elicit a detailed food/dietary supplement history from the patient
57	Azeroual O et al	2022	Multimodal Technol. Interact.	Germany	A Record Linkage-Based Data Deduplication Framework with DataCleaner Extension	To make the proposed framework usable we integrated it into a tool that is already used in practice, by developing a prototype of an extension for the well-known DataCleaner. The framework detects and visualises duplicates thereby identifying and providing the user with identified redundancies in a user-friendly manner allowing their further elimination. By removing the redundancies, the quality of the data is improved therefore improving analyses and decision-making. This study makes a call for other researchers to take a step towards the "golden record" that can be achieved when all data quality issues are recognised and resolved, thus moving towards absolute data quality.					✓		✓	In this paper, we presented a Record Linkage framework consisting of six interconnected steps, i.e., (1) data preparation, (2) search space definition, (3) attribute value comparison, (4) a decision model, (5) clustering of the duplicates and (6) verification. It was then transformed into an extension for the DataCleaner tool that was followed by validation through its application to the real-world RIS. This allowed us to easily and automatically identify duplicates stored in the system of which data holders and operators were not aware, with their subsequent elimination and enrichment of original records thereby contributing to their completeness, thus preparing data for further processing that should provide more accurate results.
58	Norén GN et al	2007	Data Mining and Knowledge Discovery	Sweden.	Duplicate detection in adverse drug reaction surveillance	We propose a duplicate detection method based on the hit-miss model for statistical record linkage described by Copas and Hilton, which handles the limited amount of training data well and is well suited for the available data (categorical and numerical rather than free text). We propose two extensions of the standard hit-miss model: a hit-miss mixture model for errors in numerical record fields and a new method to handle correlated record fields,					✓			In this paper we have introduced two generalisations of the standard hit-missmodel and demonstrated the usefulness of the extended hit-miss model forautomated duplicate detection in WHO drug safety data. Our results indicatethat the hit-miss model can detect a significant proportion of the duplicateswithout generating many false leads. Its strong theoretical basis

Appendix IV: List of excluded articles

No.	PMID	Title	Authors	Citation	First Aut	Journal/Book	Publication Year
1	22360774	Pharmacovigilance	Edwards IR.	Br J Clin Pharmacol. 2012 Jun;73(6	Edwards I	Br J Clin Pharmacol	2012
2	25123728	"Big data" and the electronic health record	Ross MK, Wei V	Yearb Med Inform. 2014 Aug 15;9(	Ross MK	Yearb Med Inform	2014
3	33634545	Suitability of databases in the Asia-Pacific for collaborative monitor	Duszynski KM,	Pharmacoepidemiol Drug Saf. 2021	Duszynsk	Pharmacoepidemiol Drug S	2021
4	35179704	High-Dimensional Propensity Score-Adjusted Case-Crossover for D	Volatier E, Salv	Drug Saf. 2022 Mar;45(3):275-285.	Volatier E	Drug Saf	2022
5	29474173	Possibility of Database Research as a Means of Pharmacovigilance i	Hirano Y, Asam	Value Health Reg Issues. 2018 May	Hirano Y	Value Health Reg Issues	2018
6	32243569	Different Strategies to Execute Multi-Database Studies for Medicine	Gini R, Sturkenb	Clin Pharmacol Ther. 2020 Aug;108	Gini R	Clin Pharmacol Ther	2020
7	31464008	Establishment of the MID-NET(®) medical information database ne	Yamaguchi M, I	Pharmacoepidemiol Drug Saf. 2019	Yamaguchi	Pharmacoepidemiol Drug S	2019
8	31933254	Artificial Intelligence Within Pharmacovigilance: A Means to Identi	Mockute R, Des	Pharmaceut Med. 2019 Apr;33(2):1	Mockute I	Pharmaceut Med	2019
9	35239736	Evaluation of pharmacovigilance systems for reporting medication e	Sabblah GT, Sea	PLoS One. 2022 Mar 3;17(3):e0264	Sabblah G	PLoS One	2022
10	30445979	Organizational capacities of national pharmacovigilance centres in A	Ampadu HH, Ho	Global Health. 2018 Nov 16;14(1):1	Ampadu H	Global Health	2018
11	36097239	Artificial Intelligent Context-Aware Machine-Learning Tool to Dete	Roosan D, Law	J Med Toxicol. 2022 Oct;18(4):311-	Roosan D	J Med Toxicol	2022
12	29714535	An Alternative to Disproportionality: A Frequency-Based Method fo	Jokinen JD, Liev	Ther Innov Regul Sci. 2018 May;52	Jokinen JI	Ther Innov Regul Sci	2018
13	30185579	Electronic healthcare databases in Europe: descriptive analysis of ch	Pacurariu A, Plu	BMJ Open. 2018 Sep 5;8(9):e02309	Pacurariu	BMJ Open	2018
14	32026758	Combining big data search analytics and the FDA Adverse Event Re	Spachos D, Siaf	Health Informatics J. 2020 Sep;26(3	Spachos D	Health Informatics J	2020
15	23512870	Drug safety data mining with a tree-based scan statistic	Kulldorff M, Da	Pharmacoepidemiol Drug Saf. 2013	Kulldorff	Pharmacoepidemiol Drug S	2013
16	25151493	Text mining for adverse drug events: the promise, challenges, and st	Harpaz R, Calla	Drug Saf. 2014 Oct;37(10):777-90.	Harpaz R	Drug Saf	2014
17	26606038	Predictive modeling of structured electronic health records for adver	Zhao J, Henriks	BMC Med Inform Decis Mak. 2015	Zhao J	BMC Med Inform Decis M	2015
18	24599513	Reducing the noise in signal detection of adverse drug reactions by s	Grundmark B, H	Eur J Clin Pharmacol. 2014 May;70	Grundmar	Eur J Clin Pharmacol	2014
19	36380085	A real-world pharmacovigilance study of FDA Adverse Event Repo	Yin Y, Shu Y, Z	Sci Rep. 2022 Nov 15;12(1):19555.	Yin Y	Sci Rep	2022
20	31677951	ADVANCE: Towards near real-time monitoring of vaccination cove	Bollaerts K, de S	Vaccine. 2020 Dec 22;38 Suppl 2:B	Bollaerts I	Vaccine	2020
21	21219405	Using primary care prescribing databases for pharmacovigilance	Mohamed IN, H	Br J Clin Pharmacol. 2011 Feb;71(2	Mohamed	Br J Clin Pharmacol	2011
22	35691583	Development and external validation of a 1- and 5-year fracture pred	Tebé C, Pallarès	Bone. 2022 Sep;162:116469. doi: 1	Tebé C	Bone	2022
23	31198563	Pharmacovigilance systems in resource-limited settings: an evaluativ	Abiri OT, Johns	J Pharm Policy Pract. 2019 Jun 11;1	Abiri OT	J Pharm Policy Pract	2019
24	25123721	Big data - smart health strategies. Findings from the yearbook 2014 s	Koutkias V, Thi	Yearb Med Inform. 2014 Aug 15;9(	Koutkias V	Yearb Med Inform	2014
25	36147337	Analysis of pharmacovigilance databases for spontaneous reports of	Pozsgai K, Szűc	Front Pharmacol. 2022 Sep 6;13:96	Pozsgai K	Front Pharmacol	2022
26	23331229	Automated method for detecting increases in frequency of spontaneo	DuMouchel W,	J Biopharm Stat. 2013;23(1):161-77	DuMouch	J Biopharm Stat	2013
27	26819727	Pharmacoepidemiology in Japan: medical databases and research ac	Tanaka S, Seto I	J Pharm Health Care Sci. 2015 May	Tanaka S	J Pharm Health Care Sci	2015
28	34694230	Adverse Drug Event Prediction Using Noisy Literature-Derived Kno	Dasgupta S, Jay	JMIR Med Inform. 2021 Oct 25;9(1	Dasgupta	JMIR Med Inform	2021
29	36438789	Data mining and safety analysis of BTK inhibitors: A pharmacovigil	Wan Q, Li Q, La	Front Pharmacol. 2022 Nov 11;13:9	Wan Q	Front Pharmacol	2022
30	28595649	Data-driven prediction of adverse drug reactions induced by drug-dr	Liu R, AbdulHa	BMC Pharmacol Toxicol. 2017 Jun	Liu R	BMC Pharmacol Toxicol	2017
31	31004158	Prevalence and incidence of narcolepsy in a US health care claims d	Scheer D, Schw	Sleep. 2019 Jul 8;42(7):zsz091. doi:	Scheer D	Sleep	2019
32	31507029	Data variability across Canadian administrative health databases: Di	Doyle CM, Lix	Pharmacoepidemiol Drug Saf. 2020	Doyle CM	Pharmacoepidemiol Drug S	2020
33	32564242	Risk Factor Considerations in Statistical Signal Detection: Using Su	Sandberg L, Taa	Drug Saf. 2020 Oct;43(10):999-100	Sandberg	Drug Saf	2020
34	23070598	Comparison of two drug safety signals in a pharmacovigilance data r	Tubert-Bitter P,	Stat Methods Med Res. 2016 Apr;2	Tubert-Bi	Stat Methods Med Res	2016
35	29446035	Sorting Through the Safety Data Haystack: Using Machine Learning	Comfort S, Pere	Drug Saf. 2018 Jun;41(6):579-590.	Comfort S	Drug Saf	2018
36	24787710	Paediatric pharmacovigilance: use of pharmacovigilance data mining	Kajungu DK, Er	PLoS One. 2014 May 1;9(5):e96388	Kajungu I	PLoS One	2014
37	28646789	Data-mining for detecting signals of adverse drug reactions of fluox	Kim S, Park K,	Psychiatry Res. 2017 Oct;256:237-2	Kim S	Psychiatry Res	2017
38	21701609	Information technology in pharmacovigilance: Benefits, challenges,	Lu Z.	Drug Healthc Patient Saf. 2009;1:35	Lu Z	Drug Healthc Patient Saf	2009
39	27868278	Differences in VigiBase® reporting of aminoglycoside and capreom	Sagwa EL, Souv	Pharmacoepidemiol Drug Saf. 2017	Sagwa EL	Pharmacoepidemiol Drug S	2017
40	23921799	Triptans and serious adverse vascular events: data mining of the FD	Roberto G, Picc	Cephalalgia. 2014 Jan;34(1):5-13. d	Roberto G	Cephalalgia	2014
41	31673326	Evaluation of quantitative signal detection in EudraVigilance for orp	Sardella M, Lun	Ther Adv Drug Saf. 2019 Oct 21;10	Sardella M	Ther Adv Drug Saf	2019

No.	PMID	Title	Authors	Citation	First Aut	Journal/Book	Publication Year
42	36050484	Detecting early safety signals of infliximab using machine learning a	Lee JE, Kim JH,	Sci Rep. 2022 Sep 1;12(1):14869. d	Lee JE	Sci Rep	2022
43	30324671	Significance of data mining in routine signal detection: Analysis bas	Fukazawa C, Hi	Pharmacoepidemiol Drug Saf. 2018	Fukazawa	Pharmacoepidemiol Drug S	2018
44	31156424	Computational Advances in Drug Safety: Systematic and Mapping R	Natsiavas P, Ma	Front Pharmacol. 2019 May 17;10:4	Natsiavas	Front Pharmacol	2019
45	28335691	Cases of drug-induced Torsade de Pointes: a review of Belgian cases	Vandael E, Van	Acta Clin Belg. 2017 Dec;72(6):385	Vandael E	Acta Clin Belg	2017
46	16134080	Signal detection in pharmacovigilance: empirical evaluation of data	Chan KA, Haub	Pharmacoepidemiol Drug Saf. 2005	Chan KA	Pharmacoepidemiol Drug S	2005
47	35745593	Pharmacovigilance Signals of the Opioid Epidemic over 10 Years: D	Chiappini S, Vi	Pharmaceuticals (Basel). 2022 May	Chiappini	Pharmaceuticals (Basel)	2022
48	36050787	Challenges and opportunities for mining adverse drug reactions: pers	Gonzalez-Herna	Database (Oxford). 2022 Sep 2;202	Gonzalez-	Database (Oxford)	2022
49	33518634	[Role and Applicability of Spontaneous Reporting Databases in Med	Sakai T.	Yakugaku Zasshi. 2021;141(2):165-	Sakai T	Yakugaku Zasshi	2021
50	32169289	Uses of pharmacovigilance databases: An overview	Bihan K, Lebrur	Therapie. 2020 Nov-Dec;75(6):591-	Bihan K	Therapie	2020
51	35904529	The Use of Artificial Intelligence in Pharmacovigilance: A Systemat	Salas M, Petrace	Pharmaceut Med. 2022 Oct;36(5):2	Salas M	Pharmaceut Med	2022
52	31623850	Pharmacovigilance - The next chapter	Moore N, Berda	Therapie. 2019 Dec;74(6):557-567.	Moore N	Therapie	2019
53	27224991	[Pharmacovigilance in Germany : It is about time]	Douros A, Scha	Internist (Berl). 2016 Jun;57(6):616	Douros A	Internist (Berl)	2016
54	33598764	Construction and analysis of a database for medication errors in a ph	Alj L, Benkiran	Eur J Clin Pharmacol. 2021 Aug;77	Alj L	Eur J Clin Pharmacol	2021
55	35788724	Evolution of adverse drug reactions reporting systems: paper based t	Madhushika MT	Eur J Clin Pharmacol. 2022 Sep;78(	Madhushi	Eur J Clin Pharmacol	2022
56	23640186	The past, present and perhaps future of pharmacovigilance: homage	Moore N.	Eur J Clin Pharmacol. 2013 May;69	Moore N	Eur J Clin Pharmacol	2013
57	35579806	Artificial Intelligence in Pharmacovigilance: An Introduction to Ter	Aronson JK.	Drug Saf. 2022 May;45(5):407-418	Aronson J	Drug Saf	2022
58	36251280	Development of a multivariate prediction model to identify individu	Gosselt HR, Baz	Pharmacoepidemiol Drug Saf. 2022	Gosselt H	Pharmacoepidemiol Drug S	2022
59	26575203	Big Data and Adverse Drug Reaction Detection	Harpaz R, DuM	Clin Pharmacol Ther. 2016 Mar;99(	Harpaz R	Clin Pharmacol Ther	2016
60	32176553	The potential role of big data in the detection of adverse drug reactio	Sultana J, Trifir	Expert Rev Clin Pharmacol. 2020 M	Sultana J	Expert Rev Clin Pharmacol	2020
61	24698186	[Terappel: description of a computerised database and examples of s	Vial T, Gouraud	Therapie. 2014 Jan-Feb;69(1):31-8.	Vial T	Therapie	2014
62	28840504	From Big Data to Smart Data for Pharmacovigilance: The Role of H	Trifirò G, Sultar	Drug Saf. 2018 Feb;41(2):143-149.	Trifirò G	Drug Saf	2018
63	22315152	Electronic healthcare databases for active drug safety surveillance: is	Coloma PM, Tri	Pharmacoepidemiol Drug Saf. 2012	Coloma P	Pharmacoepidemiol Drug S	2012
64	19745234	The EU-ADR project: preliminary results and perspective	Trifiro G, Fourr	Stud Health Technol Inform. 2009;1	Trifiro G	Stud Health Technol Inform	2009
65	26092163	[Benefits of large healthcare databases for drug risk research]	Garbe E, Pigeot	Bundesgesundheitsblatt Gesundheit	Garbe E	Bundesgesundheitsblatt Ges	2015
66	23670805	Pilot evaluation of an automated method to decrease false-positive si	Avillach P, Salv	Pharmacoepidemiol Drug Saf. 2014	Avillach P	Pharmacoepidemiol Drug S	2014
67	23208789	The EU-ADR Web Platform: delivering advanced pharmacovigilanc	Oliveira JL, Lop	Pharmacoepidemiol Drug Saf. 2013	Oliveira J	Pharmacoepidemiol Drug S	2013
68	31912439	Towards Automating Adverse Event Review: A Prediction Model fo	Muñoz MA, Dal	Drug Saf. 2020 Apr;43(4):329-338.	Muñoz M	Drug Saf	2020
69	22873501	Implemented data mining and signal management systems on sponta	de Almeida Vie	Curr Drug Saf. 2012 Apr;7(2):170-5	de Almeid	Curr Drug Saf	2012
70	22549283	Novel data-mining methodologies for adverse drug event discovery a	Harpaz R, DuM	Clin Pharmacol Ther. 2012 Jun;91(6	Harpaz R	Clin Pharmacol Ther	2012
71	18523760	Pharmacovigilance: methods, recent developments and future perspe	Härmark L, van	Eur J Clin Pharmacol. 2008 Aug;64	Härmark L	Eur J Clin Pharmacol	2008
72	23072620	Global patterns of adverse drug reactions over a decade: analyses of	Aagaard L, Stra	Drug Saf. 2012 Dec 1;35(12):1171-	Aagaard L	Drug Saf	2012
73	15366975	The general practice research database: role in pharmacovigilance	Wood L, Martin	Drug Saf. 2004;27(12):871-81. doi:	Wood L	Drug Saf	2004
74	23761351	Detection of adverse drug reactions using hospital databases-a natior	Miguel A, Bern	Pharmacoepidemiol Drug Saf. 2013	Miguel A	Pharmacoepidemiol Drug S	2013
75	15366974	Data quality management in pharmacovigilance	Lindquist M.	Drug Saf. 2004;27(12):857-70. doi:	Lindquist	Drug Saf	2004
76	15460169	Pharmacovigilance in the 21st century: new systematic tools for an c	Szarfman A, To	Pharmacotherapy. 2004 Sep;24(9):1	Szarfman	Pharmacotherapy	2004
77	16111454	The role of data mining in pharmacovigilance	Hauben M, Mad	Expert Opin Drug Saf. 2005 Sep;4(5	Hauben M	Expert Opin Drug Saf	2005
78	15221098	[Databases as a source for monitoring systems of drug safety]	Pigeot I, Ahrens	Bundesgesundheitsblatt Gesundheit	Pigeot I	Bundesgesundheitsblatt Ges	2004
79	20721787	Mining pharmacovigilance data using Bayesian logistic regression w	An L, Fung KY,	J Biopharm Stat. 2010 Sep;20(5):99	An L	J Biopharm Stat	2010
80	16180934	Data mining in pharmacovigilance: the need for a balanced perspecti	Hauben M, Pata	Drug Saf. 2005;28(10):835-42. doi:	Hauben M	Drug Saf	2005
81	17604418	Signal detection in the pharmaceutical industry: integrating clinical a	Hauben M.	Drug Saf. 2007;30(7):627-30. doi: 1	Hauben M	Drug Saf	2007
82	15360888	A knowledge based approach for automated signal generation in pha	Henegar C, Bou	Stud Health Technol Inform. 2004;1	Henegar C	Stud Health Technol Inform	2004
83	12650632	Application of quantitative signal detection in the Dutch spontaneou	van Puijenbroek	Drug Saf. 2003;26(5):293-301. doi:	van Puijer	Drug Saf	2003

No.	PMID	Title	Authors	Citation	First Aut	Journal/Book	Publication Year
84	Yuan Luo	Natural Language Processing for EHR-Based Pharmacovigilance: A	Drug Safety	Evanston USA	Yuan Luo	This article is a comprehens	2017
85	ECRI INST	Wrong-Record, Wrong-Data Errors with Health IT Systems.		Pennsylvania	ECRI INS	We define data integrity fai	2015
86	European M	Guideline on good pharmacovigilance practices (GVP) Module IX Addendum I – Mo		Netherlands	European	The decision based on the a	2015
87	FDA	Questions and Answers on FDA's Adverse Event Reporting System (FAERS)		USA	FDA	What is FAERS? The FDA	2018
88	Liu M et al	Comparative analysis of pharmacovigilance methods in the detection	Jamia	New Jersey	Liu M et a	Medication safety requires	2013
89	Norén GN	A hit-miss model for duplicate detection in the WHO drug safety database		Sweden.	Norén GN	We present two new gener	2005
90	Niklas Nor	A hit-miss model for duplicate detection in the WHO drug safety dat	Research gate		Niklas No	We demonstrate the effect	2005
91	eudrac	https://www.eudrac.com/news/good-pharmacovigilance-practice-gui	eudrac	Germany			
92	PV Drug Sa	https://www.linkedin.com/posts/pv-drug-safety-academy-84152822	PV Drug Safety	England			
93	Drug Regu	https://www.dra.gov.pk/wp-content/uploads/2022/04/Good-Pharmac	Drug Regulatory	Pakistan			
94	cod-researc	https://cod-research.com/ease-icsr-handling-through-early-duplicate-	cod-research.co	India			
95	ECA Found	https://www.gmp-compliance.org/guidelines/gmp-guideline/eudrale	ECA Foundation	Germany			
96	Applied Cl	https://www.appliedclinicaltrialsonline.com/view/pharmacovigilanc	Applied Clinical	Fort Washington USA			
97	ClinosolInd	https://www.slideshare.net/ClinosolIndia/duplicate-case	ClinosolIndia	India			
99	clarivate	https://clarivate.com/blog/seeing-double-eliminating-duplicate-refer	clarivate	Jersey			

For peer review only

Navigating Duplication in Pharmacovigilance Databases: A Scoping Review

Journal:	*BMJ Open*
Manuscript ID	bmjopen-2023-081990.R1
Article Type:	Original research
Date Submitted by the Author:	15-Mar-2024
Complete List of Authors:	Kiguba, Ronald; Makerere University, Department of Pharmacology and Therapeutics<br>Isabirye, Gerald; National Drug Authority<br>Mayengo, Julius; National Drug Authority<br>Owiny, Jonathan; National Drug Authority<br>Tregunno, Phil; Medicines and Healthcare Products Regulatory Agency<br>Harrison, Kendal; Medicines and Healthcare Products Regulatory Agency<br>Pirmohamed, Munir; University of Liverpool Institute of Integrative Biology<br>Ndagije, Helen ; National Drug Authority
**Primary Subject Heading**:	Pharmacology and therapeutics
Secondary Subject Heading:	Epidemiology, Public health
Keywords:	Adverse events < THERAPEUTICS, Pharmacology < TROPICAL MEDICINE, CLINICAL PHARMACOLOGY

I, the Submitting Author has the right to grant and does grant on behalf of all authors of the Work (as defined in the below author licence), an exclusive licence and/or a non-exclusive licence for contributions from authors who are: i) UK Crown employees; ii) where BMJ has agreed a CC-BY licence shall apply, and/or iii) in accordance with the terms applicable for US Federal Government officers or employees acting as part of their official duties; on a worldwide, perpetual, irrevocable, royalty-free basis to BMJ Publishing Group Ltd ("BMJ") its licensees and where the relevant Journal is co-owned by BMJ to the co-owners of the Journal, to publish the Work in this journal and any other BMJ products and to exploit all rights, as set out in our licence.

The Submitting Author accepts and understands that any supply made under these terms is made by BMJ to the Submitting Author unless you are acting as an employee on behalf of your employer or a postgraduate student of an affiliated institution which is paying any applicable article publishing charge ("APC") for Open Access articles. Where the Submitting Author wishes to make the Work available on an Open Access basis (and intends to pay the relevant APC), the terms of reuse of such Open Access shall be governed by a Creative Commons licence – details of these licences and which Creative Commons licence will apply to this Work are set out in our licence referred to above.

Other than as permitted in any relevant BMJ Author's Self Archiving Policies, I confirm this Work has not been accepted for publication elsewhere, is not being considered for publication elsewhere and does not duplicate material already published. I confirm all authors consent to publication of this Work and authorise the granting of this licence.

For peer review only

Navigating Duplication in Pharmacovigilance Databases: A Scoping Review

Authors:

Ronald Kiguba[1], Gerald Isabirye[2], Julius Mayengo[2], Jonathan Owiny[2], Phil Tregunno[3], Kendal Harrison[3], Munir Pirmohamed[4], Helen B. Ndagije[2]*

*Correspondence to: Ronald Kiguba (Tel: +256 701 840683; Email: kiguba@gmail.com)

Affiliations:

[1]Department of Pharmacology and Therapeutics, College of Health Sciences, Makerere University, Kampala, Uganda

[2]National Pharmacovigilance Centre, National Drug Authority, Kampala, Uganda

[3]Safety and Surveillance Group, Medicines and Healthcare products Regulatory Agency, London, United Kingdom

[4]Centre for Drug Safety Science and Wolfson Centre for Personalised Medicine, Institute of Systems, Molecular and Integrative Biology, University of Liverpool, Liverpool, United Kingdom

Running Head:

Duplication in Pharmacovigilance Databases

For peer review only - http://bmjopen.bmj.com/site/about/guidelines.xhtml

Abstract

Objectives:

Pharmacovigilance databases play a critical role in monitoring drug safety. The duplication of reports in pharmacovigilance databases, however, undermines their data integrity. This scoping review sought to provide a comprehensive understanding of duplication in pharmacovigilance databases worldwide.

Design:

A scoping review.

Data sources:

Reviewers comprehensively searched the literature in PubMed, Web of Science, Wiley Online Library, EBSCOhost, Google Scholar, and other relevant websites.

Eligibility criteria:

Peer-reviewed publications and grey literature, without language restriction, describing duplication and/or methods relevant to duplication in pharmacovigilance databases from inception to 1 September 2023.

Data extraction and synthesis:

We used the Joanna Briggs Institute guidelines for scoping reviews and conformed with the Preferred Reporting Items for Systematic Reviews and Meta-Analyses Extension for Scoping Reviews. Two reviewers independently screened titles, abstracts, and full-texts. One reviewer extracted the data and performed descriptive analysis, which the second reviewer assessed. Disagreements were resolved by discussion and consensus or in consultation with a third reviewer.

Results:

We screened 22745 unique titles and 156 were eligible for full-text review. Of the 156 titles, 58 (47 peer-reviewed; 11 grey literature) fulfilled the inclusion criteria for the scoping review. Included titles addressed the extent (5 papers), prevention strategies (15 papers), causes (32 papers), detection methods (25 papers), management strategies (24 papers), and implications (14 papers) of duplication in pharmacovigilance databases. The papers overlapped, discussing more than one field. Advances in artificial intelligence, particularly natural language processing, hold promise in enhancing the efficiency and precision of deduplication of large and complex pharmacovigilance databases.

Conclusion:

Duplication in pharmacovigilance databases compromises risk assessment and decision-making, potentially threatening patient safety. Therefore, efficient duplicate prevention, detection, and management is essential for more reliable pharmacovigilance data. To minimise duplication, consistent use of worldwide unique identifiers as the key case-identifiers is recommended alongside recent advances in artificial intelligence.

Keywords: Adverse event reporting, Duplication, Duplicate management, Pharmacovigilance, Pharmacovigilance database.

For peer review only

Article summary

Strengths and limitations of this study:

- The study involved an extensive literature search on a global scale including both peer-reviewed publications and the grey literature.
- This review adhered to the rigorous methodology published by the Joanna Briggs Institute for scoping reviews.
- We employed an *a priori* PubMed search strategy, which was then adapted across other peer-reviewed research databases (Web of Science, Wiley Online Library, EBSCOhost) using the Polyglot Search Translator. Additionally, our search for grey literature followed the guidelines outlined in the Canadian Agency for Drugs and Technologies in Health Guide.

For peer review only

For peer review only - http://bmjopen.bmj.com/site/about/guidelines.xhtml

INTRODUCTION:

Pharmacovigilance is crucial for drug safety as it promotes the prevention, detection, and evaluation of suspected adverse reactions to minimize their impact on patient health.[1, 2] One of the challenges of pharmacovigilance is the duplication of cases in pharmacovigilance databases, which distorts drug safety and efficacy assessment, and disrupts decision-making.[3-5] Duplication is defined as multiple, unconnected records that refer to the same potential adverse event.[3, 4, 6] From 2000 to 2010, about 2.5% of reports with adequate information for duplicate analysis in the World Health Organization (WHO) global pharmacovigilance database were duplicates; the percentage was higher for reports from the literature (11%) and those with fatal outcomes (5%).[4] To promote the accuracy of pharmacovigilance databases, it is important to routinely screen and remove duplicate cases.[4]

The duplication of cases should, however, be differentiated from the replication of records. Replication includes reproducing or recreating pharmacovigilance reports for various purposes, e.g., research, regulatory compliance, or data analysis. Replication involves making copies of reports for different stakeholders or systems without necessarily meaning that the content or details of the reports are identical. Replication could result in duplication if multiple reporters, e.g., pharmaceutical companies, report the same case to the same regulatory authority for compliance reasons.[7]

Global, regional, and country-level guidelines have been implemented to address duplication in pharmacovigilance databases. Global pharmacovigilance initiatives, such as the International Council for Harmonization (ICH) guidelines and the Uppsala Monitoring Centre, which coordinates the WHO Programme for International Drug Monitoring, promote standardization and harmonization of pharmacovigilance activities, that are essential for accurate and efficient data collection, analysis, and reporting.[2, 8-11] In the US, the Food and Drug Administration (FDA) uses the Adverse Event Reporting System (FAERS) to manage adverse event reports, including duplicates.[12, 13] In Europe, the European Medicines Agency (EMA) oversees the safety of medicines via the EudraVigilance system which uses unique identification numbers for each report and has a built-in deduplication mechanism to identify and eliminate duplicate reports.[14, 15] In Asia, a deduplication technique was adopted by the Pharmacovigilance Program of India, together with the assignment of unique identification numbers and data standardization. Similar to this, in China, the National Medical Products Administration has put in place a system called the Adverse Drug Reaction Monitoring and Re-evaluation System that incorporates a deduplication mechanism for reporting and monitoring suspected adverse reactions.[16] In Africa, WHO built a regional pharmacovigilance centre in Ghana to support the region's pharmacovigilance systems through training, including duplicate management.[17] Pharmaceutical companies are also responsible for managing duplicate reports. Comprehensive and integrated pharmacovigilance systems are required to effectively manage data from multiple sources, e.g. electronic health records, spontaneous reporting databases, social media, and digital devices, among others; and to eliminate duplicate reports.[18, 19] These systems should ensure compliance with the global pharmacovigilance regulations and standards.

Duplicates arise from various causes including data entry errors,[20, 21] multiple reporters and reporting channels,[22, 23] pressure from mandatory reporting requirements for healthcare professionals and pharmaceutical companies,[4, 24] challenges in data integration and harmonization in pharmacovigilance databases[19], multiple representation of the same data due to the merging of pharmacovigilance databases,[6] lack of standardized data management practices,[6] and incomplete case information of key data elements.[12, 25]

For peer review only - http://bmjopen.bmj.com/site/about/guidelines.xhtml

The implications of duplication are far-reaching, hence the need for the routine screening and elimination of duplicates from pharmacovigilance databases.[6] Software programs significantly improve efficiency, accuracy, and consistency in the detection and management of duplicates. The Uppsala Monitoring Centre developed the software VigiMatch to detect potential duplicates in VigiBase, the global pharmacovigilance database.[26] VigiBase can merge duplicate reports identified by VigiMatch.[4, 27] Automated standalone software programs have been developed to handle large volumes of data and can be customized to meet specific regulatory requirements.[28-30]

Advances in technology, e.g., machine learning and natural language processing hold promise in augmenting the efficiency and precision of pharmacovigilance efforts, particularly in the deduplication of large and complex pharmacovigilance databases.[5],[31, 32] Deduplication methods are, however, impeded by the high extent of incomplete case information which makes it difficult to conduct reliable duplicate analysis.[4] Moreover, systems ought to avoid over-reliance on automated standalone software programs as they could generate false signals. Instead, holistic methods which include manual review alongside automated processes should be preferred.[33, 34] The objective of this scoping review is to provide a comprehensive overview of duplication in pharmacovigilance databases on a global scale. This review addresses the extent, causes, prevention strategies, detection methods, management and impact of duplication in pharmacovigilance databases.

METHODS

Research Questions:

1. What is the extent of duplication in pharmacovigilance databases worldwide?
2. What are the causes of, and prevention strategies, detection methods and management strategies for duplicate reports in pharmacovigilance databases worldwide?
3. What is the impact of duplicate reports in pharmacovigilance databases worldwide?

Study Design:

This scoping review was conducted in accordance with the Joanna Briggs Institute (JBI) guidelines for scoping reviews[35] and conformed with the Preferred Reporting Items for Systematic Reviews and Meta-Analyses Extension for Scoping Reviews (PRISMA-ScR).[36] The protocol was developed according to the JBI scoping review template and registered with Open Science Framework (DOI 10.17605/OSF.IO/PBVF3).

Definition of duplication:

Duplication was defined as multiple, unconnected records that refer to the same potential adverse event.[3, 4, 6]

Eligibility Criteria:

The inclusion criteria for selection of articles, reports, and guidelines, without language restriction, were:

1. Articles, reports, and guidelines related to duplication and/ or methods relevant to duplication in pharmacovigilance databases
2. Articles published in peer-reviewed journals and grey literature from official websites of regulatory authorities and pharmacovigilance organizations.

For peer review only - http://bmjopen.bmj.com/site/about/guidelines.xhtml

Articles, reports, and guidelines were excluded if, on full-text review, they did not align with the precise objectives of the scoping review. This rigorous screening procedure ensured that only relevant papers were included in the analysis.

For peer review only

For peer review only - http://bmjopen.bmj.com/site/about/guidelines.xhtml

Search strategy:

The search strategy was designed to identify all relevant studies related to the research question(s). We therefore searched multiple databases, including PubMed, Web of Science, Wiley Online Library, EBSCOhost, Google Scholar and other relevant websites including regulatory authorities and pharmacovigilance organizations, from inception to 1 September 2023. The reference lists of relevant studies were also screened. The PubMed search string for peer-reviewed publications is detailed in **Appendix I** and was adapted across the other peer-reviewed research databases using the Polyglot Search Translator.[37] The grey literature search was based on the Canadian Agency for Drugs and Technologies in Health Guide[38] (see **Appendix II)**.

Study selection:

Two reviewers independently screened the titles and abstracts of all the identified articles for eligibility. Full-text articles were retrieved for all potentially eligible studies and independently assessed for inclusion by two reviewers. Disagreements were resolved by discussion and consensus or in consultation with a third reviewer.

Data extraction:

Data extraction was done by Gerald Isabirye (GI) using a tool created by the reviewers, as detailed in **Appendix III**. This tool was designed to capture various parameters, including the Author, Year of publication, Journal, Country, Title, Specific Objectives, Study design, Categories (extent, prevention, causes, detection, management and impact of duplication in pharmacovigilance databases), and key findings. The extracted data were reviewed by Ronald Kiguba (RK) and a third reviewer, Helen B. Ndagije (HBN), and revised accordingly.

Data analysis and presentation:

The extracted data are provided in a descriptive summary according to the review's objectives. The risk of bias or quality assessment was not done across the included studies. This decision aligns with the guidelines outlined by the JBI because a scoping review is not intended to appraise the risk of bias of a cumulative body of evidence.[36] Instead, it focuses on providing a comprehensive overview of the existing literature.

Patient and public involvement:

Patients and the public did not participate in the design, conduct, reporting and drafting of the dissemination plans of this research.

Regions and countries:

The regions and countries of papers included in this review are presented in **Appendix IV**. The papers were from the Asia Pacific region, Europe, Australia and North Africa.

For peer review only

For peer review only - http://bmjopen.bmj.com/site/about/guidelines.xhtml

RESULTS

We screened 22745 unique titles and 156 (127 peer-reviewed; 29 grey literature) fulfilled the inclusion criteria for the scoping review (see **Table 1, Figure 1** and **supplementary Table 1**). were eligible for full-text review. Of the 156 titles, 58 (47 peer-reviewed; 11 grey literature) fulfilled the inclusion criteria for the scoping review (see **Table 1, Figure 1** and **supplementary Table 1**). The included titles addressed the extent of duplication (5 papers), prevention strategies (15 papers), causes (32 papers), detection methods (25 papers), management (24 papers) and implications (14 papers) of duplicate reports. The papers overlapped, discussing more than one field. The list of excluded titles is provided in **Appendix V**.

Extent of duplication: The prevalence of duplication in the WHO global pharmacovigilance database, VigiBase, was 2.5% for reports submitted from 2000 to 2010, based on half [51%, 1.9 million of 3.7 million) of the reports with sufficient information for duplicate analysis.[4] Duplication rates were highest for reports from the published literature, e.g., peer-reviewed articles (11%), and fatal outcomes (5%).[4] In Europe, the prevalence of duplication in VigiBase was quite high for some countries [e.g., Czech Republic (15%); Austria (15%); Switzerland (4.7%)] and low for others [e.g., United Kingdom (1.4%); Denmark (1%); Spain (0.7%)].[4] In Asia, the extent of duplication in VigiBase was high in Korea (9.2%) and low in Japan (0.8%). In the US, duplicates represented 5-20% of reports in the FAERS by the year 2000.[13] A very high level of duplication, from 66% to 87%, most likely fuelled by replication, was observed in the FAERS database for reports from the literature during the first quarter of 2021.[39] In specific drug-event pair analysis, duplicates accounted for 8% (287/3572) of clozapine-myocarditis cases in VigiBase from inception to January 2021 and for 20% (28/141) of quinine-induced thrombocytopenia cases in the FAERS from 1974 to 2000.[40, 41] In Africa, information was scarce on the extent of duplication in pharmacovigilance databases.

Causes of duplication: Duplicates in pharmacovigilance databases arise from various causes including: 1) data entry errors, e.g. i) submission of follow-up reports of the same case with new identification numbers,[4] ii) multiple entries of the same case by different data entrants,[21, 42] and iii) misspelling the name of a drug or patient;[21] 2) multiple reporters e.g. patients, healthcare professionals or pharmaceutical companies;[10, 22, 42, 43] 3) pressure from mandatory reporting requirements for healthcare professionals and pharmaceutical companies;[4, 24, 44, 45] 4) challenges in data integration and harmonization in database software, e.g. technical glitches that could occur during i) data synchronization, ii) data import/export, or iii) the integration of different databases;[26, 46] 5) multiple representation of the same data due to the merging of data from different databases such as regulatory authorities, pharmaceutical companies and clinical trials;[6] 6) lack of standardized data management practices characterized by inconsistencies in data entry standards and coding systems,[6] and 7) incomplete or missing case information of key data elements, e.g. patient identifiers.[25, 46, 47]

Strategies to minimize duplication: Several papers described best practices for minimizing duplication. These strategies include standardized data entry to enable consistent recording of adverse event data, making it easier to identify duplicates;[46, 48, 49] assigning unique identifiers to individual case safety reports, such as the worldwide unique case identification number, which should not be changed during electronic transmission and re-transmission of safety reports;[3, 6, 50] regular data cleaning and deduplication[48]; automated and semi-automated search criteria based on similarities in patient demographics (age, gender), unique identifiers, adverse reactions, and suspected medicines;[4, 6, 48, 51] data validation and quality assurance processes;[52, 53] collaboration between health facilities, pharmaceutical companies, and patients;[54, 55] and, data sharing between regulatory authorities and international databases.[54, 55]

For peer review only - http://bmjopen.bmj.com/site/about/guidelines.xhtml

Detection of duplicates: Twenty-four papers discussed the detection of duplicates. Pharmacovigilance databases should be reviewed routinely for duplicates and screening for duplicates should be a priority when new reports are submitted.[6, 56] Automated or semi-automated detection systems are recommended to identify duplicates during both manual and electronic data capture.[6, 48] To detect duplicates in small databases, reports may be sorted into simple tables by case ID numbers, patient characteristics (age, sex), adverse reaction(s) or suspected medicine(s). For large databases, however, the flagging of potential duplicates is largely automated, and is frequently followed up with manual review particularly to confirm inconclusive duplicates detected by the automated methods.[29] Duplicate detection algorithms should, however, be validated and fine-tuned periodically because the level of detail in submitted reports could vary with time, e.g. if data fields become mandatory, these variables ought to be included in the algorithms.[6, 26, 57]

Approaches to duplicate detection:

Exact matching: This is a straightforward method which compares two records to identify precise matches (exact duplicates) based on key data fields such as the report ID, patient demographics, name of adverse event, onset date of the adverse event, and name of suspected drug, among others.[58, 59]

Rule-based matching: This method defines specific conditions to identify and eliminate potential duplicates based on predefined criteria informed by domain knowledge, historical data and observed patterns in the data. It allows for more targeted identification of duplicates based on known patterns, e.g., the same case with the same adverse event reported several times in a short period.[60] These rules are typically based on particular fields in the data, e.g. patient's name, date of birth, or drug name, and are intended to match reports that have similar data.[61, 62] Simple duplicates can often be found using rule-based systems, but more complex duplicates may be missed because they require more complicated algorithms.[60]

Fuzzy matching: This technique identifies records that are similar though not necessarily identical. Unlike exact matching, which requires an exact match between data fields, fuzzy matching allows for variations, misspellings, and small differences in the data. This is particularly useful where the data may be inconsistently entered or contain errors, frequently in real-world databases.[63] Fuzzy matching algorithms assess the degree of resemblance between two records using a variety of similarity metrics, including Levenshtein distance and Jaro-Winkler distance. Fuzzy matching algorithms are frequently used in pharmacovigilance to identify duplicates that contain distinct patient names, medical terms, or drug names spelled differently or abbreviated differently.[63]

Machine learning and natural language processing: This approach employs supervised machine learning models to train algorithms using labelled data that differentiate duplicate and non-duplicate pairs. These models then use the knowledge gained from labelled data to predict whether new record pairs are duplicates or not based on learned patterns.[5, 64],[65, 66] Natural language processing techniques have been developed to augment computerised duplicate detection using the narrative in adverse event reports.[66] These models can be more precise than rule-based systems, particularly when working with large and complex datasets. The performance of machine learning models can be improved over time as they gain knowledge from fresh instances and adapt to changing data.[18, 55] Machine learning models are trained on extensive datasets of adverse event reports to identify patterns and connections between various factors, including drug names, patient demographics, and symptoms.[67] Once trained, these models can be used to automatically identify and categorize duplicates based on a variety of factors, including the similarity of medication names, the occurrence of specific medical conditions, and the timing and severity of symptoms.[68, 69] Machine learning models can be very accurate and efficient in finding complex duplicates; however, their creation and maintenance demands a significant quantity of high-quality training data and specialized knowledge.[70, 71]

Probabilistic record linkage: This method identifies and remove duplicate reports from large and complex datasets. An example is the hit-miss model which uses computational methods to analyze extensive healthcare

For peer review only - http://bmjopen.bmj.com/site/about/guidelines.xhtml

databases. It distinguishes "hits" (adverse events) from "misses" (non-events) by identifying patterns and signals.[3, 48] The method relies on statistical techniques to estimate the probability that two reports refer to the same event or individual, considering various data fields, such as patient name, age, and drug name. Probabilistic record linkage is particularly useful when dealing with datasets that are incomplete, inconsistent, or have missing data fields because they can still produce reliable results. However, this method can be computationally intensive and requires substantial computational resources, making it less practical for smaller datasets or less well-resourced pharmacovigilance systems.[4]
Manual review: This method is used to confirm reports that have been flagged as potential duplicates. Manual review is used in instances where automated methods are inconclusive.[6, 48]

Confirmation of duplicates: Manual confirmation is always necessary following the detection of potential duplicates. Well documented reports, with elaborate case narratives, make the confirmation process easier.[5, 64, 66] However, reports with limited information require that reporters are contacted for additional information, which necessitates the timely review of submitted reports.[6] When duplicates are detected, root cause analysis should be done and corrective action taken. Lastly, confirmed duplicates should be managed appropriately.[6]

Management of duplicates: This is the process of merging two or more reports to form one master report. Two approaches could be used: i) allocating an existing report as the master report, with the information from other reports being added unless the same or more precise information is already present in the master report, and ii) creating a master report by combining the information from several duplicate reports.[6, 21] The master report should always include the case reference numbers of all subordinate duplicate reports for easy traceability. Subordinate duplicates remain in the database for the purpose of audit trail but are not used for any other pharmacovigilance purpose. One challenge with duplicate management is when conflicting information is provided by different reporters. Clarification of conflicting information should be obtained wherever possible.[6]

Software programs: These are valuable tools for deduplication in pharmacovigilance databases. VigiMatch is a software program developed by the Uppsala Monitoring Centre to detect potential duplicates in the global database of adverse event reports, VigiBase.[26, 72] VigiMatch compares various data elements of adverse event reports and assigns a matching score to each pair of reports.[4] Although VigiMatch and VigiBase are separate programs, they are linked in the sense that VigiMatch is often used as a tool to identify and consolidate potential duplicate reports within VigiBase. VigiBase includes data cleaning and deduplication tools that use various criteria to identify potential duplicates, including patient information, drug information, and adverse event descriptions. VigiBase can merge duplicate reports identified by VigiMatch, ensuring that the database remains accurate and efficient.[48] VigiMatch is available for use by national pharmacovigilance centers and regulatory authorities worldwide. However, access to the software depends on various factors, such as the availability of funding, infrastructure, and technical expertise to effectively implement the software.[72]

Other software packages have been developed. The Medical Dictionary for Regulatory Activities (MedDRA) coding system is a standardized software for regulatory reporting that can identify duplicates and minimize data entry errors.[73] ArisGlobal's LifeSphere is a cloud-based pharmacovigilance software with a deduplication module to identify and consolidate duplicate reports.[74] Oracle's Argus Safety is a comprehensive safety management system with a deduplication feature to eliminate duplicate reports and improve data accuracy.[75] Ablebits Ultimate Suite of tools is software with a variety of *add-ins* and *plugins* for Microsoft Office Excel and Google Sheets. The software has a range of tools, including the "Remove Duplicates" feature that allows users to find and eliminate duplicate entries in a dataset. The primary purpose of Ablebits is to enhance the functionality of spreadsheet programs and make it easier for users to perform

For peer review only - http://bmjopen.bmj.com/site/about/guidelines.xhtml

various tasks. Ablebits offers a range of tools that can automate repetitive tasks, format data, merge cells, and compare data, etc. The software can also merge duplicate reports to create a single case record, thereby reducing manual review and data entry. The goal of Ablebits is to help users save time and improve their productivity while working with Excel or Google Sheets.[76] Open source software for data deduplication, e.g, DataCleaner Extension, can be used where Excel has limits.[77]

Advantages and limitations of software programs: The **advantages** of using software programs for deduplication include improved efficiency, accuracy, and consistency in identifying and managing duplicates. These programs can also handle large volumes of data and can be customized to meet specific regulatory requirements.[49] Using Excel with the Ablebits Ultimate Suite of tools could be more cost-effective for small datasets – the software is accessible and easy to use particularly if the user is familiar with Excel. However, Ablebits may not be as efficient or accurate as the dedicated software programs, particularly for large and complex pharmacovigilance databases.

However, there are also **limitations** to using software programs for deduplication. These programs may require extensive training and expertise to operate effectively, and might not detect all potential duplicates owing to variations in data entry or differences in data elements used for comparison. Additionally, the cost of implementing and maintaining these software programs may be a barrier for some regulatory authorities, especially in low- and middle-income countries. Artificial intelligence algorithms need to be trained with large quantities of high-quality data. The technical challenges of artificial intelligence-based pharmacovigilance, particularly in low- and middle-income countries, are the lack of high-quality databases, insufficient human resources, weak artificial intelligence technology, data sharing and privacy challenges, transparency of algorithms, interoperability across multiple platforms and insufficient support from governments.[28, 33, 71, 78-80]

Impact of duplication:

Inaccurate risk assessment: Duplicates in pharmacovigilance databases can lead to overestimation or underestimation of the frequency and severity of adverse events, introduce inconsistencies in data analysis and reporting, distort the understanding of the safety profile of medicines, and hinder signal detection and risk assessment. This compromises the integrity and reliability of databases as sources of accurate information, and potentially impacts important decisions related to drug labelling, risk management, and patient safety.[3, 4, 6, 7, 13, 21, 48, 64] As an example, Brinker et al.[41] observed an exaggerated safety signal of quinine-induced thrombocytopenia as a result of duplicate reports.

Resource wastage: Duplicates consume valuable database storage space, computing resources, and human effort. Maintaining and managing duplicate records uses up resources, which could be better utilized for other critical tasks.[4, 7, 77, 81] Having duplicate entries necessitates additional manual effort to identify and resolve them. Pharmacovigilance staff need to spend more time on data cleaning and deduplication processes, which diverts their attention from other essential activities.[3]

Delays in data analysis: Sorting through duplicate entries to identify and remove them can cause delays in data analysis and signal detection. Timely identification and response to safety concerns may be hindered due to the time-consuming nature of duplicate management.[3]

Regulatory compliance challenges: Duplicates can complicate regulatory reporting and compliance obligations. Thus, submitting accurate and non-duplicated data to regulatory authorities, may become more difficult and time-consuming, particularly for pharmaceutical companies.[6]

For peer review only - http://bmjopen.bmj.com/site/about/guidelines.xhtml

DISCUSSION

This scoping review sought to evaluate the extent, causes, preventive strategies, detection methods, management, and impact of duplication in pharmacovigilance databases worldwide. The extent of duplication in pharmacovigilance databases varied across different databases. The WHO global database, VigiBase, exhibited a 2.5% prevalence of duplication for reports submitted from 2000 to 2010, based on only half of the reports with sufficient information to conduct duplicate analysis.[4] In a global context, despite that this prevalence rate is relatively low, it represents about 750,000 duplicates among the 30+ million reports in VigiBase as of 2023.[82] Duplication rates in VigiBase were highest for reports from the published literature, e.g., peer-reviewed articles (11%), and fatal outcomes (5%);[4] and could be due to the heightened awareness and documentation of certain types of adverse events, particularly serious outcomes and those of special interest.[4, 39] These prevalence rates in VigiBase should, however, be interpreted with caution since half the reports were excluded from duplicate analysis due to missing data.[4] Duplication was more extreme (66% to 87%) for individual case safety reports from the published literature in the United States' FAERS database, and is almost entirely attributable to the replication of case reports due to the obligation to report individual case safety reports from the literature as enforced on pharmaceutical companies by Competent Authorities.[39] Thus, replication of case reports is an adjunct to duplication and is largely induced by regulatory reporting obligations, contractual agreements, and partnerships leading to the creation of case replicas.[7] The resolution of replication will require significant care and possibly redesigning pharmacovigilance data management systems at the global level.

Duplication rates in VigiBase varied across countries, being high for Czech Republic (15%), Austria (15%), Korea (9.2%) and Switzerland (4.7%), and much lower for United Kingdom (1.4%), Denmark (1%), Spain (0.7%) and Japan (8%), among others.[4] Thus, special attention to data quality is required when integrating pharmacovigilance datasets from different countries with varying risks of duplication. Also, regional variations call for standardized deduplication strategies to ensure consistency and data reliability across different settings.

Duplication in pharmacovigilance databases can arise from data entry errors, multiple reporters, mandatory reporting requirements, technical software glitches, and incomplete case information. This is compounded by the growing volume of pharmacovigilance reports worldwide, particularly due to the large number of adverse event reports following mass immunization with COVID-19 vaccines.[83] Thus, the possibility of duplicates cannot be ruled out, and it is crucial to develop robust and efficient systems to minimize them.[5] Understanding the causes of duplication is important to identify and institute effective measures to prevent them. Such measures could reduce the occurrence of duplication, promote coordinated approaches to deduplication, and enhance data quality and consistency. Standardized data entry and the assignment of unique identifiers to individual case safety reports is essential for consistent recording of adverse event data, making it easier to minimise and manage duplicates. The International Council for Harmonization of Technical Requirements for Pharmaceuticals for Human Use (ICH) has adopted the ICH E2B(R3) messaging standard for electronic transmission of individual case safety reports.[50] This standard stipulates the use of a worldwide unique case identification number (Section C.1.8.1) for each safety report which should not be changed during the transmission and subsequent re-transmission of safety reports.[50] This single field becomes the primary key for case identification, and is therefore a practical solution to effectively reduce and manage duplication. Regular automated and semi-automated data deduplication mechanisms, leveraging artificial intelligence, are among the array of measures at various stages of deployment across the United States, Europe, and Asia.[4, 6, 48, 51] However, there is notable scanty information from Africa. The effectiveness of deduplication methods will require continuous improvement, with time, to be in tandem with the submission of new reports to the pharmacovigilance databases.[5, 48, 64]

For peer review only - http://bmjopen.bmj.com/site/about/guidelines.xhtml

To enhance the accuracy of pharmacovigilance databases, newly submitted adverse event reports should be screened for duplicates. This proactive approach to data entry ensures superior data quality compared to relying solely on post-entry data cleaning processes.[6] Approaches to duplicate detection range from manual review to automated methods such as exact matching, rule-based matching, fuzzy matching, probabilistic record linkage and artificial intelligence. Probabilistic record linkage is effective for dealing with incomplete or inconsistent data but demands computational resources.[4] Artificial intelligence models based on machine learning provide more precise and efficient means of identifying duplicates, especially in large and complex datasets.[18, 55] Natural language processing techniques which use the narrative in adverse event reports have also been developed to augment duplicate detection using machine learning. These advances in natural language processing hold promise in enhancing the efficiency and precision of the deduplication of large and complex pharmacovigilance databases.[66] Automated detection systems are the recommended methods for large and complex databases.[5]·[31, 32] However, manual review is necessary for inconclusive potential duplicates from automated systems.[6, 29, 48] The duplicate detection algorithms of automated systems require routine fine-tuning to adapt them to changing data requirements, ensuring database accuracy.[18, 55]

Software programs for the deduplication of pharmacovigilance databases significantly enhance the quality of pharmacovigilance data and ultimately improve patient safety. Dedicated software programs for duplicate management are suitable for very large databases and might not be cost-effective for small databases, particularly in LMIC.[26, 73-75] Additional challenges for implementing dedicated deduplication software in LMIC include poor data quality, limited resources and infrastructure, inadequate training and capacity building, a lack of harmonization and standardization across regulatory systems, and limited access to data and information. These gaps can hinder the ability of regulatory authorities in LMIC to effectively monitor and assess drug safety and efficacy, and the impact of duplication on pharmacovigilance databases.[79] Alternative deduplication software for small datasets in LMIC include the Ablebits Ultimate Suite of tools with *add-ins* for Microsoft Office Excel. Using Ablebits in addition to manual review for small datasets could be cost-effective and possibly improves the efficiency and accuracy of deduplication.[76] However, open source software solutions, e.g., DataCleaner Extension, have superior capabilities to the Ablebits Excel *add-in*.[77]

Duplicates have consequences for pharmacovigilance databases such as the inaccurate risk assessment which could lead to overestimation or underestimation of adverse event frequencies. This could, in turn, distort the understanding of a drug's safety profile and hinder signal detection.[3-5] Resource wastage, increased workload for pharmacovigilance staff, delays in data analysis, and regulatory compliance challenges are additional consequences of duplication.[3, 4, 6, 7, 77, 81] These consequences underscore the importance of addressing duplication to promote the integrity and effectiveness of pharmacovigilance systems. We therefore make the following recommendations:

- We strongly advocate for the consistent use of worldwide unique identifiers (field C.1.8.1 within the ICH E2B(R3) guideline) as the primary key for case identification to minimise case duplication
- Scale up reporting methods that minimise incomplete case information, e.g., electronic systems that enforce the obligatory inclusion of key case details at the time of reporting
- Increase investment in pharmacovigilance infrastructure and capacity building
- Improve collaboration and data sharing among regulatory authorities and industry partners
- Put more emphasis on the harmonization and standardization of regulatory systems worldwide
- Develop and implement innovative technologies such as machine learning and natural language processing to improve the efficiency and accuracy of drug safety monitoring, particularly in LMIC.

For peer review only - http://bmjopen.bmj.com/site/about/guidelines.xhtml

- Low- and middle-income countries with small pharmacovigilance datasets ought to conduct pilot studies to explore the feasibility of alternative low-cost technologies for data deduplication (e.g., Ablebits Suite of tools in Microsoft Office Excel and the open source DataCleaner Extension).

CONCLUSION

This scoping review provides a comprehensive overview of duplication in pharmacovigilance databases worldwide. It highlights the need for standardized practices, advanced detection methods, and collaborative efforts to minimize duplication and enhance the accuracy and reliability of drug safety data. Addressing duplication is crucial to supporting evidence-based decision-making in healthcare and drug regulation, ensuring patient safety. Software programs are commonly used for deduplication in pharmacovigilance databases and they significantly enhance the quality of pharmacovigilance data and ultimately improve patient safety. Overall, software programs offer advantages in terms of efficiency and accuracy, although there are limitations. For smaller datasets, Microsoft Office Excel with Ablebits Ultimate Suite may be cost-effective, and ought to be piloted, whereas larger and more complex datasets require dedicated software programs.

STATEMENTS AND DECLARATIONS

Funding

This study was funded by the Medical Research Council (MR/V03510X/1).

Conflicts of interest statement

Ronald Kiguba and Munir Pirmohamed obtained funding from the Medical Research Council; however, the funder had no role in drafting this manuscript. Ronald Kiguba, Gerald Isabirye, Julius Mayengo, Jonathan Owiny, Phil Tregunno, Kendal Harrison and Helen B. Ndagije declare that they have no conflicts of interest. Munir Pirmohamed has received partnership funding for the following: Medical Research Council Clinical Pharmacology Training Scheme (co-funded by Medical Research Council and Roche, UCB, Eli Lilly and Novartis). He also has unrestricted educational grant support for the UK Pharmacogenetics and Stratified Medicine Network from Bristol-Myers Squibb. He has developed an HLA genotyping panel with MC Diagnostics, but does not benefit financially from this. He is part of the IMI Consortium ARDAT (www.ardat.org). None of this funding MP received is related to the current paper.

Ethical approval

This review did not involve humans as study participants therefore ethical approval was not required.

Author's contributions

RK and GI conceptualized the study, collected the data, performed data curation, conducted formal analysis and data interpretation, and developed the first draft of the manuscript. JM, JO, PT, KH, MP and HBN contributed to the study design, analysis and interpretation of the data. All authors read and approved the final draft of the manuscript.

Acknowledgements

We are grateful to the National Drug Authority - Uganda, particularly the National Pharmacovigilance Centre, which supported the situation analysis of duplication in pharmacovigilance databases worldwide.

Data Sharing Statement

All data are included in the manuscript or uploaded as supplementary material.

For peer review only - http://bmjopen.bmj.com/site/about/guidelines.xhtml

References

1. World Health Organization. *The importance of pharmacovigilance*. Safety monitoring of medicinal products 2002 [cited 2023 28 August]; Available from: https://apps.who.int/iris/bitstream/handle/10665/42493/a75646.pdf.
2. World Health Organization. *Pharmacovigilance*. Regulation and Prequalification [cited 2023 13 September]; Available from: https://www.who.int/teams/regulation-prequalification/regulation-and-safety/pharmacovigilance.
3. Norén, G.N., et al., *Duplicate detection in adverse drug reaction surveillance.* Data Mining and Knowledge Discovery, 2007. **14**(3): p. 305-328.
4. Tregunno, P.M., et al., *Performance of probabilistic method to detect duplicate individual case safety reports.* Drug Saf, 2014. **37**(4): p. 249-58.
5. Norén, G.N., *The Power of the Case Narrative - Can it be Brought to Bear on Duplicate Detection?* Drug Saf, 2017. **40**(7): p. 543-546.
6. European Medicines Agency and Heads of Medicines Agencies. *Guideline on good pharmacovigilance practices (GVP) Module VI Addendum I – Duplicate management of suspected adverse reaction reports*. 2017 [cited 2023 07 August]; Available from: https://www.ema.europa.eu/en/documents/regulatory-procedural-guideline/guideline-good-pharmacovigilance-practices-gvp-module-vi-addendum-i-duplicate-management-suspected_en.pdf.
7. van Stekelenborg, J., et al., *Individual Case Safety Report Replication: An Analysis of Case Reporting Transmission Networks.* Drug Saf, 2023. **46**(1): p. 39-52.
8. European Medicines Agency. *Mapping of pharmacovigilance initiatives*. [cited 2023 13 September]; Available from: https://www.ema.europa.eu/en/documents/other/icmra-mapping-pharmacovigilance-initiatives_en.pdf.
9. Shankar, P.R., *Website review: The pharmacovigilance toolkit.* J Pharmacol Pharmacother, 2013. **4**(4): p. 307-8.
10. Council for International Organizations of Medical Sciences (CIOMS). *Practical Aspects of Signal Detection in Pharmacovigilance*. Report of CIOMS Working Group VIII, 2010 [cited 2023 21 September]; Available from: https://cioms.ch/wp-content/uploads/2018/03/WG8-Signal-Detection.pdf.
11. Council for International Organizations of Medical Sciences (CIOMS). *Making medicines safer for patients everywhere*. 2021 [cited 2023 21 September]; Available from: https://cioms.ch/wp-content/uploads/2021/11/CIOMS_SpecialNewsletter_UMC-9Nov2021.pdf.
12. United States (US) Food and Drug Administration (FDA) Adverse Event Reporting System (FAERS). *Questions and Answers on FDA's Adverse Event Reporting System (FAERS)*. 2018 [cited 2023 09 September]; Available from: https://www.fda.gov/drugs/surveillance/questions-and-answers-fdas-adverse-event-reporting-system-faers.
13. Hauben, M., et al., *'Extreme duplication' in the US FDA Adverse Events Reporting System database.* Drug Saf, 2007. **30**(6): p. 551-4.
14. European Medicines Agency. *EudraVigilance*. Human regulatory 2011 [cited 2023 29 August]; Available from: https://www.ema.europa.eu/en/human-regulatory/research-development/pharmacovigilance/eudravigilance.
15. Oliveira, J.L., et al., *The EU-ADR Web Platform: delivering advanced pharmacovigilance tools.* Pharmacoepidemiol Drug Saf, 2013. **22**(5): p. 459-67.
16. Nwokike, J. and K. Eghan. *Pharmacovigilance in Ghana: A Systems Analysis*. 2010 [cited 2023 29 August]; Available from: https://pdf.usaid.gov/pdf_docs/pnadu524.pdf.
17. Asiamah, M., et al., *Spontaneous reporting of adverse drug reaction among health professionals in Ghana.* Arch Public Health, 2022. **80**(1): p. 33.
18. Lavertu, A., et al., *A New Era in Pharmacovigilance: Toward Real-World Data and Digital Monitoring.* Clin Pharmacol Ther, 2021. **109**(5): p. 1197-1202.
19. Martin-Sanchez, F. and K. Verspoor, *Big data in medicine is driving big changes.* Yearb Med Inform, 2014. **9**(1): p. 14-20.

For peer review only - http://bmjopen.bmj.com/site/about/guidelines.xhtml

20. ECRI Institute. *Wrong-Record, Wrong-Data Errors with Health IT Systems*. 2015 [cited 2023 29 August]; Available from: https://www.ecri.org/Resources/In_the_News/PSONavigator_Data_Errors_in_Health_IT_Systems.pdf.
21. Ramya. *Duplicate search in pharmacovigilance*. 2019 [cited 2023 24 August]; Available from: https://allaboutpharmacovigilance.org/15-duplicate-search-in-pharmacovigilance/.
22. Pozsgai, K., et al., *Analysis of pharmacovigilance databases for spontaneous reports of adverse drug reactions related to substandard and falsified medical products: A descriptive study.* 2022. **13**.
23. Harpaz, R., H.S. Chase, and C. Friedman, *Mining multi-item drug adverse effect associations in spontaneous reporting systems.* BMC Bioinformatics, 2010. **11**(9): p. S7.
24. Uppsala Monitoring Centre. *Text is key for eliminating duplicate reports*. 2017 [cited 2023 29 August]; Available from: https://www.uppsalareports.org/articles/text-is-key-for-eliminating-duplicate-reports/.
25. Just, B.H., et al., *Why Patient Matching Is a Challenge: Research on Master Patient Index (MPI) Data Discrepancies in Key Identifying Fields.* Perspect Health Inf Manag, 2016. **13**(Spring): p. 1e.
26. Uppsala Monitoring Centre (UMC). *Sharing pharmacovigilance data in the WHO Programme for International Drug Monitoring* Reporting fact sheet [cited 2023 13 September]; Available from: https://who-umc.org/media/3236/reporting-fact-sheet.pdf.
27. Amedome SN and Dadson BA, *Pharmacovigilance Practices: Knowledge and Attitudes among the Healthcare Professionals at the Volta Regional Hospital of Ghana.* Journal of Pharmacovigilance, 2017. **5**(3).
28. Ghosh, R., et al., *Automation Opportunities in Pharmacovigilance: An Industry Survey.* Pharmaceut Med, 2020. **34**(1): p. 7-18.
29. Muñoz, M.A., et al., *Towards Automating Adverse Event Review: A Prediction Model for Case Report Utility.* Drug Saf, 2020. **43**(4): p. 329-338.
30. Gandomi, A. and M. Haider, *Beyond the hype: Big data concepts, methods, and analytics.* International Journal of Information Management, 2015. **35**(2): p. 137-144.
31. Lewis, D.J. and J.F. McCallum, *Utilizing Advanced Technologies to Augment Pharmacovigilance Systems: Challenges and Opportunities.* Ther Innov Regul Sci, 2020. **54**(4): p. 888-899.
32. Luo, Y., et al., *Natural Language Processing for EHR-Based Pharmacovigilance: A Structured Review.* Drug Saf, 2017. **40**(11): p. 1075-1089.
33. Hauben, M., *Artificial Intelligence and Data Mining for the Pharmacovigilance of Drug-Drug Interactions.* Clinical Therapeutics, 2023. **45**(2): p. 117-133.
34. Gosselt, H.R., et al., *Development of a multivariate prediction model to identify individual case safety reports which require clinical review.* Pharmacoepidemiol Drug Saf, 2022. **31**(12): p. 1300-1307.
35. Peters, M.D., et al., *Guidance for conducting systematic scoping reviews.* Int J Evid Based Healthc, 2015. **13**(3): p. 141-6.
36. Tricco, A.C., et al., *PRISMA Extension for Scoping Reviews (PRISMA-ScR): Checklist and Explanation.* Ann Intern Med, 2018. **169**(7): p. 467-473.
37. Clark, J.M., et al., *Improving the translation of search strategies using the Polyglot Search Translator: a randomized controlled trial.* J Med Libr Assoc, 2020. **108**(2): p. 195-207.
38. Canadian Agency for Drugs and Technologies in Health. *Grey matters: a practical tool for searching health-related grey literature.* 2023 [cited 2023 22 September]; Available from: https://greymatters.cadth.ca/.
39. Hung, E., et al., *More extreme duplication in FDA Adverse Event Reporting System detected by literature reference normalization and fuzzy string matching.* Pharmacoepidemiol Drug Saf, 2023. **32**(3): p. 387-391.
40. De Las Cuevas, C., et al., *Clozapine-associated myocarditis in the World Health Organization's pharmacovigilance database: Focus on reports from various countries.* Rev Psiquiatr Salud Ment (Engl Ed), 2022. **15**(4): p. 238-250.
41. Brinker, A.D. and J. Beitz, *Spontaneous reports of thrombocytopenia in association with quinine: clinical attributes and timing related to regulatory action.* Am J Hematol, 2002. **70**(4): p. 313-7.
42. European Medicines Agency. *Guideline on good pharmacovigilance practices (GVP) Module IX Addendum I – Methodological aspects of signal detection from spontaneous reports of suspected adverse reactions*. Module IX Addendum I – Methodological aspects of signal detection from spontaneous reports of suspected adverse reactions 2017 [cited 2023 07 August]; Available from: https://www.ema.europa.eu/en/documents/scientific-

For peer review only - http://bmjopen.bmj.com/site/about/guidelines.xhtml

guideline/guideline-good-pharmacovigilance-practices-gvp-module-ix-addendum-i-methodological-aspects-signal_en.pdf.
43. Banovac, M., et al., *Patient Reporting in the EU: Analysis of EudraVigilance Data.* Drug Saf, 2017. **40**(7): p. 629-645.
44. Candore, G., et al., *The Impact of Mandatory Reporting of Non-Serious Safety Reports to EudraVigilance on the Detection of Adverse Reactions.* Drug Saf, 2022. **45**(1): p. 83-95.
45. Lasys, T., et al., *Unintended impact of pharmacovigilance regulatory interventions: A systematic review.* Br J Clin Pharmacol, 2023.
46. Kahn, M.G., et al., *A Harmonized Data Quality Assessment Terminology and Framework for the Secondary Use of Electronic Health Record Data.* EGEMS (Wash DC), 2016. **4**(1): p. 1244.
47. US Food & Drug Administration. *Questions and Answers on FDA's Adverse Event Reporting System (FAERS).* 2018 [cited 2023 29 Augsut]; Available from: https://www.fda.gov/drugs/surveillance/questions-and-answers-fdas-adverse-event-reporting-system-faers.
48. Agency for Medicinal Products and Medical Devices of Croatia (HALMED). *Strengthening Collaboration for Operating Pharmacovigilance in Europe (SCOPE).* Duplicate Detection: Best Practice Guide [cited 2023 24 August]; Available from: https://www.ema.europa.eu/en/documents/other/scope-training-duplicate-detection-best-practice-guide_en.pdf
49. Richesson, R.L., et al., *An automated standardized system for managing adverse events in clinical research networks.* Drug Saf, 2008. **31**(10): p. 807-22.
50. European Medicines Agency. *ICH guideline E2B (R3) on electronic transmission of individual case safety reports (ICSRs) - data elements and message specification - implementation guide*. 2013 [cited 2024 11 March]; Available from: https://www.ema.europa.eu/en/documents/scientific-guideline/international-conference-harmonisation-technical-requirements-registration-pharmaceuticals-human-use-guideline-e2b-r3-electronic-transmission-individual-case-safety-reports-icsrs-data-elements_en.pdf.
51. Mockute, R., et al., *Artificial Intelligence Within Pharmacovigilance: A Means to Identify Cognitive Services and the Framework for Their Validation.* Pharmaceut Med, 2019. **33**(2): p. 109-120.
52. Bhangale, R., S. Vaity, and N. Kulkarni, *A day in the life of a pharmacovigilance case processor.* Perspect Clin Res, 2017. **8**(4): p. 192-195.
53. Paygude, P. and P.J.I.J.M.E.R. Devale, *Automated data validation testing tool for data migration quality assurance.* 2013. **3**(1): p. 599-603.
54. Postigo, R., et al., *EudraVigilance Medicines Safety Database: Publicly Accessible Data for Research and Public Health Protection.* Drug Saf, 2018. **41**(7): p. 665-675.
55. Wong, J., et al., *Applying Machine Learning in Distributed Data Networks for Pharmacoepidemiologic and Pharmacovigilance Studies: Opportunities, Challenges, and Considerations.* Drug Saf, 2022. **45**(5): p. 493-510.
56. Lindquist, M., *Data quality management in pharmacovigilance.* Drug Saf, 2004. **27**(12): p. 857-70.
57. Wadhwa, D., et al., *Automation in signal management in pharmacovigilance-an insight.* Brief Bioinform, 2021. **22**(4).
58. Hakak, S., et al., *A new split based searching for exact pattern matching for natural texts.* PLoS One, 2018. **13**(7): p. e0200912.
59. Christen, P., *Data matching: concepts and techniques for record linkage, entity resolution, and duplicate detection*. 2012, Springer: Data-centric systems and applications.
60. Liu H, Gegov A, and Stahl F. *Categorization and Construction of Rule Based Systems* 2014 [cited 2023 29 August]; Available from: https://researchportal.port.ac.uk/files/1938654/EANN2014_038.pdf.
61. Wong, C.K., et al., *Standardisation of the FAERS database: a systematic approach to manually recoding drug name variants.* Pharmacoepidemiology and drug safety, 2015. **24**(7): p. 731-737.
62. Grigoriev, I., et al., *AERS spider: an online interactive tool to mine statistical associations in Adverse Event Reporting System.* Pharmacoepidemiol Drug Saf, 2014. **23**(8): p. 795-801.
63. Yang, J.D., *F_MP: A fuzzy match framework for rule-based programming.* Data & Knowledge Engineering, 1997. **24**(2): p. 183-203.

For peer review only - http://bmjopen.bmj.com/site/about/guidelines.xhtml

64. Kreimeyer, K., et al., *Increased Confidence in Deduplication of Drug Safety Reports with Natural Language Processing of Narratives at the US Food and Drug Administration.* 2022. **2**.
65. Schmider, J., et al., *Innovation in Pharmacovigilance: Use of Artificial Intelligence in Adverse Event Case Processing.* Clin Pharmacol Ther, 2019. **105**(4): p. 954-961.
66. Kreimeyer, K., et al., *Using Probabilistic Record Linkage of Structured and Unstructured Data to Identify Duplicate Cases in Spontaneous Adverse Event Reporting Systems.* Drug Saf, 2017. **40**(7): p. 571-582.
67. Harpaz, R., et al., *Toward multimodal signal detection of adverse drug reactions.* J Biomed Inform, 2017. **76**: p. 41-49.
68. Kreimeyer, K., et al., *Feature engineering and machine learning for causality assessment in pharmacovigilance: Lessons learned from application to the FDA Adverse Event Reporting System.* Computers in Biology and Medicine, 2021. **135**: p. 104517.
69. Fusaroli, M., et al., *Development of a Network-Based Signal Detection Tool: The COVID-19 Adversome in the FDA Adverse Event Reporting System.* Front Pharmacol, 2021. **12**: p. 740707.
70. Kim, H.R., et al., *Analyzing adverse drug reaction using statistical and machine learning methods: A systematic review.* Medicine (Baltimore), 2022. **101**(25): p. e29387.
71. Kompa, B., et al., *Artificial Intelligence Based on Machine Learning in Pharmacovigilance: A Scoping Review.* Drug Saf, 2022. **45**(5): p. 477-491.
72. Council for International Organizations of Medical Sciences (CIOMS). *Making medicines safer for patients everywhere*. 2021 [cited 2023 29 August]; Available from: https://cioms.ch/wp-content/uploads/2021/11/CIOMS_SpecialNewsletter_UMC-9Nov2021.pdf.
73. MedDRA. *History*. [cited 2023 03 October]; Available from: https://www.meddra.org/about-meddra/history.
74. ArisGlobal. *ArisGlobal Announces LifeSphere® MultiVigilance 10, Industry's First End-to-End Safety System With Production-Ready Automation*. 2019 [cited 2023 09 September]; Available from: https://www.arisglobal.com/press-release/lifesphere-multivigilance-10-first-safety-system-production-ready-automation/.
75. Oracle. *Trusted safety case management*. 2021 [cited 2023 03 October]; Available from: https://www.oracle.com/a/ocom/docs/argus-trusted-safety-case-management.pdf.
76. Ablebits. *Ablebits Ultimate Suite*. 2023 [cited 2023 03 October]; Available from: https://www.ablebits.com/.
77. Azeroual, O., et al., *A Record Linkage-Based Data Deduplication Framework with DataCleaner Extension.* 2022. **6**(4): p. 27.
78. Liang, L., et al., *Artificial Intelligence-Based Pharmacovigilance in the Setting of Limited Resources.* Drug Safety, 2022. **45**(5): p. 511-519.
79. Kiguba, R., S. Olsson, and C. Waitt, *Pharmacovigilance in low- and middle-income countries: A review with particular focus on Africa.* Br J Clin Pharmacol, 2023. **89**(2): p. 491-509.
80. He, J., et al., *The practical implementation of artificial intelligence technologies in medicine.* Nature Medicine, 2019. **25**(1): p. 30-36.
81. Rathbone, J., et al., *Better duplicate detection for systematic reviewers: evaluation of Systematic Review Assistant-Deduplication Module.* Syst Rev, 2015. **4**(1): p. 6.
82. Uppsala Monitoring Centre. *What is VigiBase?* 2023 [cited 2023 16 October]; Available from: https://who-umc.org/vigibase/.
83. van der Boor, S.C., et al., *Spontaneously reported adverse events following COVID-19 basic and booster immunizations in the Netherlands.* Vaccine, 2023. **41**(29): p. 4319-4326.

For peer review only - http://bmjopen.bmj.com/site/about/guidelines.xhtml

Table 1: Summary of screening process

Screening Process	Number of Papers
Initial Screening	22745
Full Text Review	156
Eligibility Criteria	
- Met Criteria?	
- Yes	58
- No	98
Specific Categories*	
- Extent of Duplicate	5
- Prevention Strategies	15
- Causes of Duplicates	13
- Methods of Duplicate Detection	32
- Management Strategies	24
- Implications of Duplicates	14

* The papers overlapped, discussing more than one field

Figure 1: PRISMA flow diagram used to select papers for the scoping review

For peer review only

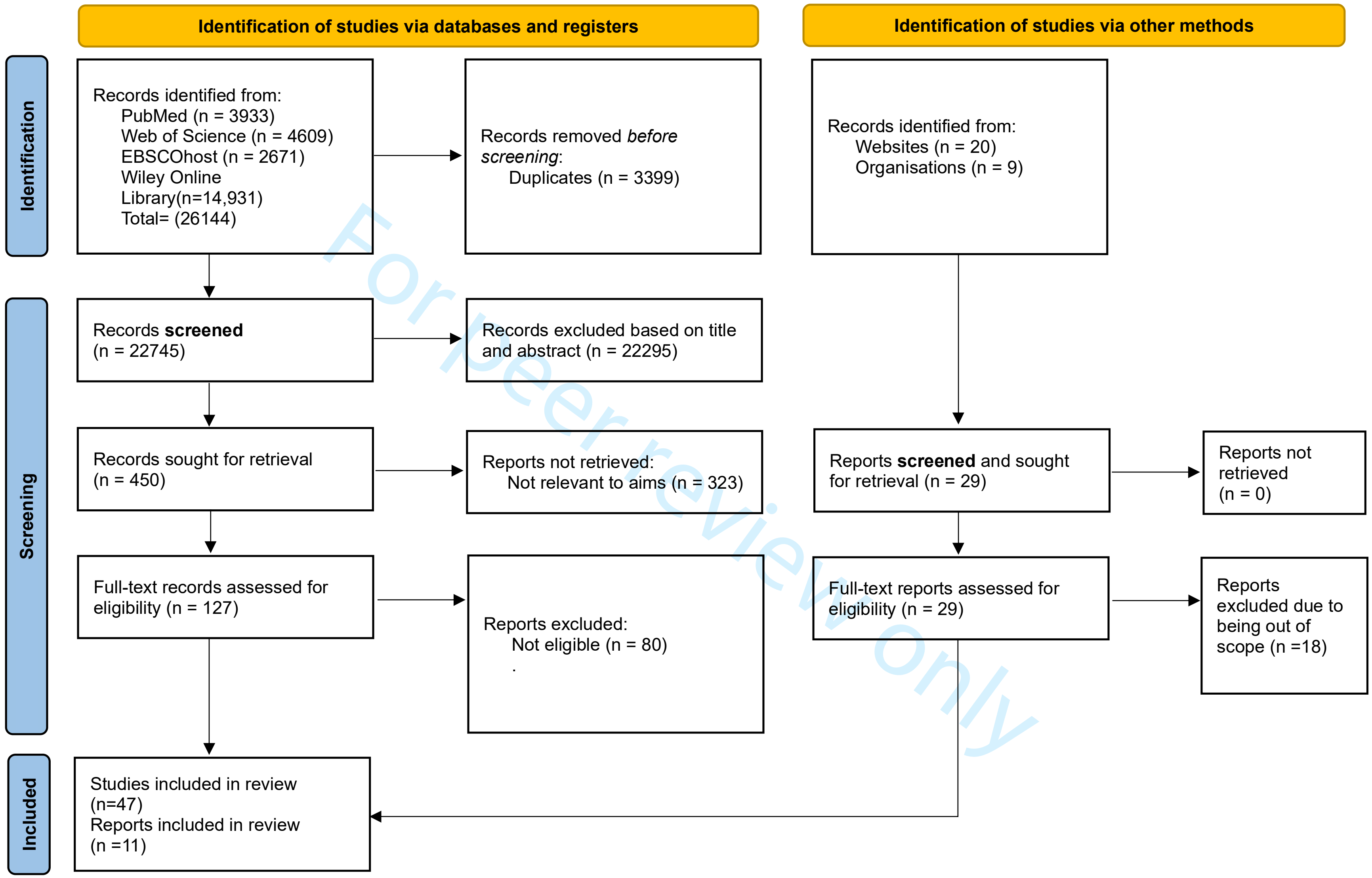

Figure 1: *PRISMA flow diagram used to select papers for the scoping review*

For peer review only

Supplementary Table 1: List of 58 papers included in the scoping review.

No.	Author(s)	Year of publication	Journal	Country	Title	Specific Objectives	Study design	Categories						Key findings
								Extent	Prevention	Causes	Detection	Management	Implications	
1	Norén GN	2017	Drug Safety	Sweden	The Power of the Case Narrative - Can it be Brought to Bear on Duplicate Detection?	To discuss the power of the case narrative in duplicate detection.	Commentary				✓			Kreimeyer et al. primarily focus on detecting duplicates in a specific drug or vaccine at a time, with examples in their study typically involving around 1000 reports each. Their research demonstrates the potential value of automated duplicate detection in this context. Manual identification becomes challenging, even with moderately sized case series, with over 400 possible pairs among 30 reports and 5000 pairs among 100 reports. Additionally, not all duplicates are easily identifiable by human assessors, as evidenced by instances of false positives where record pairs initially assumed to be algorithmic errors were later found to be genuine duplicates that had been overlooked.
2	Ramya	2019		Georgia	Duplicate search in pharmacovigilance	To define duplicates, identify their causes, address their handling, confirm their existence, and manage their occurrence.	Web page			✓		✓	✓	The detection and handling of duplicates by National Competent Authorities, Marketing Authorization Holders, and sponsors of clinical trials is an important element of good case management.
3	Likeng Liang *et al*.	2022	Drug Safety	China	Artificial Intelligence-Based Pharmacovigilance in the Setting of Limited Resources.	To review the possible solutions and future perspectives on AI-based pharmacovigilance in resource-limited settings.	Review article					✓		Artificial intelligence (AI) algorithms can process and analyse data relevant to pharmacovigilance. However, the key challenge lies in the initial training of AI algorithms with substantial amounts of high-quality data. Resource-limited settings face notable technical obstacles for AI-driven pharmacovigilance, including the scarcity of robust databases, inadequate human resources, underdeveloped AI technology, and insufficient governmental support. Enhancing the detection capabilities of AI-based pharmacovigilance through improved training and education, along with advocating for the advantages of AI-based approaches to governments, offers a pathway to addressing these challenges in settings constrained by resources. Future advancements in AI-based pharmacovigilance within resource-limited contexts are anticipated to be facilitated by collaborative research networks, advancements in pharmacogenomic research and practices, and the integration of sophisticated machine-learning algorithms. It is imperative to tailor these efforts to the specific contexts of such settings.
4	Manfred Hauben *et al*.	2023	Clinical Therapeutics.	USA	Artificial Intelligence and Data Mining for the Pharmacovigilance of Drug-Drug Interactions.	Despite increasing mechanistic understanding, undetected and underrecognized drug–drug interactions (DDIs) persist. The present article is intended to be part didactic primer and part narrative review of real-world and experimental deployment of AI and data mining activities for pharmacovigilance of DDIs.	Review article					✓		Artificial Intelligence and Data Mining for the Pharmacovigilance of Drug-Drug Interactions. The wide array of intricate and elegant methods has expanded the pharmacovigilance tool kit.

For peer review only

No.	Author(s)	Year of publication	Journal	Country	Title	Specific Objectives	Study design	Categories: Extent	Prevention	Causes	Detection	Management	Implications	Key findings
5	Wadhwa *et al*.	2021	Oxford Academic	India	Automation in signal management in pharmacovigilance—an insight.	To review the recent developments of automated systems associated with the detection and prioritization of potential signals with the help of effective healthcare databases to enhance public health safety.	Review article				✓			Automated signal detection is highly based upon the data mining of the spontaneous reporting system such as reports from healthcare professionals, observational studies, medical literature or from social media. The main components of automated signal detection are data extraction, data acquisition, data selection, and data analysis and data evaluation. This system must be developed in the correct format and context, which eventually emphasizes the quality of data collected and leads to optimal decision-making based on scientific evaluation.
6	Adam Lavertu *et al.*	2021	Clinical Pharmacology & Therapeutics	USA	A New Era in Pharmacovigilance: Toward Real-World Data and Digital Monitoring	Several different approaches to modern pharmacovigilance, including spontaneous reporting databases, electronic health record monitoring and research frameworks, social media surveillance, and the use of digital devices. Some of these platforms are well-established while others are still emerging or experimental. We highlight both the potential opportunity, as well as the existing challenges within these pharmacovigilance systems that have already begun to impact the drug development process, as well as the landscape of postmarket drug safety monitoring.	Mini review				✓			The development of massive data sources for future pharmacovigilance efforts creates an opportunity for advances in deep learning and anomaly detection. A continuously learning artificial intelligence system could not only learn to integrate these heterogeneous data sources for real-time case detection but could also help identify potential cases and interface with members of the pharmacotherapy community to gather more information when needed.
7	Rodrigo Postigo *et al*.	2018	Drug Safety	UK	EudraVigilance Medicines Safety Database: Publicly Accessible Data for Research and Public Health Protection	The main objective of this article is to describe the new access to the database together with the opportunities that this new access can bring for research. It is intended to promote an appropriate use of the data to support the safe and effective use of medicines.	Special article		✓					The European Union legislation gives the EudraVigilance (EV) database the core role of managing reports of suspected adverse reactions and determine changes in the safety profile of medicinal products used in medical practice. The EV has demonstrated its value especially in the area of signal detection, providing a tool to analyse the safety data submitted.
8	Jenna Wong *et al*	2022	Drug Safety	Switzerland	Applying Machine Learning in Distributed Data Networks for Pharmacoepidemiologic and Pharmacovigilance Studies: Opportunities, Challenges, and Considerations.	In this paper, we discuss opportunities, challenges, and considerations for applying Machine Learning (ML) in Distributed Data Networks (DDNs) for pharmacoepidemiologic and pharmacovigilance studies. First, we discuss the major types of activities performed by DDNs and how ML may be used. Next, we discuss practical data-related factors infuencing how DDNs work in practice. We then combine these discussions and jointly consider how opportunities for ML are afected by practical data-related factors for DDNs, leading to several challenges. We present diferent approaches for addressing these challenges and highlight eforts that real-world DDNs have taken or are currently taking to help mitigate them. Despite these challenges, the time is ripe for the emerging interest to use ML in DDNs, and the utility of these data-adaptive modeling techniques in pharmacoepidemiologic and pharmacovigilance studies will likely continue to increase in the coming years.	Review article		✓		✓			Using ML in DDNs for pharmacoepidemiology and pharmacovigilance holds great promise. This is an emerging area of interest for DDNs because of the recent methodologic advancements in the feld, and the valuable groundwork that many DDNs have already laid through investments in harmonizing datasets, developing standardised processes, tools, and analytics, and building collaborative relationships between data partners and with various stakeholders. The future holds much promise for the use of ML in DDNs, and we expect that the utility of these data-adaptive methods for enhancing pharmacoepidemiologic and pharmacovigilance studies will likely continue to increase in the years to come.
9	Philip Michael Tregunno *et al*	2014	Drug Safety	UK	Performance of Probabilistic Method to Detect Duplicate Individual Case Safety Reports	The aim of this study was to evaluate probabilistic record matching for duplicate detection, and to characterise the main sources of duplicate reports within each data set.	Original Research Article	✓	✓	✓	✓	✓	✓	Probabilistic record matching, as implemented in vigiMatch, achieved good predictive value for confirmed or likely duplicates in each data source. Most of the false positives corresponded to otherwise related reports; less than 10 % were altogether unrelated. A substantial proportion of the correctly identified duplicates had not previously been detected by national centre activity. On one hand, vigiMatch highlighted duplicates that had been missed by rule-based methods, and on the other hand its lower total number of suspected duplicates to review improved the accuracy of manual review.

For peer review only

No.	Author(s)	Year of publication	Journal	Country	Title	Specific Objectives	Study design	Categories						Key findings
								Extent	Prevention	Causes	Detection	Management	Implications	
10	Benjamin Kompa *et al*	2022	Drug Safety	USA	Artifcial Intelligence Based on Machine Learning in Pharmacovigilance: A Scoping Review	The present study conducted a scoping review of the use of artificial intelligence based on machine learning to understand how it is used for pharmacovigilance tasks, characterize differences with other fields, and identify opportunities to improve pharmacovigilance through the use of machine learning.	Scoping Review				✓	✓		While certain modern practices have begun to appear, many of the primary reasons for the recent success of artificial intelligence (AI) have yet to be translated into pharmacovigilance. Without certain structural changes, PV is unlikely to experience similar kinds of advancements from current approaches to AI.
11	Marin Banovac *et al*	2017	Drug Safety	UK	Patient Reporting in the EU: Analysis of EudraVigilance Data	This descriptive study aims to provide insight into patient reporting for the totality of the EU by querying the EudraVigilance (EV) database for the period of 3 years before the new pharmacovigilance legislation became operational and the 3 years after as well as comparing patient reports with those from healthcare professionals (HCPs) where feasible.	Descriptive study			✓				Patient reporting complemented reporting by HCPs. Reports were submitted for which the patient and HCP were both listed as primary sources. Patients were motivated to report adverse reactions, especially those that affected their quality of life. Sharing these results with National Competent Authorities and patient associations can inform training and awareness on patient reporting.
12	Gianmario Candore *et al*	2022	Drug Safety	Netherlands	The Impact of Mandatory Reporting of Non-Serious Safety Reports to EudraVigilance on the Detection of Adverse Reactions	This paper reports on a series of analyses performed to assess the number and nature of non-serious reports and their effect on signal detection in EudraVigilance.	Descriptive study			✓				The new requirement to report non-serious suspect reactions increased report submissions, improving sensitivity without affecting statistical efficiency. As non-serious reports in EudraVigilance rise, ongoing monitoring is crucial for signal detection impact. Further study is needed to assess the qualitative impact of non-serious reports on signal nature and evaluation.
13	John van Stekelenborg *et al*	2022	Drug Safety	USA	Individual Case Safety Report Replication: An Analysis of Case Reporting Transmission Networks	The present study aimed to investigate the magnitude of replication, the variability among recipients, and the subsequent divergence across recipients of ICSRs.	Original Research Articl						✓	Replication of ICSRs and the variation of available safety information in recipient databases were quantified and shown to be substantial. Multiple processors and medical reviewers will likely handle the same original ICSR as a result of replication. Aside from the obvious duplicate work, this phenomenon could conceivably lead to differing clinical assessments and decisions. If replication could be reduced or even eliminated, this would enable more focus on activities with a benefit for patient safety.
14	Rachel L Richesson *et al*	2008	Drug Safety	USA	An Automated Standardized System for Managing Adverse Events in Clinical Research Networks	We describe an Adverse Event Data Management System (AEDAMS) that is used across multiple study designs in the various clinical research networks and multi-site studies for which we provide data and technological support. Investigators enter AE data using a standardized and structured web-based data collection form	Author manuscript		✓			✓		The automated AEDAMS promptly reports adverse events (AEs) to various parties, enhancing participant safety oversight, especially in multi-site protocols. Standardized AE reporting in clinical research networks enables centralized monitoring, potentially improving agent safety comprehension across studies. Automated systems offer speed, efficiency, and scalability, impacting patient safety and streamlining research processes. Future studies should rigorously evaluate AE management systems, measuring efficiency, reliability, accuracy, and potential reductions in patient morbidity and mortality resulting from improved communication and decision-making. Such evaluations would facilitate faster application of investigational treatments and enhance overall research quality and safety.

For peer review only

No.	Author(s)	Year of publication	Journal	Country	Title	Specific Objectives	Study design	Categories: Extent	Prevention	Causes	Detection	Management	Implications	Key findings
15	Michele Fusaroli *et al*	2021	Frontiers in Pharmacology	Italy	Development of a Network-Based Signal Detection Tool: The COVID-19 Adversome in the FDA Adverse Event Reporting System	To propose a network-based approach on co-reported events to help assess disproportionalities and to effectively identify, in a timely manner, the disease-, comorbidity- and drug-related syndromes, especially in a rapidly changing low-resources environment such as that of COVID-19.	Brief research report article.				✔			The Adversome detects plausible new signals and iatrogenic syndromes. The network approach complements traditional pharmacovigilance analyses and may represent a more effective signal detection technique to guide clinical recommendations by regulators and specific follow-up confirmatory studies.
16	Rave Harpaz *et al*	2010	BMC Bioinformatics.	USA	Mining multi-item drug adverse effect associations in spontaneous reporting systems.	This paper examines the application of a well-established data mining method known as association rule mining and demonstrates its value.	Research article.		✔		✔	✔	✔	Our findings demonstrate that multi-item ADEs are present and can be extracted from the FDA's adverse effect reporting system using our methodology, suggesting that our method is a valid approach for the initial identification of multi-item ADEs. The study also revealed several limitations and challenges that can be attributed to both the method and quality of data.
17	F Martin-Sanchez *et al*	2014		Australia	Big data in medicine is driving big changes.	To summarise current research that takes advantage of "Big Data" in health and biomedical informatics applications.	Review article		✔	✔	✔	✔	✔	The survey highlights ongoing development of powerful new methods for turning large-scale, and often complex, data into information that provides new insights into human health, in a range of different areas. Consideration of this body of work identifies several important paradigm shifts that are facilitated by big data resources and methods in clinical and translational research, from hypothesis-driven research to data-driven research, and in medicine, from evidence-based practice to practice-based evidence. The increasing scale and availability of large quantities of health data require strategies for data management, data linkage, and data integration beyond the limits of many existing information systems. As our ability to make sense of that data improves, the value of the data will continue to increase. All areas of biomedicine stand to benefit from Big Data and the associated technologies.
18	Marie Lindquist	2004	Drug Safety	Sweden	Data Quality Management in Pharmacovigilance.	To describe the first three steps of the data processing cycle (collection, entry, storage) and the different quality dimensions associated with these steps, together with examples relevant to pharmacovigilance data	Leading article.				✔			Ensuring data quality throughout processing is crucial in pharmacovigilance. The implementation of robust data quality management practices is essential to address issues perpetuated through the processing cycle. While regulatory requirements dictate reporting standards, database systems largely manage data quality, often without comprehensive appraisal. Inter-system data exchange poses additional quality risks. However, initiatives like 'good pharmacovigilance practice' and emerging conferences indicate progress toward enhancing data quality across the pharmaceutical landscape. Transparent, well-documented quality systems are imperative. Despite acknowledgment of shortcomings in current systems, ongoing efforts aim to reduce errors and misinterpretations, facilitating safer pharmacovigilance processes globally.

For peer review only

No.	Author(s)	Year of publication	Journal	Country	Title	Specific Objectives	Study design	Categories						Key findings
								Extent	Prevention	Causes	Detection	Management	Implications	
19	Monica A. Muñ oz *et al*	2020	Drug Safety	USA	Towards Automating Adverse Event Review: A Prediction Model for Case Report Utility.	This study aimed to develop and validate a model predictive of an individual case safety report (ICSR) pharmacovigilance utility (PVU). Techniques to systematically identify and distinguish higher utility ICSRs from lower utility ones will improve timeliness in managing safety signals.	Research article.				✔			The model demonstrated the feasibility of developing a tool predictive of ICSR utility. The model's modest discriminative ability highlighted opportunities for further enhancement and suggested algorithms tailored to safety issues may be benefcial.
20	José Luis Oliveira *et al*	2013	Pharmacoepidemiology & Drug Safety	Portugal	The EU-ADR Web Platform: delivering advanced pharmacovigilance tools.	To describe the EU-ADR Web Platform built to facilitate accessing, monitoring and exploring pharmacovigilance data, enabling an in-depth analysis of adverse drug reactions risks.	Website		✔		✔	✔		The EU-ADR European project embraces innovative pharmacovigilance research methodologies through the creation of a web platform providing advanced drug data exploration and assessment features. Whereas, in the past, post-marketing drug assessment required intense validation tasks, the in silico pharmacology community is now endowed with the tools required to quickly analyse specific ADRs, further improving drug safety monitoring. The EU-ADR Web Platform enables streamlined access to drug dataset analysis features, including the evaluation of results from EU-ADR workflows and the sharing of data amongst research partners, all within a highly responsive and unique web-based workspace.
21	Helen R. Gosselt *et al*	2022	Pharmacoepidemiology & Drug Safety	Netherlands	Development of a multivariate prediction model to identify individual case safety reports which require clinical review.	Objectives: To develop a prediction model to identify individual case safety reports that require clinical review, including potential signal triggering reports. Secondly, to identify the most important features of these reports.	Original Research Article.						✔	The number of individual case safety reports in pharmacovigilance databases are increasing.<br>New approaches are required to prioritise individual case reports based on their potential signal triggering value since case-by-case review is very time consuming.<br>The authors developed a prediction model using structured data of individual case safety reports to identify potential signal triggering reports in the Netherlands.<br>The authors identified nine most important predictors that were used to identify potential signal triggering reports.<br>The prediction model could be further optimised using additional predictors such as text fields or other unstructured data from case reports.
22	Tomas Lasys *et al*	2023	BJCP	Netherland	Unintended impact of pharmacovigilance regulatory interventions: A systematic review.	This systematic literature review investigated how often the unintended impact of regulatory interventions was considered in publications of studies investigating pharmacovigilance regulatory interventions in Europe.	Review article.			✔				The unintended impact of pharmacovigilance regulatory interventions was reported in only a quarter of identified publications. There was no apparent increase in attention to unintended impact assessments after the update of the Good Pharmacovigilance Practice guidelines.
23	Ruta Mockute *et al*	2017	Drug Safety	USA	Artificial Intelligence Within Pharmacovigilance: A Means to Identify Cognitive Services and the Framework for their Validation.	The aim of this study was to identify areas across the PV value chain that can be augmented by cognitive service solutions using the methodologies of contextual analysis and cognitive load theory.	Review article.		✔					As individual case safety report volumes increase, artificial intelligence can be a means to help mitigate complex decision making for pharmacovigilance professionals. At various decision points in the PV process, cognitive services were identified and developed to assist pharmacovigilance users. These services were validated using a framework leveraging the Acceptance Quality Limit method, to ensure appropriate performance and quality control.

For peer review only

No.	Author(s)	Year of publication	Journal	Country	Title	Specific Objectives	Study design	Categories: Extent	Prevention	Causes	Detection	Management	Implications	Key findings
24	Jianxing He *et al*	2019	Nat Med.	China	The practical implementation of artificial intelligence technologies in medicine.	The development of artificial intelligence (AI)-based technologies in medicine is advancing rapidly, but real-world clinical implementation has not yet become a reality. Here we review some of the key practical issues surrounding the implementation of AI into existing clinical workflows, including data sharing and privacy, transparency of algorithms, data standardization, and interoperability across multiple platforms, and concern for patient safety. The study summarizes the current regulatory environment in the United States and highlights comparisons with other regions in the world, notably Europe and China.	Review article.					✓		AI has recently experienced an era of explosive growth across many industries, and the healthcare industry is no exception. Studies across multiple medical specialties have employed AI to mimic the diagnostic abilities of physicians. The hope is that AI may augment the ability of humans to provide healthcare. However, although these technologies are rapidly advancing, their implementation into patient-care settings has not yet become widespread.
25	Juergen Schmider	2018	Clinical Pharmacology & Therapeutics.	USA	Innovation in Pharmacovigilance: Use of Artificial Intelligence in Adverse Event Case Processing.	The current pilot was undertaken to prove the viability of commercially offered machine-learning solutions in the application for case processing. The pilot paradigm was used to simultaneously test the proposed solutions of three commercial vendors for the ability to extract case critical information from source documents to identify valid AE cases after training the machine-learning algorithms with source documents and database content rather than annotated source documents.	Research article.				✓			The pilot was successful in confirming the feasibility of using AI-based tools to support PV operations and in demonstrating the viability of an efficient training method that does not require time-consuming and costly annotations. Finally, the evaluation and scoring method used in the pilot was able to differentiate vendor capabilities and identify vendor 1 as the best candidate to move into the discovery phase.
26	David John Lewis *et al*	2019	PMC	Germany	Utilizing Advanced Technologies to Augment Pharmacovigilance Systems: Challenges and Opportunities.	This paper provides a series of considerations for the applicability of new IT, with explanations of the technology and its applicability within specific domains of the PV system. The overall goal of automation is to provide high-quality safety data in the correct format, in context, more quickly, and with less manual effort.	Rreview article		✓		✓	✓		The rapid advancement of technologies often outpaces regulations governing pharmacovigilance (PV) systems. Yet, intelligent automation offers avenues for system improvement. Owners must ensure the suitability, design, and reliability of advanced technologies, ensuring PV systems remain effective. Trust in intelligent automation is paramount, aiming to deliver safety data accurately and informatively, enhancing evidence quality for optimal scientific evaluation. Alignment with perspectives in biomedical sciences underscores the need for PV system owners to adapt progressively to artificial intelligence, starting with process automation. Algorithmic processing has demonstrated superior performance in binary, repetitive tasks, advocating for its integration into PV systems.
27	European Medicines Agency	2017	Not applicable	Europe	Guideline on good pharmacovigilance practices (GVP): Module VI – Collection, management and submission of reports of suspected adverse reactions to medicinal products (Rev 2)	Section VI.B. of this Module highlights the general principles, based on the pharmacovigilance guidelines E2A, E2B and E2D of the International Council for Harmonisation of Technical Requirements for Pharmaceuticals for Human Use (ICH) (see GVP Annex IV), in relation to the collection, recording and submission of individual reports of suspected adverse reactions associated with medicinal products for human use. The definitions and guidance provided in Section VI.A. and the EU specific requirements presented in Section VI.C. should be followed. All applicable legal requirements are referenced in the way explained in the GVP Introductory Cover Note and are usually identifiable by the modal verb "shall". Guidance for the implementation of legal requirements is provided using the modal verb "should".	Guidelines		✓		✓	✓		Duplicate detection in EudraVigilance – Collaboration between the Agency, competent authorities in Member States and marketing authorisation holders - Duplicate ICSRs submitted to EudraVigilance by the same sender and identified by the Agency.
28	ArisGlobal	2019	Not applicable	USA	ArisGlobal Announces LifeSphere® MultiVigilance 10, Industry's First End-to-End Safety System With Production-Ready Automation.	LifeSphere® MultiVigilance 10 features all new architecture and design, incorporating the latest in cognitive automation technology to deliver groundbreaking efficiency gains to PV teams	Website					✓		Under tightening budgets and evolving global regulations, safety case volumes continue to grow by nearly 15% each year, creating challenges for today's drug safety teams. To address these pressures, the software leverages cognitive computing technologies such as natural language processing and machine learning, providing a single, global database that supports end-to-end automation of all adverse event processing.

For peer review only

No.	Author(s)	Year of publication	Journal	Country	Title	Specific Objectives	Study design	Categories						Key findings
								Extent	Prevention	Causes	Detection	Management	Implications	
29	Rajesh Ghosh *et al*	2020	Pharmaceutical	USA	Automation Opportunities in Pharmacovigilance: An Industry Survey.	This paper aims to address the need for a systematic review framework for automation of the ICSR process from the value, impact, perceived risk, and opportunity point of view.	Original reserch article					✓		In total, 15 member companies of TransCelerate participated in the survey, identifying ICSR process steps with high effort, potential for significant automation benefits, manageable automation risks, and low current automation levels as prime candidates for future automation. These steps include language translations, case verification, quality control, prioritization, data entry, alerting, workflow management, and monitoring. Conversely, certain steps like submission, already automated for some time, show lower potential for further automation. Despite successful adoption of intelligent automation in other domains, its integration into pharmacovigilance, particularly the ICSR process, remains limited, partly due to perceived risks to patient safety, which are expected to decrease with further successful applications.
30	Michael G Kahn *et al*	2016	The Journal for Electronic Health Data and Methods	USA	A Harmonized Data Quality Assessment Terminology and Framework for the Secondary Use of Electronic Health Record Data.	To describe harmonized data quality (DQ) assessment terms, methods, and reporting practices and establish a common understanding of the strengths and limitations of electronic health record (EHR) data for operational analytics, quality improvement, and research.	Review article		✓	✓				A consistent, common DQ terminology, organized into a logical framework, is an initial step in enabling data owners and users, patients, and policymakers to evaluate and communicate data quality findings in a well-defined manner with a shared vocabulary. Future work will leverage the framework and terminology to develop reusable data quality assessment and reporting methods.
31	Tomas Bergvall *et al*	2013	Drug Safety	Sweden	vigiGrade: A Tool to Identify Well-Documented Individual Case Reports and Highlight Systematic Data Quality Issues.	The objective of this study was to propose a measure of completeness and identify predictors of well-documented reports, globally.	Research article.			✓				vigiGrade distinguishes different aspects of quality according to the outline in Lindquist. An important advantage compared with the earlier implementation of documentation grading in VigiBase is that the vigiGrade completeness score considers each dimension in parallel, instead of in sequence: even when information on time-to-onset is lacking. The other dimensions are evaluated and accounted for in the total completeness score. vigiGrade considers many of the same fields as the structured assessment proposed by Agbabiaka et al. but is less comprehensive. Specifically, it does not evaluate dimensions for which absence of information cannot be distinguished from information on absence in VigiBase. On the other hand, it is a scalable solution that allows automated database-wide analyses. By design, it allows for significant penalties of a variety of missing dimensions.

For peer review only

No.	Author(s)	Year of publication	Journal	Country	Title	Specific Objectives	Study design	Categories						Key findings
								Extent	Prevention	Causes	Detection	Management	Implications	
32	M. Hauben *et al*	2007	Drug Safety	USA	Extreme Duplication' in the US FDA Adverse Events Reporting System Database.	The paper describes duplication as one of the numerous well-recognised forms of data corruption and distortion in spontaneous reporting systems, with duplicate detection algorithms and procedures being included in the commercial data-mining software the authors used.	Research article.				✓	✓	✓	Extreme duplication was discovered within the US FDA Adverse Events Reporting System (AERS) Database. This duplication problem in regulatory and company databases can arise from various sources and a failure to connect related cases. Vendors do make efforts to eliminate duplicate cases before providing data to customers, but sometimes the criteria used may not be sufficient for accurate identification and removal of duplicates. The impact of extreme duplication in AERS, compared to the World Health Organization (WHO) database, is evident, as it affected data-mining results in AERS but not in WHO. Duplicate reports in spontaneous reporting systems may carry unique information value and deserve further investigation. Acknowledging the limitations and challenges of spontaneous reporting system (SRS) data, including inconsistencies, errors, duplications, and incomplete data in critical fields. It's crucial to approach any findings based on SRS data with an appropriate level of caution, taking into account these limitations and the potential impact of duplication on data analysis.
33	CIOMS	2010	Not applicable	Switzerlan d	Practical Aspects of Signal Detection in Pharmacovigilance.	The objective of the CIOMS VIII report is to provide useful points for consideration to manufacturers, regulatory authorities, international monitoring centers, and others wishing to establish or understand the output of a systematic and holistic strategy to better manage the entire "lifecycle" of a drug safety signal. This lifecycle includes signal detection, signal prioritization, and signal evaluation.	Report			✓	✓		✓	Many databases of large spontaneous reporting systems contain duplicate reports. The identification and elimination of duplicates from analyses, therefore, is advantageous for signal evaluation. However, current duplicate detection procedures, some of which are applied prospectively (i.e. prior to data mining) and others retrospectively (i.e. after data mining), have limitations and enhanced methods of duplicate detection are being developed.
34	Kevin Pozsgai *et al*	2022	Front. Pharmacol.	Hungary	Analysis of pharmacovigilance databases for spontaneous reports of adverse drug reactions related to substandard and falsified medical products: A descriptive study.	To identify and describe the characteristics of cases that are related to adverse drug reactions potentially originating from counterfeit medication using publicly available pharmacovigilance data.	Descriptive case series			✓				The identification and elimination of duplicates between pharmacovigilance databases is almost impossible. Multiple ADRs may be presented for one individual, resulting in report duplications or triplications and making it almost impossible to eliminate this bias with manual analysis.
35	Hung E *et al*	2023	Drug Safety	USA	More extreme duplication in FDA Adverse Event Reporting System detected by literature reference normalization and fuzzy string matching.	The objective of this analysis is to determine if variations of the same literature references observed in FAERS can be resolved with text normalization and fuzzy string matching.	Research article.	✓						Normalized references can be merged via fuzzy string matching to improve enumeration of all the individual case safety reports that refer to the same article. Inclusion of the PubMed ID and adherence to the Vancouver convention could facilitate identification of duplicates in the FAERS dataset. Awareness of this phenomenon may improve disproportionality analysis, especially in areas such as addictovigilance.
36	Uppsala Monitoring Centre	2017	Uppsala Reports	Sweden	Text is key for eliminating duplicate reports.	To describe the role of text in eliminating duplicate reports.	Leading article.			✓				Duplicate Cases – Two or more records describing the same occurrence of events – same adverse effect(s) experienced with the same medical product at the same time – for the same patient. Due to reporting inaccuracies, two records can be considered duplicates even if they do not contain an identical list of events.

For peer review only

No.	Author(s)	Year of publication	Journal	Country	Title	Specific Objectives	Study design	Categories						Key findings
								Extent	Prevention	Causes	Detection	Management	Implications	
37	Uppsala Monitoring Centre		Reporting Fact Sheet	Sweden	Sharing pharmacovigilance data in the WHO Programme for International Drug Monitoring.	To describe the sharing of pharmacovigilance data in the WHO Programme for International Drug Monitoring.	Leading article.			✓	✓	✓		The minimum information required for an ICSR to be valid is a case identifier, reporter and patient information, and information on suspect medicine and reaction/event. All national case reports fulfilling the minimum requirements should be shared with the WHO Programme. However, quality of data and completeness of case reports are always important and reports should include as much information as possible to facilitate assessment. Free text/additional information can be provided in the original language. All information available on the original case report should be included when sending an ICSR to VigiBase, with the exception of confidential patient and reporter details that could potentially be used to identify individuals.
38	European Medicines Agency	2017		Netherlands	Guideline on good pharmacovigilance practices (GVP) Module VI Addendum I – Duplicate management of suspected adverse reaction reports.	The revision contains the following changes: - Alignment with revision 2 of GVP Module VI; - Update of electronic reporting modalities of ICSRs in the new ICH-E2B(R3) format; - Update overall with the revised pharmacovigilance legislation as regards the roles and responsibilities of the Agency, the competent authorities in Member States as well as marketing authorisation holders in relation to the operation of duplicate detection and management of reports of suspected adverse reactions; - Guidance on how to inform the Agency of suspected duplicates in EudraVigilance; - Changes for consistent presentation of GVP document.	Report		✓	✓	✓	✓	✓	Duplicate cases are generally managed through a process of merging two or more cases into one master case. This process can consist of one of the following approaches: The master case can either be based on one of the existing cases, with information from the other subordinate duplicate cases added unless the same, or more precise, information is already present in the master case (this is referred to in this document as "Allocation of a master case"), or; The master case can be created as a new case combining the information from the subordinate duplicate cases (this is referred to in this document as "Creation of a master case"). Regardless of the approach chosen, the master case should always contain all case reference numbers from all subordinate duplicate cases, such that they can be easily traced. The master case should reflect the most accurate and up-to-date information available to the organisation.
39	Agency for Medicinal Products and Medical Devices of Croatia (HALMED)	2008-2013	Not applicable	Croatia	Strengthening Collaboration for Operating Pharmacovigilance in Europe (SCOPE).	SCOPE was divided into eight separate work packages, with five work packages focusing on pharmacovigilance topics to deliver specific and measureable objectives, ranging from improvements in Adverse Drug Reaction (ADR) reporting to the assessment of quality management systems.	Report		✓	✓	✓	✓	✓	The first step in duplicate management is to identify possible ADR report duplicates. Screening for duplicates is usually carried out when a new report arrives in the database, i.e. during data entry or during the process of loading ICSRs that have been received electronically. Duplicates can be detected even before entering the case into the database – that is usually the case during manual data entry of cases received by paper, fax or phone. Duplicates can also be detected during periodic data review and the signal management process when detailed analysis of cases is performed.

For peer review only

No.	Author(s)	Year of publication	Journal	Country	Title	Specific Objectives	Study design	Categories						Key findings
								Extent	Prevention	Causes	Detection	Management	Implications	
40	Ritesh Bhangale *et al*	2017	Perspect Clin Res	India	A day in the life of a pharmacovigilance case processor.	The paper describes the routine experiences of a PV case processor.	Leading article.		✓					For the case processor, every case is different, even within the same study. In fatal cases, the case processor will have to check many things such as the cause of death and autopsy details, whereas in other scenarios, the processor will have to check adverse events of special interest and important medical events and report the same to stakeholders. The case processor should ensure that there are no grammatical/spelling errors in the case, while routing it to the next workflow, and shares the best practices, which helps other associates to do their job efficiently.
41	Priyanka Paygude *et al* .	2013	International Journal of Modern engineering Research	India	Automated Data Validation Testing Tool for Data Migration Quality Assurance.	The paper proposed automation of the data migration validation testing process for quality assurance and risk control across industries.	Research article.		✓					Data migration projects pose significant challenges and risks, e.g., time overruns and budget constraints. The proposed system ensures data quality through standardized testing methods across the enterprise. This solution could help organizations save time, reduce costs, minimize manual efforts, and guarantee data quality assurance throughout the migration process.
42	Saqib Hakak *et al*	2018	Plos One	Malaysia	A new split based searching for exact pattern matching for natural texts.	This work proposes a novel idea to achieve both time efficiency and memory consumption by splitting query string for searching in Corpus.	Research article.				✓			This paper proposed a novel idea for exact string matching to achieve both time and space efficiency regardless of query pattern length, dataset size and scripts.
43	Christen P	2012	SpringerLink.	UK	Data matching: concepts and techniques for record linkage, entity resolution, and duplicate detection. Springer: Data-centric systems and applications.	First book on a topic of growing importance for applications. Brings together research from various areas like databases, statistics, information retrieval, data mining, and machine learning. Details the data matching process step by step. Includes an overview of freely available data matching systems and a detailed discussion of practical aspects and limitations.	Book				✓			By providing the reader with a broad range of data matching concepts and techniques and touching on all aspects of the data matching process, this book helps researchers as well as students specializing in data quality or data matching aspects to familiarize themselves with recent research advances and to identify open research challenges in the area of data matching. To this end, each chapter of the book includes a final section that provides pointers to further background and research material.
44	Liu H *et al* .	2014	EANN	UK	Categorization and Construction of Rule Based Systems.	This paper aims to introduce the theory of rule-based systems especially on categorization and construction of such systems from a conceptual point of view. This also details rule-based systems for classification tasks.	Conference paper				✓			This introduces novel insights for both researchers and practitioners, presenting a new paradigm for rule-based systems in various applications. It advocates for a data-driven approach within machine learning for constructing complex systems, contrasting with traditional knowledge-based methods. Emphasizing the significance of the data-driven approach, it delves into a specialized classification rule-based system, detailing its construction framework and crucial considerations in method selection for rule generation, simplification, and representation operations.
45	Wong CK *et al*	2015	Drug Safety	Australia	Standardisation of the FAERS database: a systematic approach to manually recoding drug name variants	The aim of this study was to develop and implement a data cleaning protocol to identify and resolve drug nomenclature issues. The key 'data treatment' plan involved standardising drug names held in the FAERS database.	Research article.				✓	✓		The millions of reports enclosed in the FAERS contain valuable information that is of interest to pharmacovigilance, toxicology and post-marketing surveillance researchers. With the standardisation of the drug nomenclature, the database can be better utilised by research groups around the world.

For peer review only

No.	Author(s)	Year of publication	Journal	Country	Title	Specific Objectives	Study design	Categories						Key findings
								Extent	Prevention	Causes	Detection	Management	Implications	
46	Jae Dong Yang.	1997	Data & Knowledge Engineering	South Korea	A fuzzy match framework for rule-based programming.	To propose a framework to support a semantic based inexact match with Fuzzy Match Predicate (F_MP).	Research article.				✓			The paper demonstrates that: (1) F_MP is a uniform framework to provide the rule-based languages with fuzzy match facilities semantically enhanced, and that (2) its semantics conform well to those of the relational one.
47	Kreimeyer K *et al*	2022	Drug Safety	USA	Increased Confidence in Deduplication of Drug Safety Reports with Natural Language Processing of Narratives at the US Food and Drug Administration.	To improve and optimize an existing deduplication algorithm that used both structured and free-text data; develop a web-based application to support data processing; and conduct a 6-month dedicated evaluation to assess the potential operationalization of the deduplication process in the FDA.	Research article.				✓	✓	✓	Comparing algorithm predictions with reviewer determinations of duplicates for twenty-seven files for case series reviews (with a median size of 281 reports), the average pairwise recall and precision were 0.71 (SD ± 0.32) and 0.67 (SD ± 0.34). Overall, reviewers felt confident about the algorithm and expressed their interest in using it. These findings support the operationalization of the deduplication process for case series review as a supplement to human review.
48	Kreimeyer K *et al*	2017	Drug Safety	USA	Using Probabilistic Record Linkage of Structured and Unstructured Data to Identify Duplicate Cases in Spontaneous Adverse Event Reporting Systems.	To develop a probabilistic record linkage algorithm for identifying duplicate cases in the US Vaccine Adverse Event Reporting System (VAERS) and the US Food and Drug Administration Adverse Event Reporting System (FAERS).	Research article.				✓		✓	The algorithm was shown to be effective at identifying pre-linked duplicate VAERS reports. The narrative text was not shown to be a key component in the automated detection evaluation; however, it is essential for supporting the semi-automated approach that is likely to be deployed at the Food and Drug Administration, where medical reviewers perform some manual review of the most highly ranked reports identified by the algorithm.
49	Harpaz R *et al*	2017	Journal of Bioinformatics.	USA	Toward multimodal signal detection of adverse drug reactions.	Improving mechanisms to detect adverse drug reactions (ADRs) is key to strengthening post-marketing drug safety surveillance. Signal detection is presently unimodal, relying on a single information source. Multimodal signal detection is based on jointly analyzing multiple information sources. Building on, and expanding the work done in prior studies, the aim of the article is to further research on multimodal signal detection, explore its potential benefits, and propose methods for its construction and evaluation.	Research article.				✓			The results support the notion that utilizing and jointly analyzing multiple data sources may lead to improved signal detection. Given certain data and benchmark limitations, the early stage of development, and the complexity of ADRs, it is currently not possible to make definitive statements about the ultimate utility of the concept. Continued development of multimodal signal detection requires a deeper understanding of the data sources used, additional benchmarks, and further research on methods to generate and synthesize signals.
50	Kreimeyer K *et al*	2021	Computers in Biology and Medicine	USA	Feature engineering and machine learning for causality assessment in pharmacovigilance: Lessons learned from application to the FDA Adverse Event Reporting System.	To support the automated classification of Food and Drug Administration (FDA) Adverse Event Reporting System (FAERS) reports for their usefulness in assessing the possibility of a causal relationship between a drug product and an adverse event.	Research article.				✓			Causal inference from FAERS reports depends on many components with complex logical relationships that are yet to be made fully computable. Efforts focused on readily addressable tasks, such as quickly eliminating unassessable reports, fit naturally in SE's thought processes to provide real enhancements for FDA workflows.
51	Kim HR *et al*	2022	Medicine	South Korea	Analyzing adverse drug reaction using statistical and machine learning methods: A systematic review.	This systematic review aimed to examine the analytical tools by considering original articles that utilized statistical and machine learning methods for detecting ADRs.	Systematic review				✓			This review provides guidelines on which databases are frequently utilized and which analysis methods can be connected. For statistical analysis, >90% of the cases were analyzed by disproportionate or regression analysis of each spontaneous reporting system or electronic medical record database; for machine learning research, however, there was a strong tendency to analyze various data combinations. Only half of the DrugBank database was occupied, and the k-nearest neighbor method accounted for the greatest proportion.

For peer review only

No.	Author(s)	Year of publication	Journal	Country	Title	Specific Objectives	Study design	Categories						Key findings
								Extent	Prevention	Causes	Detection	Management	Implications	
52	CIOMS	2021	Website	Sweden	Making medicines safer for patients everywhere.	This is the fourth of a series of special newsletters that describe the context of CIOMS activities and the work of its member organizations and partners. It describes what the Uppsala Monitoring Centre (UMC) is doing to support and promote patient safety through effective global pharmacovigilance practice.	Not applicable				✔	✔		UMC promotes collaboration between all stakeholders involved in drug risk management and supports harmonization, good pharmacovigilance practice and the dissemination of new guidelines. CIOMS is an important partner in this regard. New working groups on pharmacovigilance topics are needed, for example to explore the link between pharmacovigilance practices and public health, or the role of pharmacoepidemiology as a tool to assess the impact of emerging adverse effects.
53	MedDRA		Website	Not applicable	MeDRA History	In the 1990s, the International Conference on Harmonisation (ICH) recognized the necessity for standardized medical terminology. At that time, available terminologies like COSTART, WHO-ART, J-ART, H-ARTS, ICD-9, and ICD-9CM lacked the required scope and granularity for regulatory authorities and industry needs. Due to infrequent updates, users often created customized versions, leading to a loss of standardization.	Not applicable					✔		MedDRA was based on terminology belonging to the Medicines and Healthcare products Regulatory Agency (MHRA) of UK (previously named Medicines Control Agency) and was developed using the ICH process by the ICH partners, including WHO.
54	Oracle.	2021	Website	USA	Argus Trusted safety case management.	To describe how to promote efficient case management while reducing the manual effort of a safety and pharmacovigilance team.	Not applicable					✔		Oracle Argus enabled greater and faster insight into product safety, improved productivity, and streamlined and automated safety reporting.
55	Ablebits.	2023	Website	Poland	Ablebits. Ablebits Ultimate Suite.	To describe the tools that can automate most daily tasks in Excel and increase productivity by at least 50%	Not applicable					✔		Finding and removing duplicates in Excel may be a real headache, especially when worksheets contain thousands of rows. Duplicate Remover for Excel does the job quickly and perfectly – it can find and delete duplicates based on one or several key columns, identify unique values, highlight, copy or move the found entries.
56	Brinker AD *et al*	2002	Am J Hematol.	USA	Spontaneous reports of thrombocytopenia in association with quinine: clinical attributes and timing related to regulatory action.	This report includes clinical attributes from the largest case series to date of apparently isolated thrombocytopenia in association with quinine and trends in the receipt of spontaneous adverse event reports to FDA's Center for Drug Evaluation and Research (CDER) for this drug-event combination in relation to regulatory action. In this study, we reviewed reports of spontaneous adverse drug events received by CDER.	Descriptive study						✔	This case series confirms previous smaller series that suggest quinine-associated thrombocytopenia may present rapidly with symptoms of profound thrombocytopenia. Clinicians evaluating patients with new-onset and apparently idiopathic thrombocytopenia should maintain clinical vigilance for ingestion of quinine and elicit a detailed food/dietary supplement history from the patient.
57	Azeroual O *et al .*	2022	Multimodal Technol. Interact.	Germany	A Record Linkage-Based Data Deduplication Framework with DataCleaner Extension.	To make the proposed framework usable we integrated it into a tool that is already used in practice, by developing a prototype of an extension for the well-known DataCleaner. The framework detects and visualises duplicates thereby identifying and providing the user with identified redundancies in a user-friendly manner allowing their further elimination. By removing the redundancies, the quality of the data is improved therefore improving analyses and decision-making. This study makes a call for other researchers to take a step towards the "golden record" that can be achieved when all data quality issues are recognised and resolved, thus moving towards absolute data quality.	Research Article.				✔		✔	This paper, presents a Record Linkage framework consisting of six interconnected steps, i.e., (1) data preparation, (2) search space definition, (3) attribute value comparison, (4) a decision model, (5) clustering of the duplicates and (6) verification. It then transforms into an extension for the DataCleaner tool that follows validation through its application to the real-world RIS. This allows for easy and automatic identification of duplicates stored in the system, with their subsequent elimination. This enriches the original records and thereby contributes to their completeness, thus preparing the data for further processing.

For peer review only

No.	Author(s)	Year of publication	Journal	Country	Title	Specific Objectives	Study design	Categories						Key findings
								Extent	Prevention	Causes	Detection	Management	Implications	
58	Norén GN *et al*.	2007	Data Mining and Knowledge Discovery.	Sweden	Duplicate detection in adverse drug reaction surveillance.	We propose a duplicate detection method based on the hit-miss model for statistical record linkage described by Copas and Hilton, which handles the limited amount of training data well and is well suited for the available data (categorical and numerical rather than free text). We propose two extensions of the standard hit-miss model: a hit-miss mixture model for errors in numerical record fields and a new method to handle correlated record fields.	Research Article.				✓			This paper introduced two generalisations of the standard hit-miss model and demonstrated the usefulness of the extended hit-miss model for automated duplicate detection in WHO drug safety data. The results indicate that the hit-miss model can detect a significant proportion of the duplicates without generating many false leads. Its strong theoretical basis together with the excellent results, should make it a strong candidate for other duplicate detection and record linkage applications.

For peer review only

Appendices

Appendix I: Search strategy in PubMed

((((prevalence[tw] OR incidence[tw] OR extent[tw] **OR** epidemiology[tw] OR frequency[tw]) AND (duplication[tw] OR "Data Duplication"[tw] OR "Duplicate Report*"[tw] OR "Redundant Data"[tw] OR "duplicate record*"[tw] OR replication[tw])) AND ("pharmacovigilance database*"[tw] OR database*[tw]))

OR

(((prevention[tw] OR "prevention strateg*"[tw] OR cause*[tw] OR detection[tw] OR management[tw] OR "management strateg*"[tw] OR deduplication[tw]) AND (duplication[tw] OR "Data Duplication"[tw] OR "Duplicate Report*"[tw] OR "Redundant Data"[tw] OR "duplicate record*"[tw] OR replication[tw])) AND ("pharmacovigilance database*"[tw] OR database*[tw])))

OR

(((impact* OR consequence* OR effect*) AND (duplication[tw] OR "Data Duplication"[tw] OR "Duplicate Report*"[tw] OR "Redundant Data"[tw] OR "duplicate record*"[tw] OR replication[tw])) AND ("pharmacovigilance database*"[tw] OR database*[tw]))

Using the Polyglot Search Translator, we adapted the PubMed search strategy across the other peer-reviewed research databases, namely, Web of Science, Wiley Online Library and EBSCOhost.

Appendix II: Sources of grey literature search

Search terms use: Duplicate management in pharmacovigilance databases.

**Source**	**Organizations**	**Website Links**
London	European Medicines Agency	https://www.ema.europa.eu/en/documents/regulatory-procedural-guideline/guideline-good-pharmacovigilance-practices-gvp-module-vi-addendum-i-duplicate-management-suspected_en.pdf
India	https://allaboutpharmacovigilance.org/	https://allaboutpharmacovigilance.org/15-duplicate-search-in-pharmacovigilance/
Sweden	Uppsala	https://www.uppsalareports.org/articles/text-is-key-for-eliminating-duplicate-reports/
London	Medical literature monitoring	https://www.ema.europa.eu/en/human-regulatory/post-authorisation/pharmacovigilance/medical-literature-monitoring
London	**Clarivate PLC**	https://go.dialog.com/MLM_Duplication_Whitepaper/

For peer review only - http://bmjopen.bmj.com/site/about/guidelines.xhtml

London	PubHive	https://pubhive.com/pulse-literature-1/f/pubhive-automates-duplicate-detection-for-ema%E2%80%99s-mlm-service
Sweden	uppsala	https://who-umc.org/media/3236/reporting-fact-sheet.pdf
Maryland USA	FDA	https://www.accessdata.fda.gov/scripts/cderworld/index.cfm?action=drugsafety:main&unit=1&lesson=1&topic=17
New Jersey USA	flexdatabases	https://flexdatabases.com/6-faq-pv/
New York	citeline	https://pink.citeline.com/-/media/supporting-documents/pink-sheet/2020/02/p0220ema_9.pdf
London	GOV.UK	https://assets.publishing.service.gov.uk/government/uploads/system/uploads/attachment_data/file/949102/Exceptions_and_modifications_to_the_EU_guidance_on_good_pharmacovigilance_practices_that_apply_to_UK_MAHs_v2.pdf
San Francisco	WordPress.com	https://auditandcompliance.wordpress.com/2018/12/23/basic-steps-in-pharmacovigilance-case-processing/
Pakistan	Drug Regulatory Authority of Pakistan	https://www.dra.gov.pk/wp-content/uploads/2022/04/Good-Pharmacovigilance-Guidelines-for-Registration-Holders.pdf
Sweden	Uppsala	https://www.diva-portal.org/smash/get/diva2:1670567/FULLTEXT01.pdf
Lithuania	Biomapas	https://www.biomapas.com/selecting-a-pharmacovigilance-drug-safety-database/
Erupe	ENCePP	https://www.encepp.eu/standards_and_guidances/methodologicalGuide8_4.shtml

For peer review only - http://bmjopen.bmj.com/site/about/guidelines.xhtml

Brussels	AFMPS	https://www.afmps.be/sites/default/files/content/faqs_ev_changes_22-11-2017_afmps-fagg.pdf
Amsterdam	European medicines agency	https://www.adrreports.eu/en/data_quality.html#:~:text=a%20report%20is%20submitted%20by,to%20the%20report%20being%20erroneous.
Amsterdam	European medicines agency	https://www.ema.europa.eu/en/documents/other/scope-training-duplicate-detection-best-practice-guide_en.pdf
Maryland USA	FDA	https://www.fda.gov/drugs/surveillance/questions-and-answers-fdas-adverse-event-reporting-system-faers
New York	IEEE	https://ieeexplore.ieee.org/abstract/document/6732591/similar#similar

For peer review only - http://bmjopen.bmj.com/site/about/guidelines.xhtml

Appendix III: Data extraction instrument

Auth or(s)	Jou rnal	Ti tle	Speci fic Objec tives	Cou ntry	Stu dy des ign	Year of public ation	Ext ent	Preve ntion	Ca use s	Dete ction	Manag ement	Implic ations	Key find ings

Appendix IV: Regions and countries

Region	Number of Publications	Countries
Asia Pacific	6 publications	China<br>India<br>Malaysia<br>South Korea
Europe	27 publications	Sweden<br>Georgia<br>Switzerland<br>Netherlands<br>England<br>Italy<br>Hungary<br>Switzerland<br>Croatia<br>Germany<br>Poland<br>Portugal
Australia	3 publications	Australia
North America	22 publications	USA

For peer review only

For peer review only - http://bmjopen.bmj.com/site/about/guidelines.xhtml

Appendix V: List of 99 articles excluded on full-text review

No.	PMID	Title	Citation	First Author	Journal/Book	Publication Year
1	22360774	Pharmacovigilance	Br J Clin Pharmacol. 2012 Jun;73(6):979-82. doi: 10.1111/j.1365-2125.2012.04249.x.	Edwards IR	Br J Clin Pharmacol	2012
2	25123728	"Big data" and the electronic health record	Yearb Med Inform. 2014 Aug 15;9(1):97-104. doi: 10.15265/IY-2014-0003.	Ross MK	Yearb Med Inform	2014
3	33634545	Suitability of databases in the Asia-Pacific for collaborative monitoring of vaccine safety	Pharmacoepidemiol Drug Saf. 2021 Jul;30(7):843-857. doi: 10.1002/pds.5214. Epub 2021 Mar 23.	Duszynski KM	Pharmacoepidemiol Drug Saf	2021
4	35179704	High-Dimensional Propensity Score-Adjusted Case-Crossover for Discovering Adverse Drug Reactions from Computerized Administrative Healthcare Databases	Drug Saf. 2022 Mar;45(3):275-285. doi: 10.1007/s40264-022-01148-5. Epub 2022 Feb 18.	Volatier E	Drug Saf	2022
5	29474173	Possibility of Database Research as a Means of Pharmacovigilance in Japan Based on a Comparison with Sertraline Postmarketing Surveillance	Value Health Reg Issues. 2018 May;15:1-5. doi: 10.1016/j.vhri.2017.05.002. Epub 2017 Jun 20.	Hirano Y	Value Health Reg Issues	2018
6	32243569	Different Strategies to Execute Multi-Database Studies for Medicines Surveillance in Real-World Setting: A Reflection on the European Model	Clin Pharmacol Ther. 2020 Aug;108(2):228-235. doi: 10.1002/cpt.1833. Epub 2020 May 5.	Gini R	Clin Pharmacol Ther	2020
7	31464008	Establishment of the MID-NET(®) medical information database network as a reliable and valuable database for drug safety assessments in Japan	Pharmacoepidemiol Drug Saf. 2019 Oct;28(10):1395-1404. doi: 10.1002/pds.4879. Epub 2019 Aug 29.	Yamaguchi M	Pharmacoepidemiol Drug Saf	2019
8	31933254	Artificial Intelligence Within Pharmacovigilance: A Means to Identify Cognitive Services and the Framework for Their Validation	Pharmaceut Med. 2019 Apr;33(2):109-120. doi: 10.1007/s40290-019-00269-0.	Mockute R	Pharmaceut Med	2019
9	35239736	Evaluation of pharmacovigilance systems for reporting medication errors in Africa and the role of patients using a mixed-methods approach	PLoS One. 2022 Mar 3;17(3):e0264699. doi: 10.1371/journal.pone.0264699. eCollection 2022.	Sabblah GT	PLoS One	2022
10	30445979	Organizational capacities of national pharmacovigilance centres in Africa: assessment of resource elements associated with successful and unsuccessful	Global Health. 2018 Nov 16;14(1):109. doi: 10.1186/s12992-018-0431-0.	Ampadu HH	Global Health	2018
11	36097239	Artificial Intelligent Context-Aware Machine-Learning Tool to Detect Adverse Drug Events from Social Media Platforms	J Med Toxicol. 2022 Oct;18(4):311-320. doi: 10.1007/s13181-022-00906-2. Epub 2022 Sep 12.	Roosan D	J Med Toxicol	2022
12	29714535	An Alternative to Disproportionality: A Frequency-Based Method for Pharmacovigilance Data Mining	Ther Innov Regul Sci. 2018 May;52(3):294-299. doi: 10.1177/2168479017728986. Epub 2017 Sep 8.	Jokinen JD	Ther Innov Regul Sci	2018
13	30185579	Electronic healthcare databases in Europe: descriptive analysis of characteristics and potential for use in medicines regulation	BMJ Open. 2018 Sep 5;8(9):e023090. doi: 10.1136/bmjopen-2018-023090.	Pacurariu A	BMJ Open	2018
14	32026758	Combining big data search analytics and the FDA Adverse Event Reporting System database to detect a potential safety signal of mirtazapine abuse	Health Informatics J. 2020 Sep;26(3):2265-2279. doi: 10.1177/1460458219901232. Epub 2020 Feb 6.	Spachos D	Health Informatics J	2020
15	23512870	Drug safety data mining with a tree-based scan statistic	Pharmacoepidemiol Drug Saf. 2013 May;22(5):517-23. doi: 10.1002/pds.3423. Epub 2013 Mar 20.	Kulldorff M	Pharmacoepidemiol Drug Saf	2013
16	25151493	Text mining for adverse drug events: the promise, challenges, and state of the art	Drug Saf. 2014 Oct;37(10):777-90. doi: 10.1007/s40264-014-0218-z.	Harpaz R	Drug Saf	2014
17	26606038	Predictive modeling of structured electronic health records for adverse drug event detection	BMC Med Inform Decis Mak. 2015;15 Suppl 4(Suppl 4):S1. doi: 10.1186/1472-6947-15-S4-S1. Epub 2015 Nov 25.	Zhao J	BMC Med Inform Decis Mak	2015
18	24599513	Reducing the noise in signal detection of adverse drug reactions by standardizing the background: a pilot study on analyses of proportional reporting ratios-by-therapeutic area	Eur J Clin Pharmacol. 2014 May;70(5):627-35. doi: 10.1007/s00228-014-1658-1. Epub 2014 Mar 7.	Grundmark B	Eur J Clin Pharmacol	2014
19	36380085	A real-world pharmacovigilance study of FDA Adverse Event Reporting System (FAERS) events for osimertinib	Sci Rep. 2022 Nov 15;12(1):19555. doi: 10.1038/s41598-022-23834-1.	Yin Y	Sci Rep	2022
20	31677951	ADVANCE: Towards near real-time monitoring of vaccination coverage, benefits and risks using European electronic health record databases	Vaccine. 2020 Dec 22;38 Suppl 2:B76-B83. doi: 10.1016/j.vaccine.2019.08.012. Epub 2019 Oct 31.	Bollaerts K	Vaccine	2020
21	21219405	Using primary care prescribing databases for pharmacovigilance	Br J Clin Pharmacol. 2011 Feb;71(2):244-9. doi: 10.1111/j.1365-2125.2010.03816.x.	Mohamed IN	Br J Clin Pharmacol	2011
22	35691583	Development and external validation of a 1- and 5-year fracture prediction tool based on electronic medical records data: The EPIC risk algorithm	Bone. 2022 Sep;162:116469. doi: 10.1016/j.bone.2022.116469. Epub 2022 Jun 9.	Tebé C	Bone	2022
23	31198563	Pharmacovigilance systems in resource-limited settings: an evaluative case study of Sierra Leone	J Pharm Policy Pract. 2019 Jun 11;12:13. doi: 10.1186/s40545-019-0173-2. eCollection 2019.	Abiri OT	J Pharm Policy Pract	2019
24	25123721	Big data - smart health strategies. Findings from the yearbook 2014 special theme	Yearb Med Inform. 2014 Aug 15;9(1):48-51. doi: 10.15265/IY-2014-0031.	Koutkias V	Yearb Med Inform	2014

For peer review only

No.	PMID	Title	Citation	First Author	Journal/Book	Publication Year
25	36147337	Analysis of pharmacovigilance databases for spontaneous reports of adverse drug reactions related to substandard and falsified medical products: A descriptive study	Front Pharmacol. 2022 Sep 6;13:964399. doi: 10.3389/fphar.2022.964399. eCollection 2022.	Pozsgai K	Front Pharmacol	2022
26	23331229	Automated method for detecting increases in frequency of spontaneous adverse event reports over time	J Biopharm Stat. 2013;23(1):161-77. doi: 10.1080/10543406.2013.736809.	DuMouchel W	J Biopharm Stat	2013
27	26819727	Pharmacoepidemiology in Japan: medical databases and research achievements	J Pharm Health Care Sci. 2015 May 1;1:16. doi: 10.1186/s40780-015-0016-5. eCollection 2015.	Tanaka S	J Pharm Health Care Sci	2015
28	34694230	Adverse Drug Event Prediction Using Noisy Literature-Derived Knowledge Graphs: Algorithm Development and Validation	JMIR Med Inform. 2021 Oct 25;9(10):e32730. doi: 10.2196/32730.	Dasgupta S	JMIR Med Inform	2021
29	36438789	Data mining and safety analysis of BTK inhibitors: A pharmacovigilance investigation based on the FAERS database	Front Pharmacol. 2022 Nov 11;13:995522. doi: 10.3389/fphar.2022.995522. eCollection 2022.	Wan Q	Front Pharmacol	2022
30	28595649	Data-driven prediction of adverse drug reactions induced by drug-drug interactions	BMC Pharmacol Toxicol. 2017 Jun 8;18(1):44. doi: 10.1186/s40360-017-0153-6.	Liu R	BMC Pharmacol Toxicol	2017
31	31004158	Prevalence and incidence of narcolepsy in a US health care claims database, 2008-2010	Sleep. 2019 Jul 8;42(7):zsz091. doi: 10.1093/sleep/zsz091.	Scheer D	Sleep	2019
32	31507029	Data variability across Canadian administrative health databases: Differences in content, coding, and completeness	Pharmacoepidemiol Drug Saf. 2020 Jan;29 Suppl 1:68-77. doi: 10.1002/pds.4889. Epub 2019 Sep 10.	Doyle CM	Pharmacoepidemiol Drug Saf	2020
33	32564242	Risk Factor Considerations in Statistical Signal Detection: Using Subgroup Disproportionality to Uncover Risk Groups for Adverse Drug Reactions in VigiBase	Drug Saf. 2020 Oct;43(10):999-1009. doi: 10.1007/s40264-020-00957-w.	Sandberg L	Drug Saf	2020
34	23070598	Comparison of two drug safety signals in a pharmacovigilance data mining framework	Stat Methods Med Res. 2016 Apr;25(2):615-29. doi: 10.1177/0962280212462295. Epub 2012 Oct 14.	Tubert-Bitter P	Stat Methods Med Res	2016
35	29446035	Sorting Through the Safety Data Haystack: Using Machine Learning to Identify Individual Case Safety Reports in Social-Digital Media	Drug Saf. 2018 Jun;41(6):579-590. doi: 10.1007/s40264-018-0641-7.	Comfort S	Drug Saf	2018
36	24787710	Paediatric pharmacovigilance: use of pharmacovigilance data mining algorithms for signal detection in a safety dataset of a paediatric clinical study conducted in seven African countries	PLoS One. 2014 May 1;9(5):e96388. doi: 10.1371/journal.pone.0096388. eCollection 2014.	Kajungu DK	PLoS One	2014
37	28646789	Data-mining for detecting signals of adverse drug reactions of fluoxetine using the Korea Adverse Event Reporting System (KAERS) database	Psychiatry Res. 2017 Oct;256:237-242. doi: 10.1016/j.psychres.2017.06.038. Epub 2017 Jun 13.	Kim S	Psychiatry Res	2017
38	21701609	Information technology in pharmacovigilance: Benefits, challenges, and future directions from industry perspectives	Drug Healthc Patient Saf. 2009;1:35-45. doi: 10.2147/dhps.s7180. Epub 2009 Oct 15.	Lu Z	Drug Healthc Patient Saf	2009
39	27868278	Differences in VigiBase® reporting of aminoglycoside and capreomycin-suspected ototoxicity during tuberculosis treatment	Pharmacoepidemiol Drug Saf. 2017 Jan;26(1):1-8. doi: 10.1002/pds.4125. Epub 2016 Nov 20.	Sagwa EL	Pharmacoepidemiol Drug Saf	2017
40	23921799	Triptans and serious adverse vascular events: data mining of the FDA Adverse Event Reporting System database	Cephalalgia. 2014 Jan;34(1):5-13. doi: 10.1177/0333102413499649. Epub 2013 Aug 6.	Roberto G	Cephalalgia	2014
41	31673326	Evaluation of quantitative signal detection in EudraVigilance for orphan drugs: possible risk of false negatives	Ther Adv Drug Saf. 2019 Oct 21;10:2042098619882819. doi: 10.1177/2042098619882819. eCollection 2019.	Sardella M	Ther Adv Drug Saf	2019
42	36050484	Detecting early safety signals of infliximab using machine learning algorithms in the Korea adverse event reporting system	Sci Rep. 2022 Sep 1;12(1):14869. doi: 10.1038/s41598-022-18522-z.	Lee JE	Sci Rep	2022
43	30324671	Significance of data mining in routine signal detection: Analysis based on the safety signals identified by the FDA	Pharmacoepidemiol Drug Saf. 2018 Dec;27(12):1402-1408. doi: 10.1002/pds.4672. Epub 2018 Oct 15.	Fukazawa C	Pharmacoepidemiol Drug Saf	2018
44	31156424	Computational Advances in Drug Safety: Systematic and Mapping Review of Knowledge Engineering Based Approaches	Front Pharmacol. 2019 May 17;10:415. doi: 10.3389/fphar.2019.00415. eCollection 2019.	Natsiavas P	Front Pharmacol	2019
45	28335691	Cases of drug-induced Torsade de Pointes: a review of Belgian cases in the EudraVigilance database	Acta Clin Belg. 2017 Dec;72(6):385-390. doi: 10.1080/17843286.2017.1300217. Epub 2017 Mar 24.	Vandael E	Acta Clin Belg	2017
46	16134080	Signal detection in pharmacovigilance: empirical evaluation of data mining tools	Pharmacoepidemiol Drug Saf. 2005 Sep;14(9):597-9. doi: 10.1002/pds.1128.	Chan KA	Pharmacoepidemiol Drug Saf	2005
47	35745593	Pharmacovigilance Signals of the Opioid Epidemic over 10 Years: Data Mining Methods in the Analysis of Pharmacovigilance Datasets Collecting Adverse Drug Reactions (ADRs) Reported to EudraVigilance (EV) and the FDA Adverse Event Reporting System (FAERS)	Pharmaceuticals (Basel). 2022 May 27;15(6):675. doi: 10.3390/ph15060675.	Chiappini S	Pharmaceuticals (Basel)	2022

For peer review only

No.	PMID	Title	Citation	First Author	Journal/Book	Publication Year
48	36050787	Challenges and opportunities for mining adverse drug reactions: perspectives from pharma, regulatory agencies, healthcare providers and consumers	Database (Oxford). 2022 Sep 2;2022:baac071. doi: 10.1093/database/baac071.	Gonzalez-Hernandez G	Database (Oxford)	2022
49	33518634	[Role and Applicability of Spontaneous Reporting Databases in Medical Big Data]	Yakugaku Zasshi. 2021;141(2):165-168. doi: 10.1248/yakushi.20-00196-1.	Sakai T	Yakugaku Zasshi	2021
50	32169289	Uses of pharmacovigilance databases: An overview	Therapie. 2020 Nov-Dec;75(6):591-598. doi: 10.1016/j.therap.2020.02.022. Epub 2020 Feb 26.	Bihan K	Therapie	2020
51	35904529	The Use of Artificial Intelligence in Pharmacovigilance: A Systematic Review of the Literature	Pharmaceut Med. 2022 Oct;36(5):295-306. doi: 10.1007/s40290-022-00441-z. Epub 2022 Jul 29.	Salas M	Pharmaceut Med	2022
52	31623850	Pharmacovigilance - The next chapter	Therapie. 2019 Dec;74(6):557-567. doi: 10.1016/j.therap.2019.09.004. Epub 2019 Oct 1.	Moore N	Therapie	2019
53	27224991	[Pharmacovigilance in Germany : It is about time]	Internist (Berl). 2016 Jun;57(6):616-23. doi: 10.1007/s00108-016-0068-0.	Douros A	Internist (Berl)	2016
54	33598764	Construction and analysis of a database for medication errors in a pharmacovigilance centre-the Moroccan experience	Eur J Clin Pharmacol. 2021 Aug;77(8):1235-1246. doi: 10.1007/s00228-021-03109-w. Epub 2021 Feb 17.	Alj L	Eur J Clin Pharmacol	2021
55	35788724	Evolution of adverse drug reactions reporting systems: paper based to software based	Eur J Clin Pharmacol. 2022 Sep;78(9):1385-1390. doi: 10.1007/s00228-022-03358-3. Epub 2022 Jul 5.	Madhushika MT	Eur J Clin Pharmacol	2022
56	23640186	The past, present and perhaps future of pharmacovigilance: homage to Folke Sjoqvist	Eur J Clin Pharmacol. 2013 May;69 Suppl 1:33-41. doi: 10.1007/s00228-013-1486-8. Epub 2013 May 3.	Moore N	Eur J Clin Pharmacol	2013
57	35579806	Artificial Intelligence in Pharmacovigilance: An Introduction to Terms, Concepts, Applications, and Limitations	Drug Saf. 2022 May;45(5):407-418. doi: 10.1007/s40264-022-01156-5. Epub 2022 May 17.	Aronson JK	Drug Saf	2022
58	36251280	Development of a multivariate prediction model to identify individual case safety reports which require clinical review	Pharmacoepidemiol Drug Saf. 2022 Dec;31(12):1300-1307. doi: 10.1002/pds.5553. Epub 2022 Oct 21.	Gosselt HR	Pharmacoepidemiol Drug Saf	2022
59	26575203	Big Data and Adverse Drug Reaction Detection	Clin Pharmacol Ther. 2016 Mar;99(3):268-70. doi: 10.1002/cpt.302. Epub 2015 Dec 23.	Harpaz R	Clin Pharmacol Ther	2016
60	32176553	The potential role of big data in the detection of adverse drug reactions	Expert Rev Clin Pharmacol. 2020 Mar;13(3):201-204. doi: 10.1080/17512433.2020.1740086. Epub 2020 Mar 16.	Sultana J	Expert Rev Clin Pharmacol	2020
61	24698186	[Terappel: description of a computerised database and examples of studies]	Therapie. 2014 Jan-Feb;69(1):31-8. doi: 10.2515/therapie/2014013. Epub 2014 Apr 4.	Vial T	Therapie	2014
62	28840504	From Big Data to Smart Data for Pharmacovigilance: The Role of Healthcare Databases and Other Emerging Sources	Drug Saf. 2018 Feb;41(2):143-149. doi: 10.1007/s40264-017-0592-4.	Trifirò G	Drug Saf	2018
63	22315152	Electronic healthcare databases for active drug safety surveillance: is there enough leverage?	Pharmacoepidemiol Drug Saf. 2012 Jun;21(6):611-21. doi: 10.1002/pds.3197. Epub 2012 Feb 8.	Coloma PM	Pharmacoepidemiol Drug Saf	2012
64	19745234	The EU-ADR project: preliminary results and perspective	Stud Health Technol Inform. 2009;148:43-9.	Trifiro G	Stud Health Technol Inform	2009
65	26092163	[Benefits of large healthcare databases for drug risk research]	Bundesgesundheitsblatt Gesundheitsforschung Gesundheitsschutz. 2015 Aug;58(8):829-837. doi: 10.1007/s00103-015-2185-7.	Garbe E	Bundesgesundheitsblatt Gesund	2015
66	23670805	Pilot evaluation of an automated method to decrease false-positive signals induced by co-prescriptions in spontaneous reporting databases	Pharmacoepidemiol Drug Saf. 2014 Feb;23(2):186-94. doi: 10.1002/pds.3454. Epub 2013 May 14.	Avillach P	Pharmacoepidemiol Drug Saf	2014
67	23208789	The EU-ADR Web Platform: delivering advanced pharmacovigilance tools	Pharmacoepidemiol Drug Saf. 2013 May;22(5):459-67. doi: 10.1002/pds.3375. Epub 2012 Dec 4.	Oliveira JL	Pharmacoepidemiol Drug Saf	2013
68	31912439	Towards Automating Adverse Event Review: A Prediction Model for Case Report Utility	Drug Saf. 2020 Apr;43(4):329-338. doi: 10.1007/s40264-019-00897-0.	Muñoz MA	Drug Saf	2020
69	22873501	Implemented data mining and signal management systems on spontaneous reporting systems' databases and their availability to the scientific community - a systematic review	Curr Drug Saf. 2012 Apr;7(2):170-5. doi: 10.2174/157488612802715645.	de Almeida Vieira Lima LM	Curr Drug Saf	2012
70	22549283	Novel data-mining methodologies for adverse drug event discovery and analysis	Clin Pharmacol Ther. 2012 Jun;91(6):1010-21. doi: 10.1038/clpt.2012.50.	Harpaz R	Clin Pharmacol Ther	2012
71	18523760	Pharmacovigilance: methods, recent developments and future perspectives	Eur J Clin Pharmacol. 2008 Aug;64(8):743-52. doi: 10.1007/s00228-008-0475-9. Epub 2008 Jun 4.	Härmark L	Eur J Clin Pharmacol	2008
72	23072620	Global patterns of adverse drug reactions over a decade: analyses of spontaneous reports to VigiBase™	Drug Saf. 2012 Dec 1;35(12):1171-82. doi: 10.1007/BF03262002.	Aagaard L	Drug Saf	2012

For peer review only

No.	PMID	Title	Citation	First Author	Journal/Book	Publication Year
73	15366975	The general practice research database: role in pharmacovigilance	Drug Saf. 2004;27(12):871-81. doi: 10.2165/00002018-200427120-00004.	Wood L	Drug Saf	2004
74	23761351	Detection of adverse drug reactions using hospital databases-a nationwide study in Portugal	Pharmacoepidemiol Drug Saf. 2013 Aug;22(8):907-13. doi: 10.1002/pds.3468. Epub 2013 Jun 13.	Miguel A	Pharmacoepidemiol Drug Saf	2013
75	15366974	Data quality management in pharmacovigilance	Drug Saf. 2004;27(12):857-70. doi: 10.2165/00002018-200427120-00003.	Lindquist M	Drug Saf	2004
76	15460169	Pharmacovigilance in the 21st century: new systematic tools for an old problem	Pharmacotherapy. 2004 Sep;24(9):1099-104. doi: 10.1592/phco.24.13.1099.38090.	Szarfman A	Pharmacotherapy	2004
77	16111454	The role of data mining in pharmacovigilance	Expert Opin Drug Saf. 2005 Sep;4(5):929-48. doi: 10.1517/14740338.4.5.929.	Hauben M	Expert Opin Drug Saf	2005
78	15221098	[Databases as a source for monitoring systems of drug safety]	Bundesgesundheitsblatt Gesundheitsforschung Gesundheitsschutz. 2004 Jun;47(6):513-7. doi: 10.1007/s00103-004-0834-3.	Pigeot I	Bundesgesundheitsblatt Gesund	2004
79	20721787	Mining pharmacovigilance data using Bayesian logistic regression with James-Stein type shrinkage estimation	J Biopharm Stat. 2010 Sep;20(5):998-1012. doi: 10.1080/10543401003619056.	An L	J Biopharm Stat	2010
80	16180934	Data mining in pharmacovigilance: the need for a balanced perspective	Drug Saf. 2005;28(10):835-42. doi: 10.2165/00002018-200528100-00001.	Hauben M	Drug Saf	2005
81	17604418	Signal detection in the pharmaceutical industry: integrating clinical and computational approaches	Drug Saf. 2007;30(7):627-30. doi: 10.2165/00002018-200730070-00012.	Hauben M	Drug Saf	2007
82	15360888	A knowledge based approach for automated signal generation in pharmacovigilance	Stud Health Technol Inform. 2004;107(Pt 1):626-30.	Henegar C	Stud Health Technol Inform	2004
83	12650632	Application of quantitative signal detection in the Dutch spontaneous reporting system for adverse drug reactions	Drug Saf. 2003;26(5):293-301. doi: 10.2165/00002018-200326050-00001.	van Puijenbroek E	Drug Saf	2003
84	Yuan Luo	Natural Language Processing for EHR-Based Pharmacovigilance: A Structured Review	Evanston USA	Yuan Luo	This article is a comprehensive	2017
85	ECRI INST	Wrong-Record, Wrong-Data Errors with Health IT Systems.	Pennsylvania	ECRI INSTITUTE PSO	We define data integrity failure	2015
86	European M	Guideline on good pharmacovigilance practices (GVP) Module IX Addendum I – Methodological aspects of signal detection from spontaneous reports of suspected adverse reactions	Netherlands	European Medicines Agency	The decision based on the asses	2015
87	FDA	Questions and Answers on FDA's Adverse Event Reporting System (FAERS)	USA	FDA	What is FAERS? The FDA Ad	2018
88	Liu M et al	Comparative analysis of pharmacovigilance methods in the detection of adverse drug reactions using electronic medical records	New Jersey	Liu M et al	Medication safety requires that	2013
89	Norén GN	A hit-miss model for duplicate detection in the WHO drug safety database	Sweden.	Norén GN et al	We present two new generalisa	2005
90	Niklas Nor	A hit-miss model for duplicate detection in the WHO drug safety database		Niklas Norén et al	We demonstrate the effectivene	2005
91	eudrac	https://www.eudrac.com/news/good-pharmacovigilance-practice-guideline-updates	Germany			
92	PV Drug S	https://www.linkedin.com/posts/pv-drug-safety-academy-841528223_pharmacovigilance-activity-7071440228577660928-KRp9/	England			
93	Drug Regu	https://www.dra.gov.pk/wp-content/uploads/2022/04/Good-Pharmacovigilance-Guidelines-for-Registration-Holders.pdf	Pakistan			
94	cod-researc	https://cod-research.com/ease-icsr-handling-through-early-duplicate-identification-and-its-management/	India			
95	ECA Foun	https://www.gmp-compliance.org/guidelines/gmp-guideline/eudralex-volume-9-gvp-module-vi-addendum-i-duplicate-management-of-suspected-adverse-reaction-reports	Germany			
96	Applied Cl	https://www.appliedclinicaltrialsonline.com/view/pharmacovigilance-literature-monitoring-best-practices	Fort Washington USA			
97	ClinosolInc	https://www.slideshare.net/ClinosolIndia/duplicate-case	India			
99	clarivate	https://clarivate.com/blog/seeing-double-eliminating-duplicate-references-in-drug-safety-literature-screening/	Jersey			

For peer review only

Preferred Reporting Items for Systematic reviews and Meta-Analyses extension for Scoping Reviews (PRISMA-ScR) Checklist

SECTION	ITEM	PRISMA-ScR CHECKLIST ITEM	REPORTED ON PAGE #
**TITLE**			
Title	1	Identify the report as a scoping review.	1
**ABSTRACT**			
Structured summary	2	Provide a structured summary that includes (as applicable): background, objectives, eligibility criteria, sources of evidence, charting methods, results, and conclusions that relate to the review questions and objectives.	2
**INTRODUCTION**			
Rationale	3	Describe the rationale for the review in the context of what is already known. Explain why the review questions/objectives lend themselves to a scoping review approach.	4,5
Objectives	4	Provide an explicit statement of the questions and objectives being addressed with reference to their key elements (e.g., population or participants, concepts, and context) or other relevant key elements used to conceptualize the review questions and/or objectives.	5
**METHODS**			
Protocol and registration	5	Indicate whether a review protocol exists; state if and where it can be accessed (e.g., a Web address); and if available, provide registration information, including the registration number.	5
Eligibility criteria	6	Specify characteristics of the sources of evidence used as eligibility criteria (e.g., years considered, language, and publication status), and provide a rationale.	5
Information sources*	7	Describe all information sources in the search (e.g., databases with dates of coverage and contact with authors to identify additional sources), as well as the date the most recent search was executed.	6
Search	8	Present the full electronic search strategy for at least 1 database, including any limits used, such that it could be repeated.	20
Selection of sources of evidence†	9	State the process for selecting sources of evidence (i.e., screening and eligibility) included in the scoping review.	18
Data charting process‡	10	Describe the methods of charting data from the included sources of evidence (e.g., calibrated forms or forms that have been tested by the team before their use, and whether data charting was done independently or in duplicate) and any processes for obtaining and confirming data from investigators.	6
Data items	11	List and define all variables for which data were sought and any assumptions and simplifications made.	23
Critical appraisal of individual sources of evidence§	12	If done, provide a rationale for conducting a critical appraisal of included sources of evidence; describe the methods used and how this information was used in any data synthesis (if appropriate).	na

St. Michael's
Inspired Care.
Inspiring Science.

For peer review only - http://bmjopen.bmj.com/site/about/guidelines.xhtml

SECTION	ITEM	PRISMA-ScR CHECKLIST ITEM	REPORTED ON PAGE #
Synthesis of results	13	Describe the methods of handling and summarizing the data that were charted.	6-7
**RESULTS**			
Selection of sources of evidence	14	Give numbers of sources of evidence screened, assessed for eligibility, and included in the review, with reasons for exclusions at each stage, ideally using a flow diagram.	7
Characteristics of sources of evidence	15	For each source of evidence, present characteristics for which data were charted and provide the citations.	7
Critical appraisal within sources of evidence	16	If done, present data on critical appraisal of included sources of evidence (see item 12).	na
Results of individual sources of evidence	17	For each included source of evidence, present the relevant data that were charted that relate to the review questions and objectives.	7-10
Synthesis of results	18	Summarize and/or present the charting results as they relate to the review questions and objectives.	7-10
**DISCUSSION**			
Summary of evidence	19	Summarize the main results (including an overview of concepts, themes, and types of evidence available), link to the review questions and objectives, and consider the relevance to key groups.	11-13
Limitations	20	Discuss the limitations of the scoping review process.	na
Conclusions	21	Provide a general interpretation of the results with respect to the review questions and objectives, as well as potential implications and/or next steps.	13
**FUNDING**			
Funding	22	Describe sources of funding for the included sources of evidence, as well as sources of funding for the scoping review. Describe the role of the funders of the scoping review.	13

JBI = Joanna Briggs Institute; PRISMA-ScR = Preferred Reporting Items for Systematic reviews and Meta-Analyses extension for Scoping Reviews.
* Where *sources of evidence* (see second footnote) are compiled from, such as bibliographic databases, social media platforms, and Web sites.
† A more inclusive/heterogeneous term used to account for the different types of evidence or data sources (e.g., quantitative and/or qualitative research, expert opinion, and policy documents) that may be eligible in a scoping review as opposed to only studies. This is not to be confused with *information sources* (see first footnote).
‡ The frameworks by Arksey and O'Malley (6) and Levac and colleagues (7) and the JBI guidance (4, 5) refer to the process of data extraction in a scoping review as data charting.
§ The process of systematically examining research evidence to assess its validity, results, and relevance before using it to inform a decision. This term is used for items 12 and 19 instead of "risk of bias" (which is more applicable to systematic reviews of interventions) to include and acknowledge the various sources of evidence that may be used in a scoping review (e.g., quantitative and/or qualitative research, expert opinion, and policy document).

From: Tricco AC, Lillie E, Zarin W, O'Brien KK, Colquhoun H, Levac D, et al. PRISMA Extension for Scoping Reviews (PRISMAScR): Checklist and Explanation. Ann Intern Med. 2018;169:467–473. doi: 10.7326/M18-0850.

For peer review only - http://bmjopen.bmj.com/site/about/guidelines.xhtml

BMJ Open

Navigating Duplication in Pharmacovigilance Databases: A Scoping Review

Journal:	*BMJ Open*
Manuscript ID	bmjopen-2023-081990.R2
Article Type:	Original research
Date Submitted by the Author:	08-Apr-2024
Complete List of Authors:	Kiguba, Ronald; Makerere University, Department of Pharmacology and Therapeutics<br>Isabirye, Gerald; National Drug Authority<br>Mayengo, Julius; National Drug Authority<br>Owiny, Jonathan; National Drug Authority<br>Tregunno, Phil; Medicines and Healthcare Products Regulatory Agency<br>Harrison, Kendal; Medicines and Healthcare Products Regulatory Agency<br>Pirmohamed, Munir; University of Liverpool Institute of Integrative Biology<br>Ndagije, Helen ; National Drug Authority
**Primary Subject Heading**:	Pharmacology and therapeutics
Secondary Subject Heading:	Epidemiology, Public health
Keywords:	Adverse events < THERAPEUTICS, Pharmacology < TROPICAL MEDICINE, CLINICAL PHARMACOLOGY

SCHOLARONE™
Manuscripts

I, the Submitting Author has the right to grant and does grant on behalf of all authors of the Work (as defined in the below author licence), an exclusive licence and/or a non-exclusive licence for contributions from authors who are: i) UK Crown employees; ii) where BMJ has agreed a CC-BY licence shall apply, and/or iii) in accordance with the terms applicable for US Federal Government officers or employees acting as part of their official duties; on a worldwide, perpetual, irrevocable, royalty-free basis to BMJ Publishing Group Ltd ("BMJ") its licensees and where the relevant Journal is co-owned by BMJ to the co-owners of the Journal, to publish the Work in this journal and any other BMJ products and to exploit all rights, as set out in our licence.

The Submitting Author accepts and understands that any supply made under these terms is made by BMJ to the Submitting Author unless you are acting as an employee on behalf of your employer or a postgraduate student of an affiliated institution which is paying any applicable article publishing charge ("APC") for Open Access articles. Where the Submitting Author wishes to make the Work available on an Open Access basis (and intends to pay the relevant APC), the terms of reuse of such Open Access shall be governed by a Creative Commons licence – details of these licences and which Creative Commons licence will apply to this Work are set out in our licence referred to above.

Other than as permitted in any relevant BMJ Author's Self Archiving Policies, I confirm this Work has not been accepted for publication elsewhere, is not being considered for publication elsewhere and does not duplicate material already published. I confirm all authors consent to publication of this Work and authorise the granting of this licence.

For peer review only

Navigating Duplication in Pharmacovigilance Databases: A Scoping Review

Authors:

Ronald Kiguba[1], Gerald Isabirye[2], Julius Mayengo[2], Jonathan Owiny[2], Phil Tregunno[3], Kendal Harrison[3], Munir Pirmohamed[4], Helen B. Ndagije[2]*

*Correspondence to: Ronald Kiguba (Tel: +256 701 840683; Email: kiguba@gmail.com)

Affiliations:

[1]Department of Pharmacology and Therapeutics, College of Health Sciences, Makerere University, Kampala, Uganda

[2]National Pharmacovigilance Centre, National Drug Authority, Kampala, Uganda

[3]Safety and Surveillance Group, Medicines and Healthcare products Regulatory Agency, London, United Kingdom

[4]Centre for Drug Safety Science and Wolfson Centre for Personalised Medicine, Institute of Systems, Molecular and Integrative Biology, University of Liverpool, Liverpool, United Kingdom

Running Head:

Duplication in Pharmacovigilance Databases

For peer review only - http://bmjopen.bmj.com/site/about/guidelines.xhtml

Abstract

Objectives:

Pharmacovigilance databases play a critical role in monitoring drug safety. The duplication of reports in pharmacovigilance databases, however, undermines their data integrity. This scoping review sought to provide a comprehensive understanding of duplication in pharmacovigilance databases worldwide.

Design:

A scoping review.

Data sources:

Reviewers comprehensively searched the literature in PubMed, Web of Science, Wiley Online Library, EBSCOhost, Google Scholar, and other relevant websites.

Eligibility criteria:

Peer-reviewed publications and grey literature, without language restriction, describing duplication and/or methods relevant to duplication in pharmacovigilance databases from inception to 1 September 2023.

Data extraction and synthesis:

We used the Joanna Briggs Institute guidelines for scoping reviews and conformed with the Preferred Reporting Items for Systematic Reviews and Meta-Analyses Extension for Scoping Reviews. Two reviewers independently screened titles, abstracts, and full-texts. One reviewer extracted the data and performed descriptive analysis, which the second reviewer assessed. Disagreements were resolved by discussion and consensus or in consultation with a third reviewer.

Results:

We screened 22745 unique titles and 156 were eligible for full-text review. Of the 156 titles, 58 (47 peer-reviewed; 11 grey literature) fulfilled the inclusion criteria for the scoping review. Included titles addressed the extent (5 papers), prevention strategies (15 papers), causes (32 papers), detection methods (25 papers), management strategies (24 papers), and implications (14 papers) of duplication in pharmacovigilance databases. The papers overlapped, discussing more than one field. Advances in artificial intelligence, particularly natural language processing, hold promise in enhancing the efficiency and precision of deduplication of large and complex pharmacovigilance databases.

Conclusion:

Duplication in pharmacovigilance databases compromises risk assessment and decision-making, potentially threatening patient safety. Therefore, efficient duplicate prevention, detection, and management is essential for more reliable pharmacovigilance data. To minimise duplication, consistent use of worldwide unique identifiers as the key case-identifiers is recommended alongside recent advances in artificial intelligence.

Keywords: Adverse event reporting, Duplication, Duplicate management, Pharmacovigilance, Pharmacovigilance database.

For peer review only

Article summary

Strengths and limitations of this study:

- The study involved an extensive literature search on a global scale including both peer-reviewed publications and the grey literature.
- This review adhered to the rigorous methodology published by the Joanna Briggs Institute for scoping reviews.
- We employed an *a priori* PubMed search strategy, which was then adapted across other peer-reviewed research databases (Web of Science, Wiley Online Library, EBSCOhost) using the Polyglot Search Translator. Additionally, our search for grey literature followed the guidelines outlined in the Canadian Agency for Drugs and Technologies in Health Guide.
- The risk of bias or quality assessment of included studies was not conducted in keeping with the design for scoping reviews, which limited the ability to document the methodological rigor of the included studies.

For peer review only

For peer review only - http://bmjopen.bmj.com/site/about/guidelines.xhtml

INTRODUCTION:

Pharmacovigilance is crucial for drug safety as it promotes the prevention, detection, and evaluation of suspected adverse reactions to minimize their impact on patient health.[1, 2] One of the challenges of pharmacovigilance is the duplication of cases in pharmacovigilance databases, which distorts drug safety and efficacy assessment, and disrupts decision-making.[3-5] Duplication is defined as multiple, unconnected records that refer to the same potential adverse event.[3, 4, 6] From 2000 to 2010, about 2.5% of reports with adequate information for duplicate analysis in the World Health Organization (WHO) global pharmacovigilance database were duplicates; the percentage was higher for reports from the literature (11%) and those with fatal outcomes (5%).[4] To promote the accuracy of pharmacovigilance databases, it is important to routinely screen and remove duplicate cases.[4]

The duplication of cases should, however, be differentiated from the replication of records. Replication includes reproducing or recreating pharmacovigilance reports for various purposes, e.g., research, regulatory compliance, or data analysis. Replication involves making copies of reports for different stakeholders or systems without necessarily meaning that the content or details of the reports are identical. Replication could result in duplication if multiple reporters, e.g., pharmaceutical companies, report the same case to the same regulatory authority for compliance reasons.[7]

Global, regional, and country-level guidelines have been implemented to address duplication in pharmacovigilance databases. Global pharmacovigilance initiatives, such as the International Council for Harmonization (ICH) guidelines and the Uppsala Monitoring Centre, which coordinates the WHO Programme for International Drug Monitoring, promote standardization and harmonization of pharmacovigilance activities, that are essential for accurate and efficient data collection, analysis, and reporting.[2, 8-11] In the US, the Food and Drug Administration (FDA) uses the Adverse Event Reporting System (FAERS) to manage adverse event reports, including duplicates.[12, 13] In Europe, the European Medicines Agency (EMA) oversees the safety of medicines via the EudraVigilance system which uses unique identification numbers for each report and has a built-in deduplication mechanism to identify and eliminate duplicate reports.[14, 15] In Asia, a deduplication technique was adopted by the Pharmacovigilance Program of India, together with the assignment of unique identification numbers and data standardization. Similar to this, in China, the National Medical Products Administration has put in place a system called the Adverse Drug Reaction Monitoring and Re-evaluation System that incorporates a deduplication mechanism for reporting and monitoring suspected adverse reactions.[16] In Africa, WHO built a regional pharmacovigilance centre in Ghana to support the region's pharmacovigilance systems through training, including duplicate management.[17] Pharmaceutical companies are also responsible for managing duplicate reports. Comprehensive and integrated pharmacovigilance systems are required to effectively manage data from multiple sources, e.g. electronic health records, spontaneous reporting databases, social media, and digital devices, among others; and to eliminate duplicate reports.[18, 19] These systems should ensure compliance with the global pharmacovigilance regulations and standards.

Duplicates arise from various causes including data entry errors,[20, 21] multiple reporters and reporting channels,[22, 23] pressure from mandatory reporting requirements for healthcare professionals and pharmaceutical companies,[4, 24] challenges in data integration and harmonization in pharmacovigilance databases[19], multiple representation of the same data due to the merging of pharmacovigilance databases,[6] lack of standardized data management practices,[6] and incomplete case information of key data elements.[12, 25]

For peer review only - http://bmjopen.bmj.com/site/about/guidelines.xhtml

The implications of duplication are far-reaching, hence the need for the routine screening and elimination of duplicates from pharmacovigilance databases.[6] Software programs significantly improve efficiency, accuracy, and consistency in the detection and management of duplicates. The Uppsala Monitoring Centre developed the software VigiMatch to detect potential duplicates in VigiBase, the global pharmacovigilance database.[26] VigiBase can merge duplicate reports identified by VigiMatch.[4, 27] Automated standalone software programs have been developed to handle large volumes of data and can be customized to meet specific regulatory requirements.[28-30]

Advances in technology, e.g., machine learning and natural language processing hold promise in augmenting the efficiency and precision of pharmacovigilance efforts, particularly in the deduplication of large and complex pharmacovigilance databases.[5],[31, 32] Deduplication methods are, however, impeded by the high extent of incomplete case information which makes it difficult to conduct reliable duplicate analysis.[4] Moreover, systems ought to avoid over-reliance on automated standalone software programs as they could generate false signals. Instead, holistic methods which include manual review alongside automated processes should be preferred.[33, 34] The objective of this scoping review is to provide a comprehensive overview of duplication in pharmacovigilance databases on a global scale. This review addresses the extent, causes, prevention strategies, detection methods, management and impact of duplication in pharmacovigilance databases.

METHODS

Research Questions:

1. What is the extent of duplication in pharmacovigilance databases worldwide?
2. What are the causes of, and prevention strategies, detection methods and management strategies for duplicate reports in pharmacovigilance databases worldwide?
3. What is the impact of duplicate reports in pharmacovigilance databases worldwide?

Study Design:

This scoping review was conducted in accordance with the Joanna Briggs Institute (JBI) guidelines for scoping reviews[35] and conformed with the Preferred Reporting Items for Systematic Reviews and Meta-Analyses Extension for Scoping Reviews (PRISMA-ScR).[36] The protocol was developed according to the JBI scoping review template and registered with Open Science Framework (DOI 10.17605/OSF.IO/PBVF3).

Definition of duplication:

Duplication was defined as multiple, unconnected records that refer to the same potential adverse event.[3, 4, 6]

Eligibility Criteria:

The inclusion criteria for selection of articles, reports, and guidelines, without language restriction, were:

1. Articles, reports, and guidelines related to duplication and/ or methods relevant to duplication in pharmacovigilance databases
2. Articles published in peer-reviewed journals and grey literature from official websites of regulatory authorities and pharmacovigilance organizations.

For peer review only - http://bmjopen.bmj.com/site/about/guidelines.xhtml

For peer review only

Articles, reports, and guidelines were excluded if, on full-text review, they did not align with the precise objectives of the scoping review. This rigorous screening procedure ensured that only relevant papers were included in the analysis.

For peer review only

For peer review only - http://bmjopen.bmj.com/site/about/guidelines.xhtml

Search strategy:

The search strategy was designed to identify all relevant studies related to the research question(s). We therefore searched multiple databases, including PubMed, Web of Science, Wiley Online Library, EBSCOhost, Google Scholar and other relevant websites including regulatory authorities and pharmacovigilance organizations, from inception to 1 September 2023. The reference lists of relevant studies were also screened. The PubMed search string for peer-reviewed publications is detailed in **Appendix I** and was adapted across the other peer-reviewed research databases using the Polyglot Search Translator.[37] The grey literature search was based on the Canadian Agency for Drugs and Technologies in Health Guide[38] (see **Appendix II)**.

Study selection:

Two reviewers independently screened the titles and abstracts of all the identified articles for eligibility. Full-text articles were retrieved for all potentially eligible studies and independently assessed for inclusion by two reviewers. Disagreements were resolved by discussion and consensus or in consultation with a third reviewer.

Data extraction:

Data extraction was done by Gerald Isabirye (GI) using a tool created by the reviewers, as detailed in **Appendix III**. This tool was designed to capture various parameters, including the Author, Year of publication, Journal, Country, Title, Specific Objectives, Study design, Categories (extent, prevention, causes, detection, management and impact of duplication in pharmacovigilance databases), and key findings. The extracted data were reviewed by Ronald Kiguba (RK) and a third reviewer, Helen B. Ndagije (HBN), and revised accordingly.

Data analysis and presentation:

The extracted data are provided in a descriptive summary according to the review's objectives. The risk of bias or quality assessment was not done across the included studies. This decision aligns with the guidelines outlined by the JBI because a scoping review is not intended to appraise the risk of bias of a cumulative body of evidence.[36] Instead, it focuses on providing a comprehensive overview of the existing literature.

Patient and public involvement:

Patients and the public did not participate in the design, conduct, reporting and drafting of the dissemination plans of this research.

Regions and countries:

The regions and countries of papers included in this review are presented in **Appendix IV**. The papers were from the Asia Pacific region, Europe, Australia and North Africa.

For peer review only - http://bmjopen.bmj.com/site/about/guidelines.xhtml

RESULTS

We screened 22745 unique titles and 156 (127 peer-reviewed; 29 grey literature) fulfilled the inclusion criteria for the scoping review (see **Table 1, Figure 1** and **supplementary Table 1**). were eligible for full-text review. Of the 156 titles, 58 (47 peer-reviewed; 11 grey literature) fulfilled the inclusion criteria for the scoping review (see **Table 1, Figure 1** and **supplementary Table 1**). The included titles addressed the extent of duplication (5 papers), prevention strategies (15 papers), causes (32 papers), detection methods (25 papers), management (24 papers) and implications (14 papers) of duplicate reports. The papers overlapped, discussing more than one field. The list of excluded titles is provided in **Appendix V**.

Extent of duplication: The prevalence of duplication in the WHO global pharmacovigilance database, VigiBase, was 2.5% for reports submitted from 2000 to 2010, based on half [51%, 1.9 million of 3.7 million) of the reports with sufficient information for duplicate analysis.[4] Duplication rates were highest for reports from the published literature, e.g., peer-reviewed articles (11%), and fatal outcomes (5%).[4] In Europe, the prevalence of duplication in VigiBase was quite high for some countries [e.g., Czech Republic (15%); Austria (15%); Switzerland (4.7%)] and low for others [e.g., United Kingdom (1.4%); Denmark (1%); Spain (0.7%)].[4] In Asia, the extent of duplication in VigiBase was high in Korea (9.2%) and low in Japan (0.8%). In the US, duplicates represented 5-20% of reports in the FAERS by the year 2000.[13] A very high level of duplication, from 66% to 87%, most likely fuelled by replication, was observed in the FAERS database for reports from the literature during the first quarter of 2021.[39] In specific drug-event pair analysis, duplicates accounted for 8% (287/3572) of clozapine-myocarditis cases in VigiBase from inception to January 2021 and for 20% (28/141) of quinine-induced thrombocytopenia cases in the FAERS from 1974 to 2000.[40, 41] In Africa, information was scarce on the extent of duplication in pharmacovigilance databases.

Causes of duplication: Duplicates in pharmacovigilance databases arise from various causes including: 1) data entry errors, e.g. i) submission of follow-up reports of the same case with new identification numbers,[4] ii) multiple entries of the same case by different data entrants,[21, 42] and iii) misspelling the name of a drug or patient;[21] 2) multiple reporters e.g. patients, healthcare professionals or pharmaceutical companies;[10, 22, 42, 43] 3) pressure from mandatory reporting requirements for healthcare professionals and pharmaceutical companies;[4, 24, 44, 45] 4) challenges in data integration and harmonization in database software, e.g. technical glitches that could occur during i) data synchronization, ii) data import/export, or iii) the integration of different databases;[26, 46] 5) multiple representation of the same data due to the merging of data from different databases such as regulatory authorities, pharmaceutical companies and clinical trials;[6] 6) lack of standardized data management practices characterized by inconsistencies in data entry standards and coding systems,[6] and 7) incomplete or missing case information of key data elements, e.g. patient identifiers.[25, 46, 47]

Strategies to minimize duplication: Several papers described best practices for minimizing duplication. These strategies include standardized data entry to enable consistent recording of adverse event data, making it easier to identify duplicates;[46, 48, 49] assigning unique identifiers to individual case safety reports, such as the worldwide unique case identification number, which should not be changed during electronic transmission and re-transmission of safety reports;[3, 6, 50] regular data cleaning and deduplication[48]; automated and semi-automated search criteria based on similarities in patient demographics (age, gender), unique identifiers, adverse reactions, and suspected medicines;[4, 6, 48, 51] data validation and quality assurance processes;[52, 53] collaboration between health facilities, pharmaceutical companies, and patients;[54, 55] and, data sharing between regulatory authorities and international databases.[54, 55]

For peer review only - http://bmjopen.bmj.com/site/about/guidelines.xhtml

Detection of duplicates: Twenty-four papers discussed the detection of duplicates. Pharmacovigilance databases should be reviewed routinely for duplicates and screening for duplicates should be a priority when new reports are submitted.[6, 56] Automated or semi-automated detection systems are recommended to identify duplicates during both manual and electronic data capture.[6, 48] To detect duplicates in small databases, reports may be sorted into simple tables by case ID numbers, patient characteristics (age, sex), adverse reaction(s) or suspected medicine(s). For large databases, however, the flagging of potential duplicates is largely automated, and is frequently followed up with manual review particularly to confirm inconclusive duplicates detected by the automated methods.[29] Duplicate detection algorithms should, however, be validated and fine-tuned periodically because the level of detail in submitted reports could vary with time, e.g. if data fields become mandatory, these variables ought to be included in the algorithms.[6, 26, 57]

Approaches to duplicate detection:

Exact matching: This is a straightforward method which compares two records to identify precise matches (exact duplicates) based on key data fields such as the report ID, patient demographics, name of adverse event, onset date of the adverse event, and name of suspected drug, among others.[58, 59]

Rule-based matching: This method defines specific conditions to identify and eliminate potential duplicates based on predefined criteria informed by domain knowledge, historical data and observed patterns in the data. It allows for more targeted identification of duplicates based on known patterns, e.g., the same case with the same adverse event reported several times in a short period.[60] These rules are typically based on particular fields in the data, e.g. patient's name, date of birth, or drug name, and are intended to match reports that have similar data.[61, 62] Simple duplicates can often be found using rule-based systems, but more complex duplicates may be missed because they require more complicated algorithms.[60]

Fuzzy matching: This technique identifies records that are similar though not necessarily identical. Unlike exact matching, which requires an exact match between data fields, fuzzy matching allows for variations, misspellings, and small differences in the data. This is particularly useful where the data may be inconsistently entered or contain errors, frequently in real-world databases.[63] Fuzzy matching algorithms assess the degree of resemblance between two records using a variety of similarity metrics, including Levenshtein distance and Jaro-Winkler distance. Fuzzy matching algorithms are frequently used in pharmacovigilance to identify duplicates that contain distinct patient names, medical terms, or drug names spelled differently or abbreviated differently.[63]

Machine learning and natural language processing: This approach employs supervised machine learning models to train algorithms using labelled data that differentiate duplicate and non-duplicate pairs. These models then use the knowledge gained from labelled data to predict whether new record pairs are duplicates or not based on learned patterns.[5, 64],[65, 66] Natural language processing techniques have been developed to augment computerised duplicate detection using the narrative in adverse event reports.[66] These models can be more precise than rule-based systems, particularly when working with large and complex datasets. The performance of machine learning models can be improved over time as they gain knowledge from fresh instances and adapt to changing data.[18, 55] Machine learning models are trained on extensive datasets of adverse event reports to identify patterns and connections between various factors, including drug names, patient demographics, and symptoms.[67] Once trained, these models can be used to automatically identify and categorize duplicates based on a variety of factors, including the similarity of medication names, the occurrence of specific medical conditions, and the timing and severity of symptoms.[68, 69] Machine learning models can be very accurate and efficient in finding complex duplicates; however, their creation and maintenance demands a significant quantity of high-quality training data and specialized knowledge.[70, 71]

Probabilistic record linkage: This method identifies and remove duplicate reports from large and complex datasets. An example is the hit-miss model which uses computational methods to analyze extensive healthcare

For peer review only - http://bmjopen.bmj.com/site/about/guidelines.xhtml

databases. It distinguishes "hits" (adverse events) from "misses" (non-events) by identifying patterns and signals.[3, 48] The method relies on statistical techniques to estimate the probability that two reports refer to the same event or individual, considering various data fields, such as patient name, age, and drug name. Probabilistic record linkage is particularly useful when dealing with datasets that are incomplete, inconsistent, or have missing data fields because they can still produce reliable results. However, this method can be computationally intensive and requires substantial computational resources, making it less practical for smaller datasets or less well-resourced pharmacovigilance systems.[4]
Manual review: This method is used to confirm reports that have been flagged as potential duplicates. Manual review is used in instances where automated methods are inconclusive.[6, 48]

Confirmation of duplicates: Manual confirmation is always necessary following the detection of potential duplicates. Well documented reports, with elaborate case narratives, make the confirmation process easier.[5, 64, 66] However, reports with limited information require that reporters are contacted for additional information, which necessitates the timely review of submitted reports.[6] When duplicates are detected, root cause analysis should be done and corrective action taken. Lastly, confirmed duplicates should be managed appropriately.[6]

Management of duplicates: This is the process of merging two or more reports to form one master report. Two approaches could be used: i) allocating an existing report as the master report, with the information from other reports being added unless the same or more precise information is already present in the master report, and ii) creating a master report by combining the information from several duplicate reports.[6, 21] The master report should always include the case reference numbers of all subordinate duplicate reports for easy traceability. Subordinate duplicates remain in the database for the purpose of audit trail but are not used for any other pharmacovigilance purpose. One challenge with duplicate management is when conflicting information is provided by different reporters. Clarification of conflicting information should be obtained wherever possible.[6]

Software programs: These are valuable tools for deduplication in pharmacovigilance databases. VigiMatch is a software program developed by the Uppsala Monitoring Centre to detect potential duplicates in the global database of adverse event reports, VigiBase.[26, 72] VigiMatch compares various data elements of adverse event reports and assigns a matching score to each pair of reports.[4] Although VigiMatch and VigiBase are separate programs, they are linked in the sense that VigiMatch is often used as a tool to identify and consolidate potential duplicate reports within VigiBase. VigiBase includes data cleaning and deduplication tools that use various criteria to identify potential duplicates, including patient information, drug information, and adverse event descriptions. VigiBase can merge duplicate reports identified by VigiMatch, ensuring that the database remains accurate and efficient.[48] VigiMatch is available for use by national pharmacovigilance centers and regulatory authorities worldwide. However, access to the software depends on various factors, such as the availability of funding, infrastructure, and technical expertise to effectively implement the software.[72]

Other software packages have been developed. The Medical Dictionary for Regulatory Activities (MedDRA) coding system is a standardized software for regulatory reporting that can identify duplicates and minimize data entry errors.[73] ArisGlobal's LifeSphere is a cloud-based pharmacovigilance software with a deduplication module to identify and consolidate duplicate reports.[74] Oracle's Argus Safety is a comprehensive safety management system with a deduplication feature to eliminate duplicate reports and improve data accuracy.[75] Ablebits Ultimate Suite of tools is software with a variety of *add-ins* and *plugins* for Microsoft Office Excel and Google Sheets. The software has a range of tools, including the "Remove Duplicates" feature that allows users to find and eliminate duplicate entries in a dataset. The primary purpose of Ablebits is to enhance the functionality of spreadsheet programs and make it easier for users to perform

For peer review only - http://bmjopen.bmj.com/site/about/guidelines.xhtml

various tasks. Ablebits offers a range of tools that can automate repetitive tasks, format data, merge cells, and compare data, etc. The software can also merge duplicate reports to create a single case record, thereby reducing manual review and data entry. The goal of Ablebits is to help users save time and improve their productivity while working with Excel or Google Sheets.[76] Open source software for data deduplication, e.g., DataCleaner Extension, can be used where Excel has limits.[77]

Advantages and limitations of software programs: The **advantages** of using software programs for deduplication include improved efficiency, accuracy, and consistency in identifying and managing duplicates. These programs can also handle large volumes of data and can be customized to meet specific regulatory requirements.[49] Using Excel with the Ablebits Ultimate Suite of tools could be more cost-effective for small datasets – the software is accessible and easy to use particularly if the user is familiar with Excel. However, Ablebits may not be as efficient or accurate as the dedicated software programs, particularly for large and complex pharmacovigilance databases.

However, there are also **limitations** to using software programs for deduplication. These programs may require extensive training and expertise to operate effectively, and might not detect all potential duplicates owing to variations in data entry or differences in data elements used for comparison. Additionally, the cost of implementing and maintaining these software programs may be a barrier for some regulatory authorities, especially in low- and middle-income countries. Artificial intelligence algorithms need to be trained with large quantities of high-quality data. The technical challenges of artificial intelligence-based pharmacovigilance, particularly in low- and middle-income countries, are the lack of high-quality databases, insufficient human resources, weak artificial intelligence technology, data sharing and privacy challenges, transparency of algorithms, interoperability across multiple platforms and insufficient support from governments.[28, 33, 71, 78-80]

Impact of duplication:

Inaccurate risk assessment: Duplicates in pharmacovigilance databases can lead to overestimation or underestimation of the frequency and severity of adverse events, introduce inconsistencies in data analysis and reporting, distort the understanding of the safety profile of medicines, and hinder signal detection and risk assessment. This compromises the integrity and reliability of databases as sources of accurate information, and potentially impacts important decisions related to drug labelling, risk management, and patient safety.[3, 4, 6, 7, 13, 21, 48, 64] As an example, Brinker et al.[41] observed an exaggerated safety signal of quinine-induced thrombocytopenia as a result of duplicate reports.

Resource wastage: Duplicates consume valuable database storage space, computing resources, and human effort. Maintaining and managing duplicate records uses up resources, which could be better utilized for other critical tasks.[4, 7, 77, 81] Having duplicate entries necessitates additional manual effort to identify and resolve them. Pharmacovigilance staff need to spend more time on data cleaning and deduplication processes, which diverts their attention from other essential activities.[3]

Delays in data analysis: Sorting through duplicate entries to identify and remove them can cause delays in data analysis and signal detection. Timely identification and response to safety concerns may be hindered due to the time-consuming nature of duplicate management.[3]

Regulatory compliance challenges: Duplicates can complicate regulatory reporting and compliance obligations. Thus, submitting accurate and non-duplicated data to regulatory authorities, may become more difficult and time-consuming, particularly for pharmaceutical companies.[6]

For peer review only - http://bmjopen.bmj.com/site/about/guidelines.xhtml

DISCUSSION

This scoping review sought to evaluate the extent, causes, preventive strategies, detection methods, management, and impact of duplication in pharmacovigilance databases worldwide. The extent of duplication in pharmacovigilance databases varied across different databases. The WHO global database, VigiBase, exhibited a 2.5% prevalence of duplication for reports submitted from 2000 to 2010, based on only half of the reports with sufficient information to conduct duplicate analysis.[4] In a global context, despite that this prevalence rate is relatively low, it represents about 750,000 duplicates among the 30+ million reports in VigiBase as of 2023.[82] Duplication rates in VigiBase were highest for reports from the published literature, e.g., peer-reviewed articles (11%), and fatal outcomes (5%);[4] and could be due to the heightened awareness and documentation of certain types of adverse events, particularly serious outcomes and those of special interest.[4, 39] These prevalence rates in VigiBase should, however, be interpreted with caution since half the reports were excluded from duplicate analysis due to missing data.[4] Duplication was more extreme (66% to 87%) for individual case safety reports from the published literature in the United States' FAERS database, and is almost entirely attributable to the replication of case reports due to the obligation to report individual case safety reports from the literature as enforced on pharmaceutical companies by Competent Authorities.[39] Thus, replication of case reports is an adjunct to duplication and is largely induced by regulatory reporting obligations, contractual agreements, and partnerships leading to the creation of case replicas.[7] The resolution of replication will require significant care and possibly redesigning pharmacovigilance data management systems at the global level.

Duplication rates in VigiBase varied across countries, being high for Czech Republic (15%), Austria (15%), Korea (9.2%) and Switzerland (4.7%), and much lower for United Kingdom (1.4%), Denmark (1%), Spain (0.7%) and Japan (8%), among others.[4] Thus, special attention to data quality is required when integrating pharmacovigilance datasets from different countries with varying risks of duplication. Also, regional variations call for standardized deduplication strategies to ensure consistency and data reliability across different settings.

Duplication in pharmacovigilance databases can arise from data entry errors, multiple reporters, mandatory reporting requirements, technical software glitches, and incomplete case information. This is compounded by the growing volume of pharmacovigilance reports worldwide, particularly due to the large number of adverse event reports following mass immunization with COVID-19 vaccines.[83] Thus, the possibility of duplicates cannot be ruled out, and it is crucial to develop robust and efficient systems to minimize them.[5] Understanding the causes of duplication is important to identify and institute effective measures to prevent them. Such measures could reduce the occurrence of duplication, promote coordinated approaches to deduplication, and enhance data quality and consistency. Standardized data entry and the assignment of unique identifiers to individual case safety reports is essential for consistent recording of adverse event data, making it easier to minimise and manage duplicates. The International Council for Harmonization of Technical Requirements for Pharmaceuticals for Human Use (ICH) has adopted the ICH E2B(R3) messaging standard for electronic transmission of individual case safety reports.[50] This standard stipulates the use of a worldwide unique case identification number (Section C.1.8.1) for each safety report which should not be changed during the transmission and subsequent re-transmission of safety reports.[50] This single field becomes the primary key for case identification, and is therefore a practical solution to effectively reduce and manage duplication. Regular automated and semi-automated data deduplication mechanisms, leveraging artificial intelligence, are among the array of measures at various stages of deployment across the United States, Europe, and Asia.[4, 6, 48, 51] However, there is notable scanty information from Africa. The effectiveness of deduplication methods will require continuous improvement, with time, to be in tandem with the submission of new reports to the pharmacovigilance databases.[5, 48, 64]

For peer review only - http://bmjopen.bmj.com/site/about/guidelines.xhtml

To enhance the accuracy of pharmacovigilance databases, newly submitted adverse event reports should be screened for duplicates. This proactive approach to data entry ensures superior data quality compared to relying solely on post-entry data cleaning processes.[6] Approaches to duplicate detection range from manual review to automated methods such as exact matching, rule-based matching, fuzzy matching, probabilistic record linkage and artificial intelligence. Probabilistic record linkage is effective for dealing with incomplete or inconsistent data but demands computational resources.[4] Artificial intelligence models based on machine learning provide more precise and efficient means of identifying duplicates, especially in large and complex datasets.[18, 55] Natural language processing techniques which use the narrative in adverse event reports have also been developed to augment duplicate detection using machine learning. These advances in natural language processing hold promise in enhancing the efficiency and precision of the deduplication of large and complex pharmacovigilance databases.[66] Automated detection systems are the recommended methods for large and complex databases.[5]·[31, 32] However, manual review is necessary for inconclusive potential duplicates from automated systems.[6, 29, 48] The duplicate detection algorithms of automated systems require routine fine-tuning to adapt them to changing data requirements, ensuring database accuracy.[18, 55]

Software programs for the deduplication of pharmacovigilance databases significantly enhance the quality of pharmacovigilance data and ultimately improve patient safety. Dedicated software programs for duplicate management are suitable for very large databases and might not be cost-effective for small databases, particularly in LMIC.[26, 73-75] Additional challenges for implementing dedicated deduplication software in LMIC include poor data quality, limited resources and infrastructure, inadequate training and capacity building, a lack of harmonization and standardization across regulatory systems, and limited access to data and information. These gaps can hinder the ability of regulatory authorities in LMIC to effectively monitor and assess drug safety and efficacy, and the impact of duplication on pharmacovigilance databases.[79] Alternative deduplication software for small datasets in LMIC include the Ablebits Ultimate Suite of tools with *add-ins* for Microsoft Office Excel. Using Ablebits in addition to manual review for small datasets could be cost-effective and possibly improves the efficiency and accuracy of deduplication.[76] However, open source software solutions, e.g., DataCleaner Extension, have superior capabilities to the Ablebits Excel *add-in.*[77]

Duplicates have consequences for pharmacovigilance databases such as the inaccurate risk assessment which could lead to overestimation or underestimation of adverse event frequencies. This could, in turn, distort the understanding of a drug's safety profile and hinder signal detection.[3-5] Resource wastage, increased workload for pharmacovigilance staff, delays in data analysis, and regulatory compliance challenges are additional consequences of duplication.[3, 4, 6, 7, 77, 81] These consequences underscore the importance of addressing duplication to promote the integrity and effectiveness of pharmacovigilance systems. We therefore make the following recommendations:

- We strongly advocate for the consistent use of worldwide unique identifiers (field C.1.8.1 within the ICH E2B(R3) guideline) as the primary key for case identification to minimise case duplication
- Scale up reporting methods that minimise incomplete case information, e.g., electronic systems that enforce the obligatory inclusion of key case details at the time of reporting
- Increase investment in pharmacovigilance infrastructure and capacity building
- Improve collaboration and data sharing among regulatory authorities and industry partners
- Put more emphasis on the harmonization and standardization of regulatory systems worldwide
- Develop and implement innovative technologies such as machine learning and natural language processing to improve the efficiency and accuracy of drug safety monitoring, particularly in LMIC.

For peer review only - http://bmjopen.bmj.com/site/about/guidelines.xhtml

- Low- and middle-income countries with small pharmacovigilance datasets ought to conduct pilot studies to explore the feasibility of alternative low-cost technologies for data deduplication (e.g., Ablebits Suite of tools in Microsoft Office Excel and the open source DataCleaner Extension).

This review has important limitations. First, the review did not evaluate the risk of bias or quality of included studies, in keeping with the design for scoping reviews, which limited the ability to document the methodological rigor or validity of included studies. Second, the majority of included papers were from Europe and North America which could have affected the generalisability of findings to other geographical regions. These limitations notwithstanding, however, the review provides a comprehensive overview of the cumulative body of literature on duplication in pharmacovigilance databases worldwide.

CONCLUSION

This scoping review provides a comprehensive overview of duplication in pharmacovigilance databases worldwide. It highlights the need for standardized practices, advanced detection methods, and collaborative efforts to minimize duplication and enhance the accuracy and reliability of drug safety data. Addressing duplication is crucial to supporting evidence-based decision-making in healthcare and drug regulation, ensuring patient safety. Software programs are commonly used for deduplication in pharmacovigilance databases and they significantly enhance the quality of pharmacovigilance data and ultimately improve patient safety. Overall, software programs offer advantages in terms of efficiency and accuracy, although there are limitations. For smaller datasets, Microsoft Office Excel with Ablebits Ultimate Suite may be cost-effective, and ought to be piloted, whereas larger and more complex datasets require dedicated software programs.

STATEMENTS AND DECLARATIONS

Funding

This study was funded by the Medical Research Council (MR/V03510X/1).

Conflicts of interest statement

Ronald Kiguba and Munir Pirmohamed obtained funding from the Medical Research Council; however, the funder had no role in drafting this manuscript. Ronald Kiguba, Gerald Isabirye, Julius Mayengo, Jonathan Owiny, Phil Tregunno, Kendal Harrison and Helen B. Ndagije declare that they have no conflicts of interest. Munir Pirmohamed has received partnership funding for the following: Medical Research Council Clinical Pharmacology Training Scheme (co-funded by Medical Research Council and Roche, UCB, Eli Lilly and Novartis). He also has unrestricted educational grant support for the UK Pharmacogenetics and Stratified Medicine Network from Bristol-Myers Squibb. He has developed an HLA genotyping panel with MC Diagnostics, but does not benefit financially from this. He is part of the IMI Consortium ARDAT (www.ardat.org). None of this funding MP received is related to the current paper.

Ethical approval

This review did not involve humans as study participants therefore ethical approval was not required.

Author's contributions

RK and GI conceptualized the study, collected the data, performed data curation, conducted formal analysis and data interpretation, and developed the first draft of the manuscript. JM, JO, PT, KH, MP and HBN contributed to the study design, analysis and interpretation of the data. All authors read and approved the final draft of the manuscript.

For peer review only - http://bmjopen.bmj.com/site/about/guidelines.xhtml

Acknowledgements

We are grateful to the National Drug Authority - Uganda, particularly the National Pharmacovigilance Centre, which supported the situation analysis of duplication in pharmacovigilance databases worldwide.

Data Sharing Statement

All data are included in the manuscript or uploaded as supplementary material.

For peer review only

References

1. World Health Organization. *The importance of pharmacovigilance*. Safety monitoring of medicinal products 2002 [cited 2023 28 August]; Available from: https://apps.who.int/iris/bitstream/handle/10665/42493/a75646.pdf.
2. World Health Organization. *Pharmacovigilance*. Regulation and Prequalification [cited 2023 13 September]; Available from: https://www.who.int/teams/regulation-prequalification/regulation-and-safety/pharmacovigilance.
3. Norén, G.N., et al., *Duplicate detection in adverse drug reaction surveillance.* Data Mining and Knowledge Discovery, 2007. **14**(3): p. 305-328.
4. Tregunno, P.M., et al., *Performance of probabilistic method to detect duplicate individual case safety reports.* Drug Saf, 2014. **37**(4): p. 249-58.
5. Norén, G.N., *The Power of the Case Narrative - Can it be Brought to Bear on Duplicate Detection?* Drug Saf, 2017. **40**(7): p. 543-546.
6. European Medicines Agency and Heads of Medicines Agencies. *Guideline on good pharmacovigilance practices (GVP) Module VI Addendum I – Duplicate management of suspected adverse reaction reports*. 2017 [cited 2023 07 August]; Available from: https://www.ema.europa.eu/en/documents/regulatory-procedural-guideline/guideline-good-pharmacovigilance-practices-gvp-module-vi-addendum-i-duplicate-management-suspected_en.pdf.
7. van Stekelenborg, J., et al., *Individual Case Safety Report Replication: An Analysis of Case Reporting Transmission Networks.* Drug Saf, 2023. **46**(1): p. 39-52.
8. European Medicines Agency. *Mapping of pharmacovigilance initiatives*. [cited 2023 13 September]; Available from: https://www.ema.europa.eu/en/documents/other/icmra-mapping-pharmacovigilance-initiatives_en.pdf.
9. Shankar, P.R., *Website review: The pharmacovigilance toolkit.* J Pharmacol Pharmacother, 2013. **4**(4): p. 307-8.
10. Council for International Organizations of Medical Sciences (CIOMS). *Practical Aspects of Signal Detection in Pharmacovigilance*. Report of CIOMS Working Group VIII, 2010 [cited 2023 21 September]; Available from: https://cioms.ch/wp-content/uploads/2018/03/WG8-Signal-Detection.pdf.
11. Council for International Organizations of Medical Sciences (CIOMS). *Making medicines safer for patients everywhere*. 2021 [cited 2023 21 September]; Available from: https://cioms.ch/wp-content/uploads/2021/11/CIOMS_SpecialNewsletter_UMC-9Nov2021.pdf.
12. United States (US) Food and Drug Administration (FDA) Adverse Event Reporting System (FAERS). *Questions and Answers on FDA's Adverse Event Reporting System (FAERS)*. 2018 [cited 2023 09 September]; Available from: https://www.fda.gov/drugs/surveillance/questions-and-answers-fdas-adverse-event-reporting-system-faers.
13. Hauben, M., et al., *'Extreme duplication' in the US FDA Adverse Events Reporting System database.* Drug Saf, 2007. **30**(6): p. 551-4.
14. European Medicines Agency. *EudraVigilance*. Human regulatory 2011 [cited 2023 29 August]; Available from: https://www.ema.europa.eu/en/human-regulatory/research-development/pharmacovigilance/eudravigilance.
15. Oliveira, J.L., et al., *The EU-ADR Web Platform: delivering advanced pharmacovigilance tools.* Pharmacoepidemiol Drug Saf, 2013. **22**(5): p. 459-67.
16. Nwokike, J. and K. Eghan. *Pharmacovigilance in Ghana: A Systems Analysis*. 2010 [cited 2023 29 August]; Available from: https://pdf.usaid.gov/pdf_docs/pnadu524.pdf.
17. Asiamah, M., et al., *Spontaneous reporting of adverse drug reaction among health professionals in Ghana.* Arch Public Health, 2022. **80**(1): p. 33.
18. Lavertu, A., et al., *A New Era in Pharmacovigilance: Toward Real-World Data and Digital Monitoring.* Clin Pharmacol Ther, 2021. **109**(5): p. 1197-1202.
19. Martin-Sanchez, F. and K. Verspoor, *Big data in medicine is driving big changes.* Yearb Med Inform, 2014. **9**(1): p. 14-20.

For peer review only - http://bmjopen.bmj.com/site/about/guidelines.xhtml

20. ECRI Institute. *Wrong-Record, Wrong-Data Errors with Health IT Systems*. 2015 [cited 2023 29 August]; Available from: https://www.ecri.org/Resources/In_the_News/PSONavigator_Data_Errors_in_Health_IT_Systems.pdf.
21. Ramya. *Duplicate search in pharmacovigilance*. 2019 [cited 2023 24 August]; Available from: https://allaboutpharmacovigilance.org/15-duplicate-search-in-pharmacovigilance/.
22. Pozsgai, K., et al., *Analysis of pharmacovigilance databases for spontaneous reports of adverse drug reactions related to substandard and falsified medical products: A descriptive study.* 2022. **13**.
23. Harpaz, R., H.S. Chase, and C. Friedman, *Mining multi-item drug adverse effect associations in spontaneous reporting systems.* BMC Bioinformatics, 2010. **11**(9): p. S7.
24. Uppsala Monitoring Centre. *Text is key for eliminating duplicate reports*. 2017 [cited 2023 29 August]; Available from: https://www.uppsalareports.org/articles/text-is-key-for-eliminating-duplicate-reports/.
25. Just, B.H., et al., *Why Patient Matching Is a Challenge: Research on Master Patient Index (MPI) Data Discrepancies in Key Identifying Fields.* Perspect Health Inf Manag, 2016. **13**(Spring): p. 1e.
26. Uppsala Monitoring Centre (UMC). *Sharing pharmacovigilance data in the WHO Programme for International Drug Monitoring* Reporting fact sheet [cited 2023 13 September]; Available from: https://who-umc.org/media/3236/reporting-fact-sheet.pdf.
27. Amedome SN and Dadson BA, *Pharmacovigilance Practices: Knowledge and Attitudes among the Healthcare Professionals at the Volta Regional Hospital of Ghana.* Journal of Pharmacovigilance, 2017. **5**(3).
28. Ghosh, R., et al., *Automation Opportunities in Pharmacovigilance: An Industry Survey.* Pharmaceut Med, 2020. **34**(1): p. 7-18.
29. Muñoz, M.A., et al., *Towards Automating Adverse Event Review: A Prediction Model for Case Report Utility.* Drug Saf, 2020. **43**(4): p. 329-338.
30. Gandomi, A. and M. Haider, *Beyond the hype: Big data concepts, methods, and analytics.* International Journal of Information Management, 2015. **35**(2): p. 137-144.
31. Lewis, D.J. and J.F. McCallum, *Utilizing Advanced Technologies to Augment Pharmacovigilance Systems: Challenges and Opportunities.* Ther Innov Regul Sci, 2020. **54**(4): p. 888-899.
32. Luo, Y., et al., *Natural Language Processing for EHR-Based Pharmacovigilance: A Structured Review.* Drug Saf, 2017. **40**(11): p. 1075-1089.
33. Hauben, M., *Artificial Intelligence and Data Mining for the Pharmacovigilance of Drug-Drug Interactions.* Clinical Therapeutics, 2023. **45**(2): p. 117-133.
34. Gosselt, H.R., et al., *Development of a multivariate prediction model to identify individual case safety reports which require clinical review.* Pharmacoepidemiol Drug Saf, 2022. **31**(12): p. 1300-1307.
35. Peters, M.D., et al., *Guidance for conducting systematic scoping reviews.* Int J Evid Based Healthc, 2015. **13**(3): p. 141-6.
36. Tricco, A.C., et al., *PRISMA Extension for Scoping Reviews (PRISMA-ScR): Checklist and Explanation.* Ann Intern Med, 2018. **169**(7): p. 467-473.
37. Clark, J.M., et al., *Improving the translation of search strategies using the Polyglot Search Translator: a randomized controlled trial.* J Med Libr Assoc, 2020. **108**(2): p. 195-207.
38. Canadian Agency for Drugs and Technologies in Health. *Grey matters: a practical tool for searching health-related grey literature.* 2023 [cited 2023 22 September]; Available from: https://greymatters.cadth.ca/.
39. Hung, E., et al., *More extreme duplication in FDA Adverse Event Reporting System detected by literature reference normalization and fuzzy string matching.* Pharmacoepidemiol Drug Saf, 2023. **32**(3): p. 387-391.
40. De Las Cuevas, C., et al., *Clozapine-associated myocarditis in the World Health Organization's pharmacovigilance database: Focus on reports from various countries.* Rev Psiquiatr Salud Ment (Engl Ed), 2022. **15**(4): p. 238-250.
41. Brinker, A.D. and J. Beitz, *Spontaneous reports of thrombocytopenia in association with quinine: clinical attributes and timing related to regulatory action.* Am J Hematol, 2002. **70**(4): p. 313-7.
42. European Medicines Agency. *Guideline on good pharmacovigilance practices (GVP) Module IX Addendum I – Methodological aspects of signal detection from spontaneous reports of suspected adverse reactions*. Module IX Addendum I – Methodological aspects of signal detection from spontaneous reports of suspected adverse reactions 2017 [cited 2023 07 August]; Available from: https://www.ema.europa.eu/en/documents/scientific-

For peer review only - http://bmjopen.bmj.com/site/about/guidelines.xhtml

guideline/guideline-good-pharmacovigilance-practices-gvp-module-ix-addendum-i-methodological-aspects-signal_en.pdf.
43. Banovac, M., et al., *Patient Reporting in the EU: Analysis of EudraVigilance Data.* Drug Saf, 2017. **40**(7): p. 629-645.
44. Candore, G., et al., *The Impact of Mandatory Reporting of Non-Serious Safety Reports to EudraVigilance on the Detection of Adverse Reactions.* Drug Saf, 2022. **45**(1): p. 83-95.
45. Lasys, T., et al., *Unintended impact of pharmacovigilance regulatory interventions: A systematic review.* Br J Clin Pharmacol, 2023.
46. Kahn, M.G., et al., *A Harmonized Data Quality Assessment Terminology and Framework for the Secondary Use of Electronic Health Record Data.* EGEMS (Wash DC), 2016. **4**(1): p. 1244.
47. US Food & Drug Administration. *Questions and Answers on FDA's Adverse Event Reporting System (FAERS).* 2018 [cited 2023 29 Augsut]; Available from: https://www.fda.gov/drugs/surveillance/questions-and-answers-fdas-adverse-event-reporting-system-faers.
48. Agency for Medicinal Products and Medical Devices of Croatia (HALMED). *Strengthening Collaboration for Operating Pharmacovigilance in Europe (SCOPE).* Duplicate Detection: Best Practice Guide [cited 2023 24 August]; Available from: https://www.ema.europa.eu/en/documents/other/scope-training-duplicate-detection-best-practice-guide_en.pdf
49. Richesson, R.L., et al., *An automated standardized system for managing adverse events in clinical research networks.* Drug Saf, 2008. **31**(10): p. 807-22.
50. European Medicines Agency. *ICH guideline E2B (R3) on electronic transmission of individual case safety reports (ICSRs) - data elements and message specification - implementation guide.* 2013 [cited 2024 11 March]; Available from: https://www.ema.europa.eu/en/documents/scientific-guideline/international-conference-harmonisation-technical-requirements-registration-pharmaceuticals-human-use-guideline-e2b-r3-electronic-transmission-individual-case-safety-reports-icsrs-data-elements_en.pdf.
51. Mockute, R., et al., *Artificial Intelligence Within Pharmacovigilance: A Means to Identify Cognitive Services and the Framework for Their Validation.* Pharmaceut Med, 2019. **33**(2): p. 109-120.
52. Bhangale, R., S. Vaity, and N. Kulkarni, *A day in the life of a pharmacovigilance case processor.* Perspect Clin Res, 2017. **8**(4): p. 192-195.
53. Paygude, P. and P.J.I.J.M.E.R. Devale, *Automated data validation testing tool for data migration quality assurance.* 2013. **3**(1): p. 599-603.
54. Postigo, R., et al., *EudraVigilance Medicines Safety Database: Publicly Accessible Data for Research and Public Health Protection.* Drug Saf, 2018. **41**(7): p. 665-675.
55. Wong, J., et al., *Applying Machine Learning in Distributed Data Networks for Pharmacoepidemiologic and Pharmacovigilance Studies: Opportunities, Challenges, and Considerations.* Drug Saf, 2022. **45**(5): p. 493-510.
56. Lindquist, M., *Data quality management in pharmacovigilance.* Drug Saf, 2004. **27**(12): p. 857-70.
57. Wadhwa, D., et al., *Automation in signal management in pharmacovigilance-an insight.* Brief Bioinform, 2021. **22**(4).
58. Hakak, S., et al., *A new split based searching for exact pattern matching for natural texts.* PLoS One, 2018. **13**(7): p. e0200912.
59. Christen, P., *Data matching: concepts and techniques for record linkage, entity resolution, and duplicate detection*. 2012, Springer: Data-centric systems and applications.
60. Liu H, Gegov A, and Stahl F. *Categorization and Construction of Rule Based Systems* 2014 [cited 2023 29 August]; Available from: https://researchportal.port.ac.uk/files/1938654/EANN2014_038.pdf.
61. Wong, C.K., et al., *Standardisation of the FAERS database: a systematic approach to manually recoding drug name variants.* Pharmacoepidemiology and drug safety, 2015. **24**(7): p. 731-737.
62. Grigoriev, I., et al., *AERS spider: an online interactive tool to mine statistical associations in Adverse Event Reporting System.* Pharmacoepidemiol Drug Saf, 2014. **23**(8): p. 795-801.
63. Yang, J.D., *F_MP: A fuzzy match framework for rule-based programming.* Data & Knowledge Engineering, 1997. **24**(2): p. 183-203.

For peer review only - http://bmjopen.bmj.com/site/about/guidelines.xhtml

64. Kreimeyer, K., et al., *Increased Confidence in Deduplication of Drug Safety Reports with Natural Language Processing of Narratives at the US Food and Drug Administration.* 2022. **2**.
65. Schmider, J., et al., *Innovation in Pharmacovigilance: Use of Artificial Intelligence in Adverse Event Case Processing.* Clin Pharmacol Ther, 2019. **105**(4): p. 954-961.
66. Kreimeyer, K., et al., *Using Probabilistic Record Linkage of Structured and Unstructured Data to Identify Duplicate Cases in Spontaneous Adverse Event Reporting Systems.* Drug Saf, 2017. **40**(7): p. 571-582.
67. Harpaz, R., et al., *Toward multimodal signal detection of adverse drug reactions.* J Biomed Inform, 2017. **76**: p. 41-49.
68. Kreimeyer, K., et al., *Feature engineering and machine learning for causality assessment in pharmacovigilance: Lessons learned from application to the FDA Adverse Event Reporting System.* Computers in Biology and Medicine, 2021. **135**: p. 104517.
69. Fusaroli, M., et al., *Development of a Network-Based Signal Detection Tool: The COVID-19 Adversome in the FDA Adverse Event Reporting System.* Front Pharmacol, 2021. **12**: p. 740707.
70. Kim, H.R., et al., *Analyzing adverse drug reaction using statistical and machine learning methods: A systematic review.* Medicine (Baltimore), 2022. **101**(25): p. e29387.
71. Kompa, B., et al., *Artificial Intelligence Based on Machine Learning in Pharmacovigilance: A Scoping Review.* Drug Saf, 2022. **45**(5): p. 477-491.
72. Council for International Organizations of Medical Sciences (CIOMS). *Making medicines safer for patients everywhere*. 2021 [cited 2023 29 August]; Available from: https://cioms.ch/wp-content/uploads/2021/11/CIOMS_SpecialNewsletter_UMC-9Nov2021.pdf.
73. MedDRA. *History*. [cited 2023 03 October]; Available from: https://www.meddra.org/about-meddra/history.
74. ArisGlobal. *ArisGlobal Announces LifeSphere® MultiVigilance 10, Industry's First End-to-End Safety System With Production-Ready Automation*. 2019 [cited 2023 09 September]; Available from: https://www.arisglobal.com/press-release/lifesphere-multivigilance-10-first-safety-system-production-ready-automation/.
75. Oracle. *Trusted safety case management*. 2021 [cited 2023 03 October]; Available from: https://www.oracle.com/a/ocom/docs/argus-trusted-safety-case-management.pdf.
76. Ablebits. *Ablebits Ultimate Suite*. 2023 [cited 2023 03 October]; Available from: https://www.ablebits.com/.
77. Azeroual, O., et al., *A Record Linkage-Based Data Deduplication Framework with DataCleaner Extension.* 2022. **6**(4): p. 27.
78. Liang, L., et al., *Artificial Intelligence-Based Pharmacovigilance in the Setting of Limited Resources.* Drug Safety, 2022. **45**(5): p. 511-519.
79. Kiguba, R., S. Olsson, and C. Waitt, *Pharmacovigilance in low- and middle-income countries: A review with particular focus on Africa.* Br J Clin Pharmacol, 2023. **89**(2): p. 491-509.
80. He, J., et al., *The practical implementation of artificial intelligence technologies in medicine.* Nature Medicine, 2019. **25**(1): p. 30-36.
81. Rathbone, J., et al., *Better duplicate detection for systematic reviewers: evaluation of Systematic Review Assistant-Deduplication Module.* Syst Rev, 2015. **4**(1): p. 6.
82. Uppsala Monitoring Centre. *What is VigiBase?* 2023 [cited 2023 16 October]; Available from: https://who-umc.org/vigibase/.
83. van der Boor, S.C., et al., *Spontaneously reported adverse events following COVID-19 basic and booster immunizations in the Netherlands.* Vaccine, 2023. **41**(29): p. 4319-4326.

For peer review only

For peer review only - http://bmjopen.bmj.com/site/about/guidelines.xhtml

Table 1: Summary of screening process

Screening Process	Number of Papers
Initial Screening	22745
Full Text Review	156
Eligibility Criteria	
- Met Criteria?	
- Yes	58
- No	98
Specific Categories*	
- Extent of Duplicate	5
- Prevention Strategies	15
- Causes of Duplicates	13
- Methods of Duplicate Detection	32
- Management Strategies	24
- Implications of Duplicates	14

* The papers overlapped, discussing more than one field

Figure 1: PRISMA flow diagram used to select papers for the scoping review

For peer review only

For peer review only - http://bmjopen.bmj.com/site/about/guidelines.xhtml

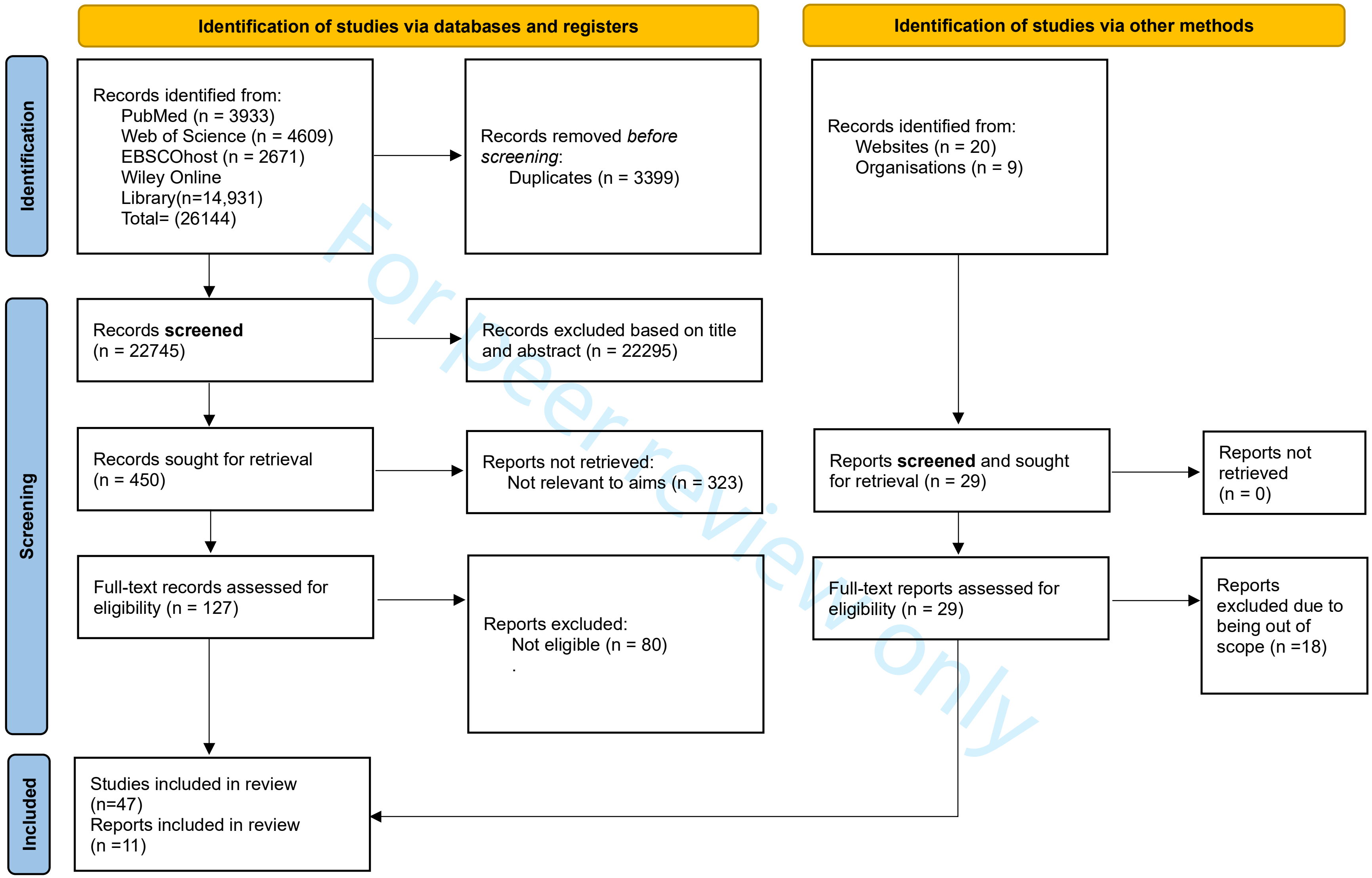

Figure 1: *PRISMA flow diagram used to select papers for the scoping review*

For peer review only

Appendices

Appendix I: Search strategy in PubMed

((((prevalence[tw] OR incidence[tw] OR extent[tw] **OR** epidemiology[tw] OR frequency[tw]) AND (duplication[tw] OR "Data Duplication"[tw] OR "Duplicate Report*"[tw] OR "Redundant Data"[tw] OR "duplicate record*"[tw] OR replication[tw])) AND ("pharmacovigilance database*"[tw] OR database*[tw]))

OR

(((prevention[tw] OR "prevention strateg*"[tw] OR cause*[tw] OR detection[tw] OR management[tw] OR "management strateg*"[tw] OR deduplication[tw]) AND (duplication[tw] OR "Data Duplication"[tw] OR "Duplicate Report*"[tw] OR "Redundant Data"[tw] OR "duplicate record*"[tw] OR replication[tw])) AND ("pharmacovigilance database*"[tw] OR database*[tw])))

OR

(((impact* OR consequence* OR effect*) AND (duplication[tw] OR "Data Duplication"[tw] OR "Duplicate Report*"[tw] OR "Redundant Data"[tw] OR "duplicate record*"[tw] OR replication[tw])) AND ("pharmacovigilance database*"[tw] OR database*[tw]))

Using the Polyglot Search Translator, we adapted the PubMed search strategy across the other peer-reviewed research databases, namely, Web of Science, Wiley Online Library and EBSCOhost.

Appendix II: Sources of grey literature search

Search terms use: Duplicate management in pharmacovigilance databases.

**Source**	**Organizations**	**Website Links**
London	European Medicines Agency	https://www.ema.europa.eu/en/documents/regulatory-procedural-guideline/guideline-good-pharmacovigilance-practices-gvp-module-vi-addendum-i-duplicate-management-suspected_en.pdf
India	https://allaboutpharmacovigilance.org/	https://allaboutpharmacovigilance.org/15-duplicate-search-in-pharmacovigilance/
Sweden	Uppsala	https://www.uppsalareports.org/articles/text-is-key-for-eliminating-duplicate-reports/
London	Medical literature monitoring	https://www.ema.europa.eu/en/human-regulatory/post-authorisation/pharmacovigilance/medical-literature-monitoring
London	**Clarivate PLC**	https://go.dialog.com/MLM_Duplication_Whitepaper/

For peer review only - http://bmjopen.bmj.com/site/about/guidelines.xhtml

London	PubHive	https://pubhive.com/pulse-literature-1/f/pubhive-automates-duplicate-detection-for-ema%E2%80%99s-mlm-service
Sweden	uppsala	https://who-umc.org/media/3236/reporting-fact-sheet.pdf
Maryland USA	FDA	https://www.accessdata.fda.gov/scripts/cderworld/index.cfm?action=drugsafety:main&unit=1&lesson=1&topic=17
New Jersey USA	flexdatabases	https://flexdatabases.com/6-faq-pv/
New York	citeline	https://pink.citeline.com/-/media/supporting-documents/pink-sheet/2020/02/p0220ema_9.pdf
London	GOV.UK	https://assets.publishing.service.gov.uk/government/uploads/system/uploads/attachment_data/file/949102/Exceptions_and_modifications_to_the_EU_guidance_on_good_pharmacovigilance_practices_that_apply_to_UK_MAHs_v2.pdf
San Francisco	WordPress.com	https://auditandcompliance.wordpress.com/2018/12/23/basic-steps-in-pharmacovigilance-case-processing/
Pakistan	Drug Regulatory Authority of Pakistan	https://www.dra.gov.pk/wp-content/uploads/2022/04/Good-Pharmacovigilance-Guidelines-for-Registration-Holders.pdf
Sweden	Uppsala	https://www.diva-portal.org/smash/get/diva2:1670567/FULLTEXT01.pdf
Lithuania	Biomapas	https://www.biomapas.com/selecting-a-pharmacovigilance-drug-safety-database/
Erupe	ENCePP	https://www.encepp.eu/standards_and_guidances/methodologicalGuide8_4.shtml

For peer review only - http://bmjopen.bmj.com/site/about/guidelines.xhtml

Brussels	AFMPS	https://www.afmps.be/sites/default/files/content/faqs_ev_changes_22-11-2017_afmps-fagg.pdf
Amsterdam	European medicines agency	https://www.adrreports.eu/en/data_quality.html#:~:text=a%20report%20is%20submitted%20by,to%20the%20report%20being%20erroneous.
Amsterdam	European medicines agency	https://www.ema.europa.eu/en/documents/other/scope-training-duplicate-detection-best-practice-guide_en.pdf
Maryland USA	FDA	https://www.fda.gov/drugs/surveillance/questions-and-answers-fdas-adverse-event-reporting-system-faers
New York	IEEE	https://ieeexplore.ieee.org/abstract/document/6732591/similar#similar

For peer review only

For peer review only - http://bmjopen.bmj.com/site/about/guidelines.xhtml

Appendix III: Data extraction instrument

Auth or(s)	Jou rnal	Ti tle	Speci fic Objec tives	Cou ntry	Stu dy des ign	Year of public ation	Ext ent	Preve ntion	Ca use s	Dete ction	Manag ement	Implic ations	Key find ings

Appendix IV: Regions and countries

Region	Number of Publications	Countries
Asia Pacific	6 publications	China<br>India<br>Malaysia<br>South Korea
Europe	27 publications	Sweden<br>Georgia<br>Switzerland<br>Netherlands<br>England<br>Italy<br>Hungary<br>Switzerland<br>Croatia<br>Germany<br>Poland<br>Portugal
Australia	3 publications	Australia
North America	22 publications	USA

For peer review only

For peer review only - http://bmjopen.bmj.com/site/about/guidelines.xhtml

Appendix V: List of 99 articles excluded on full-text review

No.	PMID	Title	Citation	First Author	Journal/Book	Publication Year
1	22360774	Pharmacovigilance	Br J Clin Pharmacol. 2012 Jun;73(6):979-82. doi: 10.1111/j.1365-2125.2012.04249.x.	Edwards IR	Br J Clin Pharmacol	2012
2	25123728	"Big data" and the electronic health record	Yearb Med Inform. 2014 Aug 15;9(1):97-104. doi: 10.15265/IY-2014-0003.	Ross MK	Yearb Med Inform	2014
3	33634545	Suitability of databases in the Asia-Pacific for collaborative monitoring of vaccine safety	Pharmacoepidemiol Drug Saf. 2021 Jul;30(7):843-857. doi: 10.1002/pds.5214. Epub 2021 Mar 23.	Duszynski KM	Pharmacoepidemiol Drug Saf	2021
4	35179704	High-Dimensional Propensity Score-Adjusted Case-Crossover for Discovering Adverse Drug Reactions from Computerized Administrative Healthcare Databases	Drug Saf. 2022 Mar;45(3):275-285. doi: 10.1007/s40264-022-01148-5. Epub 2022 Feb 18.	Volatier E	Drug Saf	2022
5	29474173	Possibility of Database Research as a Means of Pharmacovigilance in Japan Based on a Comparison with Sertraline Postmarketing Surveillance	Value Health Reg Issues. 2018 May;15:1-5. doi: 10.1016/j.vhri.2017.05.002. Epub 2017 Jun 20.	Hirano Y	Value Health Reg Issues	2018
6	32243569	Different Strategies to Execute Multi-Database Studies for Medicines Surveillance in Real-World Setting: A Reflection on the European Model	Clin Pharmacol Ther. 2020 Aug;108(2):228-235. doi: 10.1002/cpt.1833. Epub 2020 May 5.	Gini R	Clin Pharmacol Ther	2020
7	31464008	Establishment of the MID-NET(®) medical information database network as a reliable and valuable database for drug safety assessments in Japan	Pharmacoepidemiol Drug Saf. 2019 Oct;28(10):1395-1404. doi: 10.1002/pds.4879. Epub 2019 Aug 29.	Yamaguchi M	Pharmacoepidemiol Drug Saf	2019
8	31933254	Artificial Intelligence Within Pharmacovigilance: A Means to Identify Cognitive Services and the Framework for Their Validation	Pharmaceut Med. 2019 Apr;33(2):109-120. doi: 10.1007/s40290-019-00269-0.	Mockute R	Pharmaceut Med	2019
9	35239736	Evaluation of pharmacovigilance systems for reporting medication errors in Africa and the role of patients using a mixed-methods approach	PLoS One. 2022 Mar 3;17(3):e0264699. doi: 10.1371/journal.pone.0264699. eCollection 2022.	Sabblah GT	PLoS One	2022
10	30445979	Organizational capacities of national pharmacovigilance centres in Africa: assessment of resource elements associated with successful and unsuccessful	Global Health. 2018 Nov 16;14(1):109. doi: 10.1186/s12992-018-0431-0.	Ampadu HH	Global Health	2018
11	36097239	Artificial Intelligent Context-Aware Machine-Learning Tool to Detect Adverse Drug Events from Social Media Platforms	J Med Toxicol. 2022 Oct;18(4):311-320. doi: 10.1007/s13181-022-00906-2. Epub 2022 Sep 12.	Roosan D	J Med Toxicol	2022
12	29714535	An Alternative to Disproportionality: A Frequency-Based Method for Pharmacovigilance Data Mining	Ther Innov Regul Sci. 2018 May;52(3):294-299. doi: 10.1177/2168479017728986. Epub 2017 Sep 8.	Jokinen JD	Ther Innov Regul Sci	2018
13	30185579	Electronic healthcare databases in Europe: descriptive analysis of characteristics and potential for use in medicines regulation	BMJ Open. 2018 Sep 5;8(9):e023090. doi: 10.1136/bmjopen-2018-023090.	Pacurariu A	BMJ Open	2018
14	32026758	Combining big data search analytics and the FDA Adverse Event Reporting System database to detect a potential safety signal of mirtazapine abuse	Health Informatics J. 2020 Sep;26(3):2265-2279. doi: 10.1177/1460458219901232. Epub 2020 Feb 6.	Spachos D	Health Informatics J	2020
15	23512870	Drug safety data mining with a tree-based scan statistic	Pharmacoepidemiol Drug Saf. 2013 May;22(5):517-23. doi: 10.1002/pds.3423. Epub 2013 Mar 20.	Kulldorff M	Pharmacoepidemiol Drug Saf	2013
16	25151493	Text mining for adverse drug events: the promise, challenges, and state of the art	Drug Saf. 2014 Oct;37(10):777-90. doi: 10.1007/s40264-014-0218-z.	Harpaz R	Drug Saf	2014
17	26606038	Predictive modeling of structured electronic health records for adverse drug event detection	BMC Med Inform Decis Mak. 2015;15 Suppl 4(Suppl 4):S1. doi: 10.1186/1472-6947-15-S4-S1. Epub 2015 Nov 25.	Zhao J	BMC Med Inform Decis Mak	2015
18	24599513	Reducing the noise in signal detection of adverse drug reactions by standardizing the background: a pilot study on analyses of proportional reporting ratios-by-therapeutic area	Eur J Clin Pharmacol. 2014 May;70(5):627-35. doi: 10.1007/s00228-014-1658-1. Epub 2014 Mar 7.	Grundmark B	Eur J Clin Pharmacol	2014
19	36380085	A real-world pharmacovigilance study of FDA Adverse Event Reporting System (FAERS) events for osimertinib	Sci Rep. 2022 Nov 15;12(1):19555. doi: 10.1038/s41598-022-23834-1.	Yin Y	Sci Rep	2022
20	31677951	ADVANCE: Towards near real-time monitoring of vaccination coverage, benefits and risks using European electronic health record databases	Vaccine. 2020 Dec 22;38 Suppl 2:B76-B83. doi: 10.1016/j.vaccine.2019.08.012. Epub 2019 Oct 31.	Bollaerts K	Vaccine	2020
21	21219405	Using primary care prescribing databases for pharmacovigilance	Br J Clin Pharmacol. 2011 Feb;71(2):244-9. doi: 10.1111/j.1365-2125.2010.03816.x.	Mohamed IN	Br J Clin Pharmacol	2011
22	35691583	Development and external validation of a 1- and 5-year fracture prediction tool based on electronic medical records data: The EPIC risk algorithm	Bone. 2022 Sep;162:116469. doi: 10.1016/j.bone.2022.116469. Epub 2022 Jun 9.	Tebé C	Bone	2022
23	31198563	Pharmacovigilance systems in resource-limited settings: an evaluative case study of Sierra Leone	J Pharm Policy Pract. 2019 Jun 11;12:13. doi: 10.1186/s40545-019-0173-2. eCollection 2019.	Abiri OT	J Pharm Policy Pract	2019
24	25123721	Big data - smart health strategies. Findings from the yearbook 2014 special theme	Yearb Med Inform. 2014 Aug 15;9(1):48-51. doi: 10.15265/IY-2014-0031.	Koutkias V	Yearb Med Inform	2014

For peer review only

No.	PMID	Title	Citation	First Author	Journal/Book	Publication Year
25	36147337	Analysis of pharmacovigilance databases for spontaneous reports of adverse drug reactions related to substandard and falsified medical products: A descriptive study	Front Pharmacol. 2022 Sep 6;13:964399. doi: 10.3389/fphar.2022.964399. eCollection 2022.	Pozsgai K	Front Pharmacol	2022
26	23331229	Automated method for detecting increases in frequency of spontaneous adverse event reports over time	J Biopharm Stat. 2013;23(1):161-77. doi: 10.1080/10543406.2013.736809.	DuMouchel W	J Biopharm Stat	2013
27	26819727	Pharmacoepidemiology in Japan: medical databases and research achievements	J Pharm Health Care Sci. 2015 May 1;1:16. doi: 10.1186/s40780-015-0016-5. eCollection 2015.	Tanaka S	J Pharm Health Care Sci	2015
28	34694230	Adverse Drug Event Prediction Using Noisy Literature-Derived Knowledge Graphs: Algorithm Development and Validation	JMIR Med Inform. 2021 Oct 25;9(10):e32730. doi: 10.2196/32730.	Dasgupta S	JMIR Med Inform	2021
29	36438789	Data mining and safety analysis of BTK inhibitors: A pharmacovigilance investigation based on the FAERS database	Front Pharmacol. 2022 Nov 11;13:995522. doi: 10.3389/fphar.2022.995522. eCollection 2022.	Wan Q	Front Pharmacol	2022
30	28595649	Data-driven prediction of adverse drug reactions induced by drug-drug interactions	BMC Pharmacol Toxicol. 2017 Jun 8;18(1):44. doi: 10.1186/s40360-017-0153-6.	Liu R	BMC Pharmacol Toxicol	2017
31	31004158	Prevalence and incidence of narcolepsy in a US health care claims database, 2008-2010	Sleep. 2019 Jul 8;42(7):zsz091. doi: 10.1093/sleep/zsz091.	Scheer D	Sleep	2019
32	31507029	Data variability across Canadian administrative health databases: Differences in content, coding, and completeness	Pharmacoepidemiol Drug Saf. 2020 Jan;29 Suppl 1:68-77. doi: 10.1002/pds.4889. Epub 2019 Sep 10.	Doyle CM	Pharmacoepidemiol Drug Saf	2020
33	32564242	Risk Factor Considerations in Statistical Signal Detection: Using Subgroup Disproportionality to Uncover Risk Groups for Adverse Drug Reactions in VigiBase	Drug Saf. 2020 Oct;43(10):999-1009. doi: 10.1007/s40264-020-00957-w.	Sandberg L	Drug Saf	2020
34	23070598	Comparison of two drug safety signals in a pharmacovigilance data mining framework	Stat Methods Med Res. 2016 Apr;25(2):615-29. doi: 10.1177/0962280212462295. Epub 2012 Oct 14.	Tubert-Bitter P	Stat Methods Med Res	2016
35	29446035	Sorting Through the Safety Data Haystack: Using Machine Learning to Identify Individual Case Safety Reports in Social-Digital Media	Drug Saf. 2018 Jun;41(6):579-590. doi: 10.1007/s40264-018-0641-7.	Comfort S	Drug Saf	2018
36	24787710	Paediatric pharmacovigilance: use of pharmacovigilance data mining algorithms for signal detection in a safety dataset of a paediatric clinical study conducted in seven African countries	PLoS One. 2014 May 1;9(5):e96388. doi: 10.1371/journal.pone.0096388. eCollection 2014.	Kajungu DK	PLoS One	2014
37	28646789	Data-mining for detecting signals of adverse drug reactions of fluoxetine using the Korea Adverse Event Reporting System (KAERS) database	Psychiatry Res. 2017 Oct;256:237-242. doi: 10.1016/j.psychres.2017.06.038. Epub 2017 Jun 13.	Kim S	Psychiatry Res	2017
38	21701609	Information technology in pharmacovigilance: Benefits, challenges, and future directions from industry perspectives	Drug Healthc Patient Saf. 2009;1:35-45. doi: 10.2147/dhps.s7180. Epub 2009 Oct 15.	Lu Z	Drug Healthc Patient Saf	2009
39	27868278	Differences in VigiBase® reporting of aminoglycoside and capreomycin-suspected ototoxicity during tuberculosis treatment	Pharmacoepidemiol Drug Saf. 2017 Jan;26(1):1-8. doi: 10.1002/pds.4125. Epub 2016 Nov 20.	Sagwa EL	Pharmacoepidemiol Drug Saf	2017
40	23921799	Triptans and serious adverse vascular events: data mining of the FDA Adverse Event Reporting System database	Cephalalgia. 2014 Jan;34(1):5-13. doi: 10.1177/0333102413499649. Epub 2013 Aug 6.	Roberto G	Cephalalgia	2014
41	31673326	Evaluation of quantitative signal detection in EudraVigilance for orphan drugs: possible risk of false negatives	Ther Adv Drug Saf. 2019 Oct 21;10:2042098619882819. doi: 10.1177/2042098619882819. eCollection 2019.	Sardella M	Ther Adv Drug Saf	2019
42	36050484	Detecting early safety signals of infliximab using machine learning algorithms in the Korea adverse event reporting system	Sci Rep. 2022 Sep 1;12(1):14869. doi: 10.1038/s41598-022-18522-z.	Lee JE	Sci Rep	2022
43	30324671	Significance of data mining in routine signal detection: Analysis based on the safety signals identified by the FDA	Pharmacoepidemiol Drug Saf. 2018 Dec;27(12):1402-1408. doi: 10.1002/pds.4672. Epub 2018 Oct 15.	Fukazawa C	Pharmacoepidemiol Drug Saf	2018
44	31156424	Computational Advances in Drug Safety: Systematic and Mapping Review of Knowledge Engineering Based Approaches	Front Pharmacol. 2019 May 17;10:415. doi: 10.3389/fphar.2019.00415. eCollection 2019.	Natsiavas P	Front Pharmacol	2019
45	28335691	Cases of drug-induced Torsade de Pointes: a review of Belgian cases in the EudraVigilance database	Acta Clin Belg. 2017 Dec;72(6):385-390. doi: 10.1080/17843286.2017.1300217. Epub 2017 Mar 24.	Vandael E	Acta Clin Belg	2017
46	16134080	Signal detection in pharmacovigilance: empirical evaluation of data mining tools	Pharmacoepidemiol Drug Saf. 2005 Sep;14(9):597-9. doi: 10.1002/pds.1128.	Chan KA	Pharmacoepidemiol Drug Saf	2005
47	35745593	Pharmacovigilance Signals of the Opioid Epidemic over 10 Years: Data Mining Methods in the Analysis of Pharmacovigilance Datasets Collecting Adverse Drug Reactions (ADRs) Reported to EudraVigilance (EV) and the FDA Adverse Event Reporting System (FAERS)	Pharmaceuticals (Basel). 2022 May 27;15(6):675. doi: 10.3390/ph15060675.	Chiappini S	Pharmaceuticals (Basel)	2022

No.	PMID	Title	Citation	First Author	Journal/Book	Publication Year
48	36050787	Challenges and opportunities for mining adverse drug reactions: perspectives from pharma, regulatory agencies, healthcare providers and consumers	Database (Oxford). 2022 Sep 2;2022:baac071. doi: 10.1093/database/baac071.	Gonzalez-Hernandez G	Database (Oxford)	2022
49	33518634	[Role and Applicability of Spontaneous Reporting Databases in Medical Big Data]	Yakugaku Zasshi. 2021;141(2):165-168. doi: 10.1248/yakushi.20-00196-1.	Sakai T	Yakugaku Zasshi	2021
50	32169289	Uses of pharmacovigilance databases: An overview	Therapie. 2020 Nov-Dec;75(6):591-598. doi: 10.1016/j.therap.2020.02.022. Epub 2020 Feb 26.	Bihan K	Therapie	2020
51	35904529	The Use of Artificial Intelligence in Pharmacovigilance: A Systematic Review of the Literature	Pharmaceut Med. 2022 Oct;36(5):295-306. doi: 10.1007/s40290-022-00441-z. Epub 2022 Jul 29.	Salas M	Pharmaceut Med	2022
52	31623850	Pharmacovigilance - The next chapter	Therapie. 2019 Dec;74(6):557-567. doi: 10.1016/j.therap.2019.09.004. Epub 2019 Oct 1.	Moore N	Therapie	2019
53	27224991	[Pharmacovigilance in Germany : It is about time]	Internist (Berl). 2016 Jun;57(6):616-23. doi: 10.1007/s00108-016-0068-0.	Douros A	Internist (Berl)	2016
54	33598764	Construction and analysis of a database for medication errors in a pharmacovigilance centre-the Moroccan experience	Eur J Clin Pharmacol. 2021 Aug;77(8):1235-1246. doi: 10.1007/s00228-021-03109-w. Epub 2021 Feb 17.	Alj L	Eur J Clin Pharmacol	2021
55	35788724	Evolution of adverse drug reactions reporting systems: paper based to software based	Eur J Clin Pharmacol. 2022 Sep;78(9):1385-1390. doi: 10.1007/s00228-022-03358-3. Epub 2022 Jul 5.	Madhushika MT	Eur J Clin Pharmacol	2022
56	23640186	The past, present and perhaps future of pharmacovigilance: homage to Folke Sjoqvist	Eur J Clin Pharmacol. 2013 May;69 Suppl 1:33-41. doi: 10.1007/s00228-013-1486-8. Epub 2013 May 3.	Moore N	Eur J Clin Pharmacol	2013
57	35579806	Artificial Intelligence in Pharmacovigilance: An Introduction to Terms, Concepts, Applications, and Limitations	Drug Saf. 2022 May;45(5):407-418. doi: 10.1007/s40264-022-01156-5. Epub 2022 May 17.	Aronson JK	Drug Saf	2022
58	36251280	Development of a multivariate prediction model to identify individual case safety reports which require clinical review	Pharmacoepidemiol Drug Saf. 2022 Dec;31(12):1300-1307. doi: 10.1002/pds.5553. Epub 2022 Oct 21.	Gosselt HR	Pharmacoepidemiol Drug Saf	2022
59	26575203	Big Data and Adverse Drug Reaction Detection	Clin Pharmacol Ther. 2016 Mar;99(3):268-70. doi: 10.1002/cpt.302. Epub 2015 Dec 23.	Harpaz R	Clin Pharmacol Ther	2016
60	32176553	The potential role of big data in the detection of adverse drug reactions	Expert Rev Clin Pharmacol. 2020 Mar;13(3):201-204. doi: 10.1080/17512433.2020.1740086. Epub 2020 Mar 16.	Sultana J	Expert Rev Clin Pharmacol	2020
61	24698186	[Terappel: description of a computerised database and examples of studies]	Therapie. 2014 Jan-Feb;69(1):31-8. doi: 10.2515/therapie/2014013. Epub 2014 Apr 4.	Vial T	Therapie	2014
62	28840504	From Big Data to Smart Data for Pharmacovigilance: The Role of Healthcare Databases and Other Emerging Sources	Drug Saf. 2018 Feb;41(2):143-149. doi: 10.1007/s40264-017-0592-4.	Trifirò G	Drug Saf	2018
63	22315152	Electronic healthcare databases for active drug safety surveillance: is there enough leverage?	Pharmacoepidemiol Drug Saf. 2012 Jun;21(6):611-21. doi: 10.1002/pds.3197. Epub 2012 Feb 8.	Coloma PM	Pharmacoepidemiol Drug Saf	2012
64	19745234	The EU-ADR project: preliminary results and perspective	Stud Health Technol Inform. 2009;148:43-9.	Trifiro G	Stud Health Technol Inform	2009
65	26092163	[Benefits of large healthcare databases for drug risk research]	Bundesgesundheitsblatt Gesundheitsforschung Gesundheitsschutz. 2015 Aug;58(8):829-837. doi: 10.1007/s00103-015-2185-7.	Garbe E	Bundesgesundheitsblatt Gesund	2015
66	23670805	Pilot evaluation of an automated method to decrease false-positive signals induced by co-prescriptions in spontaneous reporting databases	Pharmacoepidemiol Drug Saf. 2014 Feb;23(2):186-94. doi: 10.1002/pds.3454. Epub 2013 May 14.	Avillach P	Pharmacoepidemiol Drug Saf	2014
67	23208789	The EU-ADR Web Platform: delivering advanced pharmacovigilance tools	Pharmacoepidemiol Drug Saf. 2013 May;22(5):459-67. doi: 10.1002/pds.3375. Epub 2012 Dec 4.	Oliveira JL	Pharmacoepidemiol Drug Saf	2013
68	31912439	Towards Automating Adverse Event Review: A Prediction Model for Case Report Utility	Drug Saf. 2020 Apr;43(4):329-338. doi: 10.1007/s40264-019-00897-0.	Muñoz MA	Drug Saf	2020
69	22873501	Implemented data mining and signal management systems on spontaneous reporting systems' databases and their availability to the scientific community - a systematic review	Curr Drug Saf. 2012 Apr;7(2):170-5. doi: 10.2174/157488612802715645.	de Almeida Vieira Lima LM	Curr Drug Saf	2012
70	22549283	Novel data-mining methodologies for adverse drug event discovery and analysis	Clin Pharmacol Ther. 2012 Jun;91(6):1010-21. doi: 10.1038/clpt.2012.50.	Harpaz R	Clin Pharmacol Ther	2012
71	18523760	Pharmacovigilance: methods, recent developments and future perspectives	Eur J Clin Pharmacol. 2008 Aug;64(8):743-52. doi: 10.1007/s00228-008-0475-9. Epub 2008 Jun 4.	Härmark L	Eur J Clin Pharmacol	2008
72	23072620	Global patterns of adverse drug reactions over a decade: analyses of spontaneous reports to VigiBase™	Drug Saf. 2012 Dec 1;35(12):1171-82. doi: 10.1007/BF03262002.	Aagaard L	Drug Saf	2012

For peer review only

No.	PMID	Title	Citation	First Author	Journal/Book	Publication Year
73	15366975	The general practice research database: role in pharmacovigilance	Drug Saf. 2004;27(12):871-81. doi: 10.2165/00002018-200427120-00004.	Wood L	Drug Saf	2004
74	23761351	Detection of adverse drug reactions using hospital databases-a nationwide study in Portugal	Pharmacoepidemiol Drug Saf. 2013 Aug;22(8):907-13. doi: 10.1002/pds.3468. Epub 2013 Jun 13.	Miguel A	Pharmacoepidemiol Drug Saf	2013
75	15366974	Data quality management in pharmacovigilance	Drug Saf. 2004;27(12):857-70. doi: 10.2165/00002018-200427120-00003.	Lindquist M	Drug Saf	2004
76	15460169	Pharmacovigilance in the 21st century: new systematic tools for an old problem	Pharmacotherapy. 2004 Sep;24(9):1099-104. doi: 10.1592/phco.24.13.1099.38090.	Szarfman A	Pharmacotherapy	2004
77	16111454	The role of data mining in pharmacovigilance	Expert Opin Drug Saf. 2005 Sep;4(5):929-48. doi: 10.1517/14740338.4.5.929.	Hauben M	Expert Opin Drug Saf	2005
78	15221098	[Databases as a source for monitoring systems of drug safety]	Bundesgesundheitsblatt Gesundheitsforschung Gesundheitsschutz. 2004 Jun;47(6):513-7. doi: 10.1007/s00103-004-0834-3.	Pigeot I	Bundesgesundheitsblatt Gesun	2004
79	20721787	Mining pharmacovigilance data using Bayesian logistic regression with James-Stein type shrinkage estimation	J Biopharm Stat. 2010 Sep;20(5):998-1012. doi: 10.1080/10543401003619056.	An L	J Biopharm Stat	2010
80	16180934	Data mining in pharmacovigilance: the need for a balanced perspective	Drug Saf. 2005;28(10):835-42. doi: 10.2165/00002018-200528100-00001.	Hauben M	Drug Saf	2005
81	17604418	Signal detection in the pharmaceutical industry: integrating clinical and computational approaches	Drug Saf. 2007;30(7):627-30. doi: 10.2165/00002018-200730070-00012.	Hauben M	Drug Saf	2007
82	15360888	A knowledge based approach for automated signal generation in pharmacovigilance	Stud Health Technol Inform. 2004;107(Pt 1):626-30.	Henegar C	Stud Health Technol Inform	2004
83	12650632	Application of quantitative signal detection in the Dutch spontaneous reporting system for adverse drug reactions	Drug Saf. 2003;26(5):293-301. doi: 10.2165/00002018-200326050-00001.	van Puijenbroek E	Drug Saf	2003
84	Yuan Luo	Natural Language Processing for EHR-Based Pharmacovigilance: A Structured Review	Evanston USA	Yuan Luo	This article is a comprehensive	2017
85	ECRI INST	Wrong-Record, Wrong-Data Errors with Health IT Systems.	Pennsylvania	ECRI INSTITUTE PSO	We define data integrity failure	2015
86	European M	Guideline on good pharmacovigilance practices (GVP) Module IX Addendum I – Methodological aspects of signal detection from spontaneous reports of suspected adverse reactions	Netherlands	European Medicines Agency	The decision based on the asses	2015
87	FDA	Questions and Answers on FDA's Adverse Event Reporting System (FAERS)	USA	FDA	What is FAERS? The FDA Ad	2018
88	Liu M et al	Comparative analysis of pharmacovigilance methods in the detection of adverse drug reactions using electronic medical records	New Jersey	Liu M et al	Medication safety requires that	2013
89	Norén GN	A hit-miss model for duplicate detection in the WHO drug safety database	Sweden.	Norén GN et al	We present two new generalisa	2005
90	Niklas Nor	A hit-miss model for duplicate detection in the WHO drug safety database		Niklas Norén et al	We demonstrate the effectivene	2005
91	eudrac	https://www.eudrac.com/news/good-pharmacovigilance-practice-guideline-updates	Germany			
92	PV Drug S	https://www.linkedin.com/posts/pv-drug-safety-academy-841528223_pharmacovigilance-activity-7071440228577660928-KRp9/	England			
93	Drug Regu	https://www.dra.gov.pk/wp-content/uploads/2022/04/Good-Pharmacovigilance-Guidelines-for-Registration-Holders.pdf	Pakistan			
94	cod-researc	https://cod-research.com/ease-icsr-handling-through-early-duplicate-identification-and-its-management/	India			
95	ECA Found	https://www.gmp-compliance.org/guidelines/gmp-guideline/eudralex-volume-9-gvp-module-vi-addendum-i-duplicate-management-of-suspected-adverse-reaction-reports	Germany			
96	Applied Cl	https://www.appliedclinicaltrialsonline.com/view/pharmacovigilance-literature-monitoring-best-practices	Fort Washington USA			
97	ClinosolInd	https://www.slideshare.net/ClinosolIndia/duplicate-case	India			
98	clarivate	https://clarivate.com/blog/seeing-double-eliminating-duplicate-references-in-drug-safety-literature-screening/	Jersey			

For peer review only

Supplementary Table 1: List of 58 papers included in the scoping review.

No.	Author(s)	Year of publication	Journal	Country	Title	Specific Objectives	Study design	Categories						Key findings
								Extent	Prevention	Causes	Detection	Management	Implications	
1	Norén GN	2017	Drug Safety	Sweden	The Power of the Case Narrative - Can it be Brought to Bear on Duplicate Detection?	To discuss the power of the case narrative in duplicate detection.	Commentary				✓			Kreimeyer et al. primarily focus on detecting duplicates in a specific drug or vaccine at a time, with examples in their study typically involving around 1000 reports each. Their research demonstrates the potential value of automated duplicate detection in this context. Manual identification becomes challenging, even with moderately sized case series, with over 400 possible pairs among 30 reports and 5000 pairs among 100 reports. Additionally, not all duplicates are easily identifiable by human assessors, as evidenced by instances of false positives where record pairs initially assumed to be algorithmic errors were later found to be genuine duplicates that had been overlooked.
2	Ramya	2019		Georgia	Duplicate search in pharmacovigilance	To define duplicates, identify their causes, address their handling, confirm their existence, and manage their occurrence.	Web page			✓		✓	✓	The detection and handling of duplicates by National Competent Authorities, Marketing Authorization Holders, and sponsors of clinical trials is an important element of good case management.
3	Likeng Liang *et al .*	2022	Drug Safety	China	Artificial Intelligence-Based Pharmacovigilance in the Setting of Limited Resources.	To review the possible solutions and future perspectives on AI-based pharmacovigilance in resource-limited settings.	Review article					✓		Artificial intelligence (AI) algorithms can process and analyse data relevant to pharmacovigilance. However, the key challenge lies in the initial training of AI algorithms with substantial amounts of high-quality data. Resource-limited settings face notable technical obstacles for AI-driven pharmacovigilance, including the scarcity of robust databases, inadequate human resources, underdeveloped AI technology, and insufficient governmental support. Enhancing the detection capabilities of AI-based pharmacovigilance through improved training and education, along with advocating for the advantages of AI-based approaches to governments, offers a pathway to addressing these challenges in settings constrained by resources. Future advancements in AI-based pharmacovigilance within resource-limited contexts are anticipated to be facilitated by collaborative research networks, advancements in pharmacogenomic research and practices, and the integration of sophisticated machine-learning algorithms. It is imperative to tailor these efforts to the specific contexts of such settings.
4	Manfred Hauben *et al .*	2023	Clinical Therapeutics.	USA	Artificial Intelligence and Data Mining for the Pharmacovigilance of Drug-Drug Interactions.	Despite increasing mechanistic understanding, undetected and underrecognized drug–drug interactions (DDIs) persist. The present article is intended to be part didactic primer and part narrative review of real-world and experimental deployment of AI and data mining activities for pharmacovigilance of DDIs.	Review article					✓		Artificial Intelligence and Data Mining for the Pharmacovigilance of Drug-Drug Interactions. The wide array of intricate and elegant methods has expanded the pharmacovigilance tool kit.

For peer review only

No.	Author(s)	Year of publication	Journal	Country	Title	Specific Objectives	Study design	Categories						Key findings
								Extent	Prevention	Causes	Detection	Management	Implications	
5	Wadhwa *et al*.	2021	Oxford Academic	India	Automation in signal management in pharmacovigilance—an insight.	To review the recent developments of automated systems associated with the detection and prioritization of potential signals with the help of effective healthcare databases to enhance public health safety.	Review article				✓			Automated signal detection is highly based upon the data mining of the spontaneous reporting system such as reports from healthcare professionals, observational studies, medical literature or from social media. The main components of automated signal detection are data extraction, data acquisition, data selection, and data analysis and data evaluation. This system must be developed in the correct format and context, which eventually emphasizes the quality of data collected and leads to optimal decision-making based on scientific evaluation.
6	Adam Lavertu *et al.*	2021	Clinical Pharmacology & Therapeutics	USA	A New Era in Pharmacovigilance: Toward Real-World Data and Digital Monitoring	Several different approaches to modern pharmacovigilance, including spontaneous reporting databases, electronic health record monitoring and research frameworks, social media surveillance, and the use of digital devices. Some of these platforms are well-established while others are still emerging or experimental. We highlight both the potential opportunity, as well as the existing challenges within these pharmacovigilance systems that have already begun to impact the drug development process, as well as the landscape of postmarket drug safety monitoring.	Mini review				✓			The development of massive data sources for future pharmacovigilance efforts creates an opportunity for advances in deep learning and anomaly detection. A continuously learning artificial intelligence system could not only learn to integrate these heterogeneous data sources for real-time case detection but could also help identify potential cases and interface with members of the pharmacotherapy community to gather more information when needed.
7	Rodrigo Postigo *et al*.	2018	Drug Safety	UK	EudraVigilance Medicines Safety Database: Publicly Accessible Data for Research and Public Health Protection	The main objective of this article is to describe the new access to the database together with the opportunities that this new access can bring for research. It is intended to promote an appropriate use of the data to support the safe and effective use of medicines.	Special article		✓					The European Union legislation gives the EudraVigilance (EV) database the core role of managing reports of suspected adverse reactions and determine changes in the safety profile of medicinal products used in medical practice. The EV has demonstrated its value especially in the area of signal detection, providing a tool to analyse the safety data submitted.
8	Jenna Wong *et al*	2022	Drug Safety	Switzerland	Applying Machine Learning in Distributed Data Networks for Pharmacoepidemiologic and Pharmacovigilance Studies: Opportunities, Challenges, and Considerations.	In this paper, we discuss opportunities, challenges, and considerations for applying Machine Learning (ML) in Distributed Data Networks (DDNs) for pharmacoepidemiologic and pharmacovigilance studies. First, we discuss the major types of activities performed by DDNs and how ML may be used. Next, we discuss practical data-related factors infuencing how DDNs work in practice. We then combine these discussions and jointly consider how opportunities for ML are afected by practical data-related factors for DDNs, leading to several challenges. We present diferent approaches for addressing these challenges and highlight eforts that real-world DDNs have taken or are currently taking to help mitigate them. Despite these challenges, the time is ripe for the emerging interest to use ML in DDNs, and the utility of these data-adaptive modeling techniques in pharmacoepidemiologic and pharmacovigilance studies will likely continue to increase in the coming years.	Review article		✓		✓			Using ML in DDNs for pharmacoepidemiology and pharmacovigilance holds great promise. This is an emerging area of interest for DDNs because of the recent methodologic advancements in the feld, and the valuable groundwork that many DDNs have already laid through investments in harmonizing datasets, developing standardised processes, tools, and analytics, and building collaborative relationships between data partners and with various stakeholders. The future holds much promise for the use of ML in DDNs, and we expect that the utility of these data-adaptive methods for enhancing pharmacoepidemiologic and pharmacovigilance studies will likely continue to increase in the years to come.
9	Philip Michael Tregunno *et al*	2014	Drug Safety	UK	Performance of Probabilistic Method to Detect Duplicate Individual Case Safety Reports	The aim of this study was to evaluate probabilistic record matching for duplicate detection, and to characterise the main sources of duplicate reports within each data set.	Original Research Article	✓	✓	✓	✓	✓	✓	Probabilistic record matching, as implemented in vigiMatch, achieved good predictive value for confirmed or likely duplicates in each data source. Most of the false positives corresponded to otherwise related reports; less than 10 % were altogether unrelated. A substantial proportion of the correctly identified duplicates had not previously been detected by national centre activity. On one hand, vigiMatch highlighted duplicates that had been missed by rule-based methods, and on the other hand its lower total number of suspected duplicates to review improved the accuracy of manual review.

For peer review only

No.	Author(s)	Year of publication	Journal	Country	Title	Specific Objectives	Study design	Categories						Key findings
								Extent	Prevention	Causes	Detection	Management	Implications	
10	Benjamin Kompa *et al*	2022	Drug Safety	USA	Artifcial Intelligence Based on Machine Learning in Pharmacovigilance: A Scoping Review	The present study conducted a scoping review of the use of artificial intelligence based on machine learning to understand how it is used for pharmacovigilance tasks, characterize differences with other fields, and identify opportunities to improve pharmacovigilance through the use of machine learning.	Scoping Review				✓	✓		While certain modern practices have begun to appear, many of the primary reasons for the recent success of artificial intelligence (AI) have yet to be translated into pharmacovigilance. Without certain structural changes, PV is unlikely to experience similar kinds of advancements from current approaches to AI.
11	Marin Banovac *et al*	2017	Drug Safety	UK	Patient Reporting in the EU: Analysis of EudraVigilance Data	This descriptive study aims to provide insight into patient reporting for the totality of the EU by querying the EudraVigilance (EV) database for the period of 3 years before the new pharmacovigilance legislation became operational and the 3 years after as well as comparing patient reports with those from healthcare professionals (HCPs) where feasible.	Descriptive study			✓				Patient reporting complemented reporting by HCPs. Reports were submitted for which the patient and HCP were both listed as primary sources. Patients were motivated to report adverse reactions, especially those that affected their quality of life. Sharing these results with National Competent Authorities and patient associations can inform training and awareness on patient reporting.
12	Gianmario Candore *et al*	2022	Drug Safety	Netherlands	The Impact of Mandatory Reporting of Non-Serious Safety Reports to EudraVigilance on the Detection of Adverse Reactions	This paper reports on a series of analyses performed to assess the number and nature of non-serious reports and their effect on signal detection in EudraVigilance.	Descriptive study			✓				The new requirement to report non-serious suspect reactions increased report submissions, improving sensitivity without affecting statistical efficiency. As non-serious reports in EudraVigilance rise, ongoing monitoring is crucial for signal detection impact. Further study is needed to assess the qualitative impact of non-serious reports on signal nature and evaluation.
13	John van Stekelenborg *et al*	2022	Drug Safety	USA	Individual Case Safety Report Replication: An Analysis of Case Reporting Transmission Networks	The present study aimed to investigate the magnitude of replication, the variability among recipients, and the subsequent divergence across recipients of ICSRs.	Original Research Articl						✓	Replication of ICSRs and the variation of available safety information in recipient databases were quantified and shown to be substantial. Multiple processors and medical reviewers will likely handle the same original ICSR as a result of replication. Aside from the obvious duplicate work, this phenomenon could conceivably lead to differing clinical assessments and decisions. If replication could be reduced or even eliminated, this would enable more focus on activities with a benefit for patient safety.
14	Rachel L Richesson *et al*	2008	Drug Safety	USA	An Automated Standardized System for Managing Adverse Events in Clinical Research Networks	We describe an Adverse Event Data Management System (AEDAMS) that is used across multiple study designs in the various clinical research networks and multi-site studies for which we provide data and technological support. Investigators enter AE data using a standardized and structured web-based data collection form	Author manuscript		✓			✓		The automated AEDAMS promptly reports adverse events (AEs) to various parties, enhancing participant safety oversight, especially in multi-site protocols. Standardized AE reporting in clinical research networks enables centralized monitoring, potentially improving agent safety comprehension across studies. Automated systems offer speed, efficiency, and scalability, impacting patient safety and streamlining research processes. Future studies should rigorously evaluate AE management systems, measuring efficiency, reliability, accuracy, and potential reductions in patient morbidity and mortality resulting from improved communication and decision-making. Such evaluations would facilitate faster application of investigational treatments and enhance overall research quality and safety.

For peer review only

No.	Author(s)	Year of publication	Journal	Country	Title	Specific Objectives	Study design	Categories						Key findings
								Extent	Prevention	Causes	Detection	Management	Implications	
15	Michele Fusaroli *et al*	2021	Frontiers in Pharmacology	Italy	Development of a Network-Based Signal Detection Tool: The COVID-19 Adversome in the FDA Adverse Event Reporting System	To propose a network-based approach on co-reported events to help assess disproportionalities and to effectively identify, in a timely manner, the disease-, comorbidity- and drug-related syndromes, especially in a rapidly changing low-resources environment such as that of COVID-19.	Brief research report article.				✓			The Adversome detects plausible new signals and iatrogenic syndromes. The network approach complements traditional pharmacovigilance analyses and may represent a more effective signal detection technique to guide clinical recommendations by regulators and specific follow-up confirmatory studies.
16	Rave Harpaz *et al*	2010	BMC Bioinformatics.	USA	Mining multi-item drug adverse effect associations in spontaneous reporting systems.	This paper examines the application of a well-established data mining method known as association rule mining and demonstrates its value.	Research article.		✓		✓	✓	✓	Our findings demonstrate that multi-item ADEs are present and can be extracted from the FDA's adverse effect reporting system using our methodology, suggesting that our method is a valid approach for the initial identification of multi-item ADEs. The study also revealed several limitations and challenges that can be attributed to both the method and quality of data.
17	F Martin-Sanchez *et al*	2014		Australia	Big data in medicine is driving big changes.	To summarise current research that takes advantage of "Big Data" in health and biomedical informatics applications.	Review article		✓	✓	✓	✓	✓	The survey highlights ongoing development of powerful new methods for turning large-scale, and often complex, data into information that provides new insights into human health, in a range of different areas. Consideration of this body of work identifies several important paradigm shifts that are facilitated by big data resources and methods in clinical and translational research, from hypothesis-driven research to data-driven research, and in medicine, from evidence-based practice to practice-based evidence. The increasing scale and availability of large quantities of health data require strategies for data management, data linkage, and data integration beyond the limits of many existing information systems. As our ability to make sense of that data improves, the value of the data will continue to increase. All areas of biomedicine stand to benefit from Big Data and the associated technologies.
18	Marie Lindquist	2004	Drug Safety	Sweden	Data Quality Management in Pharmacovigilance.	To describe the first three steps of the data processing cycle (collection, entry, storage) and the different quality dimensions associated with these steps, together with examples relevant to pharmacovigilance data	Leading article.				✓			Ensuring data quality throughout processing is crucial in pharmacovigilance. The implementation of robust data quality management practices is essential to address issues perpetuated through the processing cycle. While regulatory requirements dictate reporting standards, database systems largely manage data quality, often without comprehensive appraisal. Inter-system data exchange poses additional quality risks. However, initiatives like 'good pharmacovigilance practice' and emerging conferences indicate progress toward enhancing data quality across the pharmaceutical landscape. Transparent, well-documented quality systems are imperative. Despite acknowledgment of shortcomings in current systems, ongoing efforts aim to reduce errors and misinterpretations, facilitating safer pharmacovigilance processes globally.

For peer review only

No.	Author(s)	Year of publication	Journal	Country	Title	Specific Objectives	Study design	Categories: Extent	Prevention	Causes	Detection	Management	Implications	Key findings
19	Monica A. Muñoz *et al*	2020	Drug Safety	USA	Towards Automating Adverse Event Review: A Prediction Model for Case Report Utility.	This study aimed to develop and validate a model predictive of an individual case safety report (ICSR) pharmacovigilance utility (PVU). Techniques to systematically identify and distinguish higher utility ICSRs from lower utility ones will improve timeliness in managing safety signals.	Research article.				✓			The model demonstrated the feasibility of developing a tool predictive of ICSR utility. The model's modest discriminative ability highlighted opportunities for further enhancement and suggested algorithms tailored to safety issues may be benefcial.
20	José Luis Oliveira *et al*	2013	Pharmacoepidemiology & Drug Safety	Portugal	The EU-ADR Web Platform: delivering advanced pharmacovigilance tools.	To describe the EU-ADR Web Platform built to facilitate accessing, monitoring and exploring pharmacovigilance data, enabling an in-depth analysis of adverse drug reactions risks.	Website		✓		✓	✓		The EU-ADR European project embraces innovative pharmacovigilance research methodologies through the creation of a web platform providing advanced drug data exploration and assessment features. Whereas, in the past, post-marketing drug assessment required intense validation tasks, the in silico pharmacology community is now endowed with the tools required to quickly analyse specific ADRs, further improving drug safety monitoring. The EU-ADR Web Platform enables streamlined access to drug dataset analysis features, including the evaluation of results from EU-ADR workflows and the sharing of data amongst research partners, all within a highly responsive and unique web-based workspace.
21	Helen R. Gosselt *et al*	2022	Pharmacoepidemiology & Drug Safety	Netherlands	Development of a multivariate prediction model to identify individual case safety reports which require clinical review.	Objectives: To develop a prediction model to identify individual case safety reports that require clinical review, including potential signal triggering reports. Secondly, to identify the most important features of these reports.	Original Research Article.						✓	The number of individual case safety reports in pharmacovigilance databases are increasing. New approaches are required to prioritise individual case reports based on their potential signal triggering value since case-by-case review is very time consuming. The authors developed a prediction model using structured data of individual case safety reports to identify potential signal triggering reports in the Netherlands. The authors identified nine most important predictors that were used to identify potential signal triggering reports. The prediction model could be further optimised using additional predictors such as text fields or other unstructured data from case reports.
22	Tomas Lasys *et al*	2023	BJCP	Netherland	Unintended impact of pharmacovigilance regulatory interventions: A systematic review.	This systematic literature review investigated how often the unintended impact of regulatory interventions was considered in publications of studies investigating pharmacovigilance regulatory interventions in Europe.	Review article.			✓				The unintended impact of pharmacovigilance regulatory interventions was reported in only a quarter of identified publications. There was no apparent increase in attention to unintended impact assessments after the update of the Good Pharmacovigilance Practice guidelines.
23	Ruta Mockute *et al*	2017	Drug Safety	USA	Artificial Intelligence Within Pharmacovigilance: A Means to Identify Cognitive Services and the Framework for their Validation.	The aim of this study was to identify areas across the PV value chain that can be augmented by cognitive service solutions using the methodologies of contextual analysis and cognitive load theory.	Review article.		✓					As individual case safety report volumes increase, artificial intelligence can be a means to help mitigate complex decision making for pharmacovigilance professionals. At various decision points in the PV process, cognitive services were identified and developed to assist pharmacovigilance users. These services were validated using a framework leveraging the Acceptance Quality Limit method, to ensure appropriate performance and quality control.

For peer review only

No.	Author(s)	Year of publication	Journal	Country	Title	Specific Objectives	Study design	Categories						Key findings
								Extent	Prevention	Causes	Detection	Management	Implications	
24	Jianxing He *et a*	2019	Nat Med.	China	The practical implementation of artificial intelligence technologies in medicine.	The development of artificial intelligence (AI)-based technologies in medicine is advancing rapidly, but real-world clinical implementation has not yet become a reality. Here we review some of the key practical issues surrounding the implementation of AI into existing clinical workflows, including data sharing and privacy, transparency of algorithms, data standardization, and interoperability across multiple platforms, and concern for patient safety. The study summarizes the current regulatory environment in the United States and highlights comparisons with other regions in the world, notably Europe and China.	Review article.					✓		AI has recently experienced an era of explosive growth across many industries, and the healthcare industry is no exception. Studies across multiple medical specialties have employed AI to mimic the diagnostic abilities of physicians. The hope is that AI may augment the ability of humans to provide healthcare. However, although these technologies are rapidly advancing, their implementation into patient-care settings has not yet become widespread.
25	Juergen Schmide	2018	Clinical Pharmacology & Therapeutics.	USA	Innovation in Pharmacovigilance: Use of Artificial Intelligence in Adverse Event Case Processing.	The current pilot was undertaken to prove the viability of commercially offered machine-learning solutions in the application for case processing. The pilot paradigm was used to simultaneously test the proposed solutions of three commercial vendors for the ability to extract case critical information from source documents to identify valid AE cases after training the machine-learning algorithms with source documents and database content rather than annotated source documents.	Research article.				✓			The pilot was successful in confirming the feasibility of using AI-based tools to support PV operations and in demonstrating the viability of an efficient training method that does not require time-consuming and costly annotations. Finally, the evaluation and scoring method used in the pilot was able to differentiate vendor capabilities and identify vendor 1 as the best candidate to move into the discovery phase.
26	David John Le wis *et al*	2019	PMC	Germany	Utilizing Advanced Technologies to Augment Pharmacovigilance Systems: Challenges and Opportunities.	This paper provides a series of considerations for the applicability of new IT, with explanations of the technology and its applicability within specific domains of the PV system. The overall goal of automation is to provide high-quality safety data in the correct format, in context, more quickly, and with less manual effort.	Rreview article		✓		✓	✓		The rapid advancement of technologies often outpaces regulations governing pharmacovigilance (PV) systems. Yet, intelligent automation offers avenues for system improvement. Owners must ensure the suitability, design, and reliability of advanced technologies, ensuring PV systems remain effective. Trust in intelligent automation is paramount, aiming to deliver safety data accurately and informatively, enhancing evidence quality for optimal scientific evaluation. Alignment with perspectives in biomedical sciences underscores the need for PV system owners to adapt progressively to artificial intelligence, starting with process automation. Algorithmic processing has demonstrated superior performance in binary, repetitive tasks, advocating for its integration into PV systems.
27	European Medicines Agency	2017	Not applicable	Europe	Guideline on good pharmacovigilance practices (GVP): Module VI – Collection, management and submission of reports of suspected adverse reactions to medicinal products (Rev 2)	Section VI.B. of this Module highlights the general principles, based on the pharmacovigilance guidelines E2A, E2B and E2D of the International Council for Harmonisation of Technical Requirements for Pharmaceuticals for Human Use (ICH) (see GVP Annex IV), in relation to the collection, recording and submission of individual reports of suspected adverse reactions associated with medicinal products for human use. The definitions and guidance provided in Section VI.A. and the EU specific requirements presented in Section VI.C. should be followed. All applicable legal requirements are referenced in the way explained in the GVP Introductory Cover Note and are usually identifiable by the modal verb "shall". Guidance for the implementation of legal requirements is provided using the modal verb "should".	Guidelines		✓		✓	✓		Duplicate detection in EudraVigilance – Collaboration between the Agency, competent authorities in Member States and marketing authorisation holders - Duplicate ICSRs submitted to EudraVigilance by the same sender and identified by the Agency.
28	ArisGlobal	2019	Not applicable	USA	ArisGlobal Announces LifeSphere® MultiVigilance 10, Industry's First End-to-End Safety System With Production-Ready Automation.	LifeSphere® MultiVigilance 10 features all new architecture and design, incorporating the latest in cognitive automation technology to deliver groundbreaking efficiency gains to PV teams	Website					✓		Under tightening budgets and evolving global regulations, safety case volumes continue to grow by nearly 15% each year, creating challenges for today's drug safety teams. To address these pressures, the software leverages cognitive computing technologies such as natural language processing and machine learning, providing a single, global database that supports end-to-end automation of all adverse event processing.

For peer review only

No.	Author(s)	Year of publication	Journal	Country	Title	Specific Objectives	Study design	Categories						Key findings
								Extent	Prevention	Causes	Detection	Management	Implications	
29	Rajesh Ghosh *et al*	2020	Pharmaceutical	USA	Automation Opportunities in Pharmacovigilance: An Industry Survey.	This paper aims to address the need for a systematic review framework for automation of the ICSR process from the value, impact, perceived risk, and opportunity point of view.	Original reserch article					✓		In total, 15 member companies of TransCelerate participated in the survey, identifying ICSR process steps with high effort, potential for significant automation benefits, manageable automation risks, and low current automation levels as prime candidates for future automation. These steps include language translations, case verification, quality control, prioritization, data entry, alerting, workflow management, and monitoring. Conversely, certain steps like submission, already automated for some time, show lower potential for further automation. Despite successful adoption of intelligent automation in other domains, its integration into pharmacovigilance, particularly the ICSR process, remains limited, partly due to perceived risks to patient safety, which are expected to decrease with further successful applications.
30	Michael G Kahn *et al*	2016	The Journal for Electronic Health Data and Methods	USA	A Harmonized Data Quality Assessment Terminology and Framework for the Secondary Use of Electronic Health Record Data.	To describe harmonized data quality (DQ) assessment terms, methods, and reporting practices and establish a common understanding of the strengths and limitations of electronic health record (EHR) data for operational analytics, quality improvement, and research.	Review article		✓	✓				A consistent, common DQ terminology, organized into a logical framework, is an initial step in enabling data owners and users, patients, and policymakers to evaluate and communicate data quality findings in a well-defined manner with a shared vocabulary. Future work will leverage the framework and terminology to develop reusable data quality assessment and reporting methods.
31	Tomas Bergvall *et al*	2013	Drug Safety	Sweden	vigiGrade: A Tool to Identify Well-Documented Individual Case Reports and Highlight Systematic Data Quality Issues.	The objective of this study was to propose a measure of completeness and identify predictors of well-documented reports, globally.	Research article.			✓				vigiGrade distinguishes different aspects of quality according to the outline in Lindquist. An important advantage compared with the earlier implementation of documentation grading in VigiBase is that the vigiGrade completeness score considers each dimension in parallel, instead of in sequence: even when information on time-to-onset is lacking. The other dimensions are evaluated and accounted for in the total completeness score. vigiGrade considers many of the same fields as the structured assessment proposed by Agbabiaka et al. but is less comprehensive. Specifically, it does not evaluate dimensions for which absence of information cannot be distinguished from information on absence in VigiBase. On the other hand, it is a scalable solution that allows automated database-wide analyses. By design, it allows for significant penalties of a variety of missing dimensions.

For peer review only

No.	Author(s)	Year of publication	Journal	Country	Title	Specific Objectives	Study design	Categories: Extent	Prevention	Causes	Detection	Management	Implications	Key findings
32	M. Hauben *et al*	2007	Drug Safety	USA	Extreme Duplication' in the US FDA Adverse Events Reporting System Database.	The paper describes duplication as one of the numerous well-recognised forms of data corruption and distortion in spontaneous reporting systems, with duplicate detection algorithms and procedures being included in the commercial data-mining software the authors used.	Research article.				✓	✓	✓	Extreme duplication was discovered within the US FDA Adverse Events Reporting System (AERS) Database. This duplication problem in regulatory and company databases can arise from various sources and a failure to connect related cases. Vendors do make efforts to eliminate duplicate cases before providing data to customers, but sometimes the criteria used may not be sufficient for accurate identification and removal of duplicates. The impact of extreme duplication in AERS, compared to the World Health Organization (WHO) database, is evident, as it affected data-mining results in AERS but not in WHO. Duplicate reports in spontaneous reporting systems may carry unique information value and deserve further investigation. Acknowledging the limitations and challenges of spontaneous reporting system (SRS) data, including inconsistencies, errors, duplications, and incomplete data in critical fields. It's crucial to approach any findings based on SRS data with an appropriate level of caution, taking into account these limitations and the potential impact of duplication on data analysis.
33	CIOMS	2010	Not applicable	Switzerland	Practical Aspects of Signal Detection in Pharmacovigilance.	The objective of the CIOMS VIII report is to provide useful points for consideration to manufacturers, regulatory authorities, international monitoring centers, and others wishing to establish or understand the output of a systematic and holistic strategy to better manage the entire "lifecycle" of a drug safety signal. This lifecycle includes signal detection, signal prioritization, and signal evaluation.	Report			✓	✓		✓	Many databases of large spontaneous reporting systems contain duplicate reports. The identification and elimination of duplicates from analyses, therefore, is advantageous for signal evaluation. However, current duplicate detection procedures, some of which are applied prospectively (i.e. prior to data mining) and others retrospectively (i.e. after data mining), have limitations and enhanced methods of duplicate detection are being developed.
34	Kevin Pozsgai *et al*	2022	Front. Pharmacol.	Hungary	Analysis of pharmacovigilance databases for spontaneous reports of adverse drug reactions related to substandard and falsified medical products: A descriptive study.	To identify and describe the characteristics of cases that are related to adverse drug reactions potentially originating from counterfeit medication using publicly available pharmacovigilance data.	Descriptive case series			✓				The identification and elimination of duplicates between pharmacovigilance databases is almost impossible. Multiple ADRs may be presented for one individual, resulting in report duplications or triplications and making it almost impossible to eliminate this bias with manual analysis.
35	Hung E *et al*	2023	Drug Safety	USA	More extreme duplication in FDA Adverse Event Reporting System detected by literature reference normalization and fuzzy string matching.	The objective of this analysis is to determine if variations of the same literature references observed in FAERS can be resolved with text normalization and fuzzy string matching.	Research article.	✓						Normalized references can be merged via fuzzy string matching to improve enumeration of all the individual case safety reports that refer to the same article. Inclusion of the PubMed ID and adherence to the Vancouver convention could facilitate identification of duplicates in the FAERS dataset. Awareness of this phenomenon may improve disproportionality analysis, especially in areas such as addictovigilance.
36	Uppsala Monitoring Centre	2017	Uppsala Reports	Sweden	Text is key for eliminating duplicate reports.	To describe the role of text in eliminating duplicate reports.	Leading article.			✓				Duplicate Cases – Two or more records describing the same occurrence of events – same adverse effect(s) experienced with the same medical product at the same time – for the same patient. Due to reporting inaccuracies, two records can be considered duplicates even if they do not contain an identical list of events.

For peer review only

No.	Author(s)	Year of publication	Journal	Country	Title	Specific Objectives	Study design	Categories						Key findings
								Extent	Prevention	Causes	Detection	Management	Implications	
37	Uppsala Monitoring Centre		Reporting Fact Sheet	Sweden	Sharing pharmacovigilance data in the WHO Programme for International Drug Monitoring.	To describe the sharing of pharmacovigilance data in the WHO Programme for International Drug Monitoring.	Leading article.			✓	✓	✓		The minimum information required for an ICSR to be valid is a case identifier, reporter and patient information, and information on suspect medicine and reaction/event. All national case reports fulfilling the minimum requirements should be shared with the WHO Programme. However, quality of data and completeness of case reports are always important and reports should include as much information as possible to facilitate assessment. Free text/additional information can be provided in the original language. All information available on the original case report should be included when sending an ICSR to VigiBase, with the exception of confidential patient and reporter details that could potentially be used to identify individuals.
38	European Medicines Agency	2017		Netherlands	Guideline on good pharmacovigilance practices (GVP) Module VI Addendum I – Duplicate management of suspected adverse reaction reports.	The revision contains the following changes: - Alignment with revision 2 of GVP Module VI; - Update of electronic reporting modalities of ICSRs in the new ICH-E2B(R3) format; - Update overall with the revised pharmacovigilance legislation as regards the roles and responsibilities of the Agency, the competent authorities in Member States as well as marketing authorisation holders in relation to the operation of duplicate detection and management of reports of suspected adverse reactions; - Guidance on how to inform the Agency of suspected duplicates in EudraVigilance; - Changes for consistent presentation of GVP document.	Report		✓	✓	✓	✓	✓	Duplicate cases are generally managed through a process of merging two or more cases into one master case. This process can consist of one of the following approaches: The master case can either be based on one of the existing cases, with information from the other subordinate duplicate cases added unless the same, or more precise, information is already present in the master case (this is referred to in this document as “Allocation of a master case”), or; The master case can be created as a new case combining the information from the subordinate duplicate cases (this is referred to in this document as “Creation of a master case”). Regardless of the approach chosen, the master case should always contain all case reference numbers from all subordinate duplicate cases, such that they can be easily traced. The master case should reflect the most accurate and up-to-date information available to the organisation.
39	Agency for Medicinal Products and Medical Devices of Croatia (HALMED)	2008-2013	Not applicable	Croatia	Strengthening Collaboration for Operating Pharmacovigilance in Europe (SCOPE).	SCOPE was divided into eight separate work packages, with five work packages focusing on pharmacovigilance topics to deliver specific and measureable objectives, ranging from improvements in Adverse Drug Reaction (ADR) reporting to the assessment of quality management systems.	Report		✓	✓	✓	✓	✓	The first step in duplicate management is to identify possible ADR report duplicates. Screening for duplicates is usually carried out when a new report arrives in the database, i.e. during data entry or during the process of loading ICSRs that have been received electronically. Duplicates can be detected even before entering the case into the database – that is usually the case during manual data entry of cases received by paper, fax or phone. Duplicates can also be detected during periodic data review and the signal management process when detailed analysis of cases is performed.

For peer review only

No.	Author(s)	Year of publication	Journal	Country	Title	Specific Objectives	Study design	Categories						Key findings
								Extent	Prevention	Causes	Detection	Management	Implications	
40	Ritesh Bhangale *et al*	2017	Perspect Clin Res	India	A day in the life of a pharmacovigilance case processor.	The paper describes the routine experiences of a PV case processor.	Leading article.		✓					For the case processor, every case is different, even within the same study. In fatal cases, the case processor will have to check many things such as the cause of death and autopsy details, whereas in other scenarios, the processor will have to check adverse events of special interest and important medical events and report the same to stakeholders. The case processor should ensure that there are no grammatical/spelling errors in the case, while routing it to the next workflow, and shares the best practices, which helps other associates to do their job efficiently.
41	Priyanka Paygude *et al* .	2013	International Journal of Modern engineering Research	India	Automated Data Validation Testing Tool for Data Migration Quality Assurance.	The paper proposed automation of the data migration validation testing process for quality assurance and risk control across industries.	Research article.		✓					Data migration projects pose significant challenges and risks, e.g., time overruns and budget constraints. The proposed system ensures data quality through standardized testing methods across the enterprise. This solution could help organizations save time, reduce costs, minimize manual efforts, and guarantee data quality assurance throughout the migration process.
42	Saqib Hakak *et al*	2018	Plos One	Malaysia	A new split based searching for exact pattern matching for natural texts.	This work proposes a novel idea to achieve both time efficiency and memory consumption by splitting query string for searching in Corpus.	Research article.				✓			This paper proposed a novel idea for exact string matching to achieve both time and space efficiency regardless of query pattern length, dataset size and scripts.
43	Christen P	2012	SpringerLink.	UK	Data matching: concepts and techniques for record linkage, entity resolution, and duplicate detection. Springer: Data-centric systems and applications.	First book on a topic of growing importance for applications. Brings together research from various areas like databases, statistics, information retrieval, data mining, and machine learning. Details the data matching process step by step. Includes an overview of freely available data matching systems and a detailed discussion of practical aspects and limitations.	Book				✓			By providing the reader with a broad range of data matching concepts and techniques and touching on all aspects of the data matching process, this book helps researchers as well as students specializing in data quality or data matching aspects to familiarize themselves with recent research advances and to identify open research challenges in the area of data matching. To this end, each chapter of the book includes a final section that provides pointers to further background and research material.
44	Liu H *et al* .	2014	EANN	UK	Categorization and Construction of Rule Based Systems.	This paper aims to introduce the theory of rule-based systems especially on categorization and construction of such systems from a conceptual point of view. This also details rule-based systems for classification tasks.	Conference paper				✓			This introduces novel insights for both researchers and practitioners, presenting a new paradigm for rule-based systems in various applications. It advocates for a data-driven approach within machine learning for constructing complex systems, contrasting with traditional knowledge-based methods. Emphasizing the significance of the data-driven approach, it delves into a specialized classification rule-based system, detailing its construction framework and crucial considerations in method selection for rule generation, simplification, and representation operations.
45	Wong CK *et al*	2015	Drug Safety	Australia	Standardisation of the FAERS database: a systematic approach to manually recoding drug name variants	The aim of this study was to develop and implement a data cleaning protocol to identify and resolve drug nomenclature issues. The key 'data treatment' plan involved standardising drug names held in the FAERS database.	Research article.				✓	✓		The millions of reports enclosed in the FAERS contain valuable information that is of interest to pharmacovigilance, toxicology and post-marketing surveillance researchers. With the standardisation of the drug nomenclature, the database can be better utilised by research groups around the world.

For peer review only

No.	Author(s)	Year of publication	Journal	Country	Title	Specific Objectives	Study design	Categories						Key findings
								Extent	Prevention	Causes	Detection	Management	Implications	
46	Jae Dong Yang.	1997	Data & Knowledge Engineering	South Korea	A fuzzy match framework for rule-based programming.	To propose a framework to support a semantic based inexact match with Fuzzy Match Predicate (F_MP).	Research article.				✓			The paper demonstrates that: (1) F_MP is a uniform framework to provide the rule-based languages with fuzzy match facilities semantically enhanced, and that (2) its semantics conform well to those of the relational one.
47	Kreimeyer K *et al*	2022	Drug Safety	USA	Increased Confidence in Deduplication of Drug Safety Reports with Natural Language Processing of Narratives at the US Food and Drug Administration.	To improve and optimize an existing deduplication algorithm that used both structured and free-text data; develop a web-based application to support data processing; and conduct a 6-month dedicated evaluation to assess the potential operationalization of the deduplication process in the FDA.	Research article.				✓	✓	✓	Comparing algorithm predictions with reviewer determinations of duplicates for twenty-seven files for case series reviews (with a median size of 281 reports), the average pairwise recall and precision were 0.71 (SD ± 0.32) and 0.67 (SD ± 0.34). Overall, reviewers felt confident about the algorithm and expressed their interest in using it. These findings support the operationalization of the deduplication process for case series review as a supplement to human review.
48	Kreimeyer K *et al*	2017	Drug Safety	USA	Using Probabilistic Record Linkage of Structured and Unstructured Data to Identify Duplicate Cases in Spontaneous Adverse Event Reporting Systems.	To develop a probabilistic record linkage algorithm for identifying duplicate cases in the US Vaccine Adverse Event Reporting System (VAERS) and the US Food and Drug Administration Adverse Event Reporting System (FAERS).	Research article.				✓		✓	The algorithm was shown to be effective at identifying pre-linked duplicate VAERS reports. The narrative text was not shown to be a key component in the automated detection evaluation; however, it is essential for supporting the semi-automated approach that is likely to be deployed at the Food and Drug Administration, where medical reviewers perform some manual review of the most highly ranked reports identified by the algorithm.
49	Harpaz R *et al*	2017	Journal of Bioinformatics .	USA	Toward multimodal signal detection of adverse drug reactions.	Improving mechanisms to detect adverse drug reactions (ADRs) is key to strengthening post-marketing drug safety surveillance. Signal detection is presently unimodal, relying on a single information source. Multimodal signal detection is based on jointly analyzing multiple information sources. Building on, and expanding the work done in prior studies, the aim of the article is to further research on multimodal signal detection, explore its potential benefits, and propose methods for its construction and evaluation.	Research article.				✓			The results support the notion that utilizing and jointly analyzing multiple data sources may lead to improved signal detection. Given certain data and benchmark limitations, the early stage of development, and the complexity of ADRs, it is currently not possible to make definitive statements about the ultimate utility of the concept. Continued development of multimodal signal detection requires a deeper understanding of the data sources used, additional benchmarks, and further research on methods to generate and synthesize signals.
50	Kreimeyer K *et al*	2021	Computers in Biology and Medicine	USA	Feature engineering and machine learning for causality assessment in pharmacovigilance: Lessons learned from application to the FDA Adverse Event Reporting System.	To support the automated classification of Food and Drug Administration (FDA) Adverse Event Reporting System (FAERS) reports for their usefulness in assessing the possibility of a causal relationship between a drug product and an adverse event.	Research article.				✓			Causal inference from FAERS reports depends on many components with complex logical relationships that are yet to be made fully computable. Efforts focused on readily addressable tasks, such as quickly eliminating unassessable reports, fit naturally in SE's thought processes to provide real enhancements for FDA workflows.
51	Kim HR *et al*	2022	Medicine	South Korea	Analyzing adverse drug reaction using statistical and machine learning methods: A systematic review.	This systematic review aimed to examine the analytical tools by considering original articles that utilized statistical and machine learning methods for detecting ADRs.	Systematic review				✓			This review provides guidelines on which databases are frequently utilized and which analysis methods can be connected. For statistical analysis, >90% of the cases were analyzed by disproportionate or regression analysis of each spontaneous reporting system or electronic medical record database; for machine learning research, however, there was a strong tendency to analyze various data combinations. Only half of the DrugBank database was occupied, and the k-nearest neighbor method accounted for the greatest proportion.

For peer review only

No.	Author(s)	Year of publication	Journal	Country	Title	Specific Objectives	Study design	Categories						Key findings
								Extent	Prevention	Causes	Detection	Management	Implications	
52	CIOMS	2021	Website	Sweden	Making medicines safer for patients everywhere.	This is the fourth of a series of special newsletters that describe the context of CIOMS activities and the work of its member organizations and partners. It describes what the Uppsala Monitoring Centre (UMC) is doing to support and promote patient safety through effective global pharmacovigilance practice.	Not applicable				✓	✓		UMC promotes collaboration between all stakeholders involved in drug risk management and supports harmonization, good pharmacovigilance practice and the dissemination of new guidelines. CIOMS is an important partner in this regard. New working groups on pharmacovigilance topics are needed, for example to explore the link between pharmacovigilance practices and public health, or the role of pharmacoepidemiology as a tool to assess the impact of emerging adverse effects.
53	MedDRA		Website	Not applicable	MeDRA History	In the 1990s, the International Conference on Harmonisation (ICH) recognized the necessity for standardized medical terminology. At that time, available terminologies like COSTART, WHO-ART, J-ART, H-ARTS, ICD-9, and ICD-9CM lacked the required scope and granularity for regulatory authorities and industry needs. Due to infrequent updates, users often created customized versions, leading to a loss of standardization.	Not applicable					✓		MedDRA was based on terminology belonging to the Medicines and Healthcare products Regulatory Agency (MHRA) of UK (previously named Medicines Control Agency) and was developed using the ICH process by the ICH partners, including WHO.
54	Oracle.	2021	Website	USA	Argus Trusted safety case management.	To describe how to promote efficient case management while reducing the manual effort of a safety and pharmacovigilance team.	Not applicable					✓		Oracle Argus enabled greater and faster insight into product safety, improved productivity, and streamlined and automated safety reporting.
55	Ablebits.	2023	Website	Poland	Ablebits. Ablebits Ultimate Suite.	To describe the tools that can automate most daily tasks in Excel and increase productivity by at least 50%	Not applicable					✓		Finding and removing duplicates in Excel may be a real headache, especially when worksheets contain thousands of rows. Duplicate Remover for Excel does the job quickly and perfectly – it can find and delete duplicates based on one or several key columns, identify unique values, highlight, copy or move the found entries.
56	Brinker AD *et al*	2002	Am J Hematol.	USA	Spontaneous reports of thrombocytopenia in association with quinine: clinical attributes and timing related to regulatory action.	This report includes clinical attributes from the largest case series to date of apparently isolated thrombocytopenia in association with quinine and trends in the receipt of spontaneous adverse event reports to FDA's Center for Drug Evaluation and Research (CDER) for this drug-event combination in relation to regulatory action. In this study, we reviewed reports of spontaneous adverse drug events received by CDER.	Descriptive study						✓	This case series confirms previous smaller series that suggest quinine-associated thrombocytopenia may present rapidly with symptoms of profound thrombocytopenia. Clinicians evaluating patients with new-onset and apparently idiopathic thrombocytopenia should maintain clinical vigilance for ingestion of quinine and elicit a detailed food/dietary supplement history from the patient.
57	Azeroual O *et al* .	2022	Multimodal Technol. Interact.	Germany	A Record Linkage-Based Data Deduplication Framework with DataCleaner Extension.	To make the proposed framework usable we integrated it into a tool that is already used in practice, by developing a prototype of an extension for the well-known DataCleaner. The framework detects and visualises duplicates thereby identifying and providing the user with identified redundancies in a user-friendly manner allowing their further elimination. By removing the redundancies, the quality of the data is improved therefore improving analyses and decision-making. This study makes a call for other researchers to take a step towards the "golden record" that can be achieved when all data quality issues are recognised and resolved, thus moving towards absolute data quality.	Research Article.				✓		✓	This paper, presents a Record Linkage framework consisting of six interconnected steps, i.e., (1) data preparation, (2) search space definition, (3) attribute value comparison, (4) a decision model, (5) clustering of the duplicates and (6) verification. It then transforms into an extension for the DataCleaner tool that follows validation through its application to the real-world RIS. This allows for easy and automatic identification of duplicates stored in the system, with their subsequent elimination. This enriches the original records and thereby contributes to their completeness, thus preparing the data for further processing.

For peer review only

No.	Author(s)	Year of publication	Journal	Country	Title	Specific Objectives	Study design	Categories						Key findings
								Extent	Prevention	Causes	Detection	Management	Implications	
58	Norén GN *et al*.	2007	Data Mining and Knowledge Discovery.	Sweden	Duplicate detection in adverse drug reaction surveillance.	We propose a duplicate detection method based on the hit-miss model for statistical record linkage described by Copas and Hilton, which handles the limited amount of training data well and is well suited for the available data (categorical and numerical rather than free text). We propose two extensions of the standard hit-miss model: a hit-miss mixture model for errors in numerical record fields and a new method to handle correlated record fields.	Research Article.				✓			This paper introduced two generalisations of the standard hit-miss model and demonstrated the usefulness of the extended hit-miss model for automated duplicate detection in WHO drug safety data. The results indicate that the hit-miss model can detect a significant proportion of the duplicates without generating many false leads. Its strong theoretical basis together with the excellent results, should make it a strong candidate for other duplicate detection and record linkage applications.

For peer review only

Preferred Reporting Items for Systematic reviews and Meta-Analyses extension for Scoping Reviews (PRISMA-ScR) Checklist

SECTION	ITEM	PRISMA-ScR CHECKLIST ITEM	REPORTED ON PAGE #
**TITLE**			
Title	1	Identify the report as a scoping review.	1
**ABSTRACT**			
Structured summary	2	Provide a structured summary that includes (as applicable): background, objectives, eligibility criteria, sources of evidence, charting methods, results, and conclusions that relate to the review questions and objectives.	2
**INTRODUCTION**			
Rationale	3	Describe the rationale for the review in the context of what is already known. Explain why the review questions/objectives lend themselves to a scoping review approach.	4,5
Objectives	4	Provide an explicit statement of the questions and objectives being addressed with reference to their key elements (e.g., population or participants, concepts, and context) or other relevant key elements used to conceptualize the review questions and/or objectives.	5
**METHODS**			
Protocol and registration	5	Indicate whether a review protocol exists; state if and where it can be accessed (e.g., a Web address); and if available, provide registration information, including the registration number.	5
Eligibility criteria	6	Specify characteristics of the sources of evidence used as eligibility criteria (e.g., years considered, language, and publication status), and provide a rationale.	5
Information sources*	7	Describe all information sources in the search (e.g., databases with dates of coverage and contact with authors to identify additional sources), as well as the date the most recent search was executed.	6
Search	8	Present the full electronic search strategy for at least 1 database, including any limits used, such that it could be repeated.	20
Selection of sources of evidence†	9	State the process for selecting sources of evidence (i.e., screening and eligibility) included in the scoping review.	18
Data charting process‡	10	Describe the methods of charting data from the included sources of evidence (e.g., calibrated forms or forms that have been tested by the team before their use, and whether data charting was done independently or in duplicate) and any processes for obtaining and confirming data from investigators.	6
Data items	11	List and define all variables for which data were sought and any assumptions and simplifications made.	23
Critical appraisal of individual sources of evidence§	12	If done, provide a rationale for conducting a critical appraisal of included sources of evidence; describe the methods used and how this information was used in any data synthesis (if appropriate).	na

For peer review only

SECTION	ITEM	PRISMA-ScR CHECKLIST ITEM	REPORTED ON PAGE #
Synthesis of results	13	Describe the methods of handling and summarizing the data that were charted.	6-7
**RESULTS**			
Selection of sources of evidence	14	Give numbers of sources of evidence screened, assessed for eligibility, and included in the review, with reasons for exclusions at each stage, ideally using a flow diagram.	7
Characteristics of sources of evidence	15	For each source of evidence, present characteristics for which data were charted and provide the citations.	7
Critical appraisal within sources of evidence	16	If done, present data on critical appraisal of included sources of evidence (see item 12).	na
Results of individual sources of evidence	17	For each included source of evidence, present the relevant data that were charted that relate to the review questions and objectives.	7-10
Synthesis of results	18	Summarize and/or present the charting results as they relate to the review questions and objectives.	7-10
**DISCUSSION**			
Summary of evidence	19	Summarize the main results (including an overview of concepts, themes, and types of evidence available), link to the review questions and objectives, and consider the relevance to key groups.	11-13
Limitations	20	Discuss the limitations of the scoping review process.	na
Conclusions	21	Provide a general interpretation of the results with respect to the review questions and objectives, as well as potential implications and/or next steps.	13
**FUNDING**			
Funding	22	Describe sources of funding for the included sources of evidence, as well as sources of funding for the scoping review. Describe the role of the funders of the scoping review.	13

JBI = Joanna Briggs Institute; PRISMA-ScR = Preferred Reporting Items for Systematic reviews and Meta-Analyses extension for Scoping Reviews.

* Where *sources of evidence* (see second footnote) are compiled from, such as bibliographic databases, social media platforms, and Web sites.

† A more inclusive/heterogeneous term used to account for the different types of evidence or data sources (e.g., quantitative and/or qualitative research, expert opinion, and policy documents) that may be eligible in a scoping review as opposed to only studies. This is not to be confused with *information sources* (see first footnote).

‡ The frameworks by Arksey and O'Malley (6) and Levac and colleagues (7) and the JBI guidance (4, 5) refer to the process of data extraction in a scoping review as data charting.

§ The process of systematically examining research evidence to assess its validity, results, and relevance before using it to inform a decision. This term is used for items 12 and 19 instead of "risk of bias" (which is more applicable to systematic reviews of interventions) to include and acknowledge the various sources of evidence that may be used in a scoping review (e.g., quantitative and/or qualitative research, expert opinion, and policy document).

From: Tricco AC, Lillie E, Zarin W, O'Brien KK, Colquhoun H, Levac D, et al. PRISMA Extension for Scoping Reviews (PRISMAScR): Checklist and Explanation. Ann Intern Med. 2018;169:467–473. doi: 10.7326/M18-0850.
